# Synthesis, Anthelmintic Activity, and Mechanism of Action of 5‑Aryl‑1*H*‑indoles

**DOI:** 10.1021/acs.jafc.5c14071

**Published:** 2025-12-16

**Authors:** Alena Kadlecová, Karolina Dzedulionytė Müldür, Miroslav Peřina, Kristýna Bieleszová, Chao Zhang, Daniel Kováříček, Elora Valderas-García, Dominik Vítek, Miglė Valikonytė, Algirdas Šačkus, Joana Solovjova, Vida Malinauskienė, Karel Doležal, Ondřej Novák, Florian M. W. Grundler, Peter Roy, Maria Martínez-Valladares, Jiří Voller, A. Sylvia S. Schleker, Asta Žukauskaitė

**Affiliations:** † Depatment of Experimental Biology, Faculty of Science, Palacký University, Šlechtitelů 27, CZ-77900 Olomouc, Czech Republic; ‡ Department of Organic Chemistry, 70309 Kaunas University of Technology, Radvilėnu pl. 19, LT-50254 Kaunas, Lithuania; § Department of Pharmaceutical Sciences, Division of Pharmaceutical Chemistry, Faculty of Life Sciences, University of Vienna, Josef-Holaubek-Platz 2, A-1090 Vienna, Austria; ∥ Department of Chemical Biology, Faculty of Science, Palacký University, Šlechtitelů 27, CZ-77900 Olomouc, Czech Republic; ⊥ Laboratory of Growth Regulators, Institute of Experimental Botany, The Czech Academy of Sciences & Faculty of Science, Palacký University, Šlechtitelů 27, CZ-77900 Olomouc, Czech Republic; # Instituto de Ganadería de Montaña (CSIC-Universidad de León), Departamento de Sanidad Animal, Grulleros, ES-24346 León, Spain; ∇ Institute of Molecular and Translational Medicine, Faculty of Medicine, Palacký University, CZ-77515 Olomouc, Czech Republic; ○ INRES - Molecular Phytomedicine, University Bonn, Karlrobert-Kreiten-Str. 13, D-53115 Bonn, Germany; ◆ Department of Molecular Genetics & Department of Pharmacology & Toxicology, University of Toronto, The Donnelly Centre, Rm 1202 160 College St., ON M5S 3E1 Toronto, Canada

**Keywords:** indole, parasitic nematodes, C. elegans, mitochondrial complex II, SDH inhibitor

## Abstract

Parasitic nematodes are a significant concern in human and veterinary medicine as well as agriculture. In this study, we prepared twenty-seven 5-phenyl-1*H*-indole derivatives bearing various substituents on the phenyl ring and assessed their efficacy against nematodes. Using *Caenorhabditis elegans*, we selected the most potent compounds and evaluated their toxicity on selected animal and plant-parasitic nematode species. Compounds featuring 4-chloro, 4-fluoro, and 4-trifluoromethoxy groups on the phenyl ring inhibited the motility of exsheathed L3 larvae of *Hemonchus contortus* while exhibiting limited cytotoxicity in mammalian cell cultures. These compounds showed similar effects against the plant-parasitic nematodes *Heterodera schachtii* and *Ditylenchus destructor*, albeit with reduced potency. We propose that the compounds might act as inhibitors of mitochondrial complex II as inferred from molecular modeling, decreased mitochondrial membrane potential, and reduced activity in *C. elegans* complex II mutants.

## Graphical abstract

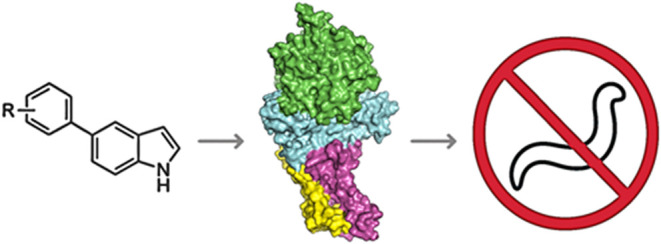

## 1 Introduction

Nematodes are among the most abundant members of the Animalia kingdom, with an estimated population of approximately 4.4 × 10^20^ individuals, roughly 60 billion for every human on Earth.^1^ They exhibit remarkable adaptability across diverse habitats and display a wide range of lifestyles. Parasitic nematodes impact human and animal health, livestock production, and agriculture. In humans, they can cause debilitating diseases such as filariasis and hookworm infections, affecting millions of people worldwide.^2,3^ In livestock, nematode infections negatively affect animal health and reduce productivity, with estimates suggesting annual losses of 1.8 billion EUR in Europe alone.^4^ According to some studies, helminth infections may also exacerbate greenhouse gas emissions in livestock systems.^5,6^ Moreover, plant-parasitic nematodes (PPN) infest agricultural crops, causing significant yield reductions and economic losses.^7^

The control of parasitic nematodes faces two major challenges: the emergence of resistance to anthelmintic drugs in both human^8^ and veterinary medicine^9^ and the environmental and health risks associated with widely used nematicides.^10^ Many nematicides have been banned or gradually phased out in recent decades, creating a need for sustainable alternatives.^11^ Finding new affordable strategies to manage parasitic nematodes while minimizing ecological and health impacts remains a critical challenge in parasitology and agriculture.

To address this need, we screened a portion of our in-house chemical library, which primarily contains phytochemical-inspired structures, for toxicity in the model nematode *C. elegans*. Among the hits, several 5-aryl-1*H*-indoles emerged as possible lead compounds. To the best of our knowledge, this scaffold has not been thoroughly investigated for its nematotoxic potential. Nevertheless, our finding is consistent with previous studies showing that indole, an important interspecies and interkingdom signaling molecule,^12^ and its derivatives can modulate nematode survival and behavior. Numerous natural and synthetic indoles have also been reported to exhibit nematotoxic activity.^13–25^ For example, fungal indole alkaloids such as paraherquamide A, chaetoglobosin A, and marcfortine A are nematotoxic.^13,14^ The semisynthetic spiroindole derquantel was commercialized in 2010 as part of Startect.^15^ Other indolic compound, serotonin, can induce uncoordinated, directionless movement and paralysis in free-living and parasitic nematodes.^16,17^ Camalexin and related phytoalexins contribute to plant defense against nematode infections.^18,19^ Indole-3-acetic acid (IAA) triggers abnormal vacuolization and subsequent cell death through methuosis in *Meloidogyne incognita* although at relatively high concentrations (LC50 117 μg/mL).^20^ Similarly, several synthetic indoles induce vacuolization in nematodes; despite requiring relatively high concentrations, they have been suggested as environmentally friendly nematicides due to their low toxicity to plants, with compounds such as 5-iodoindole showing promising activity against multiple PPN species.^21–25^ Indoles also influence chemotaxis, egg-laying, and survival in *C. elegans*.^25,26^ In addition, indole-containing ascarosides, a class of nematode-specific pheromones, act as aggregation signals.^27^

In this study, we further investigate the nematotoxic properties of the 5-aryl-1*H*-indole scaffold with the goal of exploring its potential as a structurally simple and inexpensive alternative for controlling parasitic nematodes. The employed ligand-free Pd­(OAc)_2_-catalyzed Suzuki-Miyaura cross-coupling efficiently furnishes 5-aryl-1*H*-indoles, and hydroxy-substituted derivatives can be further diversified by *O*-alkylation, providing a practical and efficient route to a broad range of compounds.

## 2 Materials and Methods

### 2.1 Chemistry

#### 2.1.1 General Methods

The reagents and solvents were purchased from commercial suppliers and used without further purification. Water was demineralized using a reverse osmosis system Aqual 29–2 (Aqual, Czech Republic). The microwave irradiation-assisted reactions were performed in a Discover SP microwave reactor (CEM) in 10 mL glass vials that were sealed with silicone/PTFE caps. Reaction progress was monitored by thin-layer chromatography (TLC) on aluminum plates coated with silica gel 60 F254 (Merck); the components were visualized by ultraviolet (UV) light (254/365 nm) and staining solutions (vanillin or potassium permanganate). The purification of the products was performed by column chromatography on silica gel (40–63 μm Davisil LC60 A, Grace Davison, U.K.). The ^1^H and ^13^C NMR spectra were recorded in chloroform-D (CDCl_3_) or dimethyl sulfoxide-*d*_6_ (DMSO-*d*_6_) at room temperature on either Jeol ECA-500 (500 MHz - ^1^H NMR, 125 MHz - ^13^C NMR) or Jeol EC2 400R (400 MHz - ^1^H NMR, 100 MHz - ^13^C NMR) spectrometer equipped with a 5 mm Royal probe (JEOL Ltd., Japan). The LC-MS analyses were done on an ACQUITY UPLC H-Class system combined with a UPLC PDA detector and a single quadrupole mass spectrometer QDa (Waters, U.K.). Melting points were determined using a Büchi B-540 apparatus (BÜCHI Labortechnik AG, Switzerland) and were not corrected. The IR spectra were recorded on a Bruker α (Platinum-ATR sampling module) FTIR spectrometer (Bruker Optics, Germany) by using neat samples.

#### 2.1.2 General Procedure I for Suzuki-Miyaura Cross-Coupling

To a solution of 5-bromo-1*H*-indole (0.70 mmol) in a mixture of ethanol (4.2 mL) and water (1.4 mL), appropriate phenylboronic acid (1.05 mmol), cesium carbonate (456 mg, 1.40 mmol), and palladium­(II) acetate (22 mg, 0.10 mmol) were added under an argon atmosphere. The mixture was stirred at 100 °C under microwave irradiation (50 W) for 30 min. Upon completion, the reaction mixture was cooled to room temperature and filtered through a pad of Celite, and the filter cake was washed with ethyl acetate (20 mL). The residue was diluted with water (20 mL) and extracted with ethyl acetate (3 × 25 mL). The combined organic layers were washed with water (10 mL) and brine (10 mL), dried over anhydrous sodium sulfate, filtered, and evaporated under reduced pressure. The crude was purified by column chromatography on silica gel.

#### 2.1.3 General Procedure II for *O*-alkylation

To a solution of appropriate (1*H*-indol-5-yl)­phenol (0.35 mmol) in dry dimethylformamide (3 mL) were added cesium carbonate (0.70 mmol) and appropriate iodoalkane (0.53 mmol) at 0 °C. The reaction mixture was stirred at room temperature overnight. Upon completion, the reaction mixture was cooled to 0 °C, diluted with water (15 mL), and extracted with ethyl acetate (3 × 20 mL). The combined organic layers were washed with water (1 × 10 mL) and brine (3 × 15 mL), dried over anhydrous sodium sulfate, filtered, and evaporated under reduced pressure. The crude was purified by column chromatography on silica gel.

### 2.2 Biology

#### 2.2.1 Compound Preparation for Bioassays

For bioassays, all compounds were dissolved in dimethyl sulfoxide (DMSO) to prepare 50 mM stock solutions. Stock solutions were stored at −20 °C and diluted in water or the appropriate media for each bioassay to achieve the final concentrations indicated below. Solubility was evaluated by visual inspection of stock solutions (assessing clarity or cloudiness) and by microscopic examination of media to detect potential crystal formation. The final DMSO concentrations in the bioassays were kept at levels that had no detectable impact on test organisms, as determined by comparison with water-treated controls (0.1% for cell cultures, 0.2% for nematodes, and 0.3% for *Arabidopsis thaliana* Col-0).

#### 2.2.2 Nematode Maintenance

*C. elegans* strains used in this study were N2 (wild-type), levamisole-resistant strains ZZ15 (*lev-8*(x15) X) and CB221 (*lev-1*(e211) IV), ivermectin-resistant strain DA1316 (*avr-14*(ad1305) I*; avr-15*(vu227) *glc-1*(pk54) V), mebendazole-resistant strain CB2484 (*ben-1*(e1880) III), and three strains with mutations in succinate dehydrogenase, RP2699, RP2700, and RP2702.^28^ The worms were cultivated on Nematode Growth Medium agar plates seeded with *Escherichia coli* OP50 in 20 °C according to the standard cultivation protocols.^29^ Strains RP2699, RP2700, and RP2702 were generated by Prof. Peter Roy’s group. All other strains and bacteria were purchased from the *Caenorhabditis* Genetics Center (CGC, Minneapolis, MN).

Populations of *D. destructor*, kindly provided by Central Institute for Supervising and Testing in Agriculture, the Ministry of Agriculture of the Czech Republic, were maintained according to the protocol previously described here.^30^

*H. schachtii* was maintained on mustard (*Sinapsis alba* cv. Albatros) roots grown *in vitro* on modified KNOP medium according to the previously published protocols.^30,31^

*H. contortus* L3 were obtained by culturing feces collected from experimentally infected sheep (located in the facilities of the Instituto de Ganadera de Montaa, Leon, Spain) in aerated and humidified closed containers, which were placed in a climatic chamber at 28 °C for 5 days. Larvae were recovered by immersing the feces in tap water for 5 h to stimulate migration. The suspension was then filtered through a 20 μm sieve. Subsequently, larvae were collected using a Baermann apparatus with a 30 μm mesh overnight at 23 °C. Exsheathment of L3 larvae was performed by incubating them in 0.1% (v/v) sodium hypochlorite for 1 h at room temperature on a rotator, followed by three washes with tap water to remove residual bleach.

#### 2.2.3 Experimental Infection of Sheep with *H. contortus*

A 3-month-old Merino lamb was infected with a susceptible isolate of *H. contortus*. For this purpose, the animal was orally administered 20,000 infective third-stage larvae (L3) at the facilities of the Mountain Livestock Institute (Instituto de Ganadera de Montaa), León, Spain. The study protocol was reviewed and approved by the University of León Animal Care Committee, in compliance with current Spanish and European animal welfare legislation (R.D. 53/2013 and EU Directive 2010/63/EU; project code AGL2016–79813-C2–1*R*/2R).

#### 2.2.4 Chitinase Assay

The effect of compounds on *C. elegans* was evaluated using the Chitinase assay as we described previously.^32^

#### 2.2.5 Scoring

Prior to the Chitinase assay and for the evaluation of the effect of fluopyram and compound **i-19** on *C. elegans* wild-type and complex II mutant strains, the experimental plates were evaluated under a microscope, and worm populations in each well received a score from 1 to 5. Dead or mostly immobile worms, arrested at the L1 stage, received a score of 1. Populations with severely delayed development were scored as 2. More developmentally advanced, but still obviously delayed worms were given the score of 3. Adult worms that produce a noticeably lower number of progeny received a score of 4. Healthy worms that reproduce normally (i.e., hundreds of eggs and larvae are present) were scored as 5 (see Figure 1C for representative images). In a typical, correctly prepared experiment, vehicle-treated populations should receive a score of 5, while worms treated with ivermectin (positive control) should receive a score of 1.

**Figure 1:**
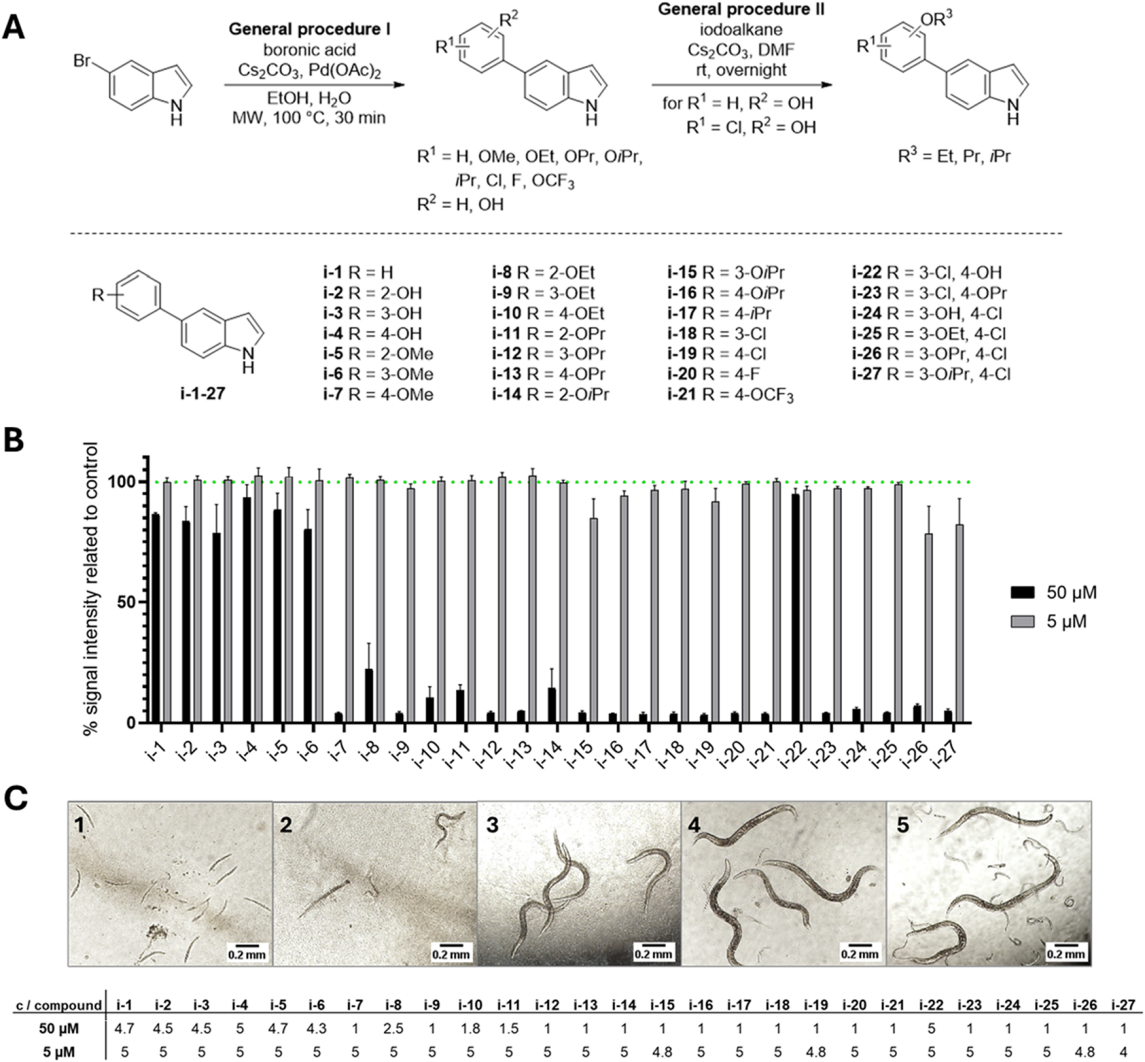
Synthetic sequence and structures of prepared and tested 5-aryl-1*H*-indoles **i-1–27** (A) and their effect in wild-type *C. elegans* (B, C). Most of the compounds reduce the Chitinase activity, a marker of hatching, suggesting diminished vitality and reproductive capacity of the nematodes (B). The graph shows the mean ± SEM of at least three biological replicates (with at least nine independent wells in total). Representative images (C) of the nematode populations receiving scores 1–5 (from left to right). The table shows the average scores (calculated from at least three biological replicates, with at least nine independent wells in total) assigned to the worms treated with the tested derivatives.

#### 2.2.6 Motility Measurements on WMicrotracker ONE

The effect of compounds on motility of *H. contortus*, *D. destructor*, and *H. schachtii* was evaluated using the WMicrotracker ONE platform according to previously published protocols.^30,33^

The effect of compounds on *C. elegans* N2 and SDH mutant strains was measured in a similar manner. Briefly, for evaluation of the acute effect of the compounds, age-synchronized young adults of each strain, kept in S complete medium supplemented with *E. coli* OP50 (3 mg/mL, fresh weight), were transferred onto microtiter plates (96-well, U bottom), and their initial motility was measured for 30 min. Then, the nematodes were treated with compound **i-19**, fluopyram (positive control), or vehicle alone (DMSO 0.2%), and their motility was remeasured on WMicrotracker ONE at the indicated time points. To investigate the recovery rates of pretreated worms, age-synchronized L1 larvae were exposed to **i-19**, fluopyram, or vehicle alone (DMSO 0.2%) for 72 h. Afterward, the compounds were washed away, and worms, supplemented with fresh liquid media and food, were distributed onto 96-well plates and allowed to recover. Their activity was measured using WMicrotracker ONE at the indicated time points.

#### 2.2.7 Nematicidal Assays in Second-Stage Juveniles *H. schachtii*

To assess the nematicidal effect of compounds on *H. schachtii* second-stage juveniles (J2), approximately 50 J2 in sterile double-distilled water were transferred into each well of a flat-bottom 96-well plate and exposed to the test compounds or vehicle alone (DMSO 0.2%). Two days after the treatment, nematodes were agitated by the addition of NaOH (to a final concentration of 62.5 mM), and the surviving (curled) worms were counted under a microscope.^34^

#### 2.2.8 Mitochondrial Membrane Potential Measurement

Age-synchronized *C. elegans* young adults kept in S complete medium supplemented with 3 mg/mL of *E. coli* OP50 were treated with different concentrations of compound **i-19**, fluopyram (positive control), or vehicle alone. After 24 h, the compounds were washed away. Then, the mitochondrial membrane potential was measured using a specialized fluorescent dye (kit MAK147, Sigma-Aldrich). The kit was used according to the manufacturer’s instructions with modified incubation temperature and time (20 °C, 4 h) to account for the fact that living intact worms were used instead of cell cultures.^35,36^ Dyed worms (approximately 200 per well) were distributed onto a black 96-well plate, and the fluorescence was measured using a Tecan Infinite 200 Pro plate reader.

#### 2.2.9 Germination Rate and Phytotoxicity in *A. thaliana*

Wild-type *A. thaliana* (Col-0 ecotype) seeds were sterilized with 70% EtOH with 0.1% Tween-20 solution (2 × 1 mL) for 10 min, rinsed with 96% EtOH (1 × 1 mL) for 10 min, and kept in the fridge (4 °C in the dark). Seeds were sown on a sterile 1/2 MS medium supplemented with 0.3% DMSO as a mock and test compounds at 5, 10, 50, and 100 μM concentrations in 0.3% DMSO. Plates were kept in long-day light conditions (22 °C/20 °C, 16 h light/8 h dark, 100 μmol m^–2^ s^–1^). After 5 days, the germination rate was evaluated. After an additional 5 days, primary root growth was measured with ImageJ software (https://imagej.nih.gov/ij/), and seedlings were weighted.

#### 2.2.10 Resazurin Assay

Skin fibroblasts (BJ) were obtained from the American Type Culture Collection (ATCC, Manassas, VA), while human keratinocytes (HaCaT) were sourced from the German Cancer Research Center in Heidelberg, Germany. Cells were cultured in a standard DMEM medium from Sigma-Aldrich, supplemented with 10% fetal bovine serum, 2 mM glutamine, 100 U/mL penicillin, and 100 μg/mL streptomycin. Cultures were maintained under standard conditions (37 °C, 5% CO_2_, humidified atmosphere) and subcultured two to three times per week. We assessed the effect of 3-day compound treatments on cell viability using a resazurin reduction assay measuring mitochondrial activity. Approximately 5,000 cells in 80 μL of culture medium were seeded into the wells of a 96-well plate. Twenty-four hours later, 20 μL of 5-fold concentrated test compound solutions was added and tested across three concentrations prepared through 2-fold serial dilutions. The highest final concentration was 40 μM. After 72 h, we added an 11-fold concentrated resazurin solution to the wells, achieving a final concentration of 0.0125 μM. Fluorescence (ex = 570 nm, em = 610 nm) was measured after 3 h of incubation.

#### 2.2.11 Molecular Docking

Molecular docking was performed in the cocrystal structures of *Ascaris suum* (*A. suum*) MCII with inhibitors flutolanil (*N*-(3-propan-2-yloxyphenyl)-2-(trifluoromethyl)­benzamide) (PDB: 3VRB) or NN23 (*N*-[(4-*tert*-butylphenyl)­methyl]-2-(trifluoromethyl)­benzamide) (PDB: 4YSX) and appropriate models of *C. elegans* MCII constructed by homologous modeling using Swiss-Model. The three-dimensional (3D) structures of all compounds in several conformations with the lowest energy were prepared using molecular mechanics with Avogadro 1.90.0. Ligands were adjusted, and polar hydrogens were added to ligand and protein with the AutoDock Tools. The 3D structures of flutolanil and fluopyram were extracted from PubChem. The rigid docking was performed using the AutoDock Vina module in PyRx 0.8.^37^ Interactions between ligands and proteins were determined using Biowia Discovery Studio 2021, ver. 21.1.0.20298 (Dassault Systemes, Vélizy-Villacoublay, France), and the figures were generated using Pymol ver. 2.0.4 (Schrödinger, LLC, New York, NY).

#### 2.2.12 DESI-MSI

For in situ visualization of selected compounds, desorption electrospray ionization mass spectrometry imaging (DESI-MSI) was performed by using a Synapt G2-Si MS instrument coupled to a 2D-DESI source (Waters). The spray solvent (95% MeOH with 0.1% ammonia) was delivered at 2 μL/min and nebulized with 0.5 Mbps ultrapure nitrogen on sample slides. The DESI source was optimized as follows: tip-to-surface distance: 1 mm; tip-to-inlet distance: 5 mm; incidence angle: 55°; collection angle: 10°; capillary voltage: 4 kV; cone voltage: 30 eV; mass range: 100–600, with 16 000–17 000 full width at half-maximum (fwhm) mass resolution. Data were acquired in the negative mode using MassLynx software (v4.1, Waters, Milford, MA). Spectra, with a spatial resolution of 30 μm, were acquired every second. All acquired spectra were then recalibrated with the exact mass of the standard compounds and normalized based on root mean square (RMS) intensities. Afterward, acquired MSI data were imported into msIQuant 2.x (Uppsala University, Sweden) for the ion intensity maps generation. DESI-MS/MS was performed on the same sampling area with a 20 eV collision energy scanning of nematodes, and the acquired MS/MS spectra were summarized for the fragmentation ion assignment, and the correct molecular formulas were first calculated using MassLynx software (v4.1) and then identified by their masses acquired from the standard solution.

#### 2.2.13 Data Analysis

Bioassays with nematodes and plants were evaluated using GraphPad Prism and ImageJ. All of the data are displayed as the mean ± the standard error of the mean (SEM). The data were analyzed by one-way analysis of variance (ANOVA) or repeated-measures two-way ANOVA followed by Dunnett’s post hoc test or Tukey’s multiple comparison test.

## 3 Results and Discussion

### 3.1 Synthesis of the Compounds

The synthesis and full characterization of the target compounds are detailed in the Supporting Information file 1 (Figures S1.1–S1.35). In short, to obtain a set of 5-aryl-1*H*-indoles, 5-bromo-1*H*-indole was employed in ligand-free Pd-catalyzed Suzuki-Miyaura cross-coupling, as described previously.^38^ Namely, 5-bromo-1*H*-indole was treated with various arylboronic acids in the presence of cesium carbonate as a base and catalytic amount of palladium­(II) acetate in aqueous ethanol at 100 °C under microwave irradiation furnishing corresponding indole derivatives, designated as **i-1–7**, **i-10**, **i-13**, **i-16–22**, and **i-24**. Subsequently, prepared (1*H*-indol-5-yl)­phenols **i-2–4**, **i-22**, and **i-24** were used as substrates to obtain the rest of the target *O*-alkylated derivatives **i-8–9**, **i-11–12**, **i-14–15**, **i-23**, and **i-25–27**. Overall, 27 compounds **i-1–27** were prepared (Figure 1 A), which were then investigated for their biological activity.

### 3.2 Compounds Reduce Vitality and Reproductive Capacity of both *C. elegans* Wild-Type and Strains Resistant to Several Classes of Anthelmintics

First, we evaluated the activity of all 27 indole derivatives **i-1**–**27** (Figure 1) on *C. elegans*, a free-living model nematode with numerous advantageous characteristics that make it suitable for use in various biological fields.^39^ Due to its convenience, *C. elegans* is also frequently used as a “model parasite”, enabling researchers to study molecular mechanisms of emerging resistance, conduct high-throughput screening for compounds with antinematode activity, and study their mechanism of action.^28,40^

After exposing L1 larvae to the compounds for 4 days, we observed the populations under a microscope and measured the Chitinase activity. The larvae produce the enzyme while hatching, and its activity is indicative of the reproductive capacity of the worms.^41^ Populations that fail to reproduce due to acute or reproductive toxicity or developmental abnormalities are detected. Initially, we screened the compounds in the wild-type N2 strain of *C. elegans* at two concentrations −5 and 50 μM, revealing several compounds to possess promising activity (Figure 1 B).

Worms treated with **i-1–6** and **i-22** displayed almost normal behavior and reproduction. These compounds likely do not have substantial anthelmintic effects, even at the higher concentration tested. At 50 μM concentration, **i-8**, **i-10**, and **i-11** caused worms in some wells to only be delayed in their development, displayed by average scores of 2.5, 1.8, and 1.5, respectively, resulting in a decrease in the reproductive capacity of the population (Figure 1 C). However, since several more promising compounds could be identified in this assay with average scores of 1, the aforementioned less active ones were excluded from further testing. The rest of the compounds were then tested in a wider concentration range to determine their IC_50_.

Microscopic evaluation revealed that in all strains tested, the active compounds were able to immobilize or completely arrest the development of worms at higher concentrations tested, while at concentrations close to the indicated IC_50_ value, the worms were delayed in their development. Based on the results with the *C. elegans* wild-type, we further tested nine compounds with an IC_50_ below 10 μM on mutant *C. elegans* strains resistant to three classes of commonly used anthelmintic drugs (Table 1). Mutations in *lev-1* and *lev-8*, both encoding subunits of the nicotinic acetylcholine receptor, confer resistance to cholinergic agonists such as levamisole.^42,43^ Worms resistant to microtubule destabilizing drugs, benzimidazoles, possess a loss-of-function mutation in gene *ben-1*, encoding β-tubulin.^44^ Lastly, strain DA1316 has mutations in three genes encoding subunits of the glutamate-gated chloride channel^45^ and is highly resistant to avermectins. Although active, **i-17** (IC_50_ in N2 7.60 ± 2.30 = μM) was excluded from further testing due to precipitation of stock solutions after a single freeze–thaw cycle.

**Table 1:** Effect of the Most Active Compounds on Reproductive Capacity (Determined by the Chitinase Assay) of *C. elegans* Wild-Type and Anthelmintic-Resistant Strains^a^

compound	**N2** wild-type	**CB211** *lev-1*(e211) IV.	**ZZ15** *lev-8*(x15) X.	**CB3474** *ben-1*(e1880) III.	**DA1316** *avr-14*(ad1305) I; *avr-15*(vu227) *glc-1*(pk54) V.
**i-9**	9.76 ± 0.93	9.65 ± 1.82	9.71 ± 1.34	10.15 ± 0.50	9.34 ± 2.01
**i-12**	9.60 ± 0.90	8.69 ± 1.26	8.03 ± 1.13	8.32 ± 0.74	7.59 ± 1.99
**i-13**	7.09 ± 0.72	8.80 ± 1.21	7.63 ± 0.91	8.28 ± 0.99	8.45 ± 1.36
**i-15**	7.93 ± 0.66	8.86 ± 0.90	8.55 ± 1.04	10.15 ± 0.51	9.10 ± 2.21
**i-19**	6.67 ± 0.60	6.80 ± 1.95	5.92 ± 0.39	6.61 ± 0.85	5.15 ± 1.01
**i-20**	9.05 ± 0.62	9.02 ± 0.75	10.95 ± 0.51	9.97 ± 1.06	10.42 ± 1.09
**i-21**	8.58 ± 0.99	10.14 ± 0.39	10.58 ± 0.48	9.01 ± 0.78	10.38 ± 0.41
**i-25**	9.74 ± 1.65	9.36 ± 0.43	8.72 ± 0.13	7.79 ± 0.36	9.93 ± 0.68
**i-27**	9.75 ± 1.65	9.85 ± 0.33	8.83 ± 0.47	7.48 ± 0.06	10.79 ± 0.08

a Numerical values indicate the average IC_50_ value ± SEM in μM from at least three repeated experiments (with at least nine independent wells in total).

These results enabled us to draw some basic conclusions regarding the structure–activity relationship of the compounds. While compounds with 2-substitution on the phenyl ring lacked promising activity (inactive at 50 μM), those with substitution at the 4-position generally tended to be more active than the 3-substituted ones. Only in the case of ethoxy- and isopropyloxy-substituted derivatives, namely, 3-substituted **i-9** and **i-15** (IC_50_ < 10 μM) seemed to perform better than their 4-substituted counterparts **i-10** (excluded during the first round of screening due to lack of activity) and **i-16** (IC_50_ in N2 = 15.23 ± 0.89 μM). Overall, among alkyloxy-substituted compounds, 4-propyloxy derivative **i-13** (IC_50_ = 7.09 μM) was the most active, outperforming compounds with shorter aliphatic chains, **i-7** (IC_50_ in N2 = 15.79 ± 1.78 μM) and **i-10**. Halogenated derivatives, especially 4-chlorinated **i-19** (IC_50_ = 6.67 μM), appeared to be the most promising. The better activity of 3-substituted **i-9** and **i-15** compared to their 4-substituted counterparts prompted us to also prepare disubstituted derivatives **i-22**-**27**, which, although generally effective, did not show an apparent improvement compared to compounds with a single substitution. Interestingly, the presence of a hydroxyl group in the molecule completely abolished activity of the compounds, which could only be partially averted by 4-chloro substitution in **i-24** (50 μM > IC_50_ > 25 μM).

The compounds showed comparable activity in the mutant strains and in wild-type worms. Small inconsistencies can likely be ascribed to physiological differences in the resistant strains arising from the mutations they carry. For example, ivermectin-resistant worms (DA1316) are known to be slightly inefficient at food uptake,^45^ and accordingly, we observed them to grow slightly slower than wild-type worms.

#### 3.2.1 Halogenated Derivatives Reduce Motility of Plant-Parasitic Nematodes

Our next step was to confirm whether the preselected active derivatives would also show a toxic effect in actual parasitic nematodes. Although *C. elegans* is undoubtedly a useful tool, especially when screening a larger set of compounds,^40^ there are many biological, physiological, and molecular differences between free-living and parasitic nematode species.

We evaluated the effect of the compounds on the two plant-parasitic nematode species *D. destructor* and *H. schachtii*. *D. destructor* is a migratory endoparasite found mostly in temperate regions, causing significant damage, especially in tuber and root crops. *H. schachtii*, on the other hand, is a sedentary PPN (a group generally considered to be the most economically important^7^) with a wide host range and is distributed worldwide.^46^

We evaluated whether 10 of the most promising compounds (IC_50_ < 10 μM in wild-type *C. elegans*) inhibit the motility of the plant-parasitic nematodes using the WMicrotracker ONE platform.^33^ The instrument evaluates the activity of small animals by counting how many times infrared light microbeams passing through the wells of a microtiter plate are interrupted. In age-unsynchronized populations of *D. destructor*, we observed reduced motility of worm populations treated with the halogenated derivatives **i-19**, **i-20**, and **i-21** (Figures 2A, S2.1A, S2.2A and Table S2.1). After 72 h of treatment with the highest compound concentration (100 μM), the mean activity counts decreased by 62%, 99%, and 57%, respectively. On the other hand, in *H. schachtii* J2, only **i-19** and **i-20** showed statistically significant effects (Figures 2B, S2.1B, S2.2B, Table S2.2). The decreases in mean activity counts after 72 h of treatment at 100 μM were 85% and 97%, respectively.

**Figure 2:**
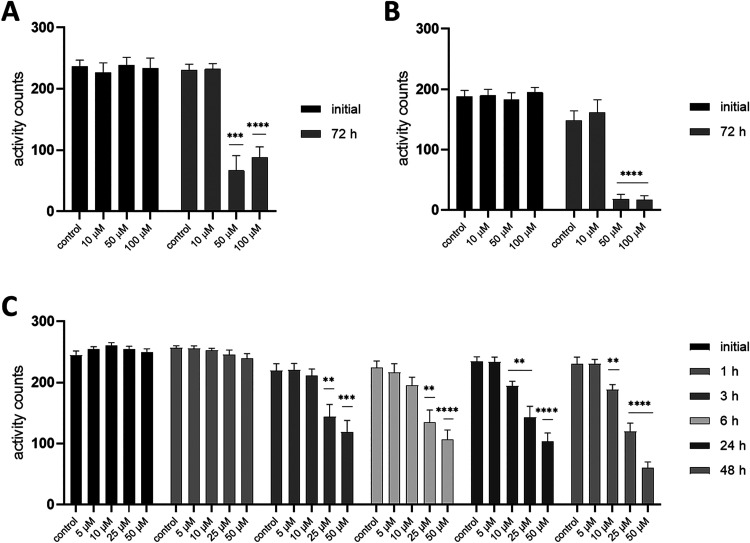
Effect of **i-19** on the motility of age-unsynchronized populations of *D. destructor* (A), *H. schachtii* J2 (B), and xL3 *H. contortus* (C) measured by the WMicrotracker platform. The graphs show average activity counts + SEM from three biological replicates (12 independent wells total). Time point “initial” represents the motility of the population prior to the treatment. Asterisks indicate statistical significance in comparison to vehicle-treated control populations (repeated-measures two-way ANOVA with Dunnett’s multiple comparison test, * *p* < 0.05, ** *p* < 0.01, *** *p* < 0.001, **** *p* < 0.0001).

It was previously demonstrated that dead *H. schachtii* J2 can be easily distinguished from inactive worms by the addition of NaOH,^34^ which causes living larvae to curl up, while dead worms stay straight. Interestingly, when NaOH was added to the wells, most of the worms treated with 50 μM of **i-19** and **i-20** for 48 h displayed some changes in body shape, which indicates that these compounds immobilize worms rather than directly kill them (Figure S2.3).

Alkoxy-substituted derivatives did not show a significant effect on the tested plant-parasitic nematode species. Interestingly, even the disubstituted derivatives containing both the alkoxy chain and chloro substituent in either 4- or 3-positions of the phenyl ring did not significantly decrease the motility of the worms (Tables S1, S2). We presumed the most likely explanation of this lack of effect in comparison to *C. elegans* is probably lower absorption of the compounds, as *C. elegans*, unlike the parasites, feeds during the course of the experiment. Using DESI-MS, we were able to detect signals corresponding to **i-13** and **i-19** in both *C. elegans* and *H. schachtii* (Figures S2.4, S2.5), suggesting that the compounds are at least to some extent absorbed by the parasites as well. However, different nematode species might have different capacities to metabolize the compounds or be inherently more resistant to them. In the future, further exploration of this area might yield valuable insights into, among others, the possible pitfalls of using the model nematode *C. elegans* for PPN-related research.^47^

Our next step was to investigate the effect of the compounds, especially **i-19**, **i-20**, and **i-21**, on the host organism. We tested the effect of the selected derivatives on the growth of the model plant *A. thaliana* wild-type (Col-0) (Figure S2.6, Tables S2.3, S2.4, S2.5). The compounds did not influence the seed germination rates (Figure S2.6A); however, especially at high concentrations, which were needed to inhibit the movement rates of the plant-parasitic nematodes, we observed an inhibition of primary root growth, which also corresponded with a reduction in plant weight (Figure S2.6B, C).

Overall, the data suggest that although the compounds show some potential in protection against plant-parasitic nematodes, further structure optimization would likely be necessary to develop more active compounds that are better tolerated by the plants, as was done, for example, here.^48^

### 3.3 Halogenated Derivatives Reduce Motility of xL3 *H. contortus*

Next, we tested the anthelmintic effect of the compounds on exsheeted *H. controtus* L3 larvae. *H. contortus* is an economically important gastrointestinal parasite of ruminants, found mostly in moist tropical, subtropical, and warm temperate regions. At the beginning of its life cycle, this nematode feeds on bacteria. Only as L3 do they become infectious, and upon being ingested, they establish themselves in the host’s abomasum where they feed on blood. The fact that *H. contortus* is partially free-living makes this species more feasible to work with in the laboratory compared to other mammalian parasitic species, making it an important model for anthelmintic drug discovery.^49^

We mostly focused on the three derivatives containing a halogen substitution in the 4-position of the phenyl ring, namely, **i-19**, **i-20**, and **i-21**. An initial experiment was also conducted with a representative compound with an aliphatic chain, namely, **i-12**, in which we observed only a relatively small effect on the motility of larvae (approximately 30% decrease in motility of nematodes treated with 100 μM of the compound after 48 h in comparison to vehicle-treated worms; Figure S2.7). This suggested to us that, similarly as in plant-parasitic species, compounds with aliphatic chains likely do not reliably affect the worms. On the other hand, all three halogenated compounds significantly reduced the motility of worms in both time- and dose-dependent manner (Figures 2 C, S2.1 C, S2.2 C). The effect was apparent starting at 3 h and beyond. At 48 h, the latest time point was tested, and the movement rates of nematodes exposed to 50 μM of **i-19**, **i-20**, and **i-21** were reduced by 76%, 80%, and 82%, respectively.

As an initial assessment of potential host toxicity, we evaluated the cytotoxicity of the 10 most active derivatives in two noncancerous mammalian cell lines—human primary skin fibroblasts (BJ) and immortalized keratinocytes (HaCaT)—using the resazurin assay^50^ after a 3-day incubation period. Most of the compounds tested showed a favorable toxicological profile (Table S2.6), and their IC_50_ values were far above the highest concentration tested (40 μM). Compound **i-19** showed no effect on the viability of fibroblasts and only a minor toxic effect (decrease of approximately 5% at 40 μM) in the more sensitive keratinocytes. The disubstituted derivatives **i-25** and **i-27** were the most toxic in the set, causing around a 50% decrease in viability of HaCaT cells at 40 μM.

While these findings are promising, further experiments, such as tests in more complex mammalian models and pharmacokinetic and toxicity profiling, would be needed to determine the true potential of the compounds as viable anthelmintic candidates.

### 3.4 Compounds Likely Act as Inhibitors of the Nematode Succinate Dehydrogenase

Determining the mechanism of action of compounds is always challenging; however, the nature of their effect, as well as the structural similarity to compounds with known properties, can provide useful insights. One of our hypotheses was that the compounds might interfere with energy production. We based this assumption on the behavior of compound-treated worms (delayed development, lower fecundity, and relatively rapid decrease in motility rates but not necessarily death). Impaired mitochondrial function would be accompanied by a decrease in mitochondrial membrane potential (MMP).^51^ We observed that exposure of young adult *C. elegans* to **i-19** for 24 h led to a significant dose-dependent decrease in MMP, similar to fluopyram, a known nematicide,^52,53^ which was used as a positive control (Figure S2.8). On average, the signal detected in wells containing nematodes treated with 100 μM **i-19** or fluopyram, the signal was approximately 80% or 85% lower, respectively, than in vehicle-treated control animals. Though all three halogenated derivatives exhibited similar promising activity, we chose **i-19** as a representative in follow-up experiments, as it showed the best activity against *C. elegans* and significantly affected all parasitic species as well.

Furthermore, we noted some shared structural motifs between **i-1–27** and previously described or suspected inhibitors of mitochondrial complex II (MCII, frequently referred to as succinate dehydrogenase, SDH, due to its typical physiological function).^54,55^ The MCII complex is a transmembrane protein composed of four subunits (SDHA-D) that can be found in inner mitochondrial membranes of eukaryotes and in bacteria. The enzyme oxidizes succinate to fumarate and is a crucial part of both the respiratory electron transfer chain and the citric acid cycle. While inhibition of such an important and ubiquitous target can be associated with a risk of significant toxicity to nontarget organisms,^56^ SDH inhibitors have already found application as fungicides^57^ and are also considered a promising target for anthelmintic drug and nematicide discovery. MCII is conserved across the phylum Nematoda but exhibits sufficient structural differences from the enzyme of nontarget organisms.^52^ This already allowed the discovery of inhibitors with excellent activity in nematodes and, at the same time, a very promising safety profile.^28,52,58^

To investigate the binding of **i-1–27** to the quinone-binding site of MCII, which is known from published inhibitors fluopyram and flutolanil,^59,60^ molecular docking modeling was performed. Since there is no experimentally solved structure of *C. elegans* MCII, we initially used the available structures of *A. suum* mitochondrial complex II to assess the potential binding of the compounds to the quinone-binding site.^61^ The *A. suum* protein is composed of four subunits, where the quinone-binding site is constructed by Ip, CybL, and CybS subunits and is located in the mitochondrial inner membrane near the surface of the matrix side (Figure S2.9). The detailed analysis of the known crystal structures of mitochondrial complex II with ligands revealed the differences in the size and shape of the quinone cavity, showing the flexibility of the binding site. The cavity with the natural ligand rhodoquinone (PDB: 5C2T) is much smaller and shallow compared to cavities with inhibitor flutolanil (PDB: 3VRB) or NN23 (PDB: 4YSX). Further, flutolanil induces the formation of a wide bulge at the surface near the entrance, interacting there with its terminal moiety, while in the case of NN23, the entrance remains narrow, with the terminal moiety pointing outward (Figure S2.9A).^59,60^ Hence, molecular docking was performed on both the crystal structure with wide (PDB: 3VRB) and narrow (PDB: 4YSX) entrances to the binding sites. To compare the binding to the *C. elegans* MCII, two corresponding models were constructed using homology modeling (Swiss-model), described as the *C. elegans* *3VRB* model and the *C. elegans* *4YSX* model. Both models were subsequently evaluated and compared with the original *A. suum* crystal structures, confirming a high-level homology in the binding sites. Subsequently, the binding of all derivatives **i-1–27** to the quinone-binding site alongside fluopyram and flutolanil was modeled. The docking protocol was validated by redocking of the ligands and comparison with original cocrystal structures (PDB: 3VRB and 4YSX), which showed RMSD values lower than 0.6 Å, proving high reliability (Figure S2.10). For the best-scored poses in the binding site, the binding energies are listed in Table S7.

The binding energies of the compounds indicated that the crystal structure with a wider opening and bulge (3VRB) provided more favorable binding energies, as in the case of flutolanil and fluopyram, which interact with the surface through their terminal moieties.^59,60^ Methoxy- and ethoxy-substituted **i-6–9** exhibited strong binding affinities toward the 4YSX model, with docking energies around −7.9 kcal/mol, indicating stronger interactions compared to other tested compounds as well as to the reference fluopyram and flutolanil. In contrast, their binding to the 3VRB model was weaker, with docking energies of approximately −8.5 kcal/mol. Conversely, the disubstituted derivatives **i-22–26** showed an opposite trend, displaying stronger binding to the 3VRB model (−9.0 kcal/mol) and weaker interactions with the 4YSX model (−6.8 kcal/mol). When searching for compounds capable of binding effectively to both conformations of the quinone-binding site, halogenated derivatives **i-19–21** emerged as the most promising hits. Among them, **i-19** demonstrated balanced and strong binding energies (−7.6 kcal/mol for 4YSX and −9.0 kcal/mol for 3VRB), comparable to fluopyram (−7.7 and −9.8 kcal/mol, respectively), suggesting its potential as a dual-binding MCII inhibitor (Table S2.7).

Derivative **i-19** effectively bound to the quinone site (Figure S2.11A), with 4*-*chlorophenyl buried deep into the cavity, and established nonpolar interactions with Ser70, Arg74, Pro211, Trp215, His258, and Ile260. The indole ring of **i-19** was pointing out, toward the bulge at the entrance, sandwiched between Trp67 and Trp214 by π–π interactions and mainly by a hydrogen bond of amine hydrogen on the indole ring with the backbone carbonyl of Leu58. It was further stabilized by nonpolar interactions with Thr59 and Tyr61 (Figure S2.11B). Compound **i-19** occupied the same site as flutolanil and displayed similar interactions with respective amino acid residues (including Leu60, Arg 76, Trp69, Tyr107, Trp197, and Ile242 in the original cocrystal structure (PDB: 3VRB) (Figure S2.12C, D)).^59^

In the *C. elegans* homologous model to the *A. suum* crystal structure with NN23 (PDB: 4YSX), described to have a narrow entrance, **i-19** bound in the same way but shallower and with a weaker binding energy (−7.6 kcal/mol). A similar trend was also observed for the standards flutolanil and fluopyram (−7.2 and −7.7 kcal/mol, respectively) and corresponded with binding energies in the original *A. suum* crystal structure (PDB: 4YSX) (Table S2.7). The best pose of **i-19** in the *C. elegans* homologous model displayed very similar interactions in the quinone site. The 4*-*chlorophenyl was buried in the cavity, interacting mainly by nonpolar interactions. On the other hand, the indole ring of **i-19** pointed out of the binding site, and since there was no bulge at the entrance, the hydrogen bond with Leu60 and π–π interactions with Trp67 observed in the 3VRB model were lost (Figure S2.9B).

Given the fact that the MCII is an important part of the respiratory chain and that it is highly conserved, we decided to model the selectivity of **i-19** by docking it to the porcine MCII with bound 2-iodo-*N*-(3-isopropoxy-phenyl)-benzamide (PDB: 3AE7) and avian MCII with bound flutolanil (PDB: 6MYO), both in wide-open conformation. The **i-19** generally displayed impaired binding with clashes leading to similar positions of the ligand but a much lower docking score of – 7.7 and −7.8 for the porcine and avian MCII, respectively, compared to a docking score of −9.0 for the nematode MCII in the same topology of the binding site. These results suggest the possible selectivity of **i-19**, which might be influenced by the different conformation of the residues Trp173B, Trp35C, and Arg46C in nematode and vertebrate MCII, as was described earlier.^59^ Overall, **i-19** demonstrated very promising binding into both possible conformations of nematode MCII, exhibiting similar interactions to flutolanil and its derivative NN23.^59^ It corresponded with a potent decrease in MMP, further supporting its potential as an effective MCII inhibitor and highlighting its suitability as a promising scaffold for future development of potent, parasite-selective inhibitors.

To experimentally support this promising initial finding, we tested the effect of **i-19** against several *C. elegans* MCII mutant strains. These nematodes carry point mutations that result in amino acid changes in regions of subunits SDHC and SDHD that contribute to the formation of the ubiquinone-binding pocket, thereby influencing its shape and the ability of inhibitors to interact with the protein. We observed that nematodes with mutated MCII exhibit higher resistance to **i-19** and to fluopyram, which served as a positive control when compared to wild-type nematodes. The compounds were tested in multiple setups. At first, young adults were exposed to higher doses (100, 50, and 10 μM) of the compounds for up to 48 h, and changes in motility were evaluated using WMicrotracker. For both compounds, we observed a less pronounced decrease in movement rates of MCII mutants compared to wild-type worms, in both a dose- and time-dependent manner (Figure 3).

**Figure 3:**
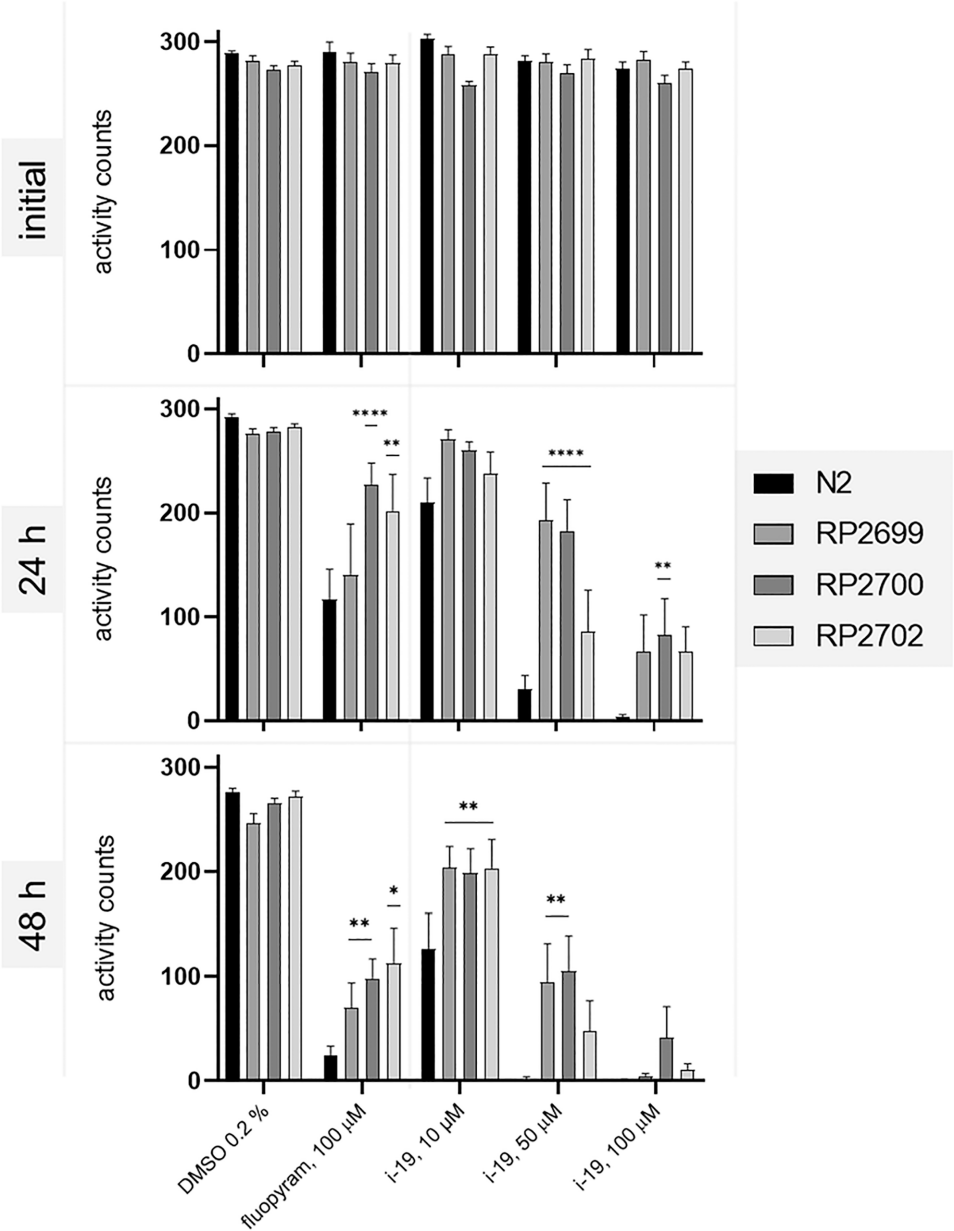
*C. elegans* MCII mutants are more motile than the wild-type after 24 and 48 h exposure to various concentrations of **i-19** and fluopyram. The graphs show average activity counts from three independent experiments, and error bars indicate SEM. Time point “initial” represents the motility of the population prior to the treatment. Asterisks indicate statistical significance in comparison to *C. elegans* wild-type subjected to the same treatment (repeated-measures two-way ANOVA with Tukey’s multiple comparison test, * *p* < 0.05, ** *p* < 0.01, *** *p* < 0.001, **** *p* < 0.0001).

In the case of **i-19**, the difference was the most pronounced at 50 μM. After 24 h of treatment, the mean activity counts decreased by 89% for the wild-type *C. elegans*. In the three MCII mutant strains tested (RP2699, RP2700 and RP2702), the decreases were 46%, 32%, and 70%, respectively. For fluopyram, we observed a statistically significant difference only at 100 μM. The mean activity counts decreased by 60% in the N2 strain and by 53%, 16%, and 28% in the mutants after 24 h.

We noted a relatively low efficacy of fluopyram in our setup in comparison to the previously published data.^28,62^ Fluopyram typically strongly affects wild-type *C. elegans* in concentrations below 1 μM. We attribute this discrepancy to methodological differences: in previous studies, earlier larval stages, longer exposure times, and different evaluation methods, possibly more sensitive than WMicrotracker, were used. To test this, we also performed an assay more closely following the procedure described here,^28^ exposing L1 larvae to **i-19** and fluopyram for 5 days and scoring the plates (see Section 2.2.5) afterward. As expected, this setup required much lower doses of fluopyram to elicit an effect (Figure S2.12A, Table S2.8). All strains were severely affected by fluopyram at concentrations >6.25 μM. At concentrations between 3.13 and 0.01 μM, the three MCII mutant strains grew and developed significantly faster than wild-type *C. elegans*. MCII mutants treated with **i-19** also appeared less susceptible than wild-type worms (Figure S2.12B, table S2.8) although differences were apparent only at higher doses (31.64–13.35 μM).

Lastly, we tested the compounds in a setup resembling that described here.^52^ *C. elegans* L1 larvae were exposed to 5 or 10 μM of the compounds for 72 h, after which the compounds were washed away, and the recovery of the nematodes was evaluated using WMicrotracker. In this assay as well, MCII mutants treated with either compound appeared less affected and tended to recover faster. However, the effect varied among biological replicates and was apparent in only two out of the three complex II mutant strains used (Figure S2.13). While the magnitude of effects varied depending on the assay protocol, all experiments consistently demonstrated a similar trend, namely, increased resistance of the MCII mutants to both fluopyram and **i-19**.

Altogether, both the obtained molecular modeling and experimental data support, albeit indirectly, our hypothesis that 5-aryl-1*H*-indoles act as MCII inhibitors. While the structural diversity of MCII inhibitors is vast,^63,64^ 5-aryl-1*H*-indoles stand out due to their straightforward and readily accessible structures. In the future, we plan to directly assess the interaction between the compounds and the target enzyme. Further insight into the characteristics of the interaction could also inspire future compound design aimed at obtaining more active and selective derivatives.

Lastly, we performed *in silico* ADME analysis using SwissADME.^65^ Compounds **i-19–21** fulfilled the key criteria of drug-likeness according to Lipinski, Veber, and Egan rules, with no major violations observed (Table S2.9). The reference standards flutolanil and fluopyram showed moderate lipophilicity (logP = 3.7 and 4.5, respectively) and moderate water solubility, whereas **i-19–21** exhibited comparable or slightly higher lipophilicity (logP = 3.7 and 4.8) and similar solubility profiles. Both **i-19** and **i-20** displayed low polar surface areas (topological polar surface area, TPSA 12 Å^2^), indicating high gastrointestinal absorption and good predicted blood–brain barrier permeability, while **i-21** showed a higher TPSA (21 Å^2^) but remained within the range associated with high absorption. All of the compounds were predicted to be P-glycoprotein substrates, which may influence efflux and bioavailability, yet they retained bioavailability scores (0.55) similar to those of the standards. Regarding expected metabolic stability, the analogues showed a narrower CYP450 inhibition spectrum than the reference molecules: while fluopyram and flutolanil inhibited multiple CYP isoforms, **i-19–21** were predicted to inhibit CYP1A2 and CYP2D6, suggesting potentially lower off-target metabolic interactions. All compounds demonstrated good synthetic accessibility (1.7–2.4), supporting feasibility for scale-up and field application. While these results would need to be further supported by experimental evidence in the future, the predicted features support the potential of 5-aryl-1*H*-indoles as MCII inhibitors suitable for agricultural or veterinary antiparasitic applications.

## Supplementary Material



## Notes

Additional experimental details on the synthesis and characterization of compounds **i-1–27;** including FTIR, ^1^H NMR, ^13^C NMR; and MS spectra as well as LC–MS chromatograms (DOC, PNG); supporting tables S2.1**–**S2.9; figures S2.1–S2.13 providing additional details on the biological activity of compounds **i-1–27** (PDF)

## Abbreviations

DMSO: dimethyl sulfoxide
DPS: days post seeding
EtOH: ethanol
IAA: indole-3-acetic acid
MCII: mitochondrial complex II
MMP: mitochondrial membrane potential
PPN: plant-parasitic nematodes
SDH: succinate dehydrogenase
SEM: standard error of the mean
TPSA: topological polar surface area
xL3: exsheathed third-stage larvae
