# Supplementary material for: Synthesis, Anthelmintic Activity, and Mechanism of Action of 5‑Aryl‑1H‑indoles
Source: J Agric Food Chem. 2025 Dec 16;73(52):33059–69. doi: 10.1021/acs.jafc.5c14071 (PMC12766724; doi:10.1021/acs.jafc.5c14071)
Supplement: Supplementary file 1 [file jf5c14071_si_001.pdf]

SUPPORTING INFORMATION

Synthesis, anthelmintic activity and mechanism of action of 5-aryl-1*H*-indoles

Alena Kadlecová[1,*], Karolina Dzedulionytė Müldür[2,3], Miroslav Peřina[1], Kristýna Bieleszová[4], Chao Zhang[5], Daniel Kovářı́ček[4], Elora Valderas-García[6], Dominik Vítek[7], Miglė Valikonytė[2], Algirdas Šačkus[2], Joana Solovjova[2], Vida Malinauskienė[2], Karel Doležal[4,5], Ondřej Novák[5], Florian M. W. Grundler[8], Peter Roy[9], Maria Martínez-Valladares[8], Jiří Voller[1,7], A. Sylvia S. Schleker[8], Asta Žukauskaitė[4,*]

[1] Depatment of Experimental Biology, Faculty of Science, Palacký University, Šlechtitelů 27, CZ-77900 Olomouc, Czech Republic

[2] Department of Organic Chemistry, Kaunas University of Technology, Radvilėnų pl. 19, LT-50254 Kaunas, Lithuania

[3] Department of Pharmaceutical Sciences, Division of Pharmaceutical Chemistry, Faculty of Life Sciences, University of Vienna, Josef-Holaubek-Platz 2, A-1090 Vienna, Austria

[4] Department of Chemical Biology, Faculty of Science, Palacký University, Šlechtitelů 27, CZ-77900 Olomouc, Czech Republic

[5] Laboratory of Growth Regulators, Institute of Experimental Botany, The Czech Academy of Sciences & Faculty of Science, Palacký University, Šlechtitelů 27, CZ-77900 Olomouc, Czech Republic

[6] Instituto de Ganadería de Montaña (CSIC-Universidad de León), Departamento de Sanidad Animal, Grulleros, ES-24346 León, Spain

[7] Institute of Molecular and Translational Medicine, Faculty of Medicine, Palacký University, CZ-77515 Olomouc, Czech Republic

[8] INRES - Molecular Phytomedicine, University Bonn, Karlrobert-Kreiten-Str. 13, D-53115 Bonn, Germany

[9] Department of Molecular Genetics & Department of Pharmacology & Toxicology, University of Toronto, The Donnelly Centre, Rm 1202 160 College St., ON M5S 3E1, Toronto, Canada

Section S1 – Chemistry supporting information

Synthesis and characterization of compounds i-1-27

5-Phenyl-1*H*-indole **i-1**

Previously reported in [1]. Prepared in accordance with general procedure **I** from 5-bromo-1*H*-indole (165 mg, 0.84 mmol) and phenylboronic acid (154 mg, 1.26 mmol). Yield 56%, pale yellow solid; mp = 64.7–65.5 °C, R_f = 0.30 (*n*-hexane/ethyl acetate, 4/1). IR (neat) ν_{max}, cm^{-1}: 3409 (N-H), 1463, 1085, 747, 697, 488. ^{1}H NMR (500 MHz, $CDCl_3$): δ 6.63-6.67 (1H, m, CH), 7.22-7.25 (1H, m, CH), 7.33-7.39 (1H, m, CH), 7.46-7.52 (4H, m, 4×CH), 7.68-7.73 (2H, m, 2×CH), 7.90-7.93 (1H, m, CH), 8.11 (1H, br s, NH). ^{13}C NMR (125 MHz, $CDCl_3$): δ 103.1 (CH), 111.4 (CH), 119.4 (CH), 122.0 (CH), 125.0 (CH), 126.5 (CH), 127.5 (2×CH), 128.5 (C), 128.8 (2×CH), 133.5 (C), 135.4 (C), 142.7 (C). MS (pos. mode): m/z (%): 194.1 (M+H$^+$, 100%).

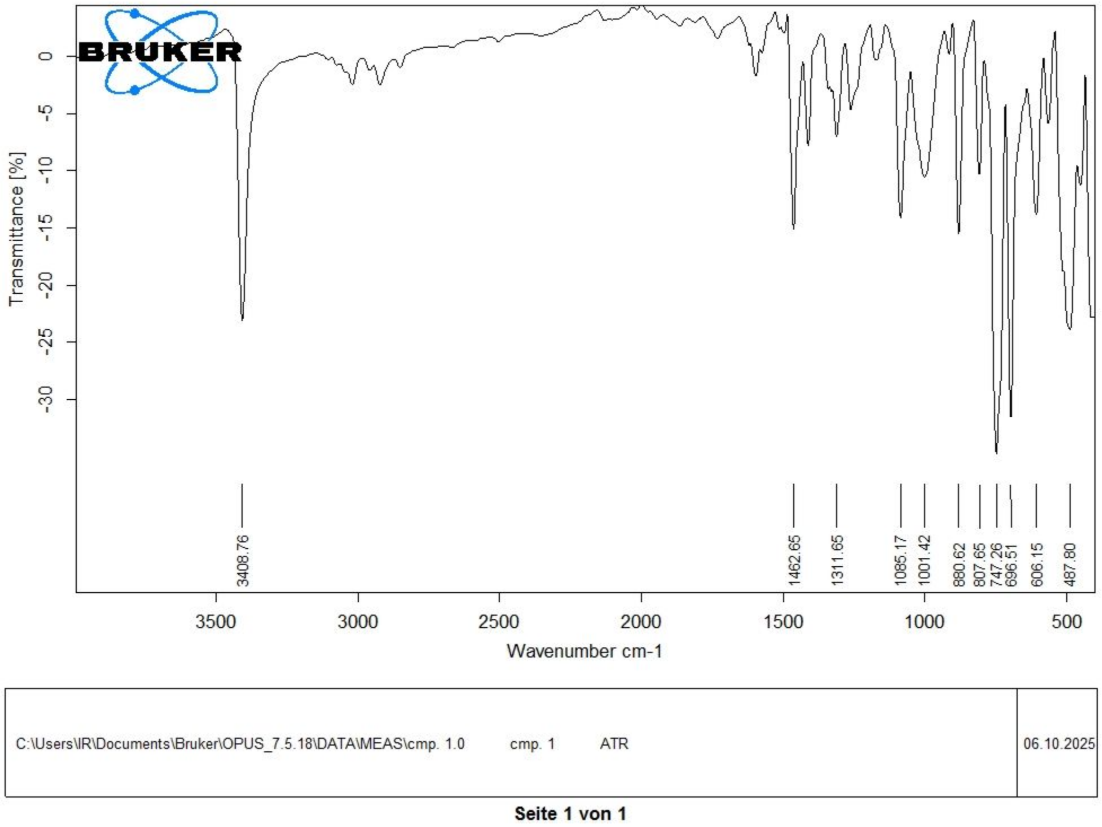

Figure S1.1 FTIR spectrum of compound **i-1**.

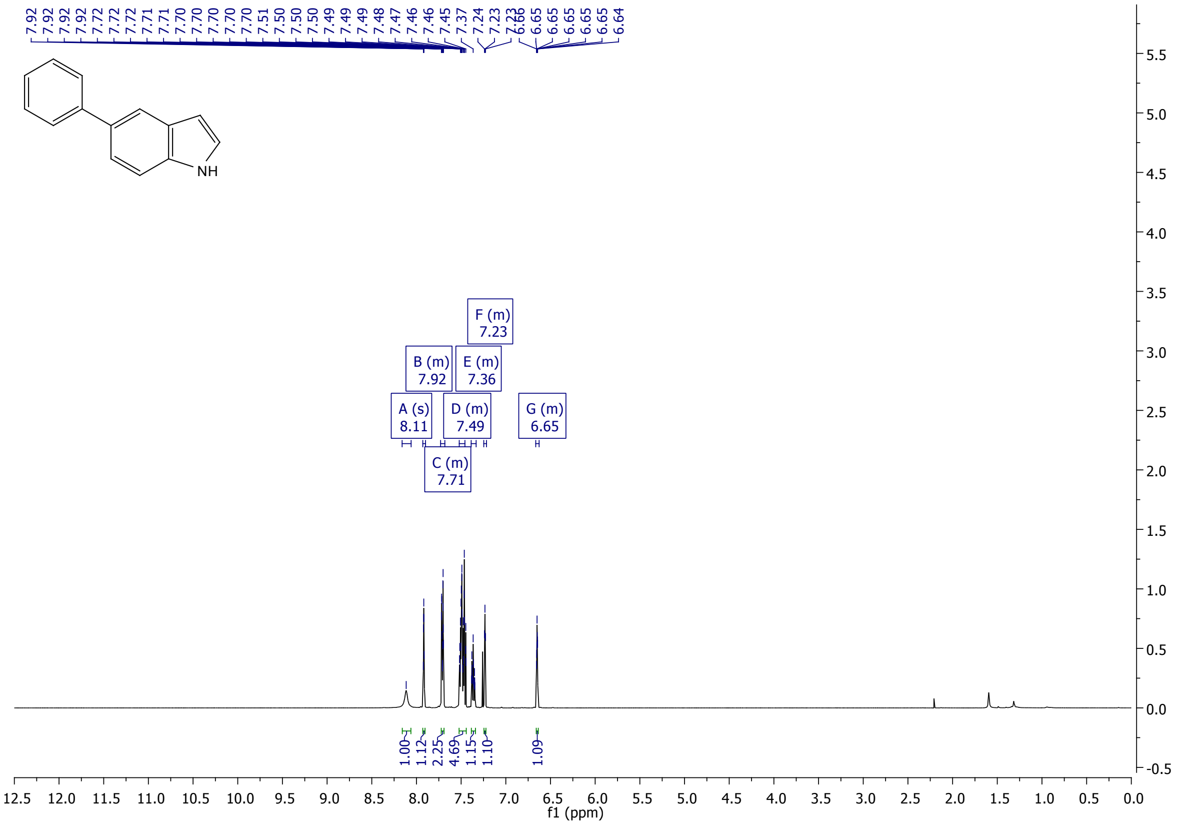

Figure S1.2 ^{1}H NMR spectrum of compound **i-1** in $CDCl_3$.

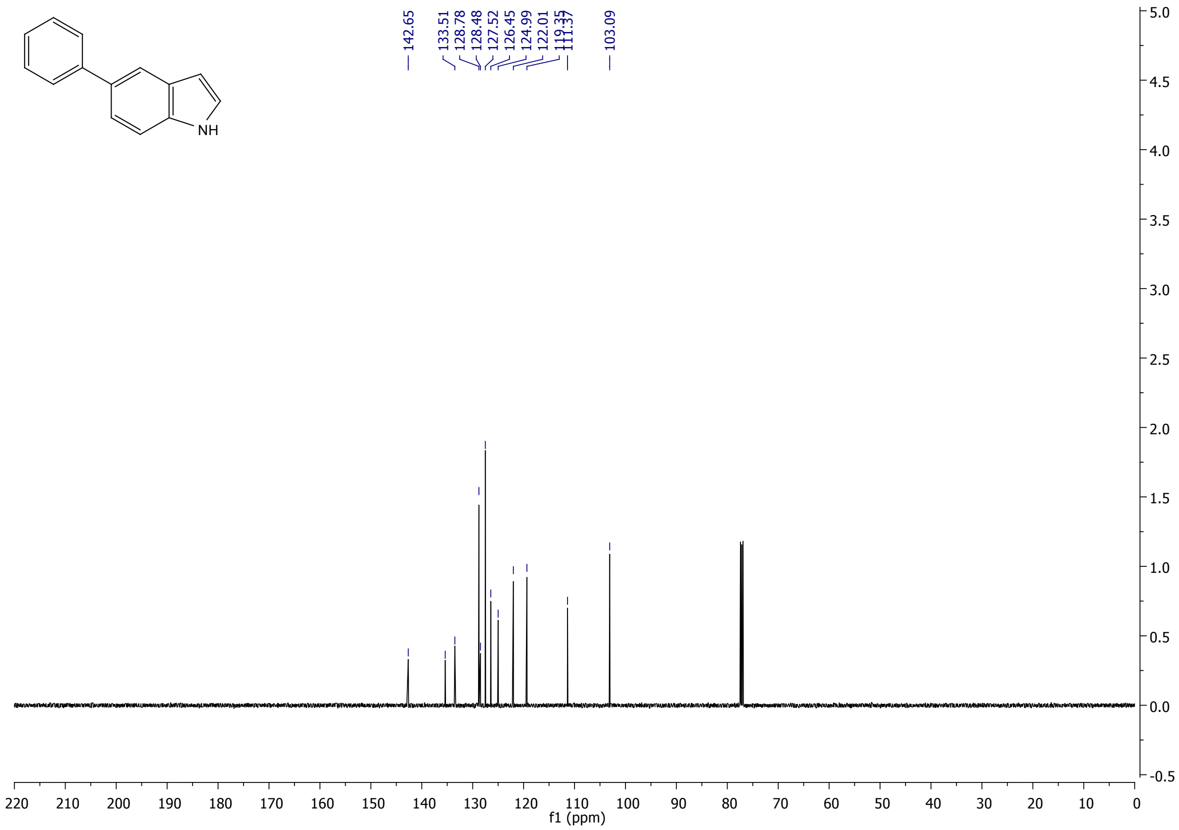

Figure S1.3 ^{13}C NMR spectrum of compound **i-1** in $CDCl_3$.

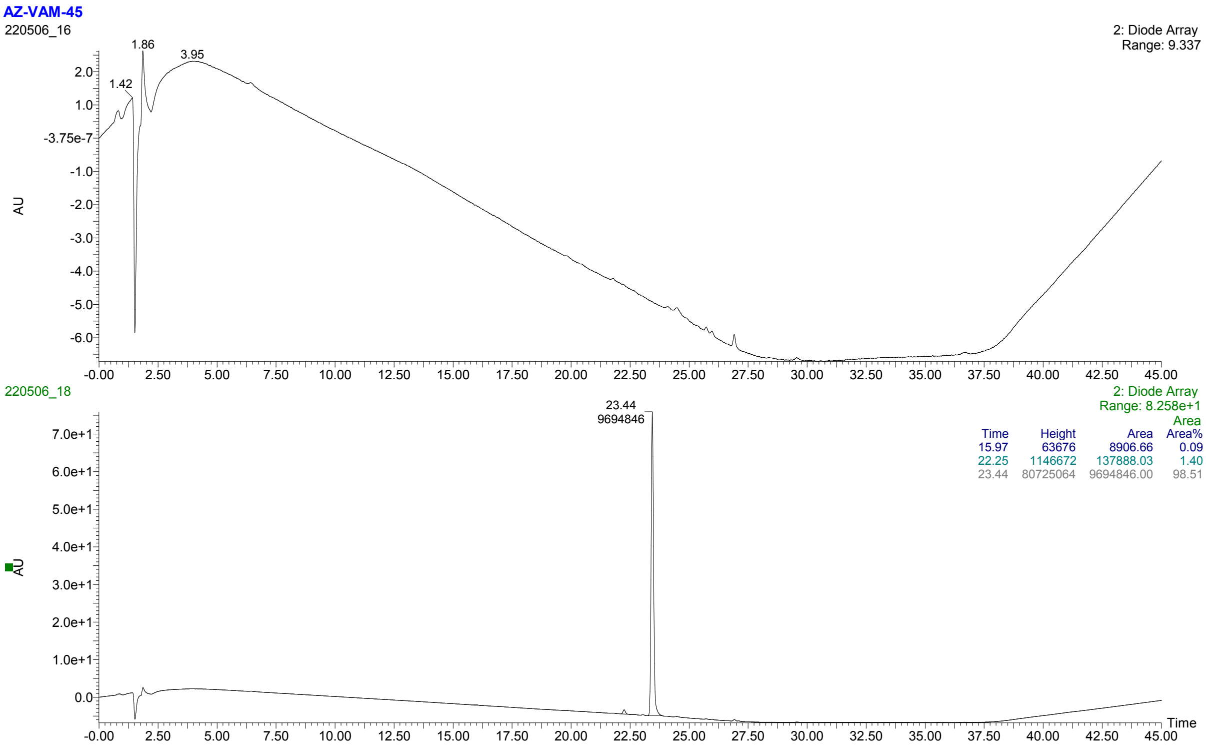

Figure S1.4 LC-MS chromatogram of compound **i-1**.

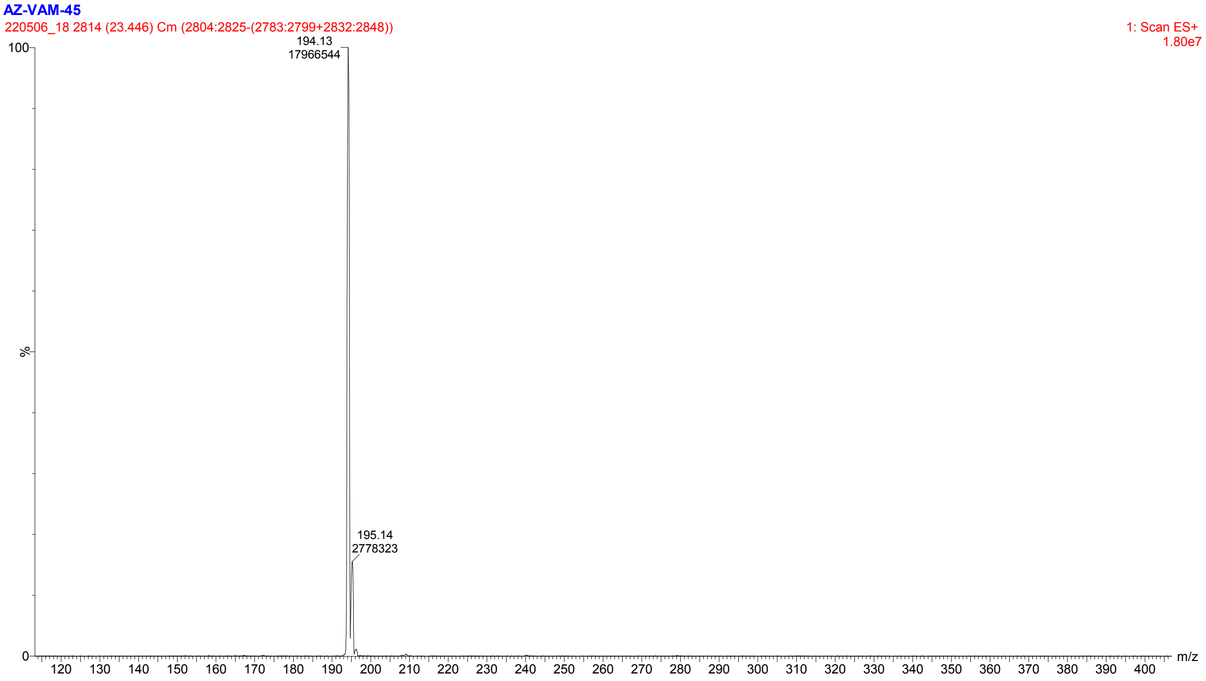

Figure S1.5 MS spectrum of compound **i-1**.

2-(1*H*-Indol-5-yl)phenol **i-2**

Prepared in accordance with general procedure **I** from 5-bromo-1*H*-indole (137 mg, 0.70 mmol) and (2-hydroxyphenyl)boronic acid (145 mg, 1.05 mmol). Yield 75%, pale yellow viscous liquid, $R_f = 0.31$ (*n*-hexane/ethyl acetate, 7/3). IR (neat) ν_{max}, cm^{-1}: 3405 (br, N-H and O-H), 1698, 1464, 1190, 730. ^{1}H NMR (400 MHz, DMSO-d_6): δ 6.42-6.44 (1H, m, CH), 6.82-6.87 (1H, m, CH), 6.90-6.94 (1H, m, CH), 7.07-7.14 (1H, m, CH), 7.23-7.28 (2H, m, 2×CH), 7.33 (1H, t, *J* = 2.7 Hz, CH), 7.38 (1H, d, *J* = 8.4 Hz, CH), 7.65-7.67 (1H, m, CH), 9.28 (1H, s, OH), 11.06 (1H, br s, NH). ^{13}C NMR (100 MHz, DMSO-d_6): δ 101.3 (CH), 110.7 (CH), 115.9 (CH), 119.3 (CH), 120.5 (CH), 122.9 (CH), 125.5 (CH), 127.4 (CH), 127.5 (C), 129.36 (C), 129.41 (C), 130.7 (CH), 134.9 (C), 154.3 (C). MS (pos. mode): m/z (%): 210.2 ($M+H^+$, 100%).

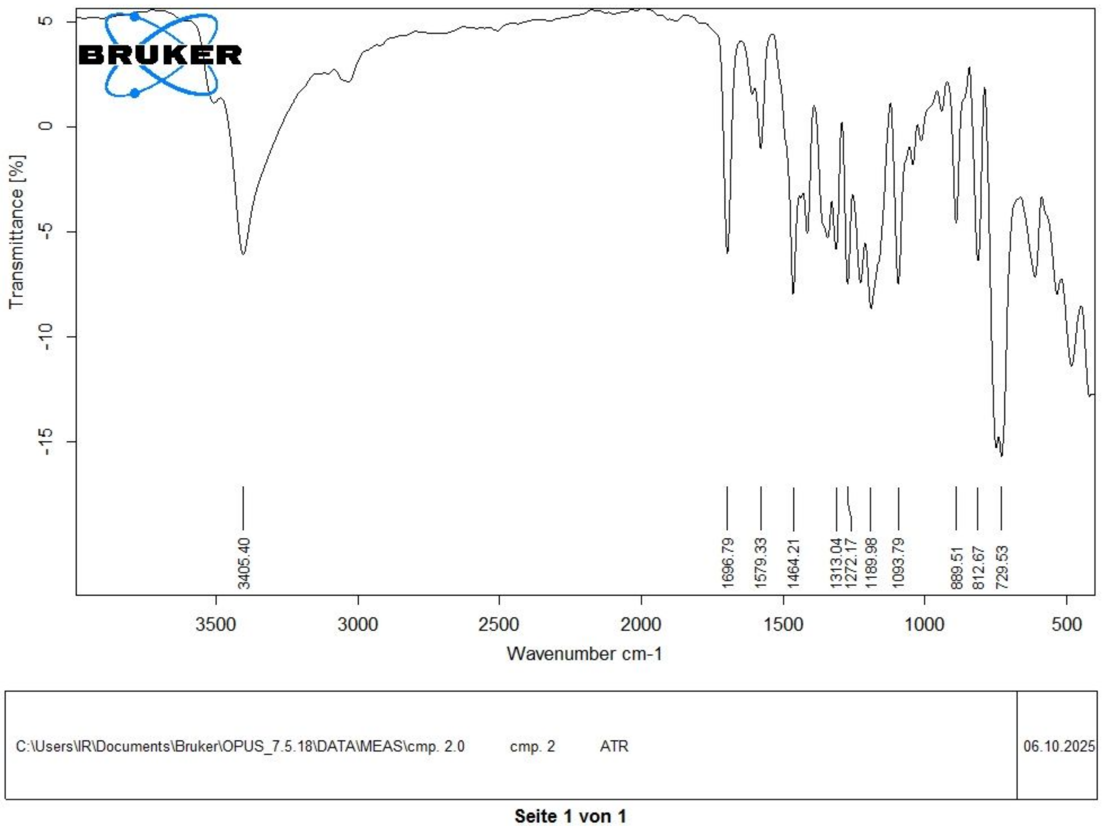

Figure S1.6 FTIR spectrum of compound **i-2**.

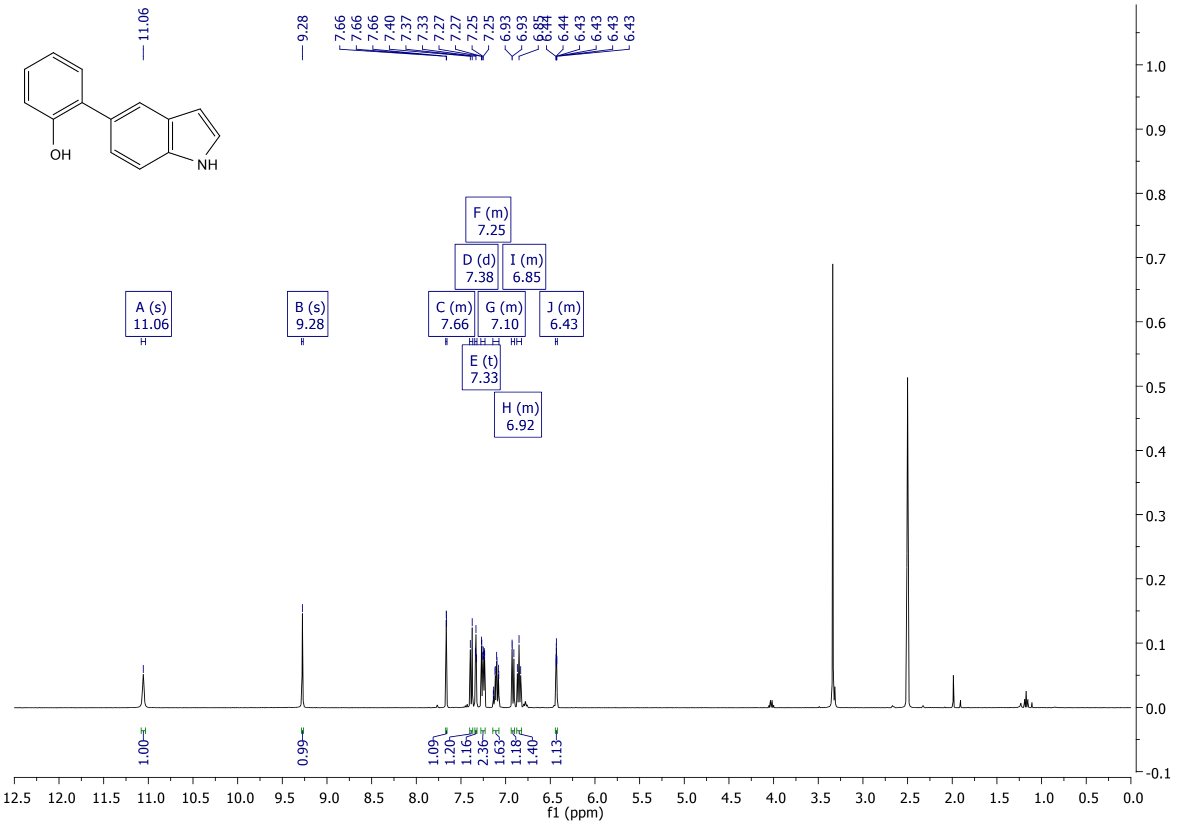

Figure S1.7 ^{1}H NMR spectrum of compound **i-2** in DMSO-d_6.

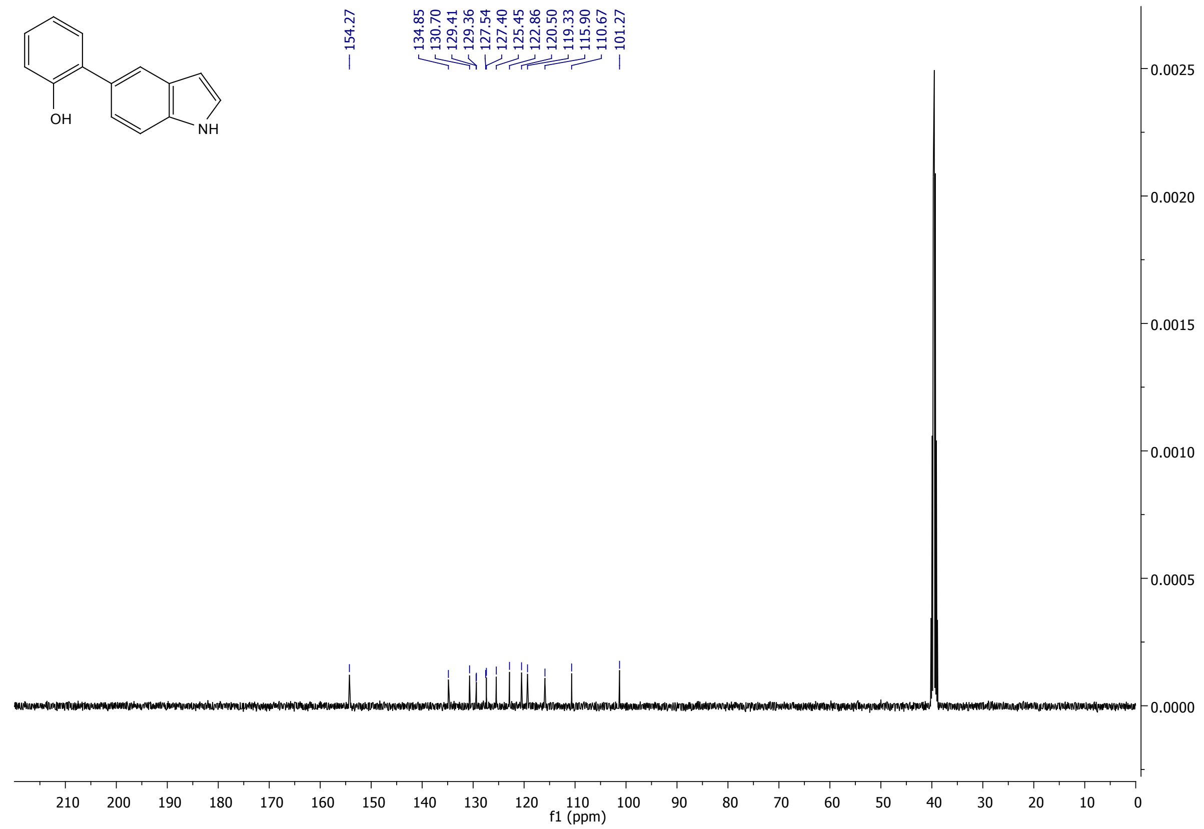

Figure S1.8 ^{13}C NMR spectrum of compound **i-2** in DMSO-d_6.

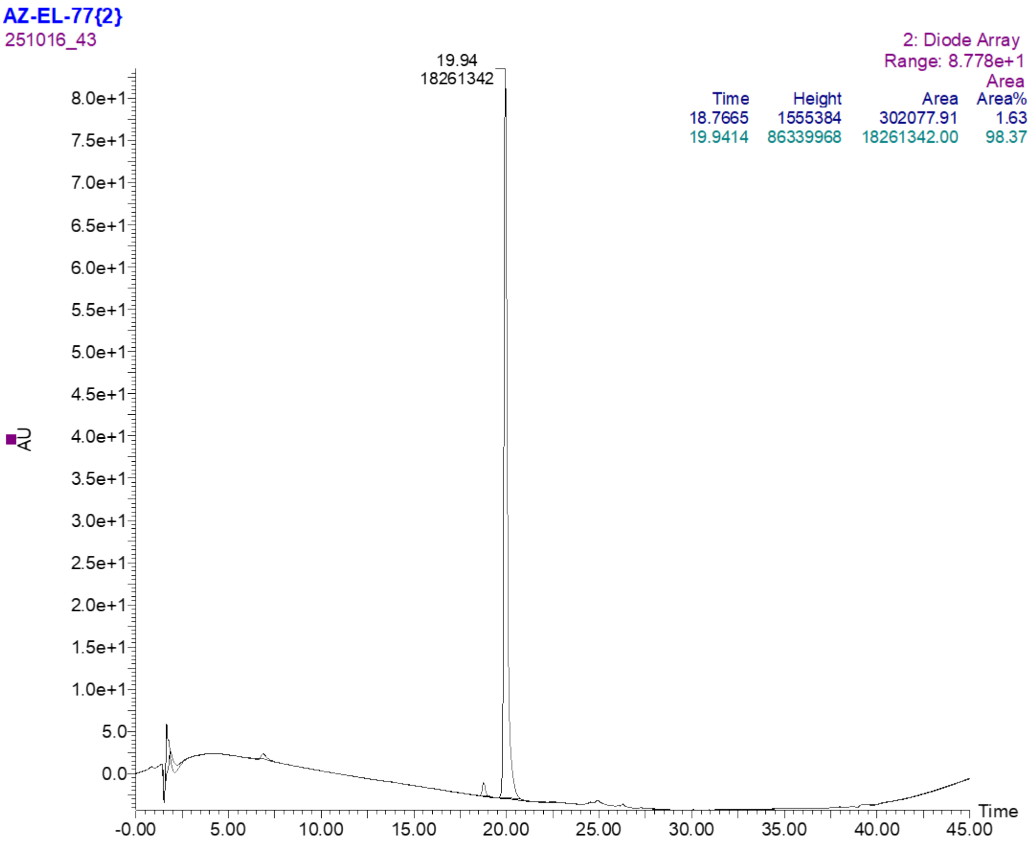

Figure S1.9 LC-MS chromatogram of compound **i-2**.

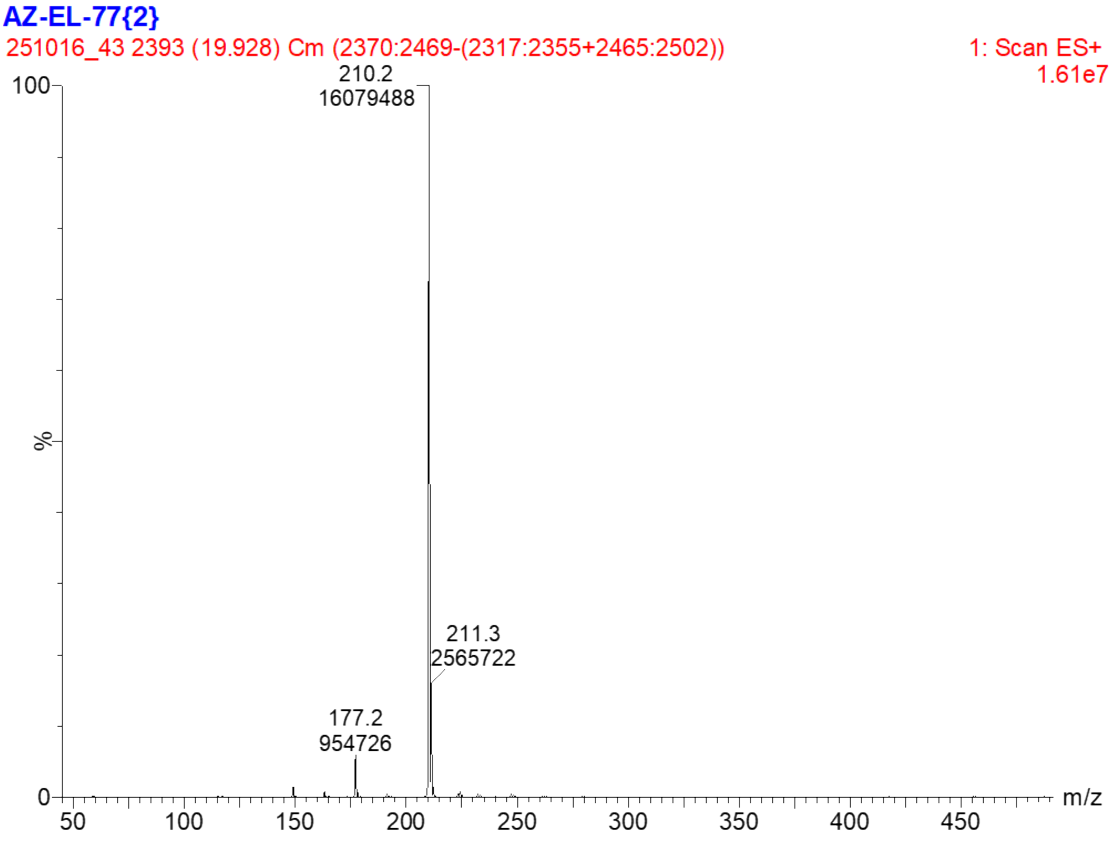

Figure S1.10 MS spectrum of compound **i-2**.

3-(1*H*-Indol-5-yl)phenol **i-3**

Prepared in accordance with general procedure **I** from 5-bromo-1*H*-indole (137 mg, 0.70 mmol) and (3-hydroxyphenyl)boronic acid (145 mg, 1.05 mmol). Yield 95%, white solid; mp = 106.5–107.2 °C, R_f = 0.21 (*n*-hexane/ethyl acetate, 7/3). IR (neat) ν_{max}, cm^{-1}: 3465 (), 1594, 1417, 1190, 1156, 1086, 793, 768, 740, 472, 418. ^{1}H NMR (500 MHz, DMSO-d_6): δ 6.47-6.50 (1H, m, CH), 6.70 (1H, dd, *J* = 8.0, 2.3 Hz, CH), 7.02-7.10 (2H, m, 2×CH), 7.22 (1H, t, *J* = 7.8 Hz, CH), 7.33 (1H, dd, *J* = 8.5, 1.5 Hz, CH), 7.36-7.39 (1H, m, CH), 7.46 (1H, d, *J* = 8.5 Hz, CH), 7.73-7.76 (1H, m, CH), 9.45 (1H, s, OH), 11.14 (1H, br s, NH). ^{13}C NMR (125 MHz, DMSO-d_6): δ 101.6 (CH), 111.8 (CH), 113.2 (CH), 113.6 (CH), 117.6 (CH), 118.0 (CH), 120.4 (CH), 126.1 (CH), 128.2 (C), 129.8 (CH), 131.6 (C), 135.5 (C), 143.4 (C), 157.7 (C). MS (pos. mode): m/z (%): 210.2 (M+H$^+$, 100%).

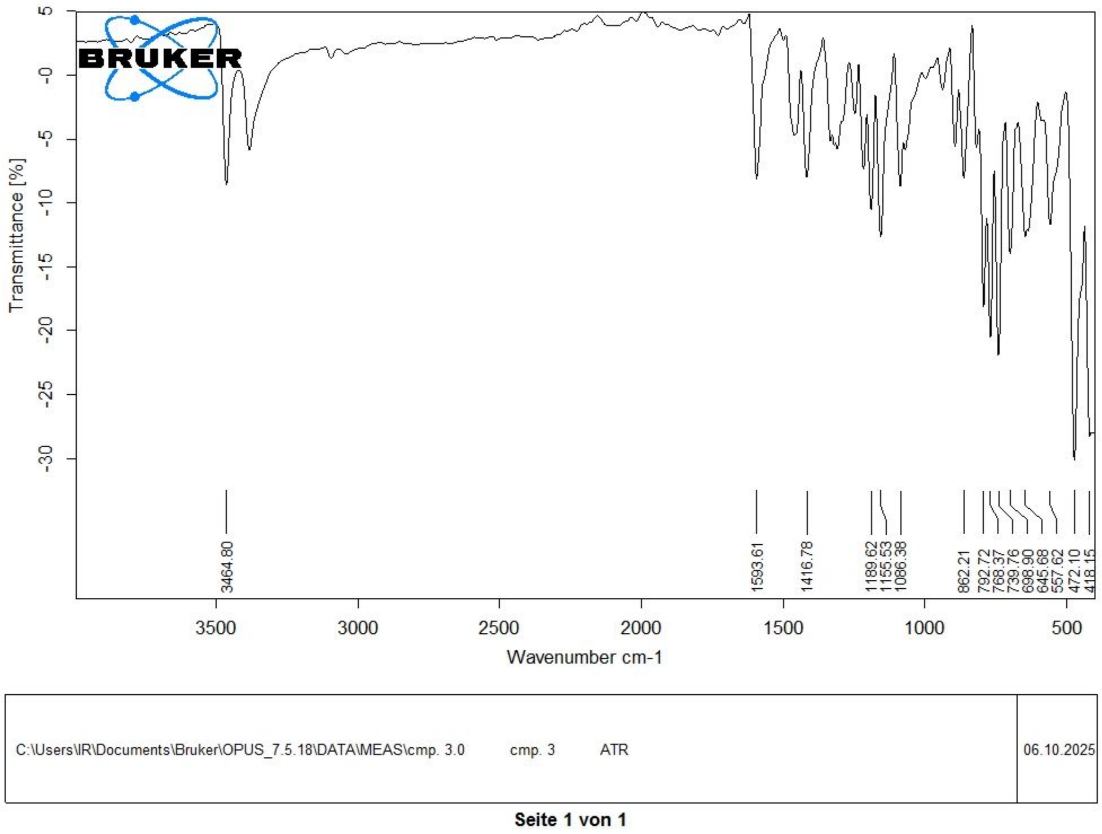

Figure S1.11 FTIR spectrum of compound **i-3**.

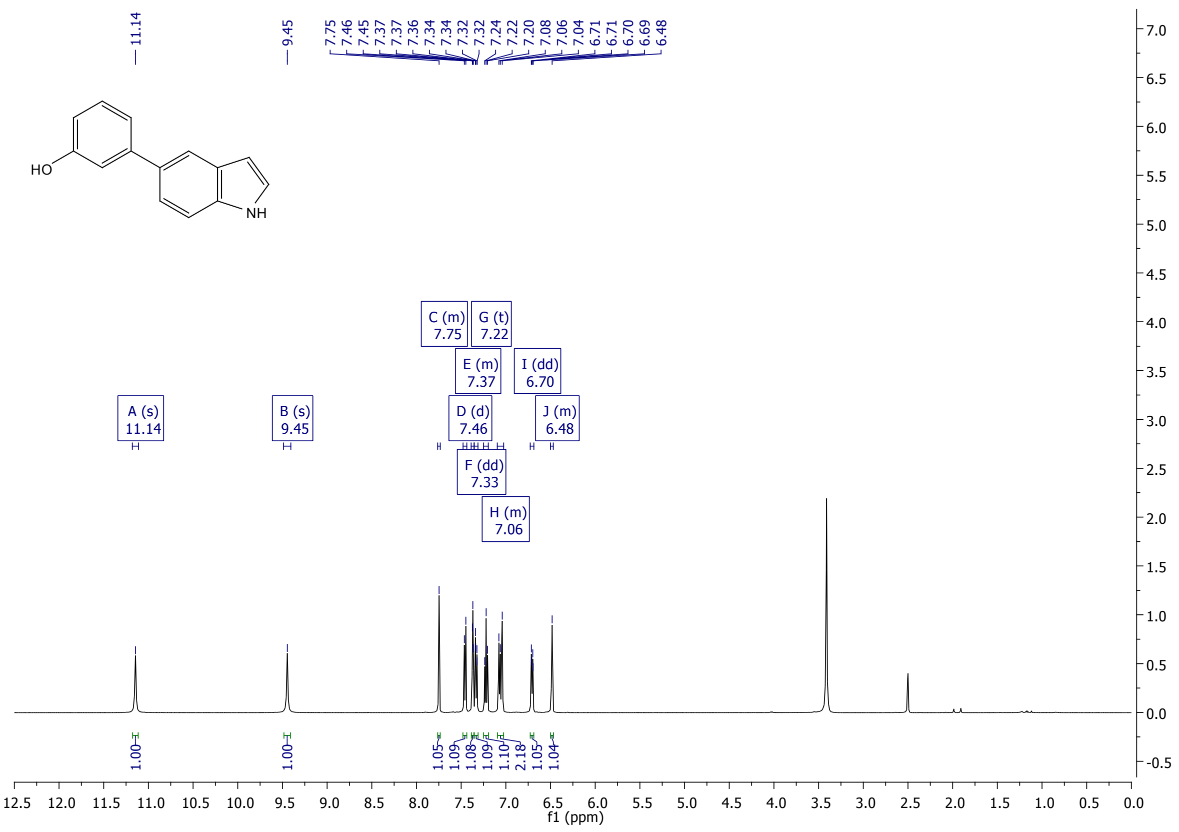

Figure S1.12 ^{1}H NMR spectrum of compound **i-3** in DMSO-d_6.

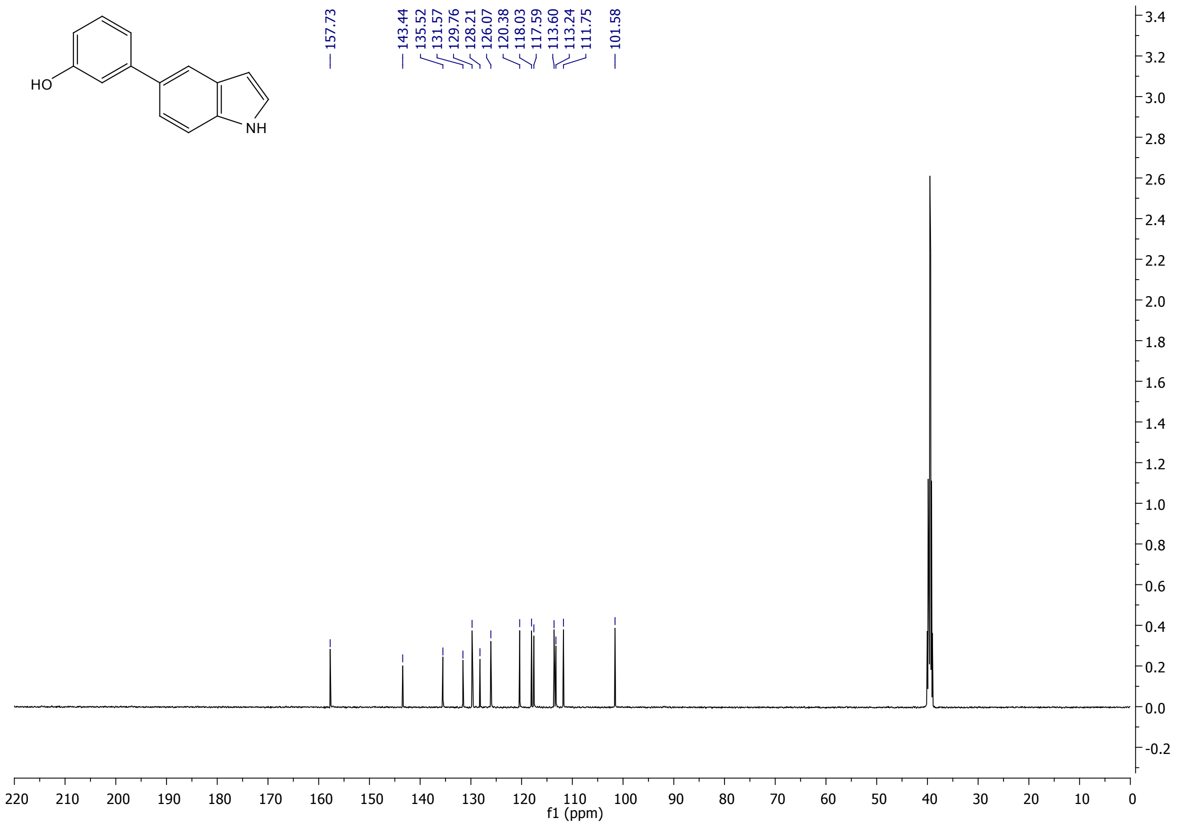

Figure S1.13 ^{13}C NMR spectrum of compound **i-3** in DMSO-d_6.

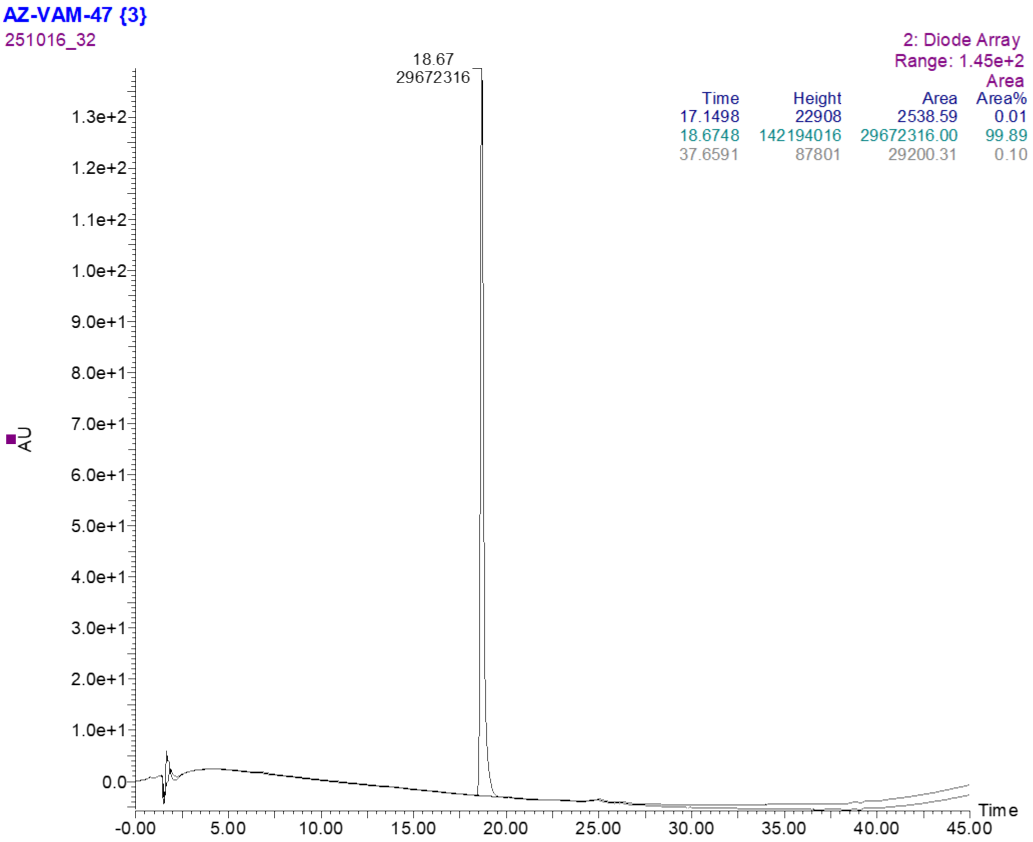

Figure S1.14 LC-MS chromatogram of compound **i-3**.

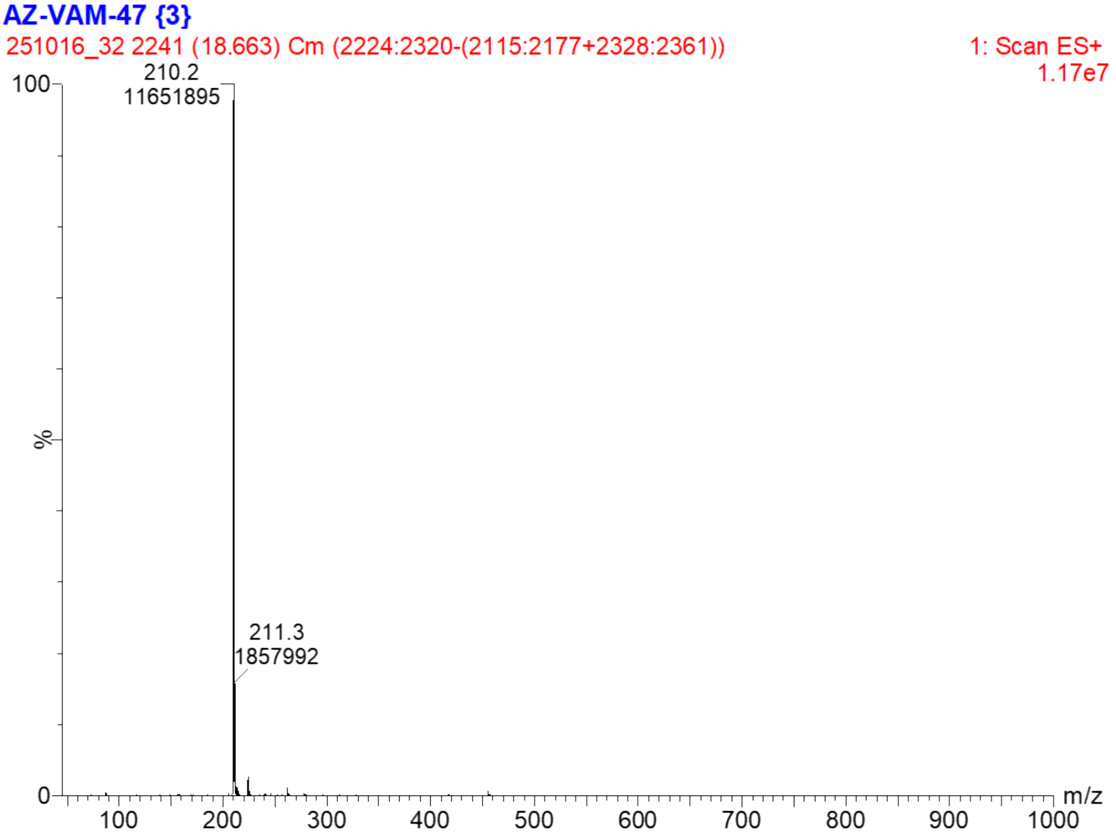

Figure S1.15 MS spectrum of compound **i-3**.

4-(1*H*-Indol-5-yl)phenol **i-4**

Previously reported in [2]. Prepared in accordance with general procedure **I** from 5-bromo-1*H*-indole (137 mg, 0.70 mmol) and (4-hydroxyphenyl)boronic acid (145 mg, 1.05 mmol). Yield 90%, white solid; mp = 115.9–116.6 °C, R_f = 0.23 (*n*-hexane/ethyl acetate, 7/3). IR (neat) ν_{max}, cm^{-1}: 3401, 1463, 1220, 802, 765, 732, 607, 466, 427. ^{1}H NMR (500 MHz, DMSO-d_6): δ 6.43 (1H, s, CH), 6.82 (2H, d, *J* = 8.3 Hz, 2×CH), 7.29 (1H, d, *J* = 8.0 Hz, CH), 7.32-7.35 (1H, m, CH), 7.38-7.48 (3H, m, 3×CH), 7.68 (1H, s, CH), 9.37 (1H, s, OH), 11.06 (1H, s, NH). ^{13}C NMR (125 MHz, DMSO-d_6): δ 101.4 (CH), 111.6 (CH), 115.6 (2×CH), 117.3 (CH), 120.1 (CH), 125.8 (CH), 127.7 (2×CH), 128.3 (C), 131.6 (C), 132.8 (C), 135.0 (C), 156.2 (C). MS (pos. mode): m/z (%): 210.2 (M+H$^+$, 100%).

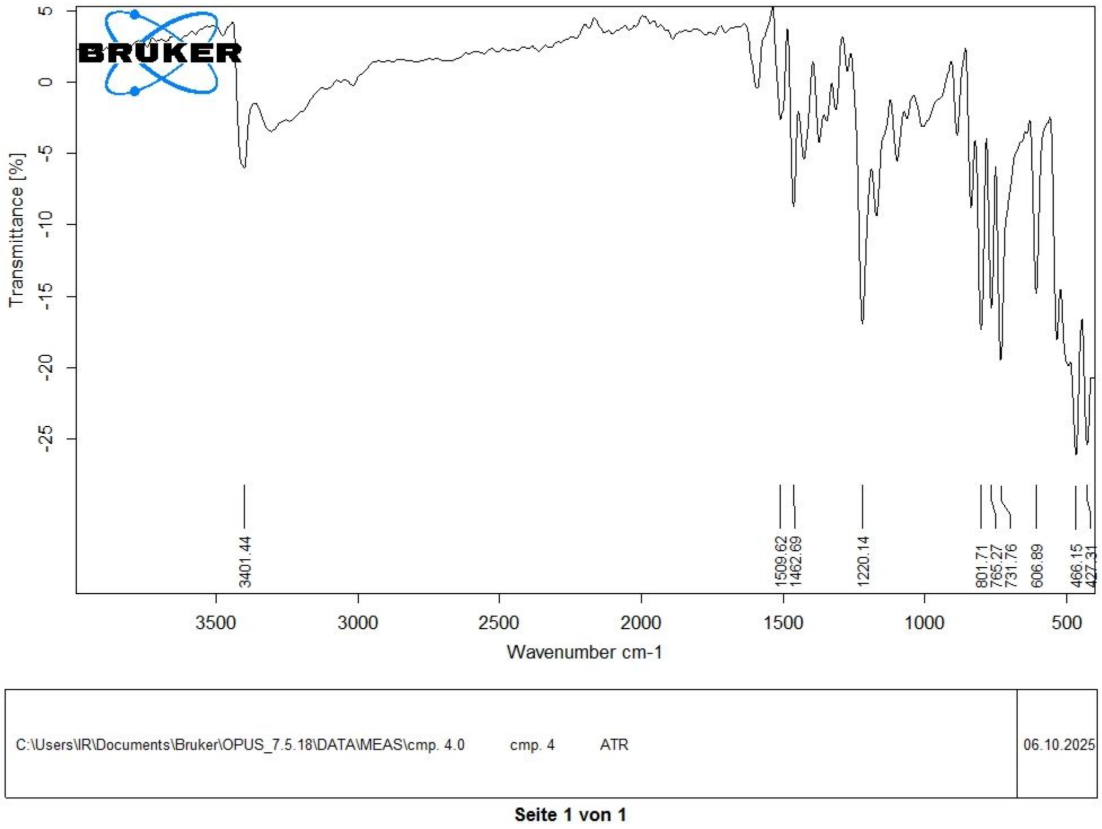

Figure S1.16 FTIR spectrum of compound **i-4**.

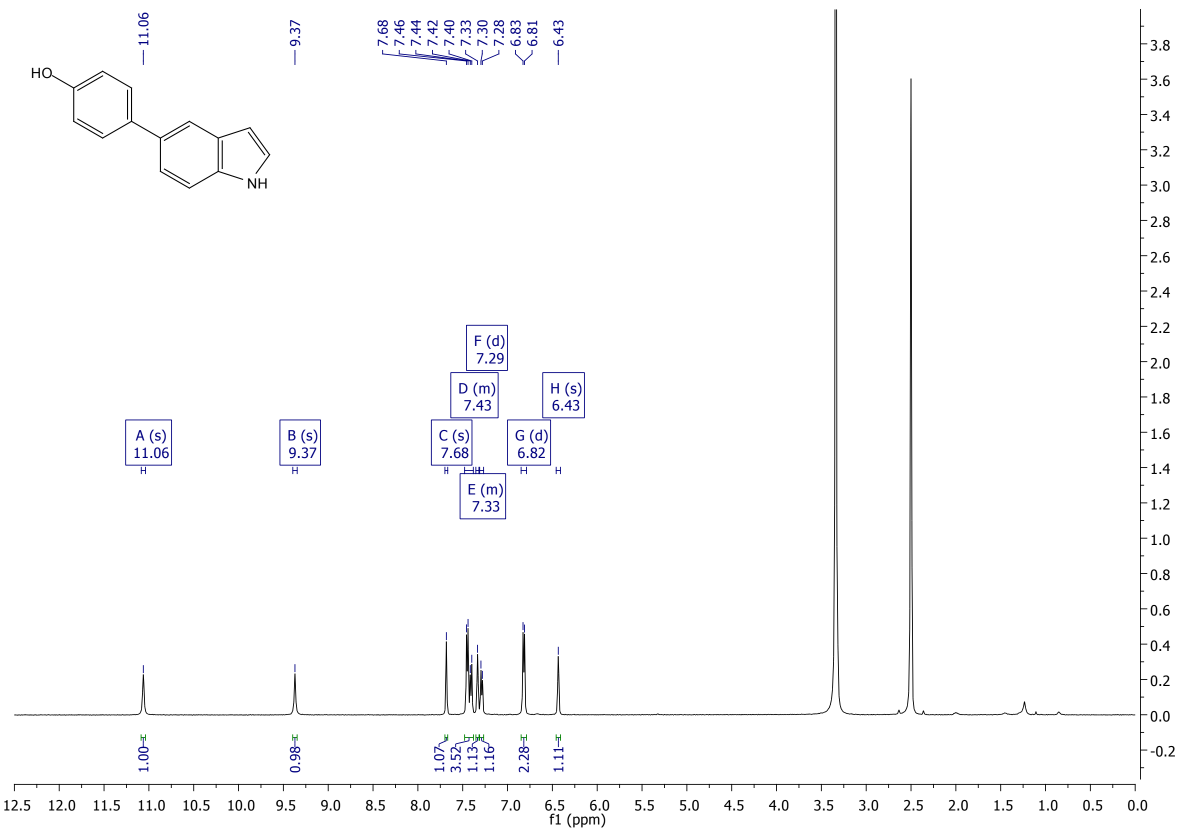

Figure S1.17 ¹H NMR spectrum of compound **i-4** in DMSO-d_6.

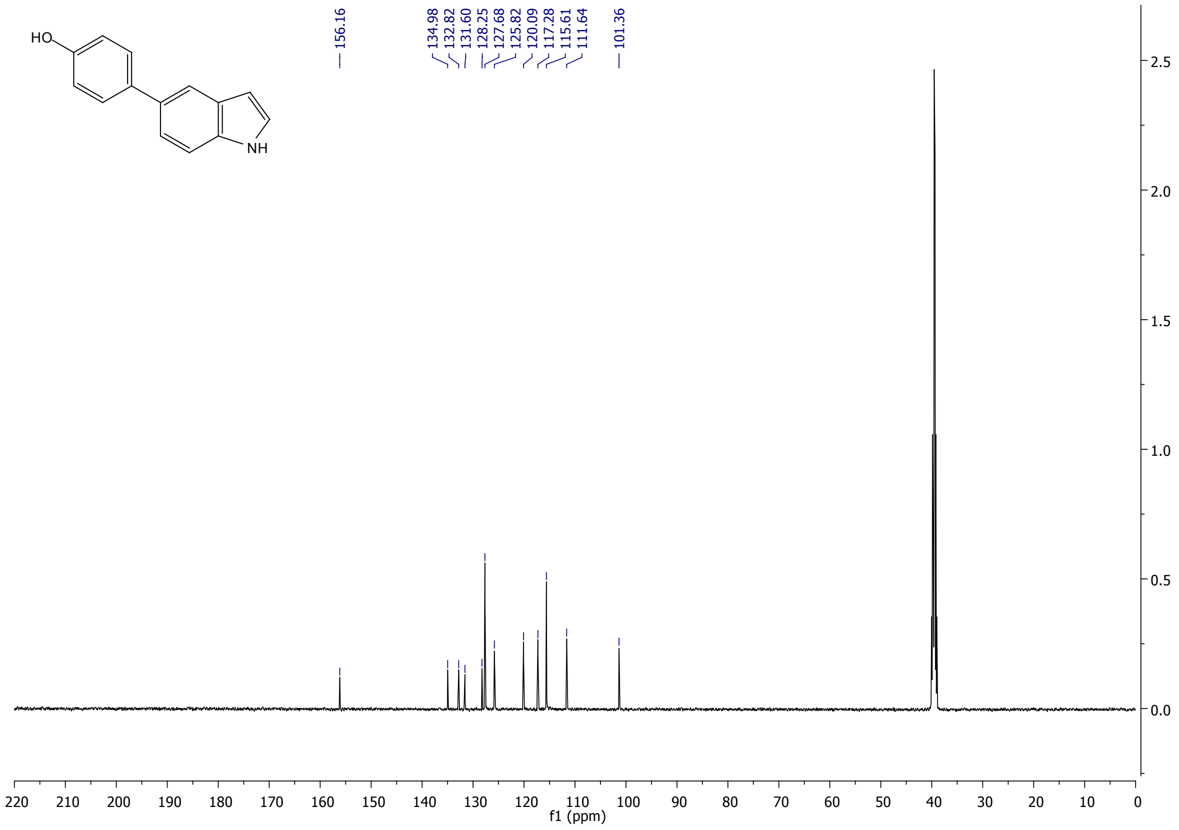

Figure S1.18 ¹³C NMR spectrum of compound **i-4** in DMSO-d_6.

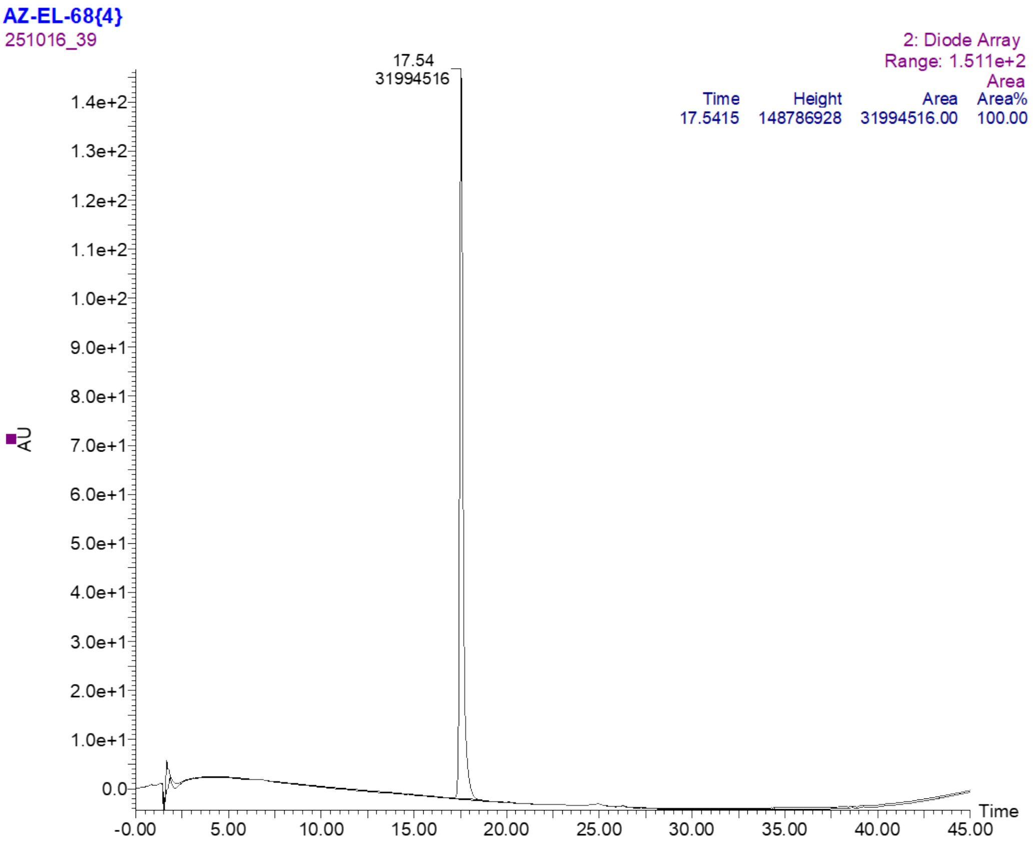

Figure S1.19 LC-MS chromatogram of compound **i-4**.

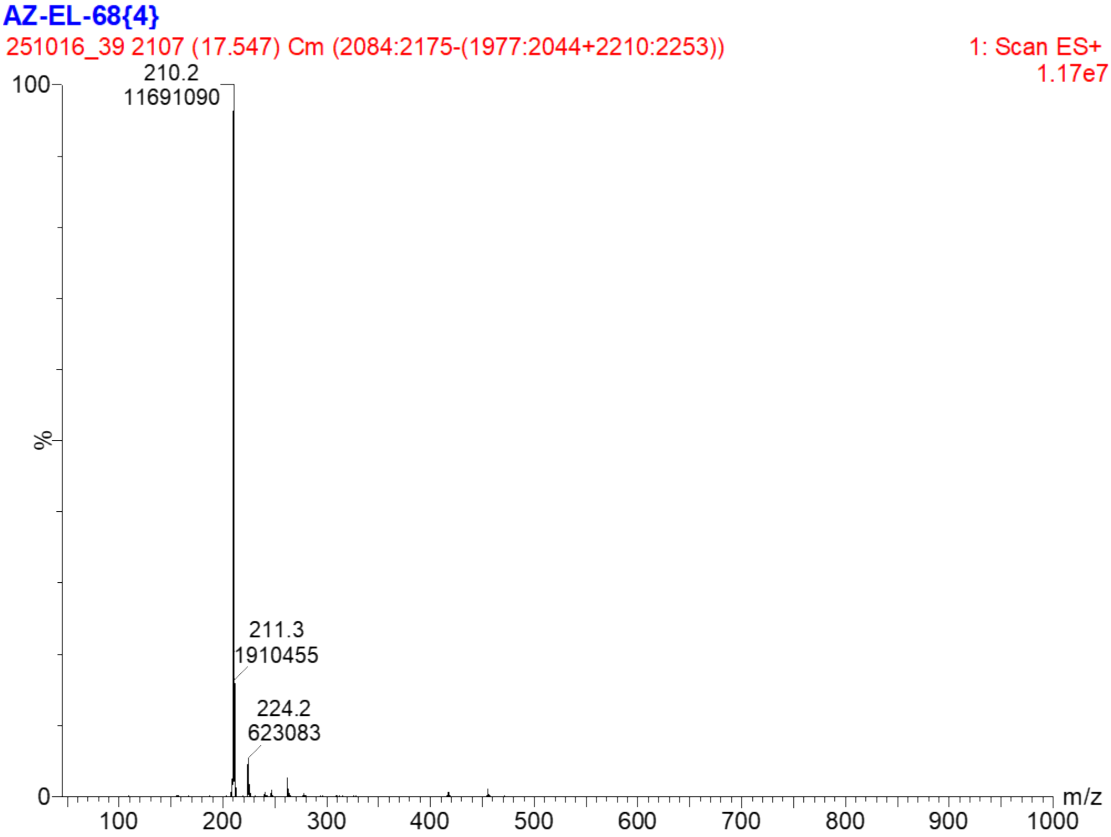

Figure S1.20 MS spectrum of compound **i-4**.

5-(2-Methoxyphenyl)-1*H*-indole **i-5**

Previously reported in [3]. Prepared in accordance with general procedure **I** from 5-bromo-1*H*-indole (150 mg, 0.77 mmol) and (2-methoxyphenyl)boronic acid (174 mg, 1.15 mmol). Yield 71%, pale yellow solid; mp = 92.3–93.1 °C, R_f = 0.26 (*n*-hexane/ethyl acetate, 4/1). IR (neat) ν_{max}, cm^{-1}: 3387 (N-H), 1458, 1227, 1018, 731, 618, 532. ^{1}H NMR (400 MHz, DMSO-d_6): δ 3.74 (3H, s, CH_3), 6.43-6.45 (1H, m, CH), 7.01 (1H, td, *J* = 7.4, 1.1 Hz, CH), 7.07-7.10 (1H, m, CH), 7.19 (1H, dd, *J* = 8.4, 1.6 Hz, CH), 7.26-7.31 (2H, m, 2×CH), 7.35 (1H, t, *J* = 2.8 Hz, CH), 7.39 (1H, d, *J* = 8.4 Hz, CH), 7.59-7.61 (1H, m, CH), 11.09 (1H, br s, NH). ^{13}C NMR (100 MHz, DMSO-d_6): δ 55.4 (CH_3), 101.3 (CH), 110.7 (CH), 111.7 (CH), 120.65 (CH), 120.68 (CH), 122.9 (CH), 125.6 (CH), 127.5 (C),127.8 (CH), 129.0 (C), 130.8 (CH), 131.5 (C), 135.0 (C), 156.3 (C). MS (pos. mode): m/z (%): 224.1 (M+H$^+$, 100%).

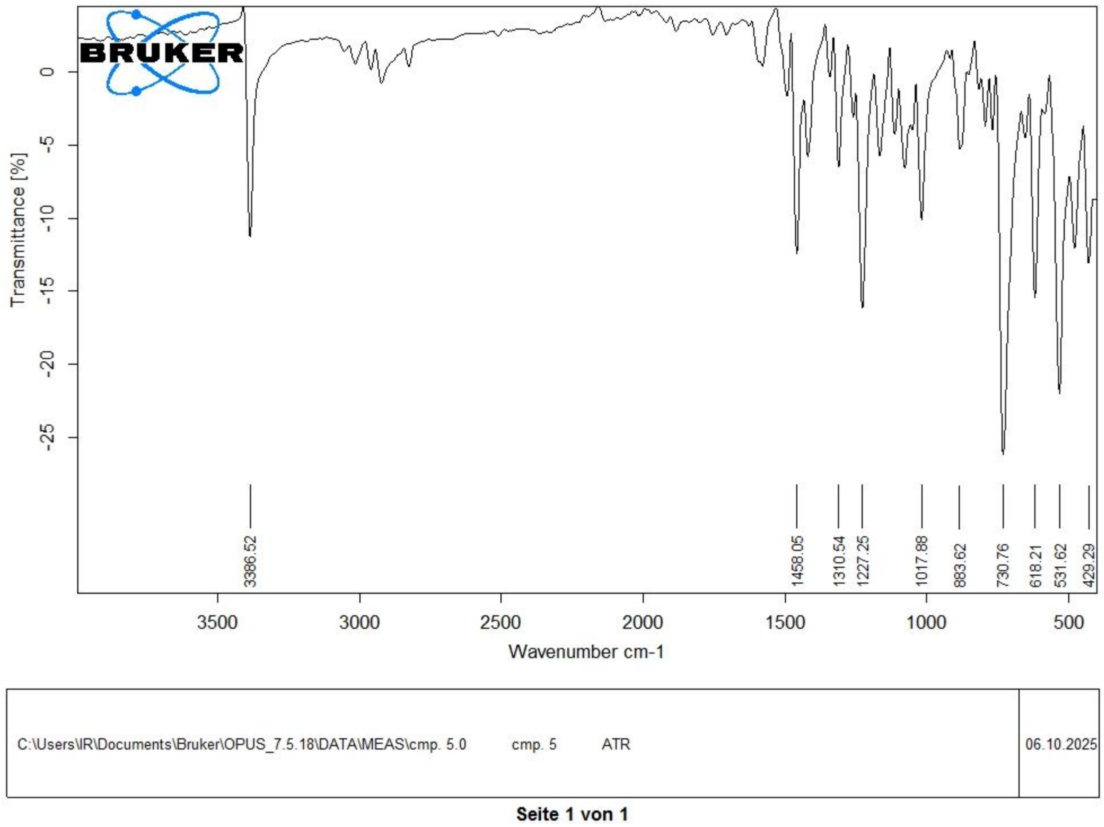

Figure S1.21 FTIR spectrum of compound **i-5**.

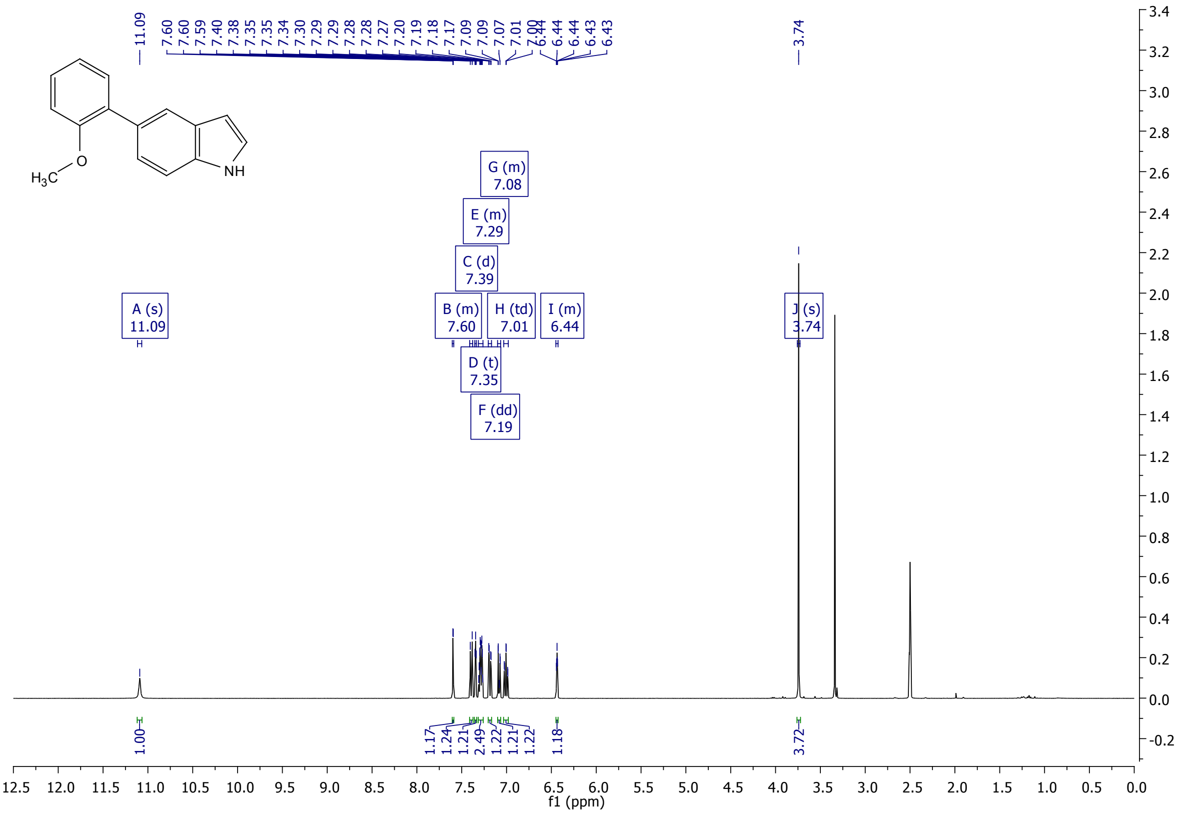

Figure S1.22 ^{1}H NMR spectrum of compound **i-5** in DMSO-d_6.

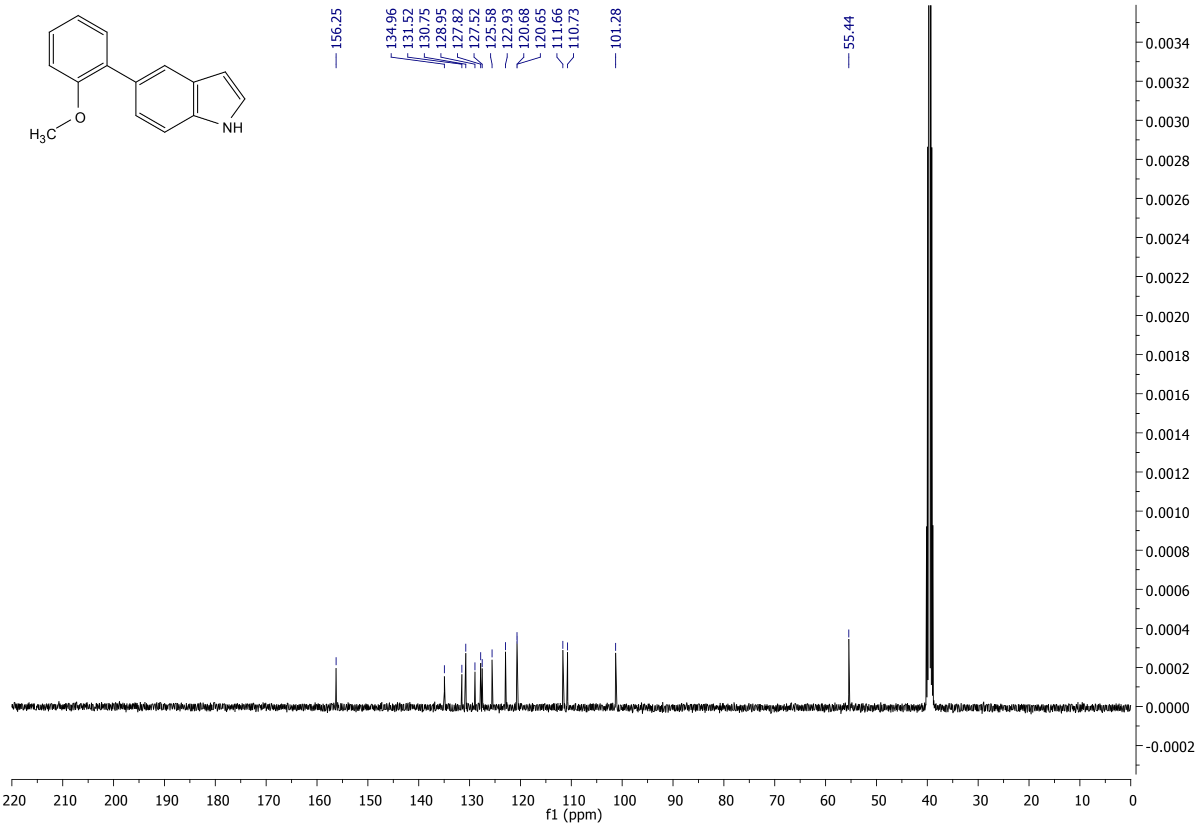

Figure S1.23 ^{13}C NMR spectrum of compound **i-5** in DMSO-d_6.

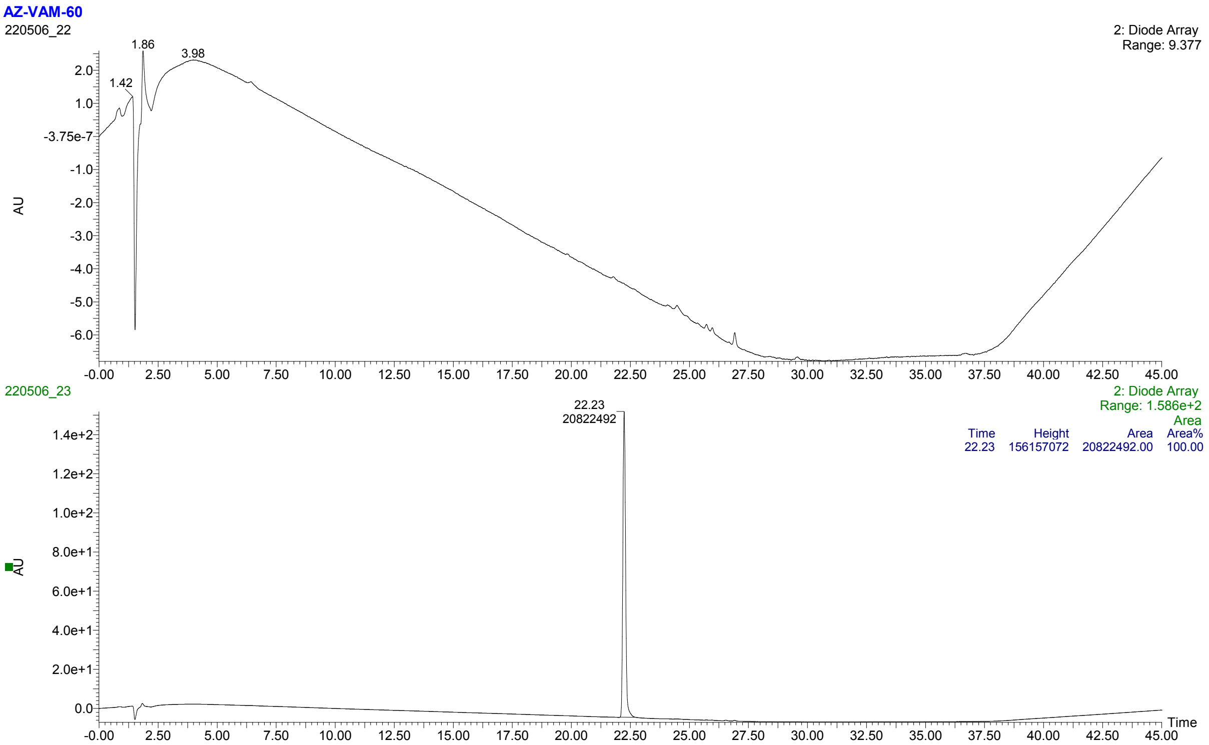

Figure S1.24 LC-MS chromatogram of compound **i-5**.

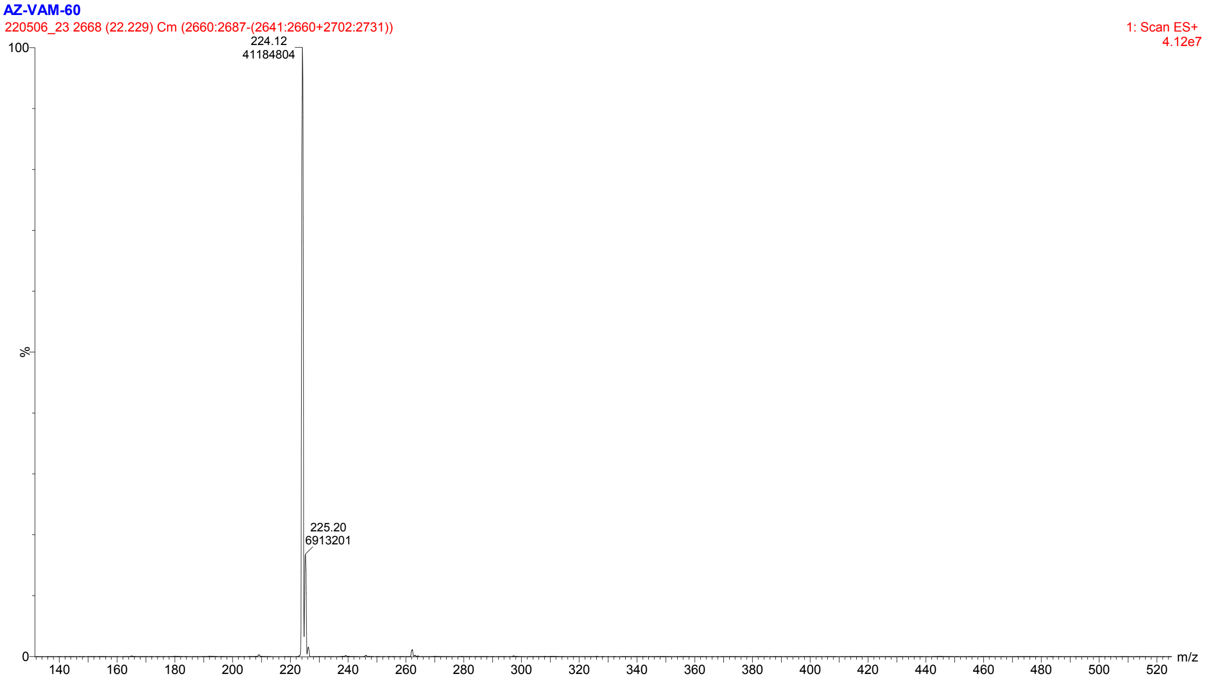

Figure S1.25 MS spectrum of compound **i-5**.

5-(3-Methoxyphenyl)-1*H*-indole **i-6**

Prepared in accordance with general procedure **I** from 5-bromo-1*H*-indole (150 mg, 0.77 mmol) and (3-methoxyphenyl)boronic acid (174 mg, 1.15 mmol). Yield 83%, pale yellow solid; mp = 81.8–82.6 °C, R_f = 0.24 (*n*-hexane/ethyl acetate, 4/1). IR (neat) ν_{max}, cm^{-1}: 3405 (N-H), 1459, 1216, 773, 733, 493, 425. ^{1}H NMR (500 MHz, DMSO-d_6): δ 3.83 (3H, s, CH_3), 6.48-6.51 (1H, m, CH), 6.86 (1H, dd, J = 8.1, 2.0 Hz, CH), 7.19-7.21 (1H, m, CH), 7.22-7.25 (1H, m, CH), 7.34 (1H, d, J = 7.9 Hz, CH), 7.38-7.42 (2H, m, 2×CH), 7.48 (1H, d, J = 8.4 Hz, CH), 7.82-7.85 (1H, m, CH), 11.17 (1H, br s, NH). ^{13}C NMR (125 MHz, DMSO-d_6): δ 55.0 (CH_3), 101.6 (CH), 111.75 (CH), 111.79 (CH), 112.2 (CH), 118.3 (CH), 119.1 (CH), 120.5 (CH), 126.1 (CH), 128.2 (C), 129.8 (CH), 131.3 (C), 135.6 (C), 143.5 (C), 159.7 (C). MS (pos. mode): m/z (%): 224.1 ($M+H^+$, 100%).

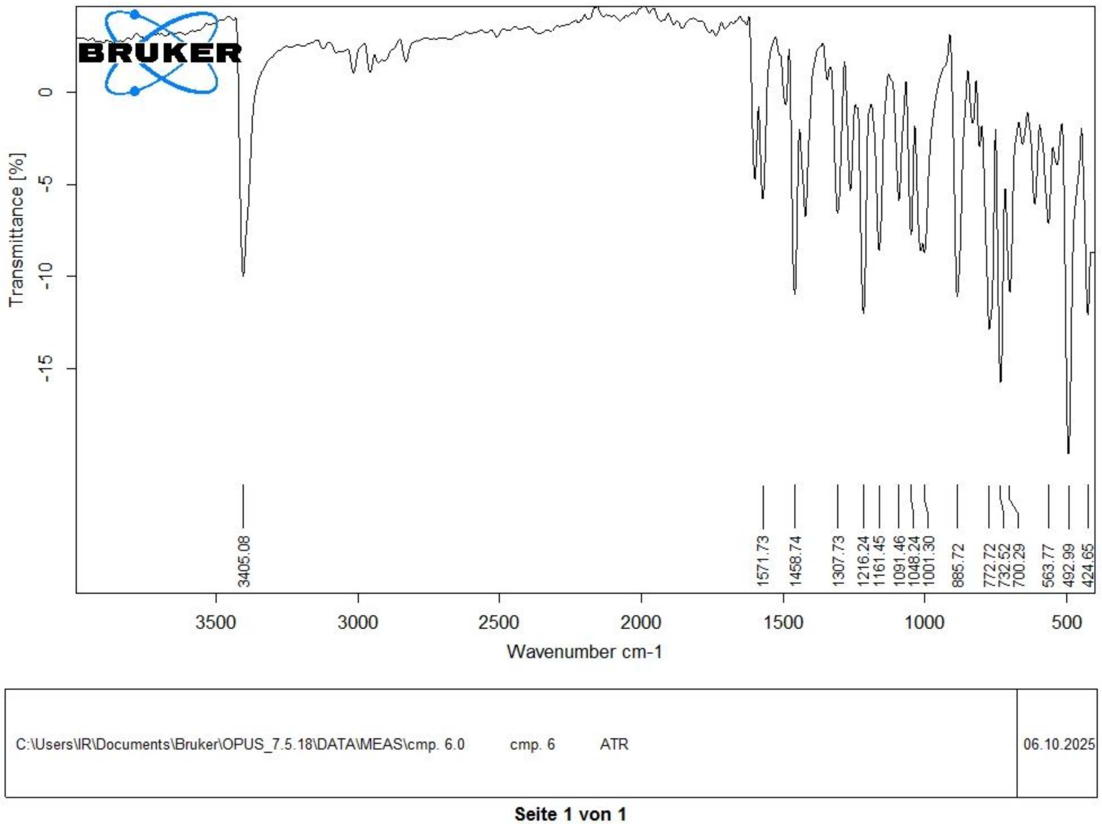

Figure S1.26 FTIR spectrum of compound **i-6**.

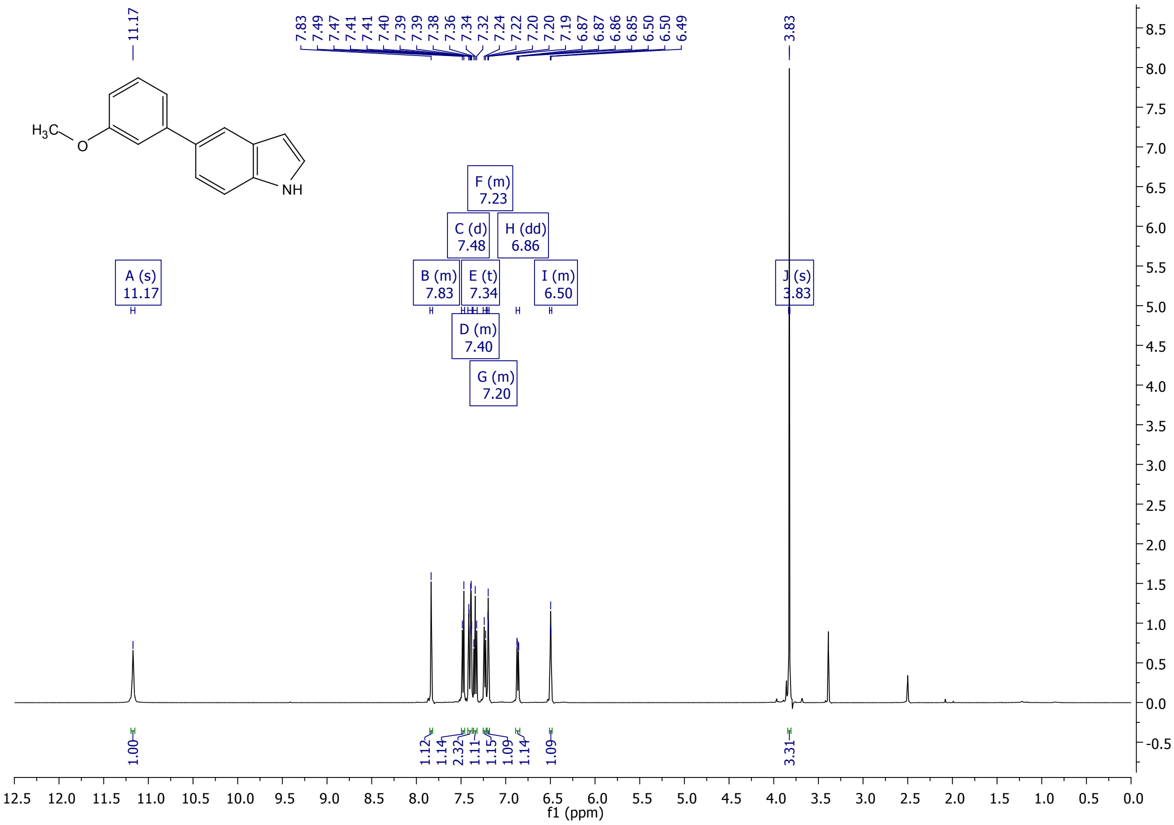

Figure S1.27 ^{1}H NMR spectrum of compound **i-6** in DMSO-d_6.

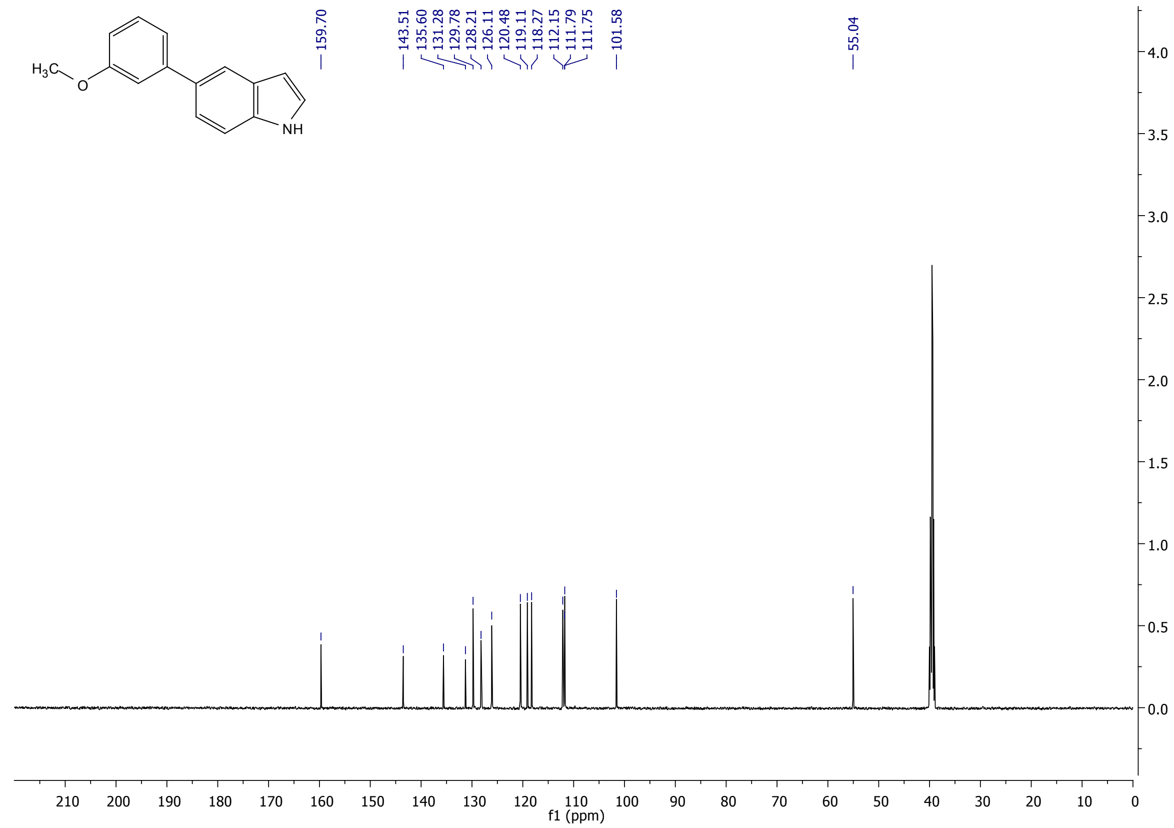

Figure S1.28 ^{13}C NMR spectrum of compound **i-6** in DMSO-d_6.

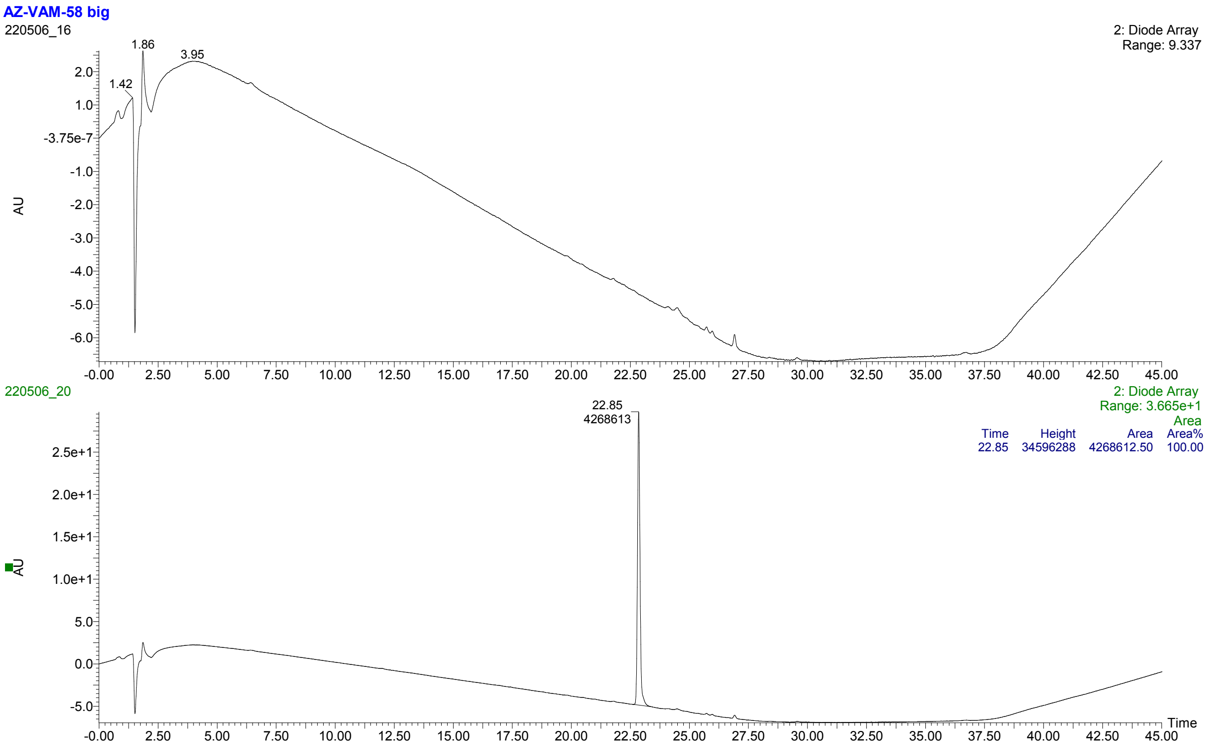

Figure S1.29 LC-MS chromatogram of compound **i-6**.

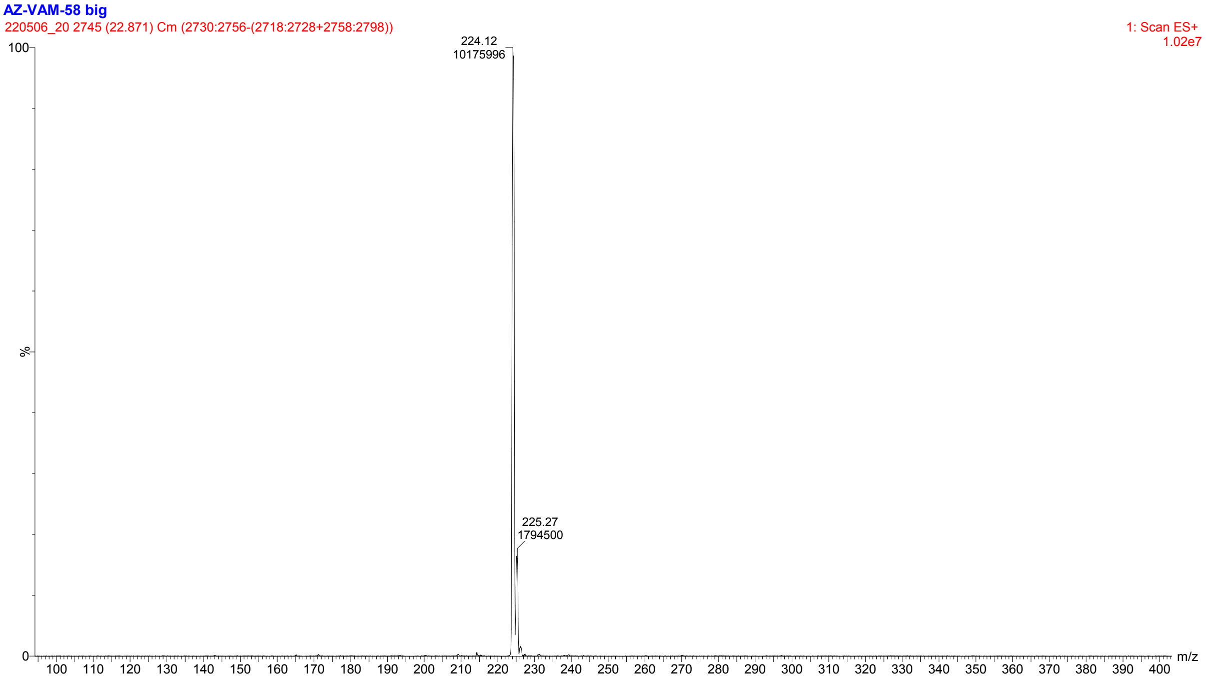

Figure S1.30 MS spectrum of compound **i-6**.

5-(4-Methoxyphenyl)-1*H*-indole **i-7**

Previously reported in [4]. Prepared in accordance with general procedure **I** from 5-bromo-1*H*-indole (50 mg, 0.26 mmol) and (4-methoxyphenyl)boronic acid (58 mg, 0.38 mmol). Yield 74%, pale pink solid; mp = 127.8–129.6 °C, R_f = 0.24 (*n*-hexane/ethyl acetate, 4/1). IR (neat) ν_{max}, cm^{-1}: 3413 (N-H), 1457, 1234, 1176, 1026, 780, 765, 732, 603, 473. ^1H NMR (400 MHz, DMSO-d_6): δ 3.78 (3H, s, CH_3), 6.44-6.47 (1H, m, CH), 6.97-7.02 (2H, m, 2×CH), 7.31-7.37 (2H, m, 2×CH), 7.43 (1H, d, J = 8.4 Hz, CH), 7.55-7.60 (2H, m, 2×CH), 7.72-7.75 (1H, m, CH), 11.10 (1H, br s, NH). ^{13}C NMR (100 MHz, DMSO-d_6): δ 55.1 (CH_3), 101.3 (CH), 111.7 (CH), 114.2 (2×CH), 117.5 (CH), 120.2 (CH), 125.9 (CH), 127.7 (2×CH), 128.2 (C), 131.1 (C), 134.4 (C), 135.1 (C), 158.0 (C). MS (pos. mode): m/z (%): 224.2 ($M+H^+$, 100%).

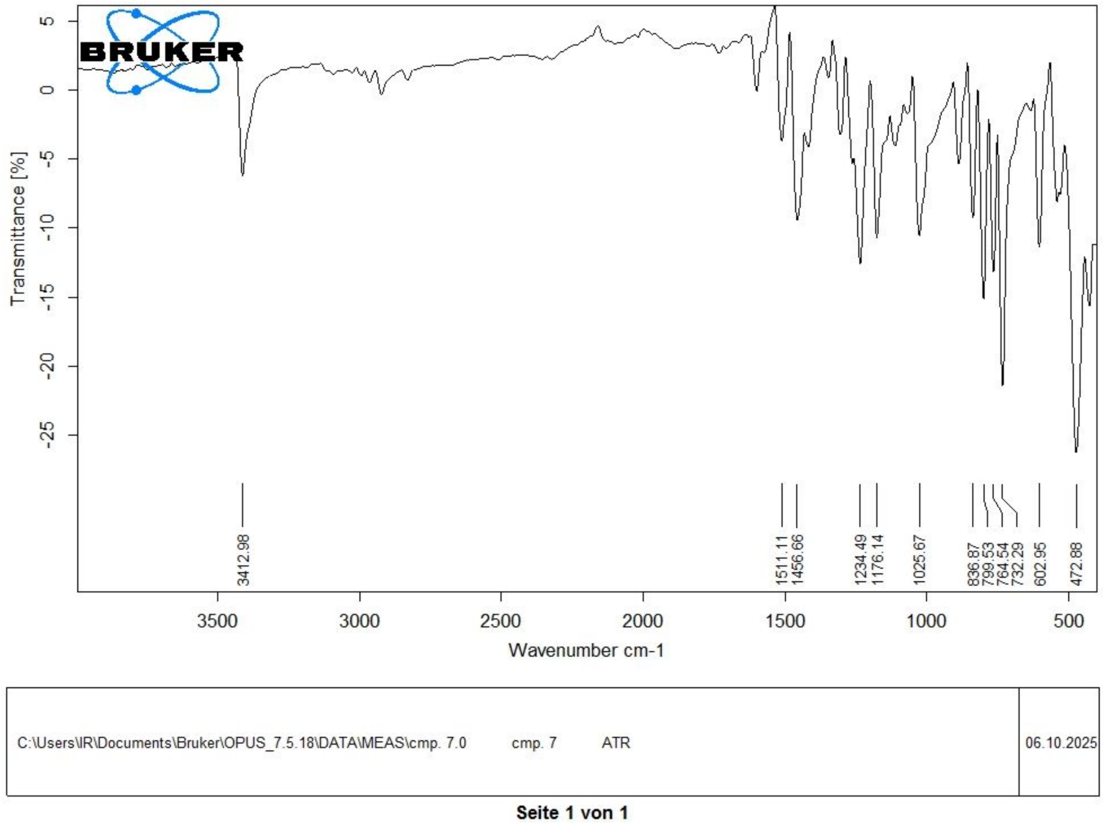

Figure S1.31 FTIR spectrum of compound **i-7**.

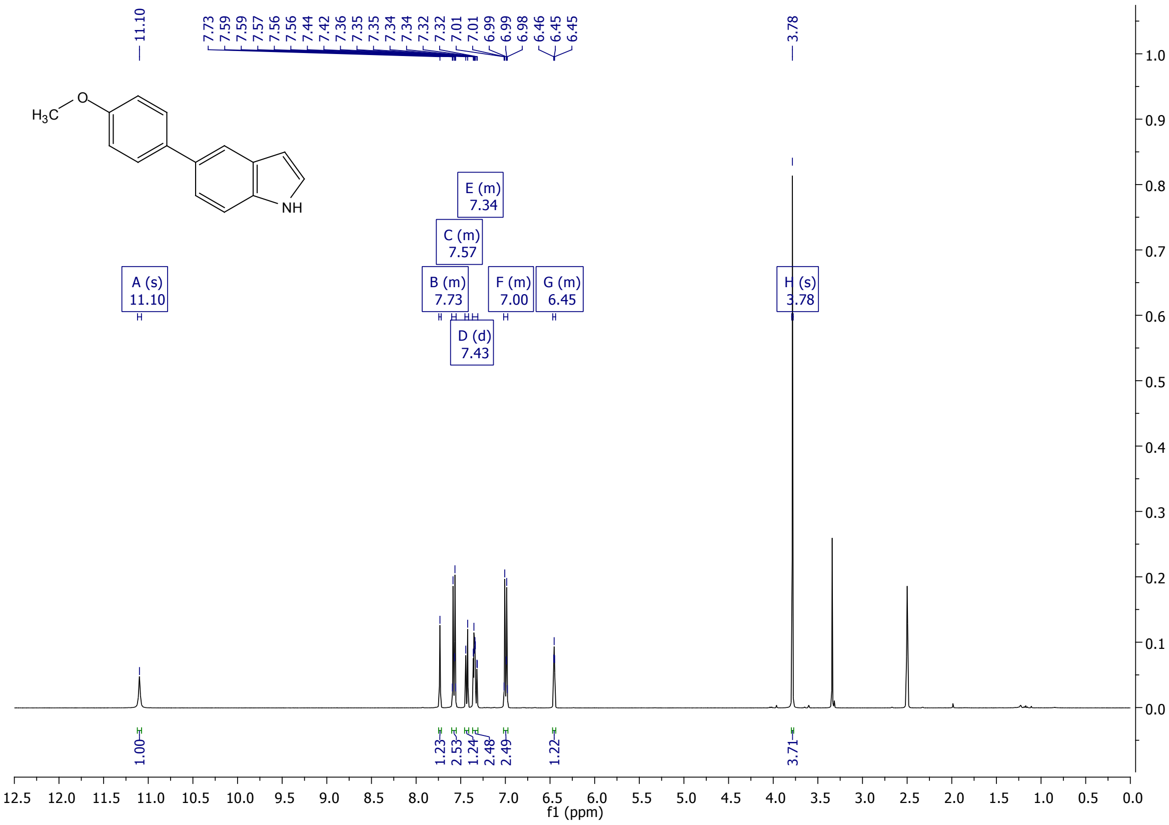

Figure S1.32 ^{1}H NMR spectrum of compound **i-7** in DMSO-d_6.

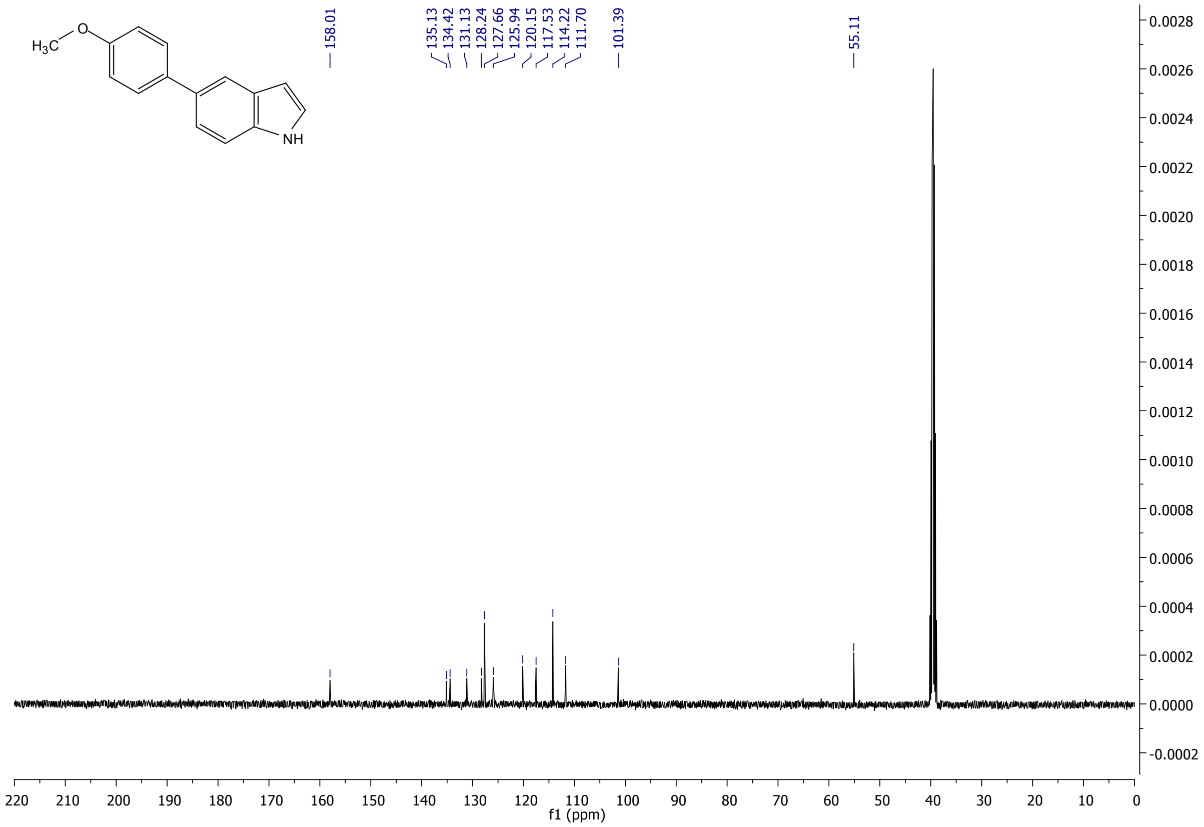

Figure S1.33 ^{13}C NMR spectrum of compound **i-7** in DMSO-d_6.

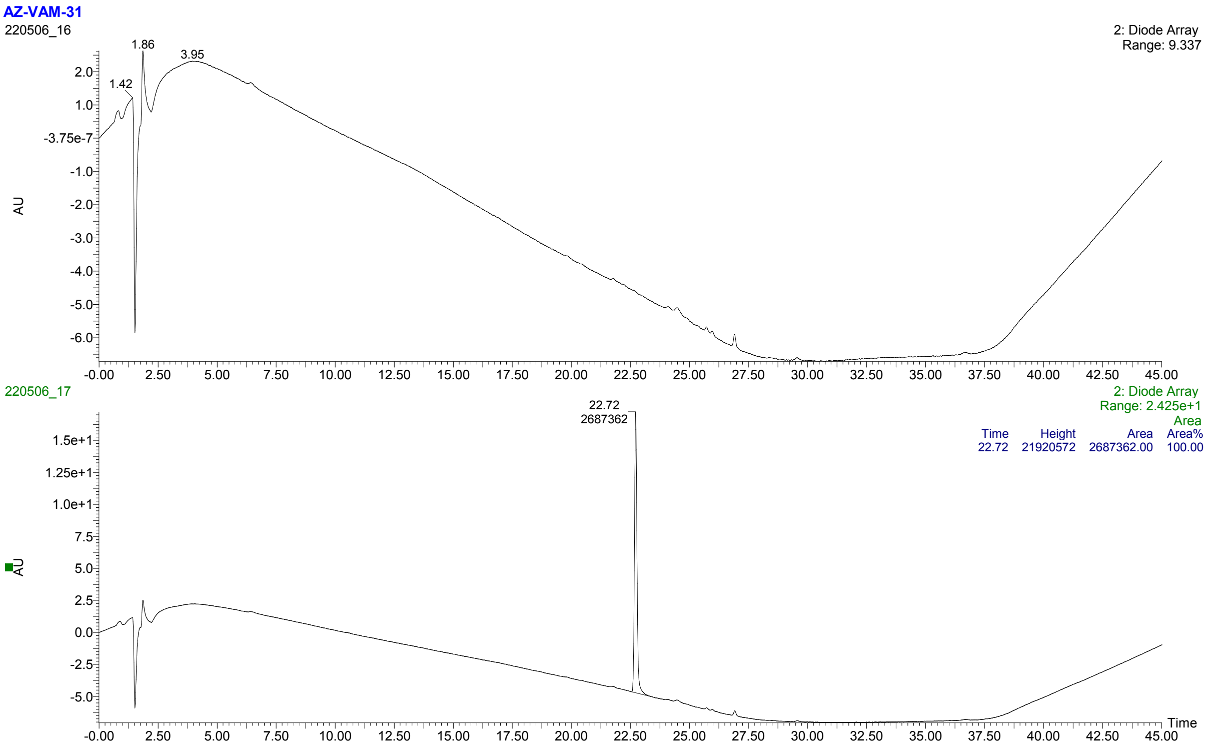

Figure S1.34 LC-MS chromatogram of compound **i-7**.

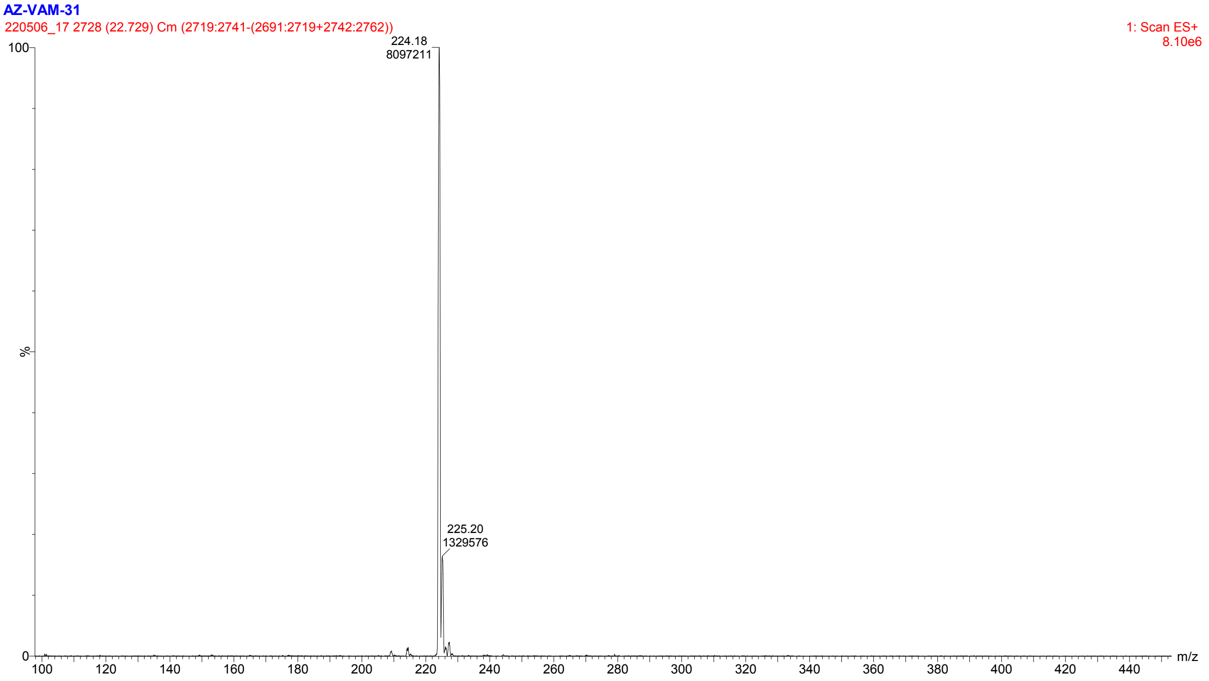

Figure S1.35 MS spectrum of compound **i-7**.

5-(2-Ethoxyphenyl)-1*H*-indole **i-8**

Prepared in accordance with general procedure **II** from 2-(1*H*-indol-5-yl)phenol **i-2** (30 mg, 0.14 mmol) and iodoethane (17 µL, 0.22 mmol). Yield 71%, brown viscous oil, R_f = 0.30 (*n*-hexane/ethyl acetate, 4/1). IR (neat) ν_{max}, cm^{-1}: 3407 (N-H), 1464, 1225, 1120, 1040, 744, 484. ^1H NMR (500 MHz, DMSO-d_6): δ 1.24 (3H, t, *J* = 6.9 Hz, CH_3), 4.02 (2H, q, *J* = 6.9 Hz, CH_2), 6.42-6.45 (1H, m, CH), 7.00 (1H, t, *J* = 7.1, CH), 7.07 (1H, d, *J* = 7.9 Hz, CH), 7.23-7.31 (3H, m, 3×CH), 7.33-7.35 (1H, m, CH), 7.39 (1H, d, *J* = 8.4 Hz, CH), 7.63 (1H, s, CH), 11.08 (1H, br s, NH). ^{13}C NMR (125 MHz, DMSO-d_6): δ 14.7 (CH_3), 63.5 (CH_2), 101.4 (CH), 110.7 (CH), 112.9 (CH), 120.7 (CH), 120.8 (CH), 123.0 (CH), 125.5 (CH), 127.6 (C), 127.8 (CH), 129.1 (C), 130.9 (CH), 131.7 (C), 135.0 (C), 155.5 (C). MS (pos. mode): m/z (%): 238.1 ($M+H^+$, 100%).

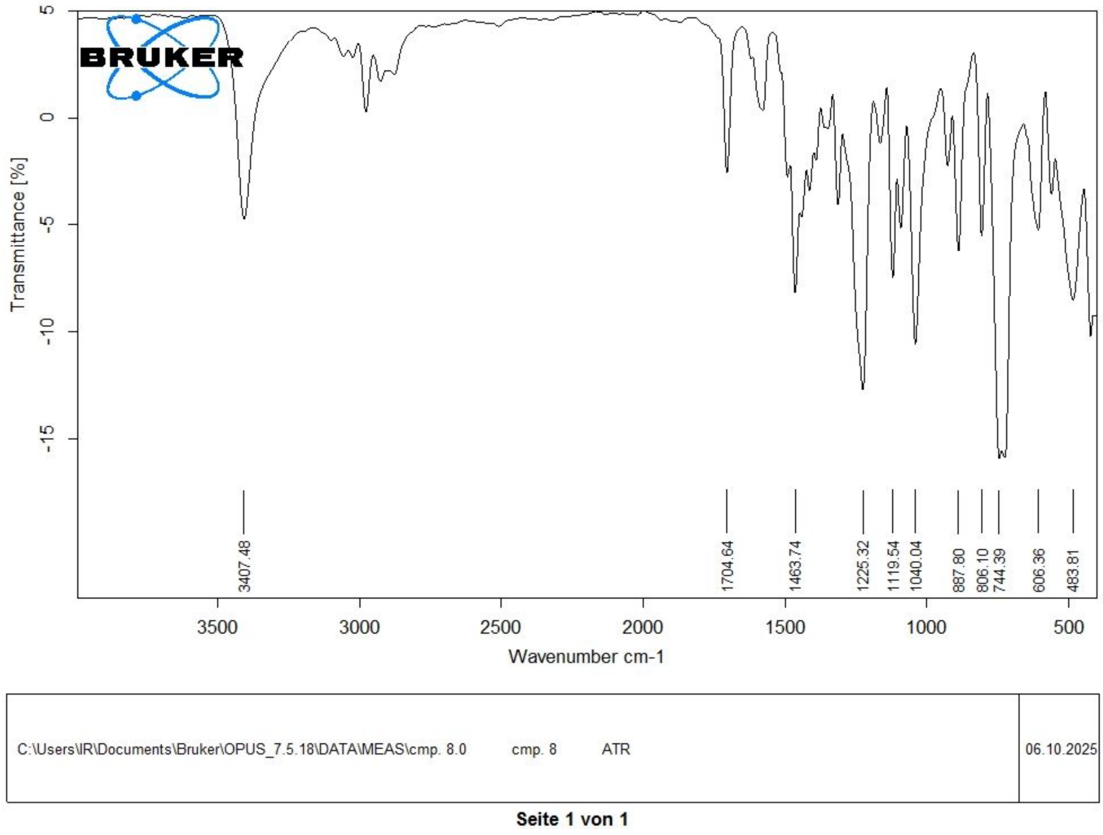

Figure S1.36 FTIR spectrum of compound **i-8**.

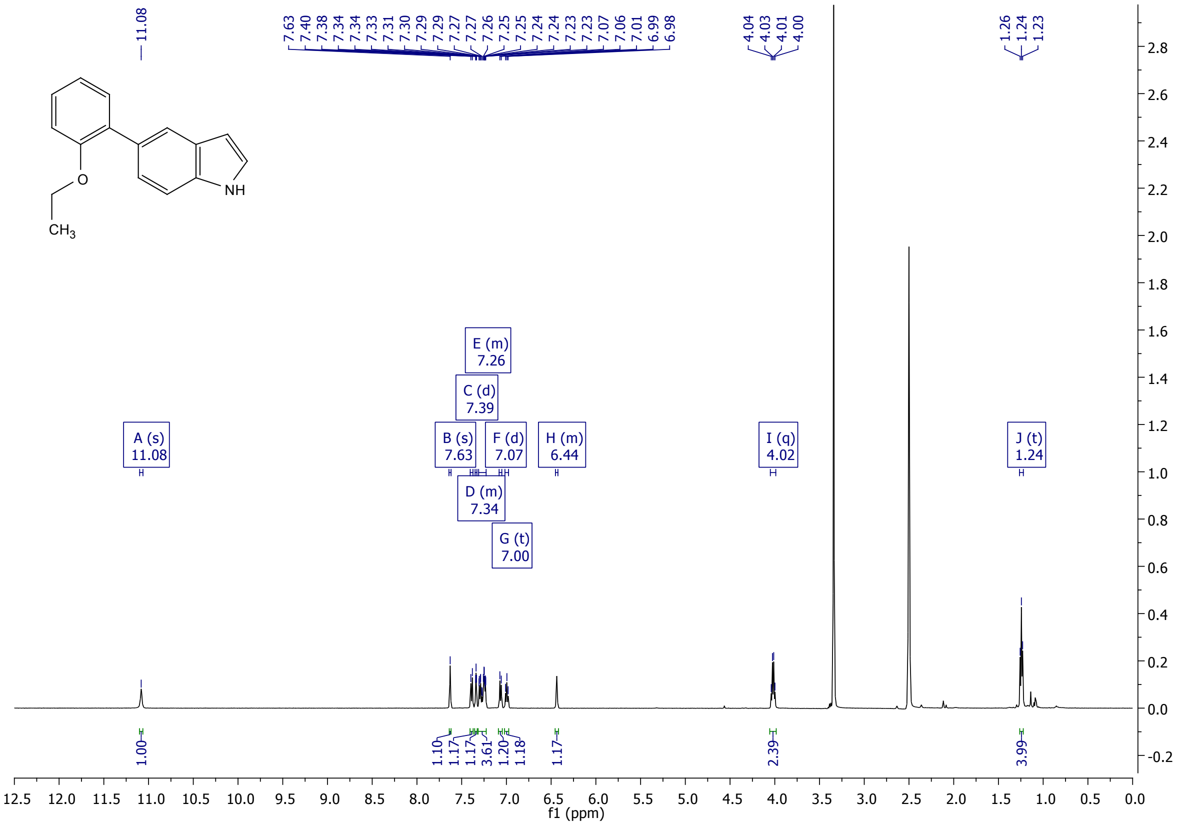

Figure S1.37 ^{1}H NMR spectrum of compound **i-8** in DMSO-d_6.

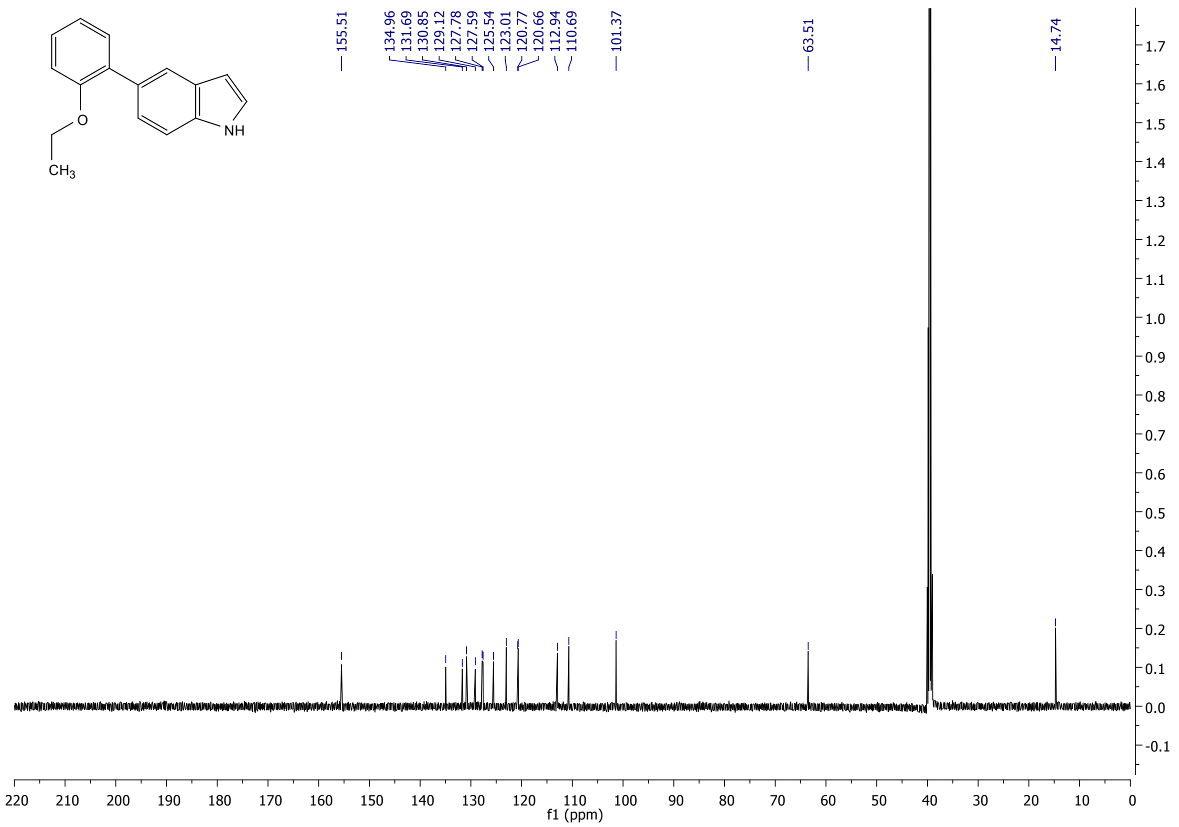

Figure S1.38 ^{13}C NMR spectrum of compound **i-8** in DMSO-d_6.

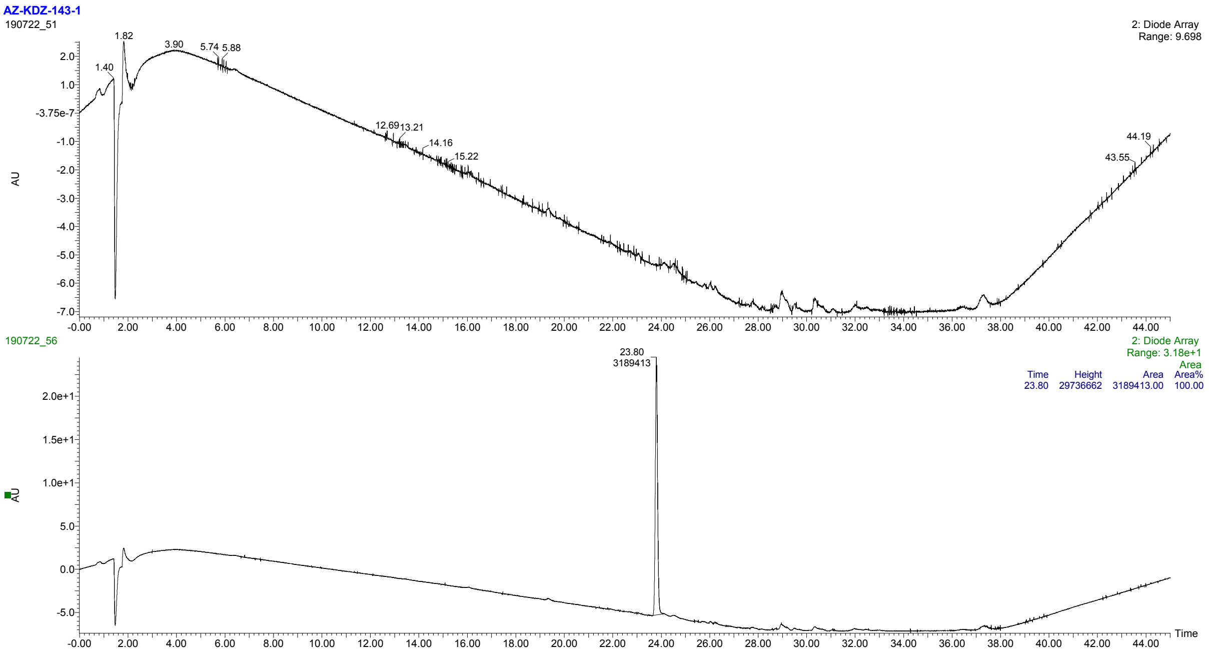

Figure S1.39 LC-MS chromatogram of compound **i-8**.

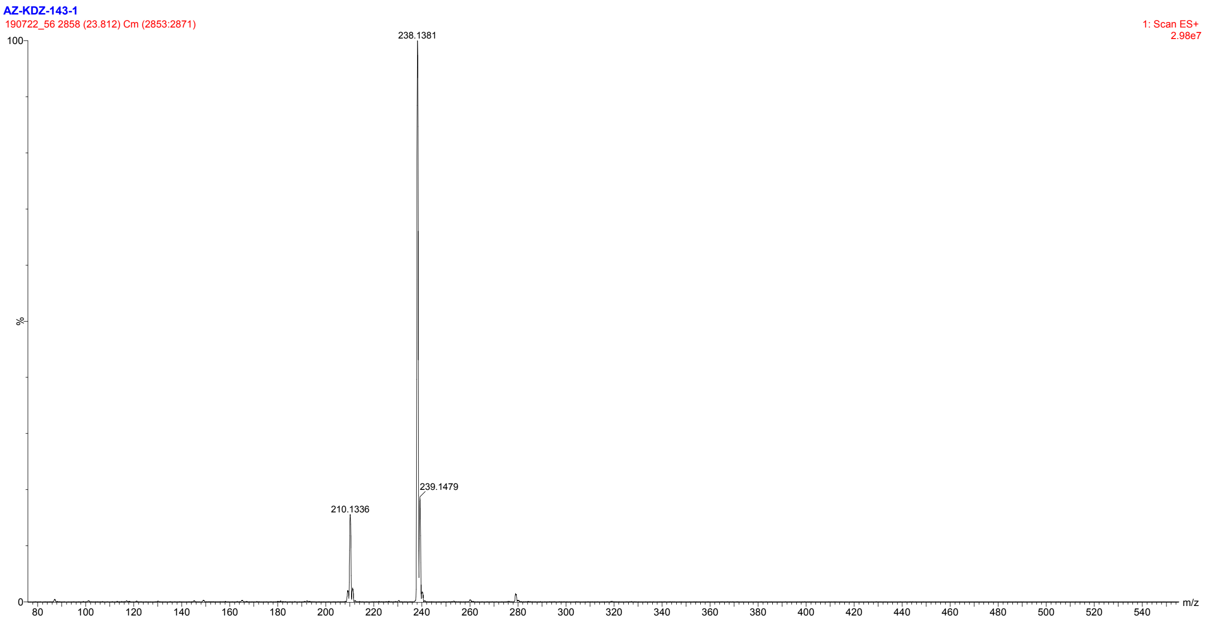

Figure S1.40 MS spectrum of compound **i-8**.

5-(3-Ethoxyphenyl)-1*H*-indole **i-9**

Prepared in accordance with general procedure **II** from 3-(1*H*-indol-5-yl)phenol **i-2** (40 mg, 0.19 mmol) and iodoethane (23 µL, 0.29 mmol). Yield 76%, light brown viscous oil, R_f = 0.29 (*n*-hexane/ethyl acetate, 4/1). IR (neat) ν_{max}, cm^{-1}: 3411 (N-H), 2978, 1704, 1598, 1462, 1212, 1046, 768, 726, 696. ^{1}H NMR (500 MHz, DMSO-d_6): δ 1.35 (3H, t, *J* = 6.9 Hz, CH_3), 4.10 (2H, q, *J* = 6.9 Hz, CH_2), 6.46-6.49 (1H, m, CH), 6.84 (1H, dd, *J* = 8.0, 1.8 Hz, CH), 7.14-7.16 (1H, m, CH), 7.21 (1H, d, *J* = 7.6 Hz, CH), 7.32 (1H, t, *J* = 7.9 Hz, CH), 7.36-7.40 (2H, m, 2×CH), 7.45 (1H, d, *J* = 8.4 Hz, CH), 7.81 (1H,s, CH), 11.14 (1H, br s, NH). ^{13}C NMR (125 MHz, DMSO-d_6): δ 14.8 (CH_3), 63.0 (CH_2), 101.6 (CH), 111.8 (CH), 112.3 (CH), 112.6 (CH), 118.3 (CH), 119.0 (CH), 120.5 (CH), 126.1 (CH), 128.2 (C), 129.8 (CH), 131.3 (C), 135.6 (CH), 143.5 (C), 159.0 (C). MS (pos. mode): m/z (%): 238.2 ($M+H^+$, 100%).

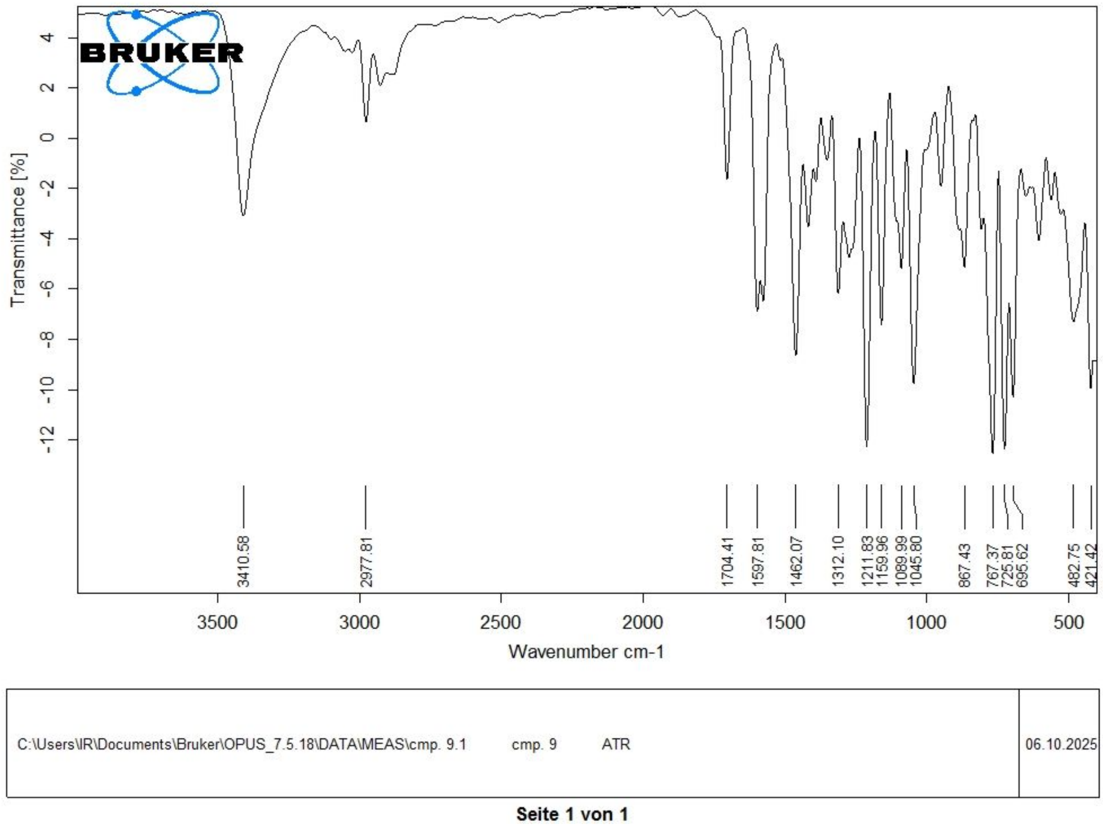

Figure S1.41 FTIR spectrum of compound **i-9**.

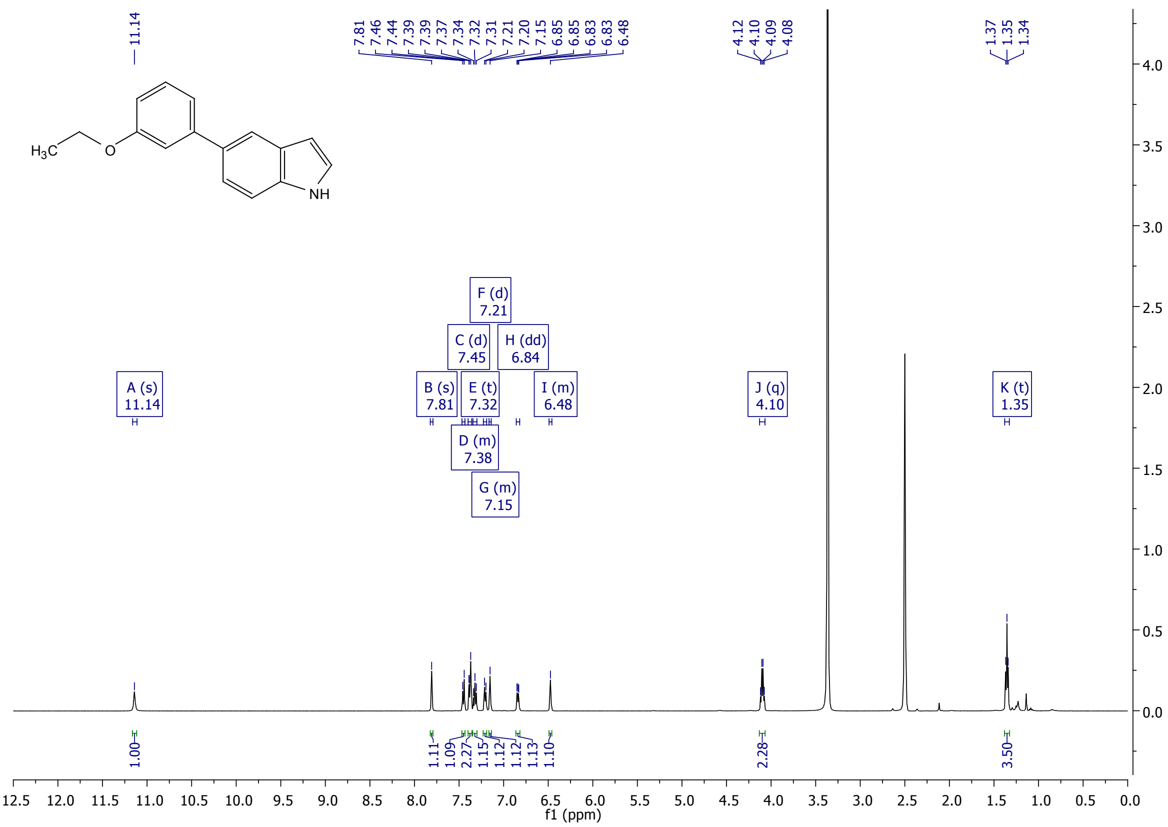

Figure S1.42 ^{1}H NMR spectrum of compound **i-9** in DMSO-d_6.

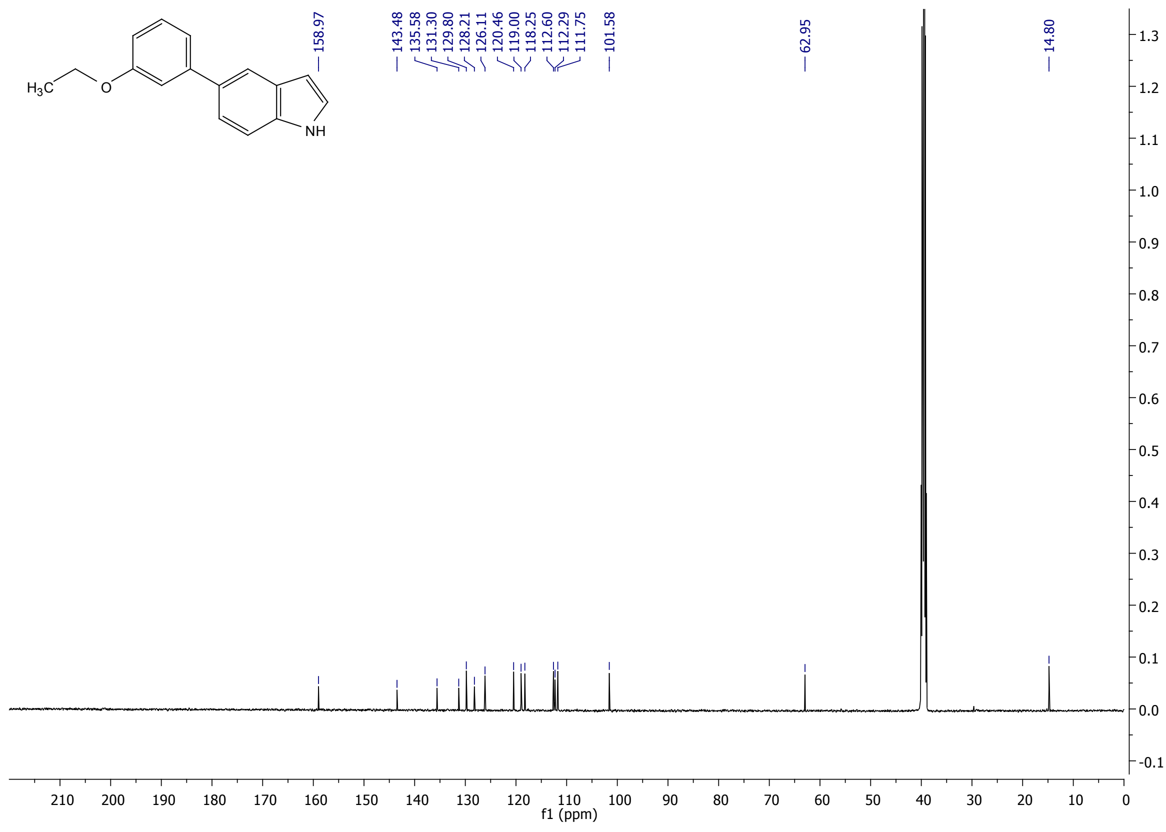

Figure S1.43 ^{13}C NMR spectrum of compound **i-9** in DMSO-d_6.

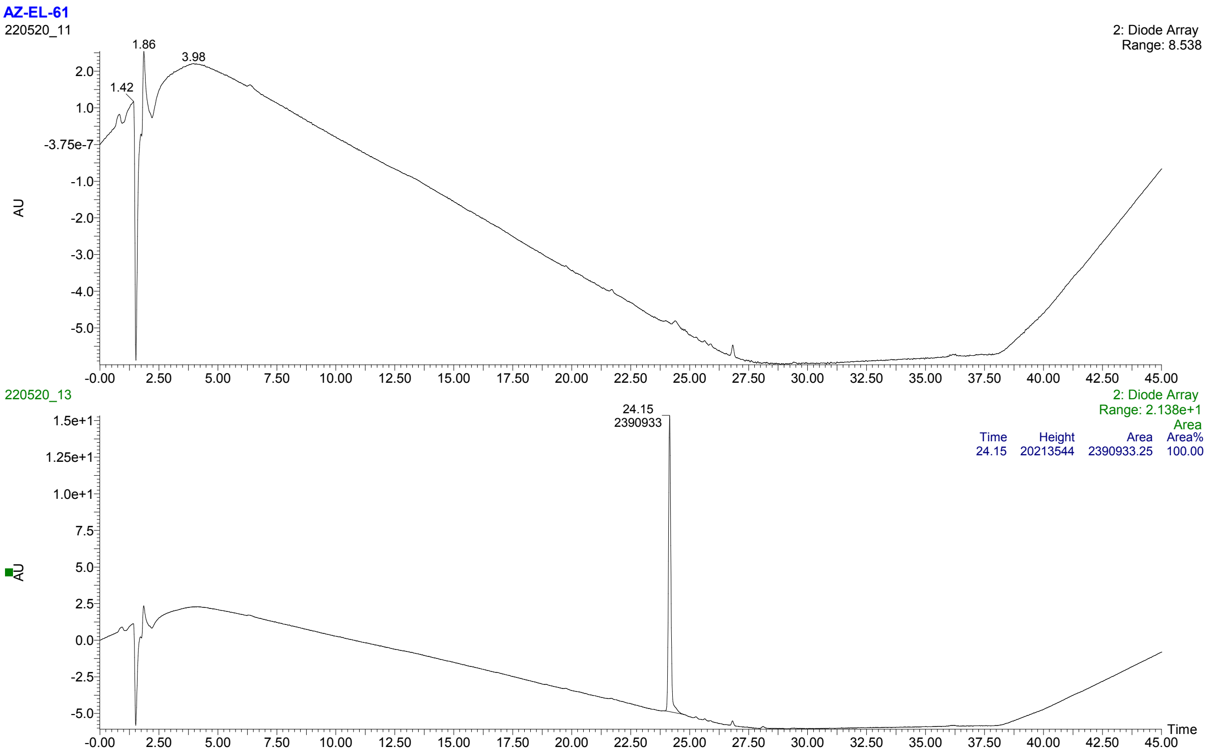

Figure S1.44 LC-MS chromatogram of compound **i-9**.

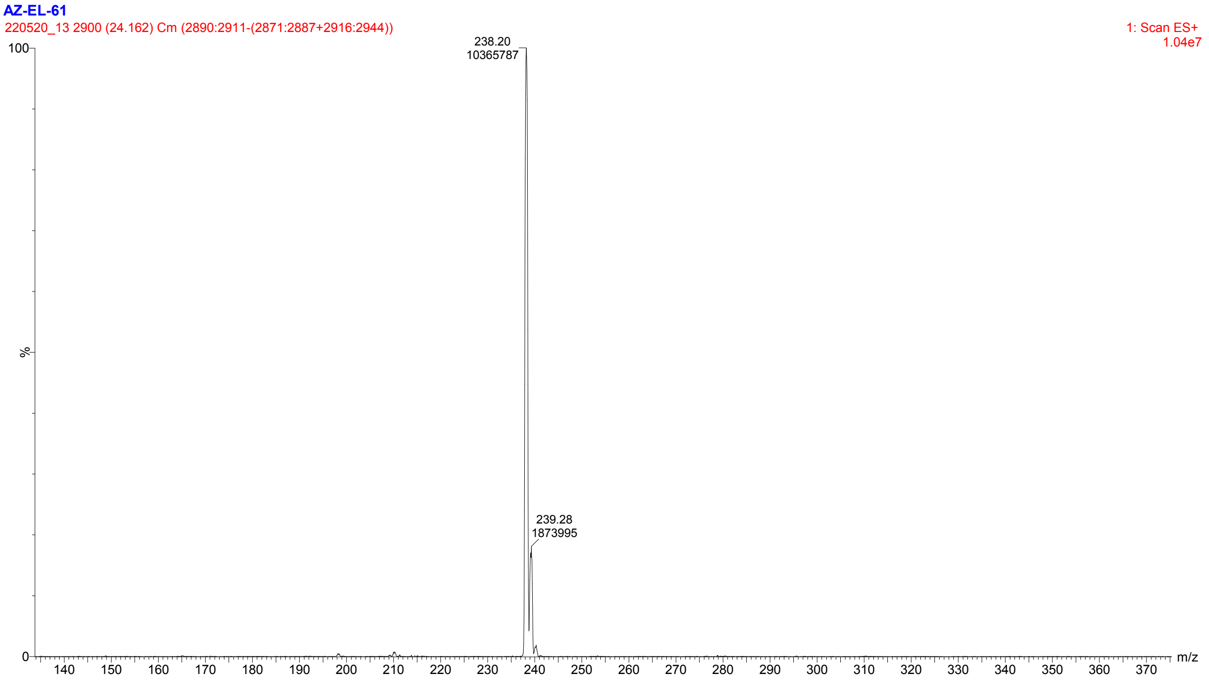

Figure S1.45 MS spectrum of compound **i-9**.

5-(4-Ethoxyphenyl)-1*H*-indole **i-10**

Prepared in accordance with general procedure **I** from 5-bromo-1*H*-indole (137 mg, 0.70 mmol) and (4-ethoxyphenyl)boronic acid (174 mg, 1.05 mmol). Yield 83%, white solid; mp = 102.3–102.9 °C, R_f = 0.27 (*n*-hexane/ethyl acetate, 4/1). IR (neat) ν_{max}, cm^{-1}: 3392 (N-H), 1460, 1237, 1045, 798, 730, 536, 427. ^{1}H NMR (500 MHz, DMSO-d_6): δ 1.34 (3H, t, *J* = 6.9 Hz, CH_3), 4.05 (2H, q, *J* = 6.7 Hz, CH_2), 6.42-6.49 (1H, m, CH), 6.97 (2H, d, *J* = 8.4 Hz, 2×CH), 7.30-7.38 (2H, m, 2×CH), 7.43 (1H, d, *J* = 8.3 Hz, CH), 7.56 (2H, d, *J* = 8.4 Hz, 2×CH), 7.71-7.75 (1H, m, CH), 11.10 (1H, br s, NH). ^{13}C NMR (125 MHz, DMSO-d_6): δ 14.8 (CH_3), 63.0 (CH_2), 101.4 (CH), 111.7 (CH), 114.7 (2×CH), 117.5 (CH), 120.1 (CH), 125.9 (CH), 127.7 (2×CH), 128.3 (C), 131.2 (C), 134.3 (C), 135.1 (C), 157.3 (C). MS (pos. mode): m/z (%): 238.3 (M+H$^+$, 100%).

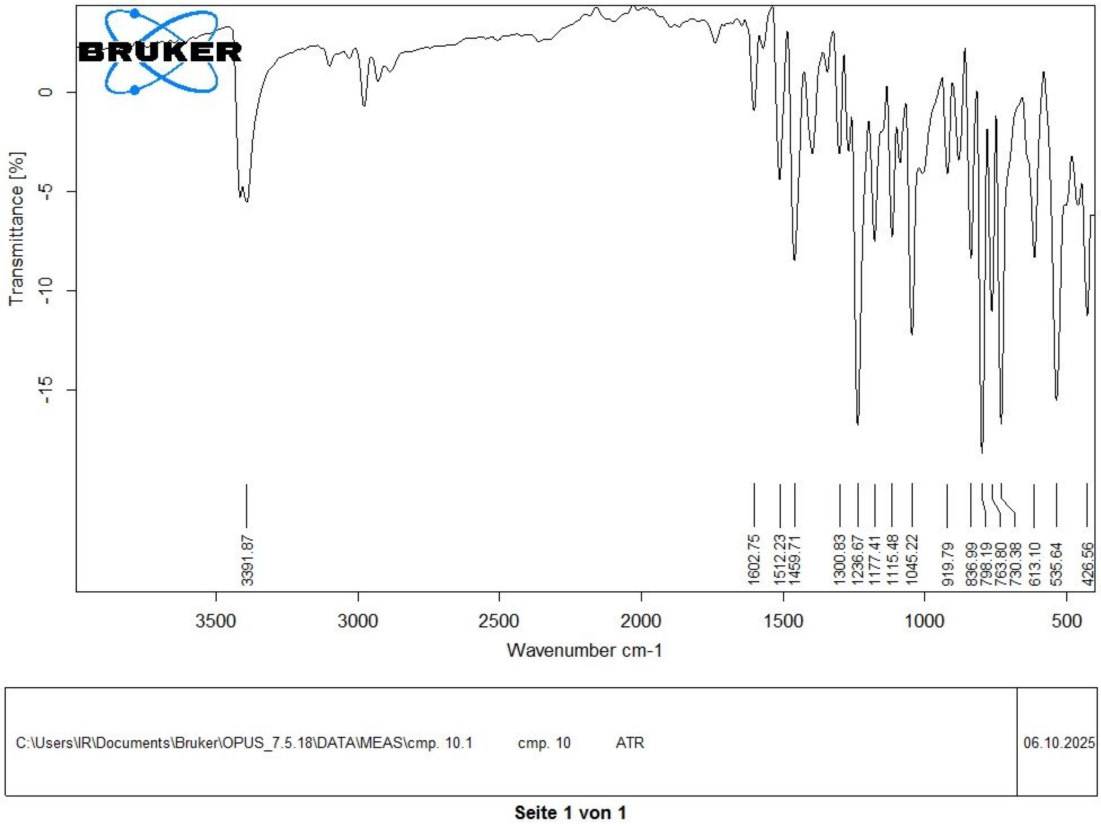

Figure S1.46 FTIR spectrum of compound **i-10**.

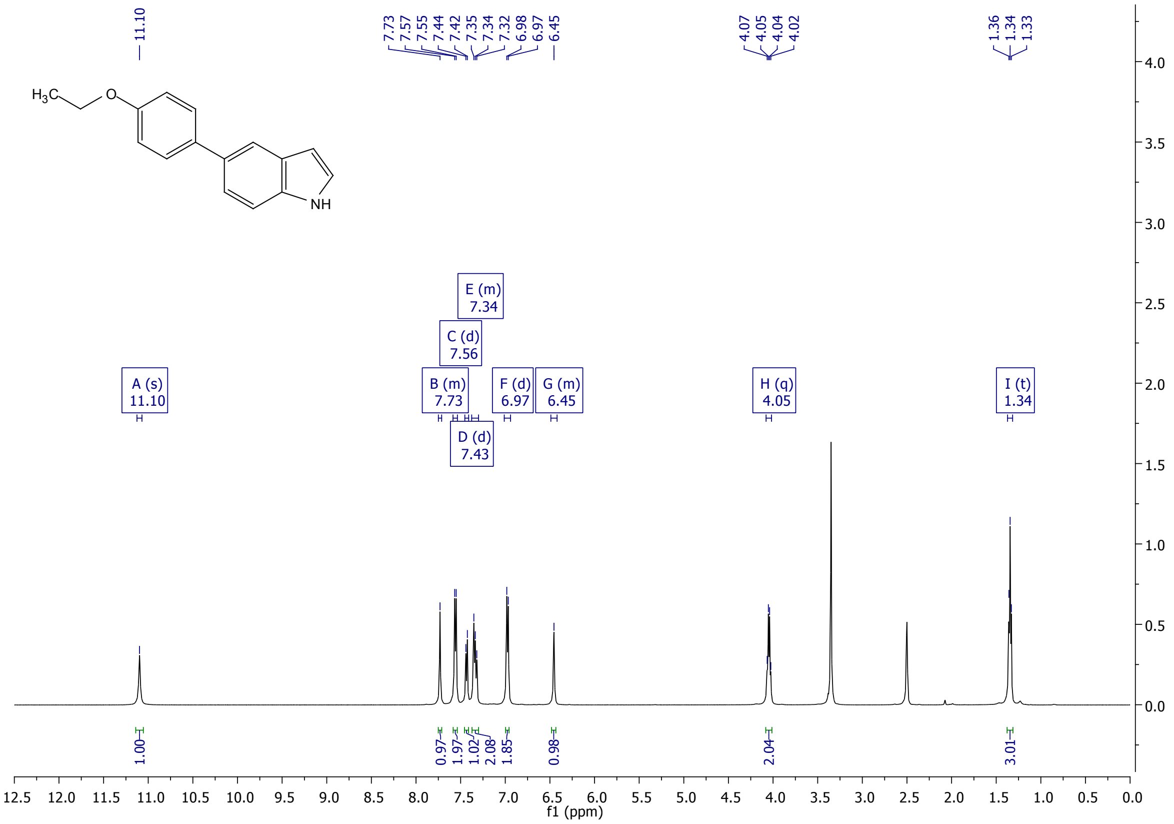

Figure S1.47 ^{1}H NMR spectrum of compound **i-10** in DMSO-d_6.

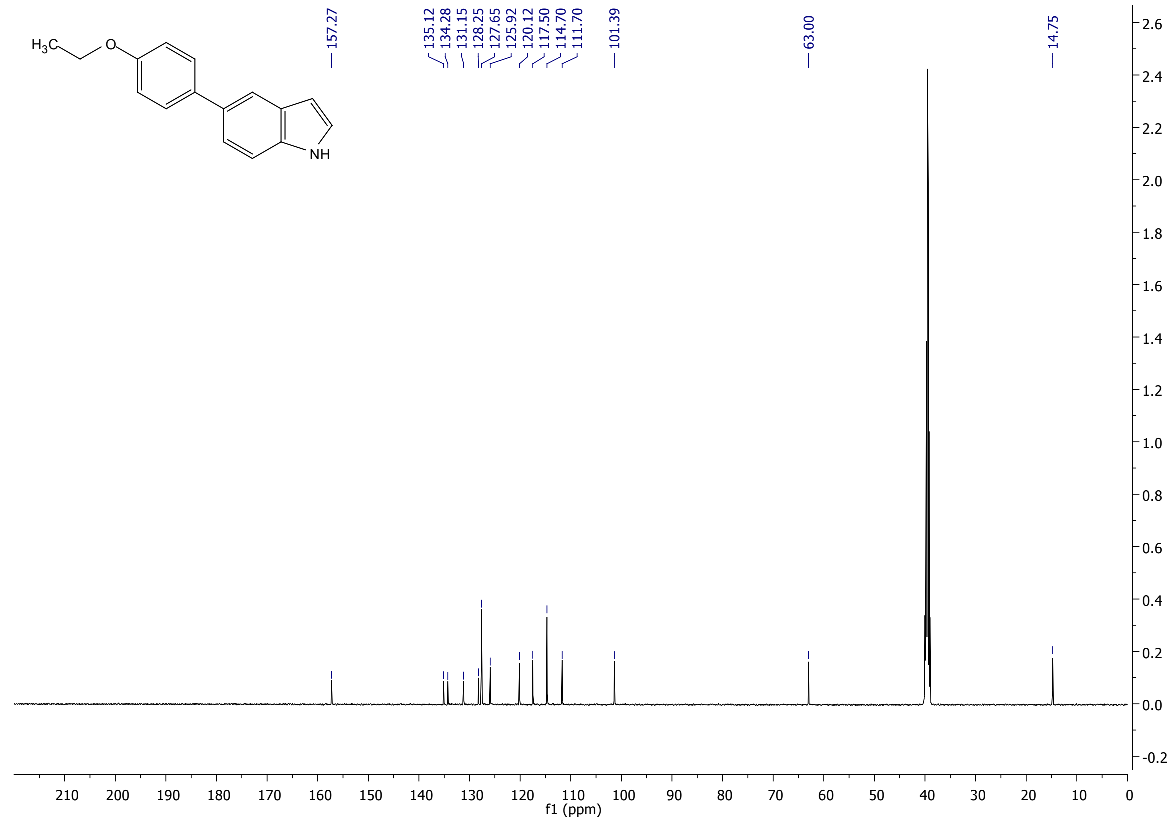

Figure S1.48 ^{13}C NMR spectrum of compound **i-10** in DMSO-d_6.

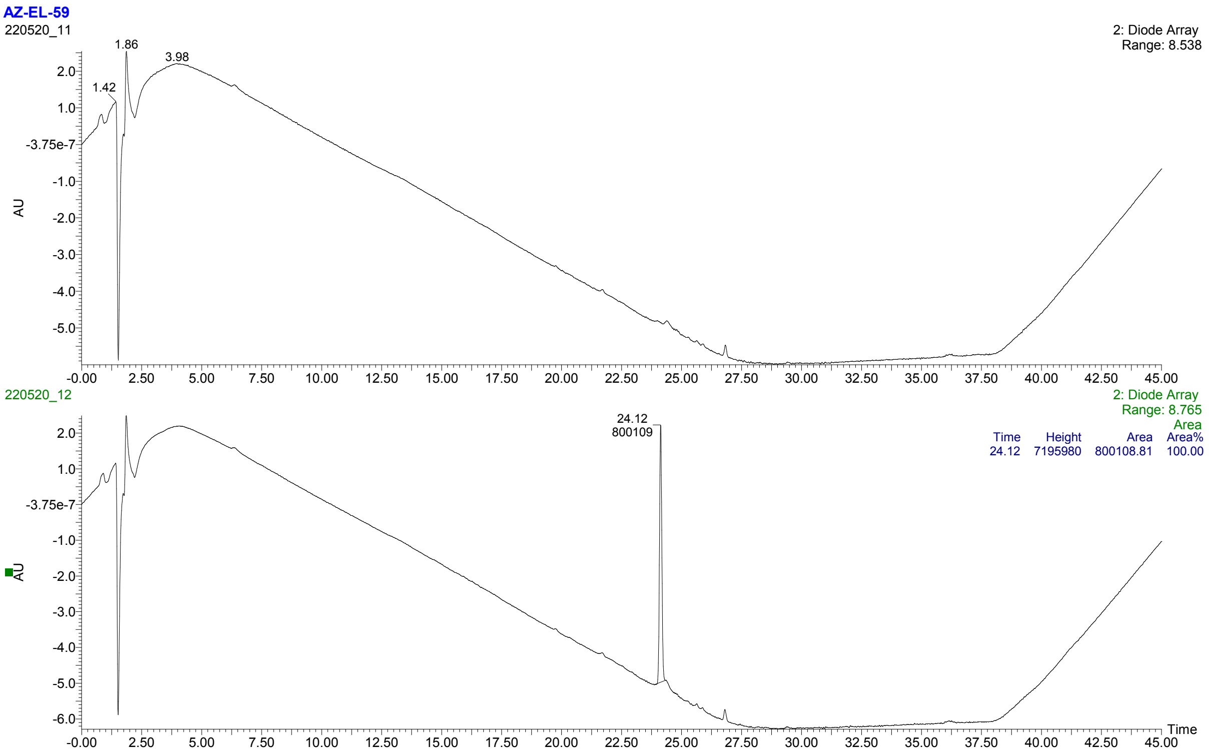

Figure S1.49 LC-MS chromatogram of compound **i-10**.

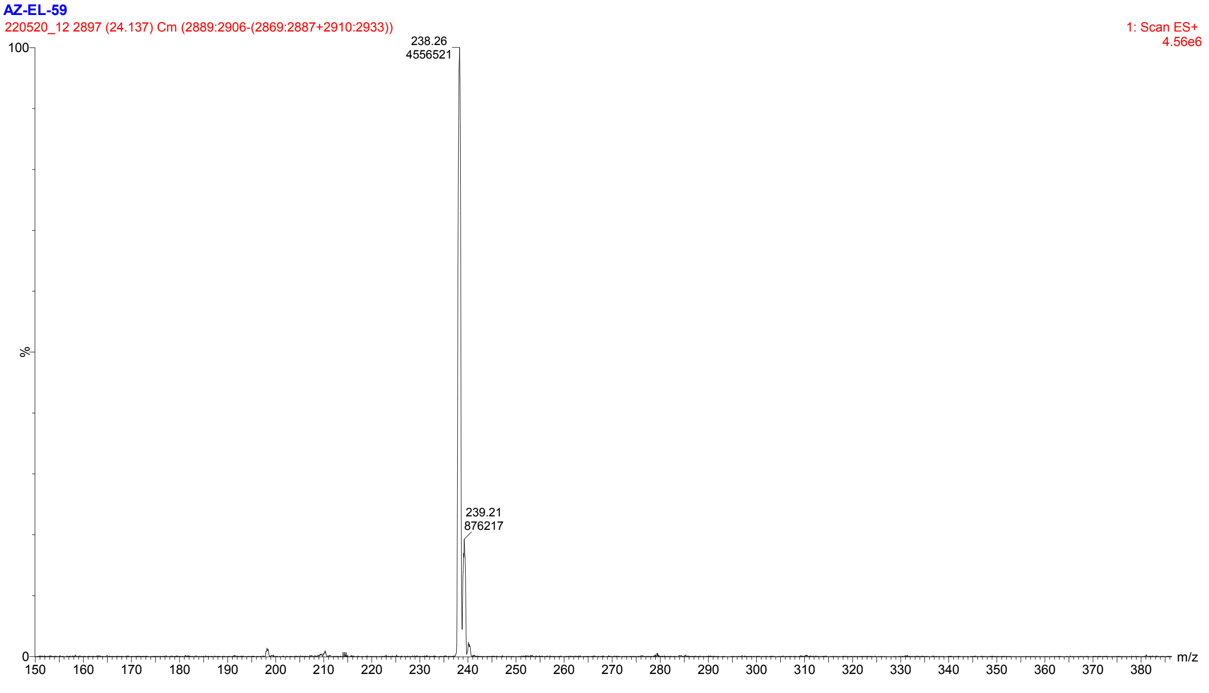

Figure S1.50 MS spectrum of compound **i-10**.

5-(2-Propoxyphenyl)-1*H*-indole **i-11**

Prepared in accordance with general procedure **II** from 2-(1*H*-indol-5-yl)phenol **i-2** (21 mg, 0.10 mmol) and 1-iodopropane (15 µL, 0.15 mmol). Yield 58%, light brown viscous oil, $R_f = 0.38$ (*n*-hexane/ethyl acetate, 4/1). IR (neat) ν_{max}, cm^{-1}: 3410 (N-H), 2965, 1462, 1226, 1058, 974, 887, 745. ^1H NMR (500 MHz, DMSO-d_6): δ 0.91 (3H, t, J = 7.4 Hz, CH_3), 1.64 (2H, sext, J = 7.0 Hz, CH_2), 3.92 (2H, t, J = 6.3 Hz, CH_2), 6.41-6.44 (1H, m, CH), 6.99 (1H, t, J = 7.4, CH), 7.06 (1H, d, J = 8.0, CH), 7.23-7.28 (2H, m, 2×CH), 7.30 (1H, dd, J = 7.5, 1.6 Hz, CH), 7.33-7.35 (1H, m, CH), 7.39 (1H, d, J = 8.4 Hz, CH), 7.62-7.65 (1H, m, CH), 11.09 (1H, br s, NH). ^{13}C NMR (125 MHz, DMSO-d_6): δ 10.6 (CH_3), 22.2 (CH_2), 69.3 (CH_2), 101.3 (CH), 110.6 (CH), 112.7 (CH), 120.65 (CH), 120.66 (CH), 123.0 (CH), 125.5 (CH), 127.5 (C), 127.7 (CH), 129.0 (C), 130.8 (CH), 131.6 (C), 134.9 (C), 155.6 (C). MS (pos. mode): m/z (%): 252.2 ($M+H^+$, 100%).

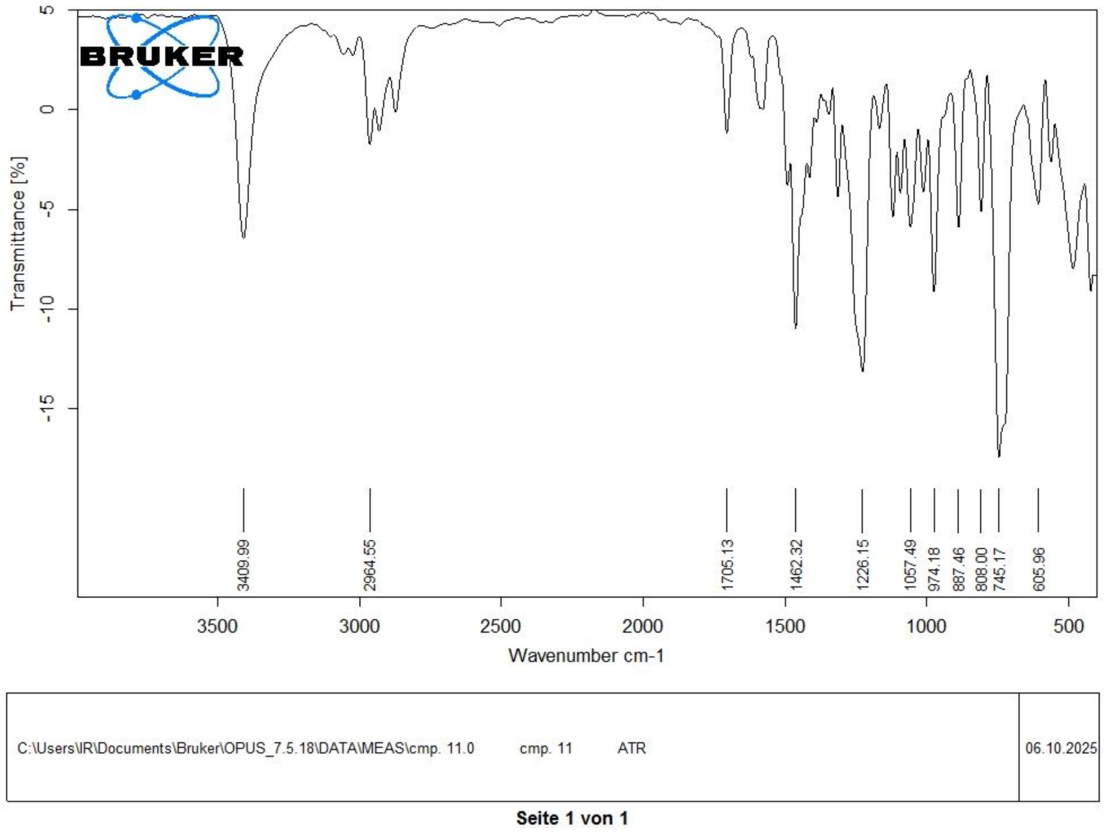

Figure S1.51 FTIR spectrum of compound **i-11**.

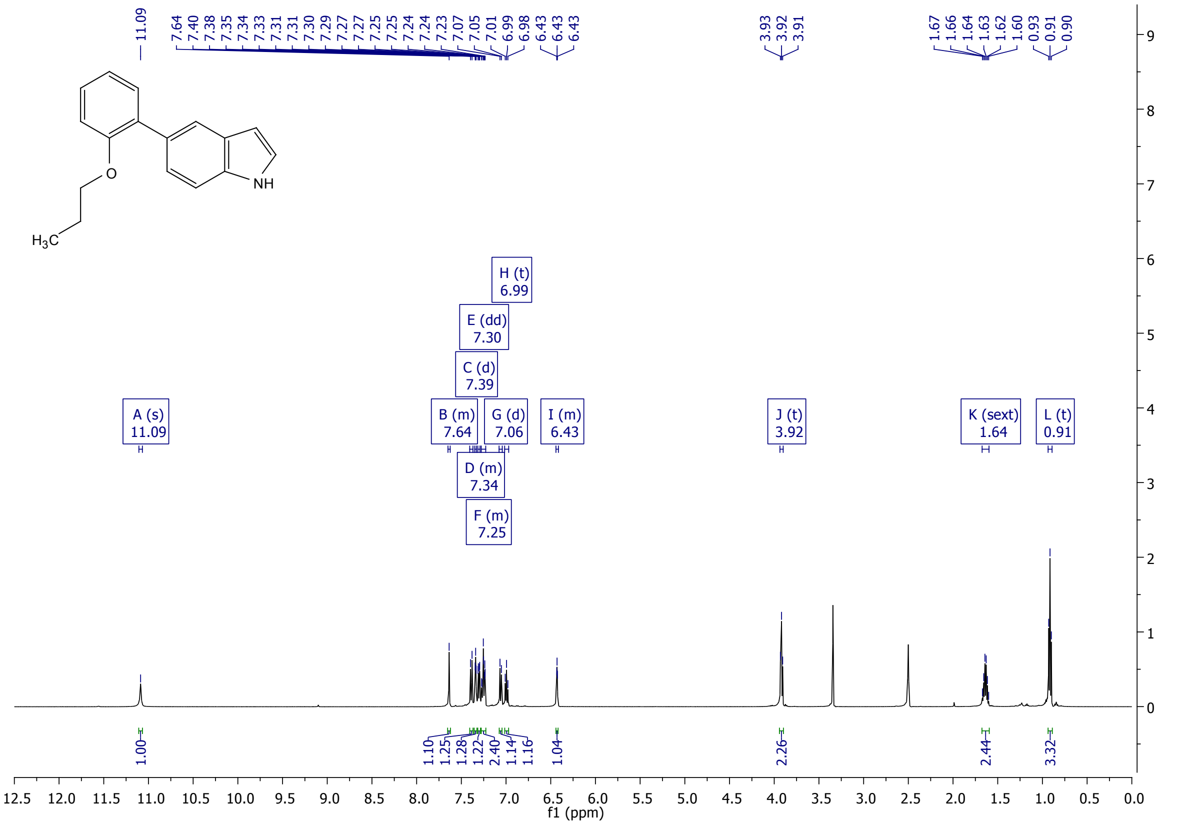

Figure S1.52 ^{1}H NMR spectrum of compound **i-11** in DMSO-d_6.

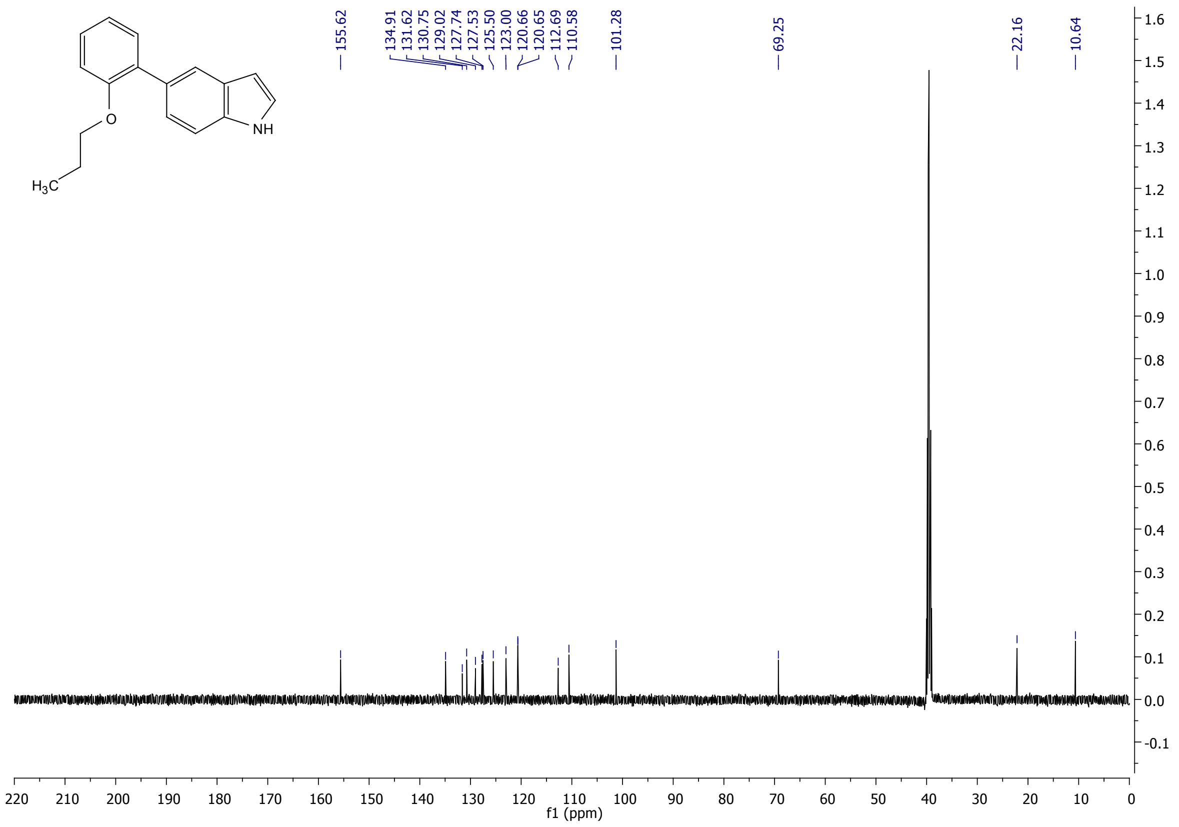

Figure S1.53 ^{13}C NMR spectrum of compound **i-11** in DMSO-d_6.

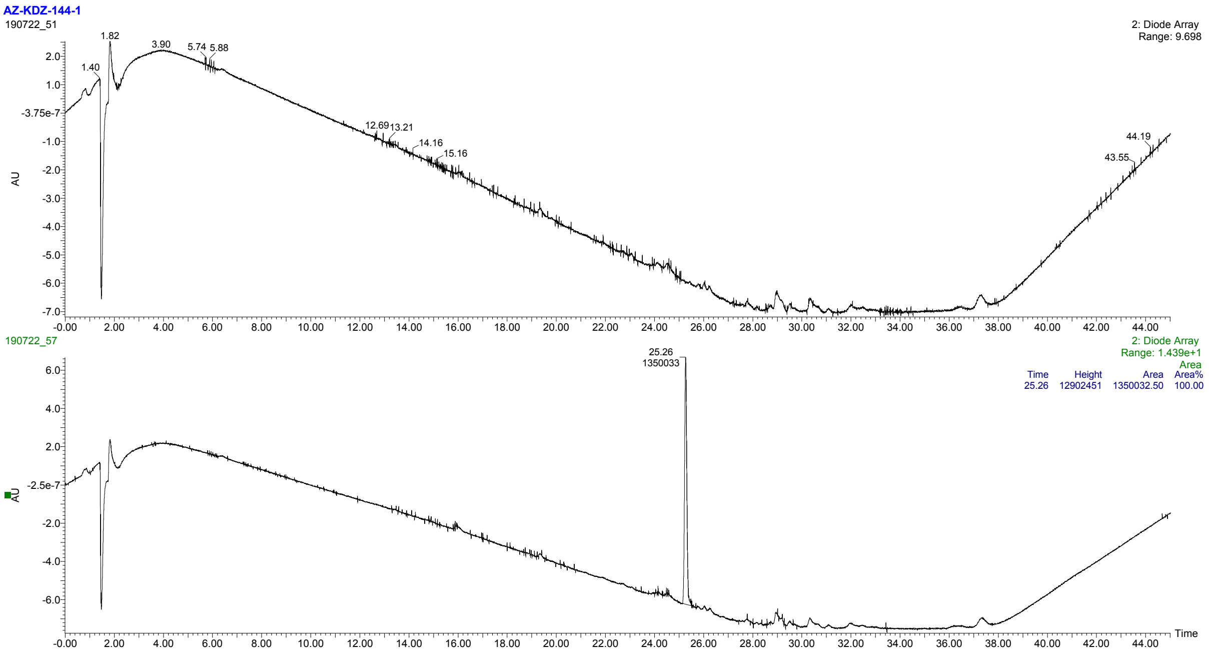

Figure S1.54 LC-MS chromatogram of compound **i-11**.

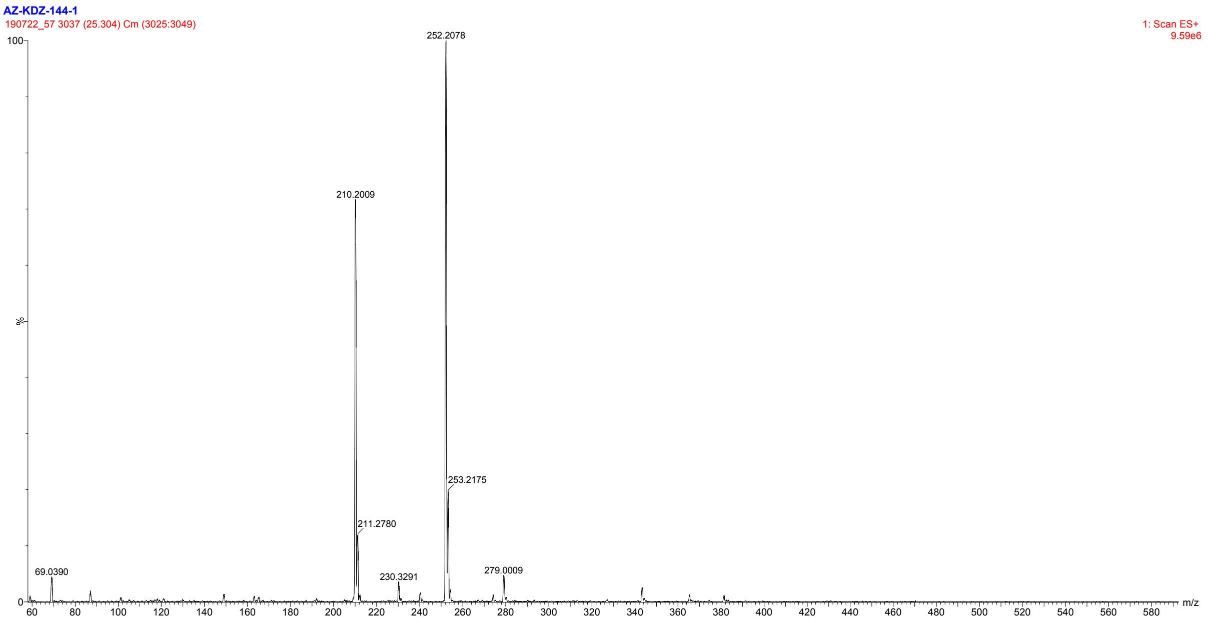

Figure S1.55 MS spectrum of compound **i-11**.

5-(3-Propoxyphenyl)-1*H*-indole **i-12**

Prepared in accordance with general procedure **II** from 3-(1*H*-indol-5-yl)phenol **i-2** (40 mg, 0.19 mmol) and 1-iodopropane (28 µL, 0.29 mmol). Yield 44%, beige solid; mp = 67.1–68.5 °C, R_f = 0.33 (*n*-hexane/ethyl acetate, 4/1). IR (neat) ν_{max}, cm^{-1}: 3407 (N-H), 1572, 1456, 1211, 978, 782, 724, 489. ^{1}H NMR (500 MHz, DMSO-d_6): δ 1.00 (3H, t, J = 7.4 Hz, CH_3), 1.76 (2H, sext, J = 7.1 Hz, CH_2), 4.00 (2H, t, J = 6.6 Hz, CH_2), 6.47-6.48 (1H, m, CH), 6.85 (1H, ddd, J = 8.2, 2.5, 0.8 Hz, CH), 7.15-7.17 (1H, m, CH), 7.21 (1H, ddd, J = 7.7, 1.6, 0.9 Hz, CH), 7.32 (1H, t, J = 7.9 Hz, CH), 7.36-7.40 (2H, m, 2×CH), 7.44-7.46 (1H, m, CH), 7.81-7.82 (1H, m, CH), 11.14 (1H br s, NH). ^{13}C NMR (125 MHz, DMSO-d_6): δ 10.5 (CH_3), 22.2 (CH_2), 68.9 (CH_2), 101.6 (CH), 111.7 (CH), 112.3 (CH), 112.6 (CH), 118.2 (CH), 119.0 (CH), 120.4 (CH), 126.1 (CH), 128.2 (C), 129.8 (CH), 131.3 (C), 135.6 (C), 143.5 (C), 159.1 (C). MS (pos. mode): m/z (%): 252.3 (M+H$^+$, 100%).

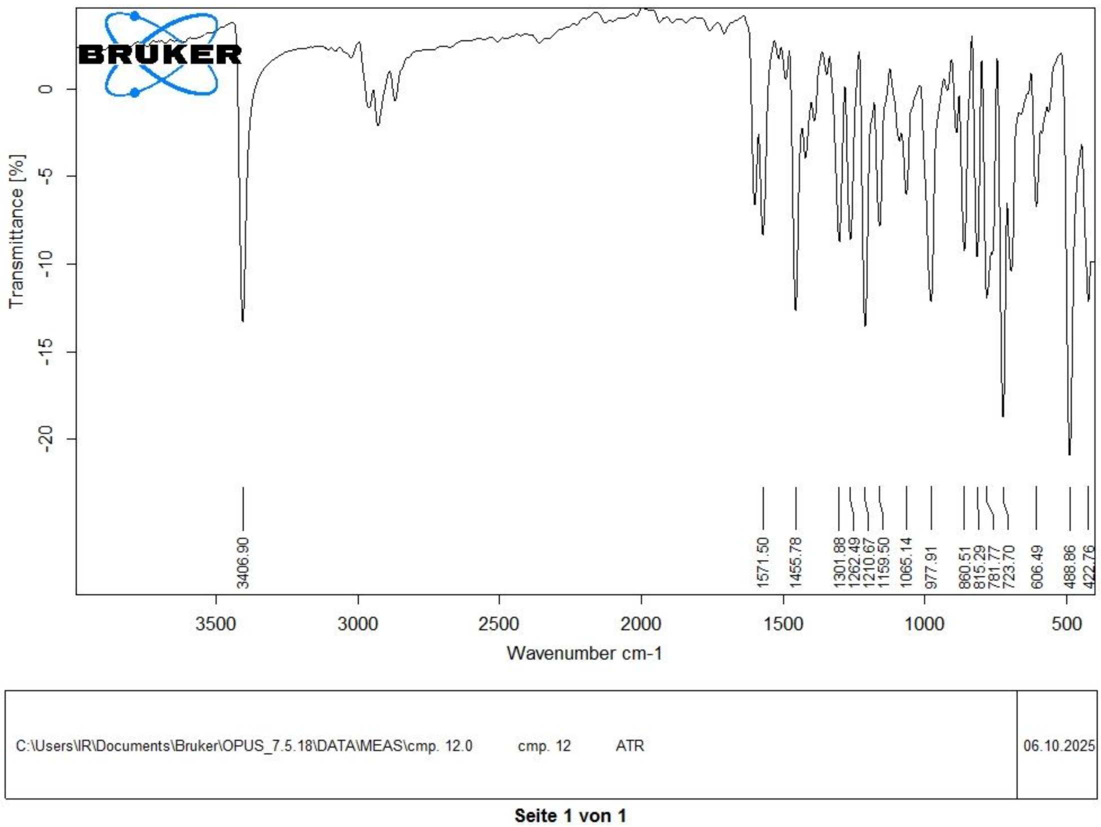

Figure S1.56 FTIR spectrum of compound **i-12**.

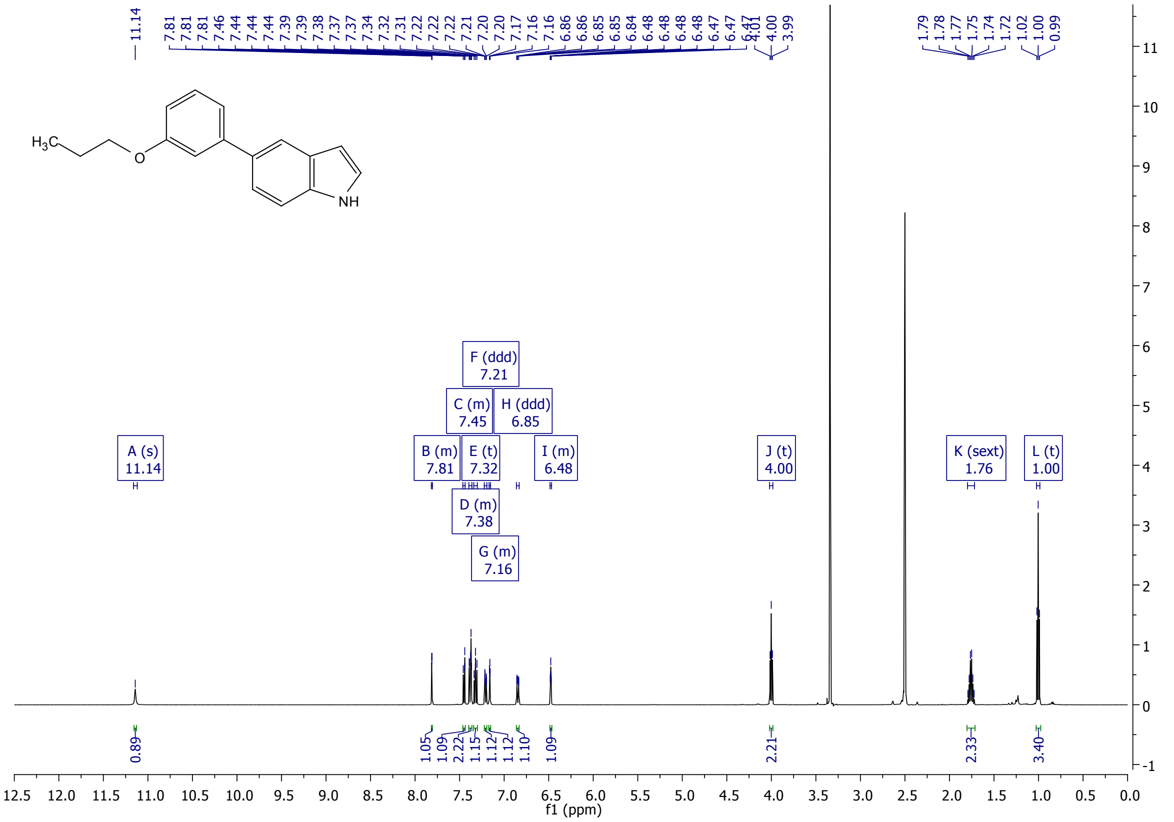

Figure S1.57 ^{1}H NMR spectrum of compound **i-12** in DMSO-d_6.

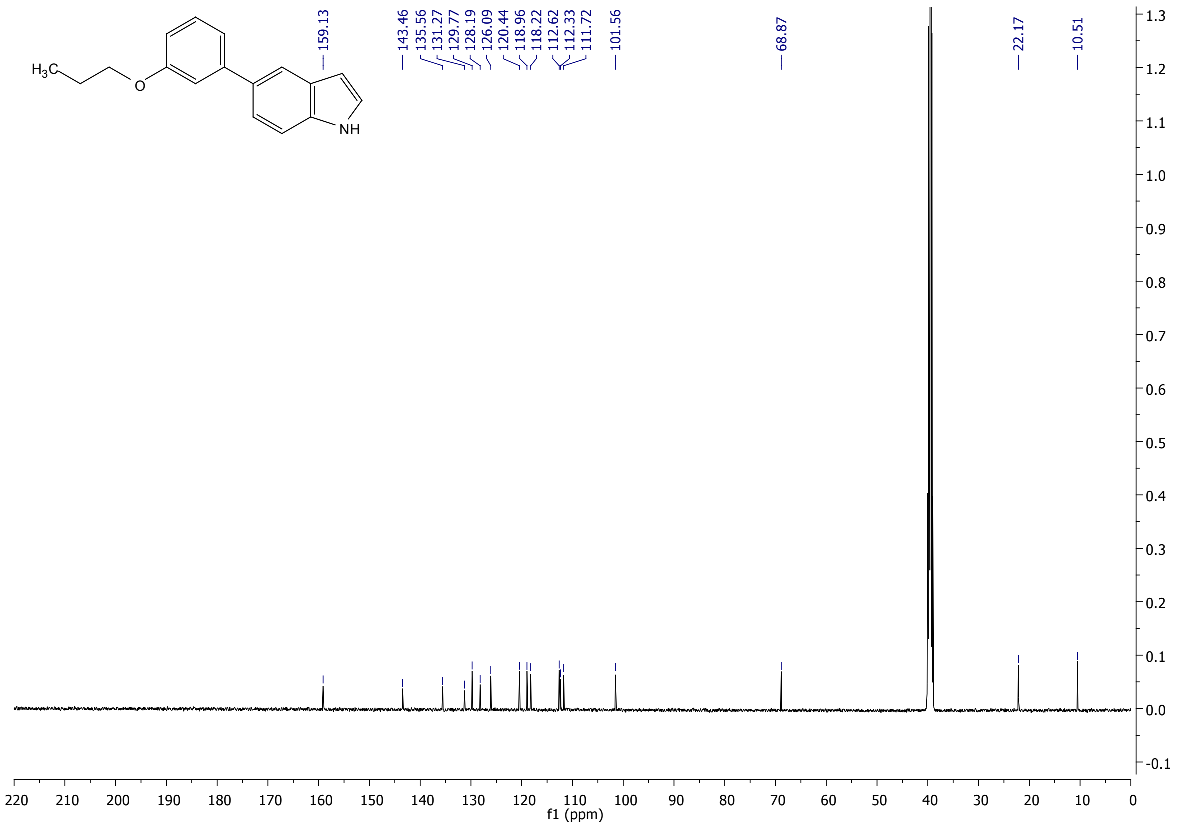

Figure S1.58 ^{13}C NMR spectrum of compound **i-12** in DMSO-d_6.

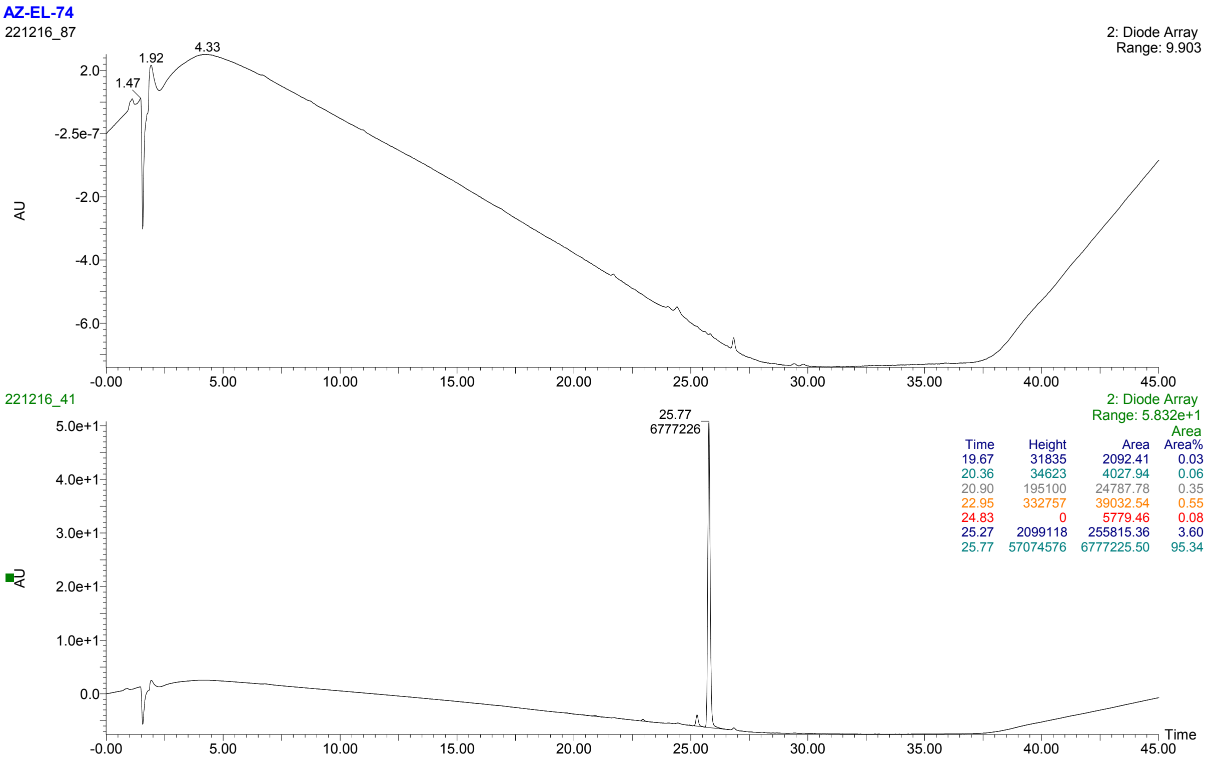

Figure S1.59 LC-MS chromatogram of compound **i-12**.

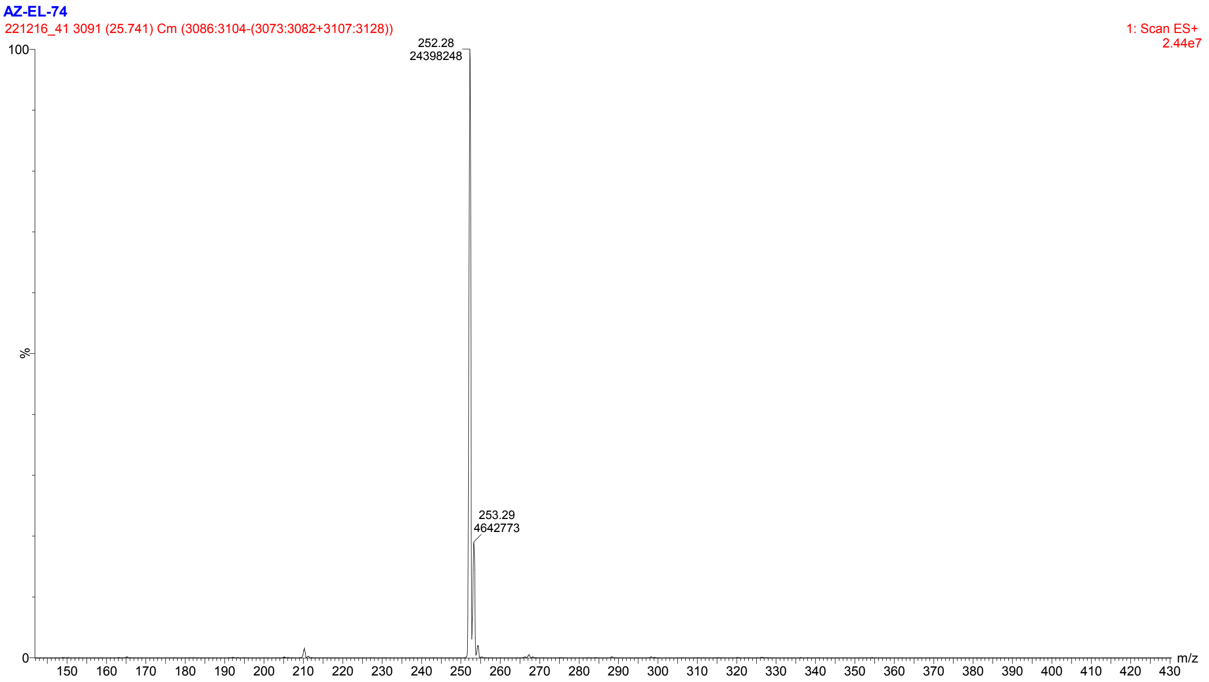

Figure S1.60 MS spectrum of compound **i-12**.

5-(4-Propoxyphenyl)-1*H*-indole **i-13**

Prepared in accordance with general procedure **I** from 5-bromo-1*H*-indole (137 mg, 0.70 mmol) and (4-propoxyphenyl)boronic acid (189 mg, 1.05 mmol). Yield 83%, pale yellow solid; mp = 73.4–74.2 °C, R_f = 0.31 (*n*-hexane/ethyl acetate, 4/1). IR (neat) ν_{max}, cm^{-1}: 3430 (N-H), 1513, 1462, 1242, 1175, 971, 801, 725, 489.^{1}H NMR (500 MHz, DMSO-d_6): δ 0.99 (3H, t, J = 7.4 Hz, CH_3), 1.74 (2H, sext, J = 7.0 Hz, CH_2), 3.94 (2H, t, J = 6.5 Hz, CH_2), 6.45-6.47 (1H, m, CH), 6.96-6.99 (2H, m, 2×CH), 7.33 (1H, dd, J = 8.5, 1.8 Hz, CH), 7.35-7.37 (1H, m, CH), 7.44 (1H, d, J = 8.4 Hz, CH), 7.54-7.57 (2H, m, 2×CH), 7.73-7.74 (1H, m, CH), 11.11 (1H, br s, NH). ^{13}C NMR (125 MHz, DMSO-d_6): δ 10.5 (CH_3), 22.1 (CH_2), 68.9 (CH_2), 101.4 (CH), 111.7 (CH), 114.8 (2×CH), 117.5 (CH), 120.1 (CH), 125.9 (CH), 127.7 (2×CH), 128.3 (C), 131.2 (C), 134.3 (C), 135.1 (C), 157.4 (C). MS (pos. mode): m/z (%): 252.3 (M+H$^+$, 100%).

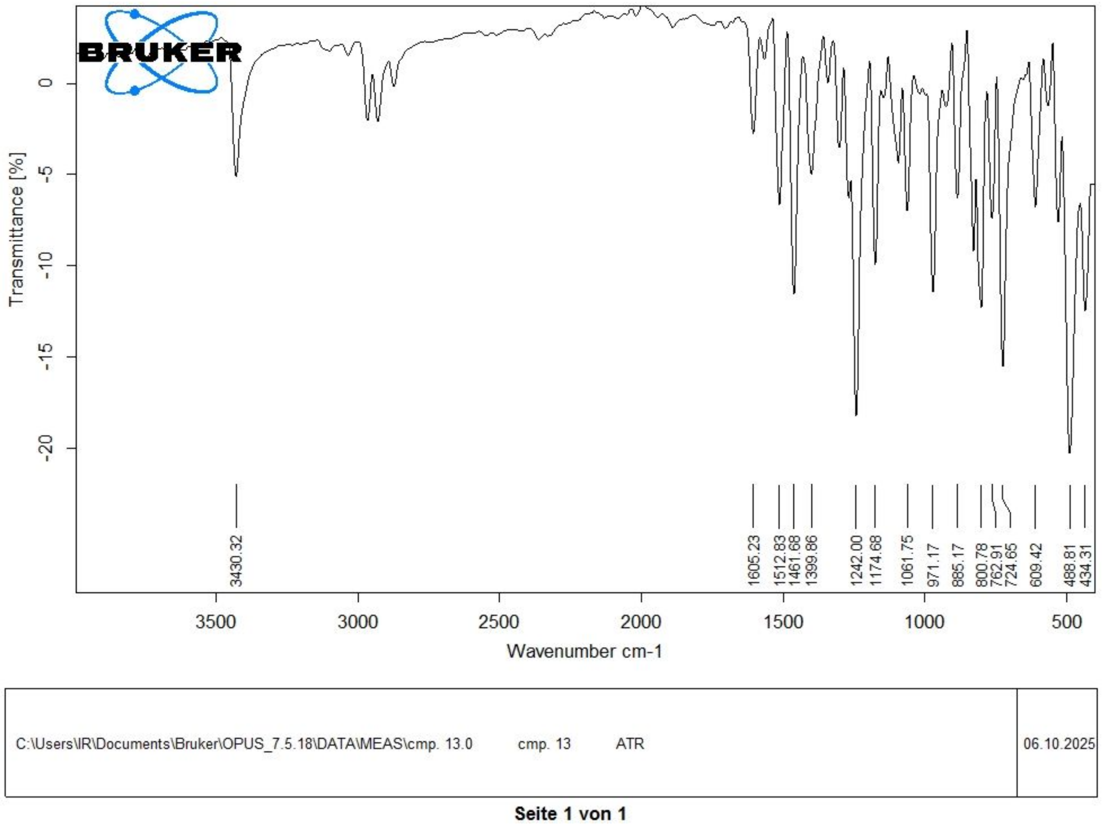

Figure S1.61 FTIR spectrum of compound **i-13**.

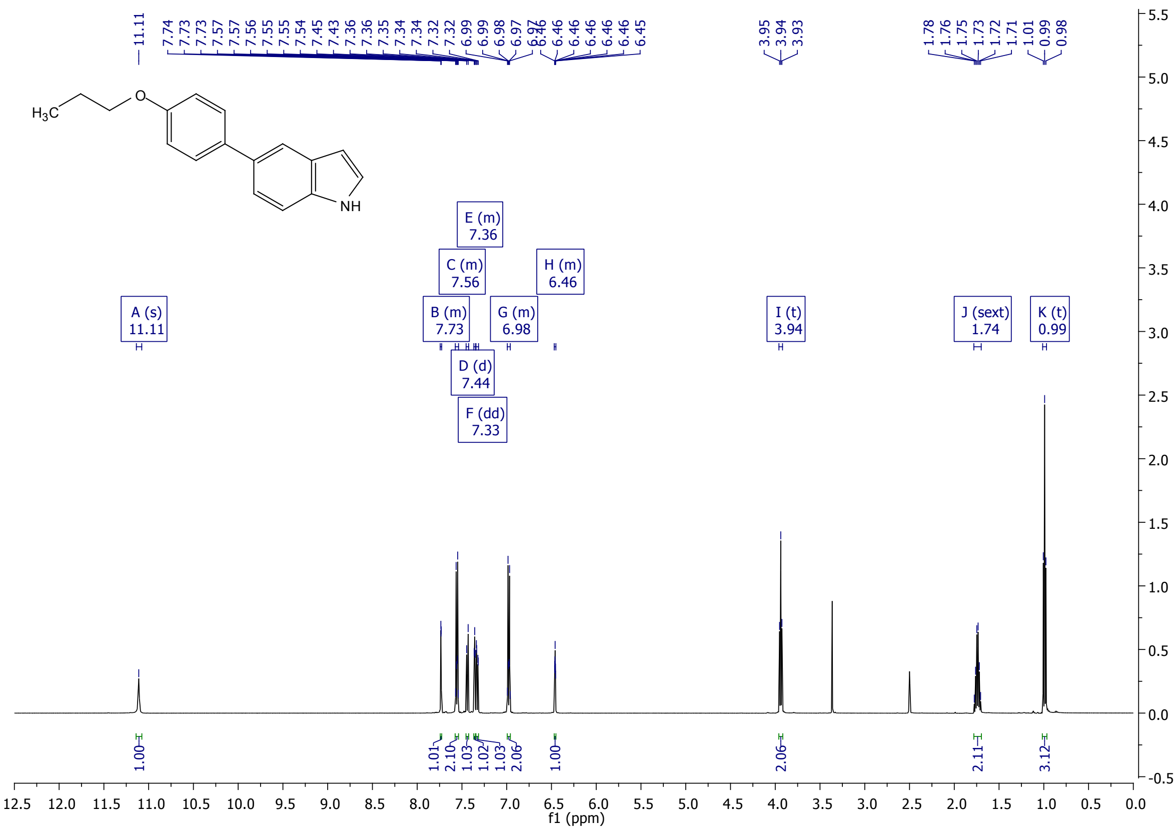

Figure S1.62 ^{1}H NMR spectrum of compound **i-13** in DMSO-d_6.

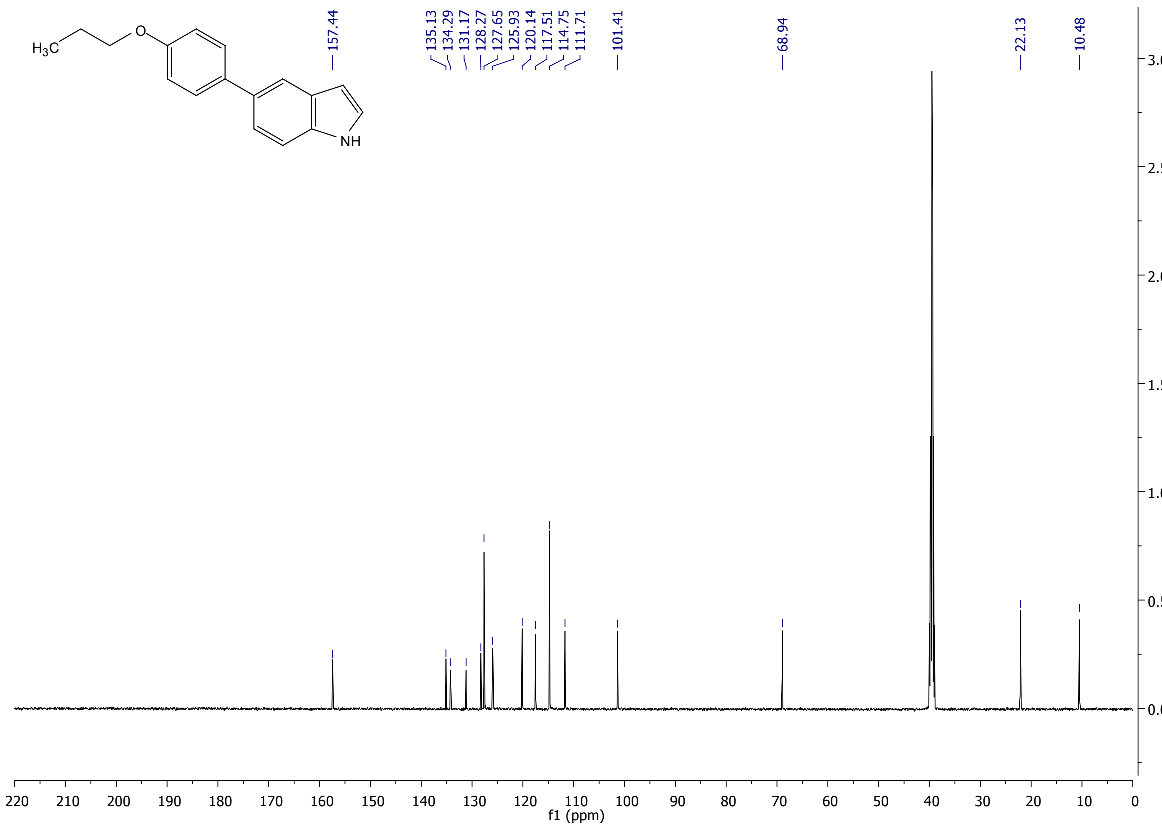

Figure S1.63 ^{13}C NMR spectrum of compound **i-13** in DMSO-d_6.

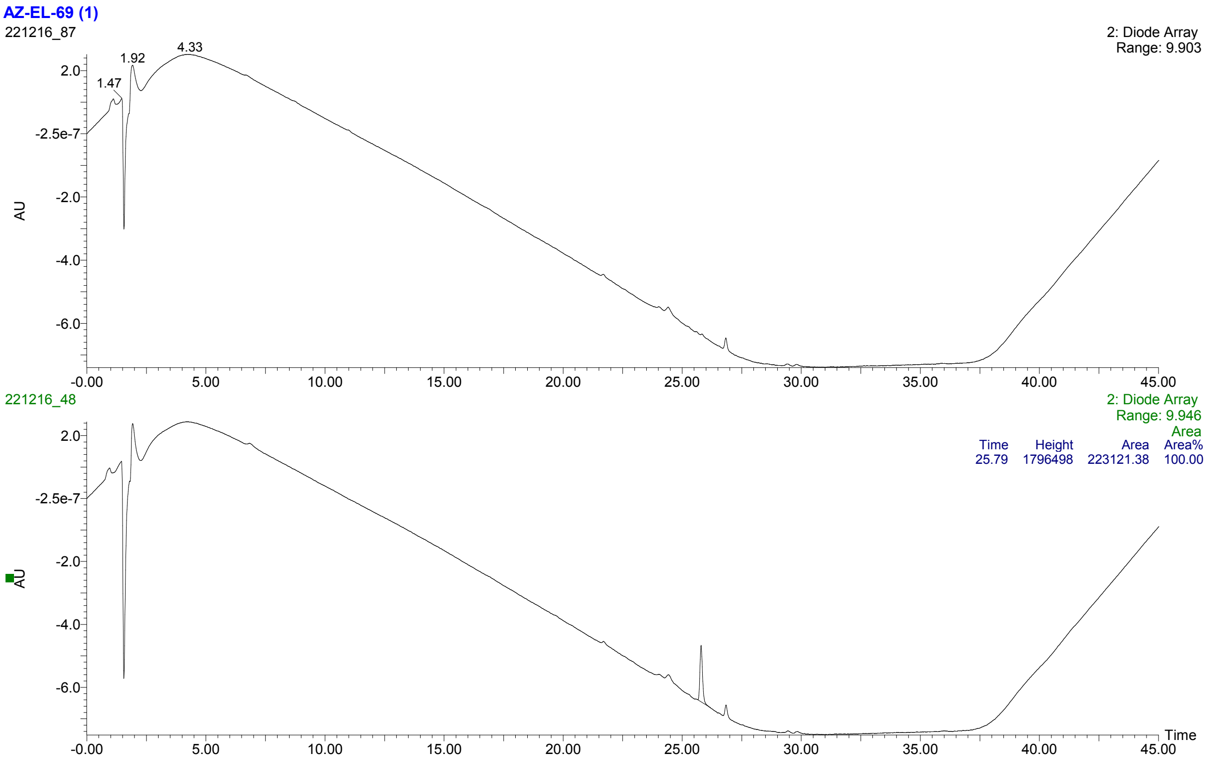

Figure S1.64 LC-MS chromatogram of compound **i-13**.

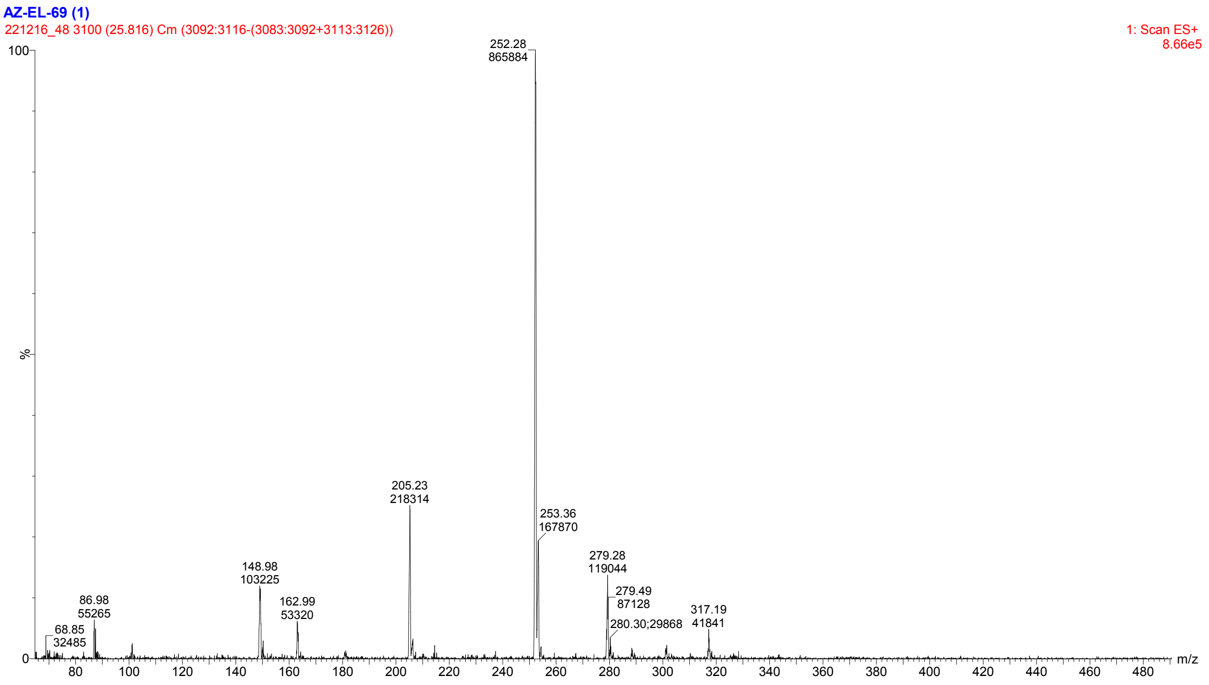

Figure S1.65 MS spectrum of compound **i-13**.

5-(2-Isopropoxyphenyl)-1*H*-indole **i-14**

Prepared in accordance with general procedure **II** from 2-(1*H*-indol-5-yl)phenol **i-2** (32 mg, 0.15 mmol) and 2-iodopropane (23 µL, 0.23 mmol). Yield 47%, pink solid; mp = 73.1–73.9 °C, R_f = 0.32 (*n*-hexane/ethyl acetate, 4/1). IR (neat) ν_{max}, cm^{-1}: 3387 (N-H), 2972, 1466, 1227, 1101, 948, 748, 717, 607, 516. ^{1}H NMR (400 MHz, DMSO-d_6): δ 1.17 (6H, d, *J* = 6.0 Hz, 2×CH_3), 4.48 (1H, hept, *J* = 6.0 Hz, CH), 6.42-6.46 (1H, m, CH), 6.99 (1H, t, *J* = 7.3 Hz, CH), 7.06 (1H, d, *J* = 8.0 Hz, CH), 7.22-7.27 (2H, m, 2×CH), 7.30 (1H, dd, *J* = 7.5, 1.6 Hz, CH), 7.34 (1H, t, *J* = 2.7 Hz, CH), 7.39 (1H, d, *J* = 8.4 Hz, CH), 7.62-7.64 (1H, m, CH), 11.09 (1H, br s, NH). ^{13}C NMR (100 MHz, DMSO-d_6): δ 21.9 (2×CH_3), 69.8 (CH), 101.3 (CH), 110.6 (CH), 115.2 (CH), 120.6 (CH), 120.9 (CH), 123.0 (CH), 125.5 (CH), 127.57 (C), 127.63 (CH), 129.3 (C), 131.1 (CH), 132.7 (C), 134.9 (C), 154.4 (C). MS (pos. mode): m/z (%): 252.3 (M+H$^+$, 70%).

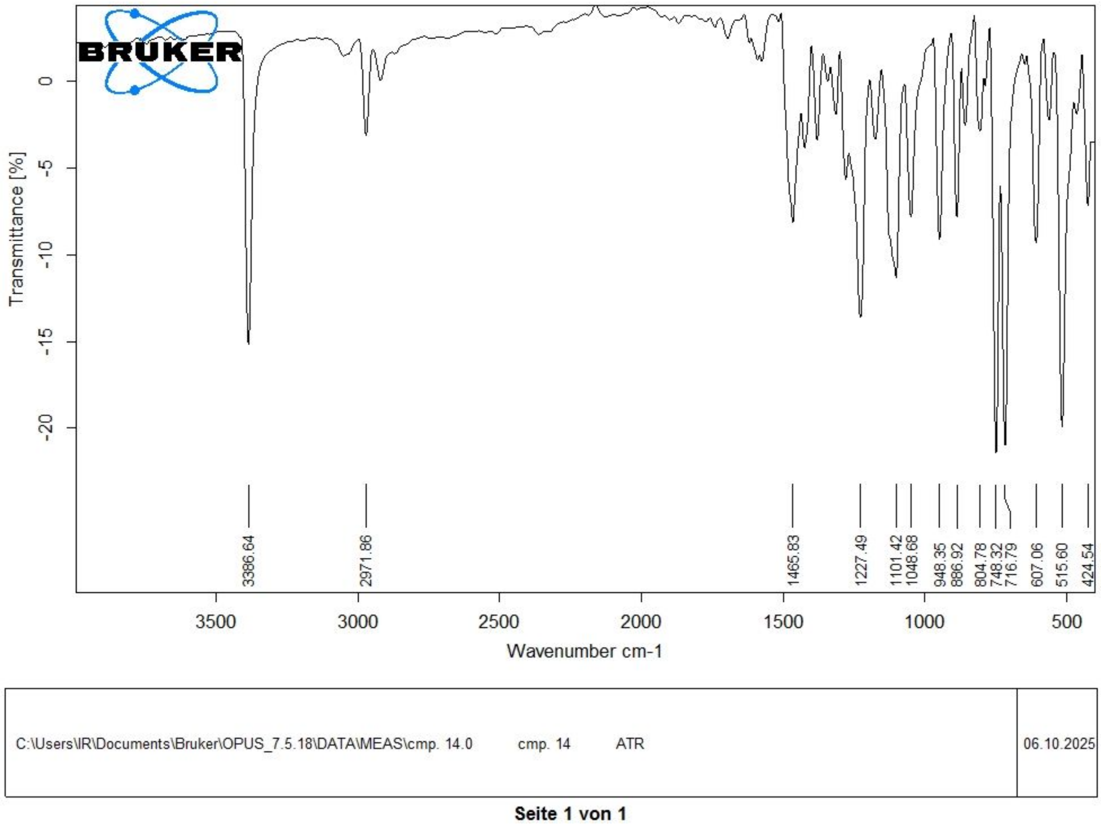

Figure S1.66 FTIR spectrum of compound **i-14**.

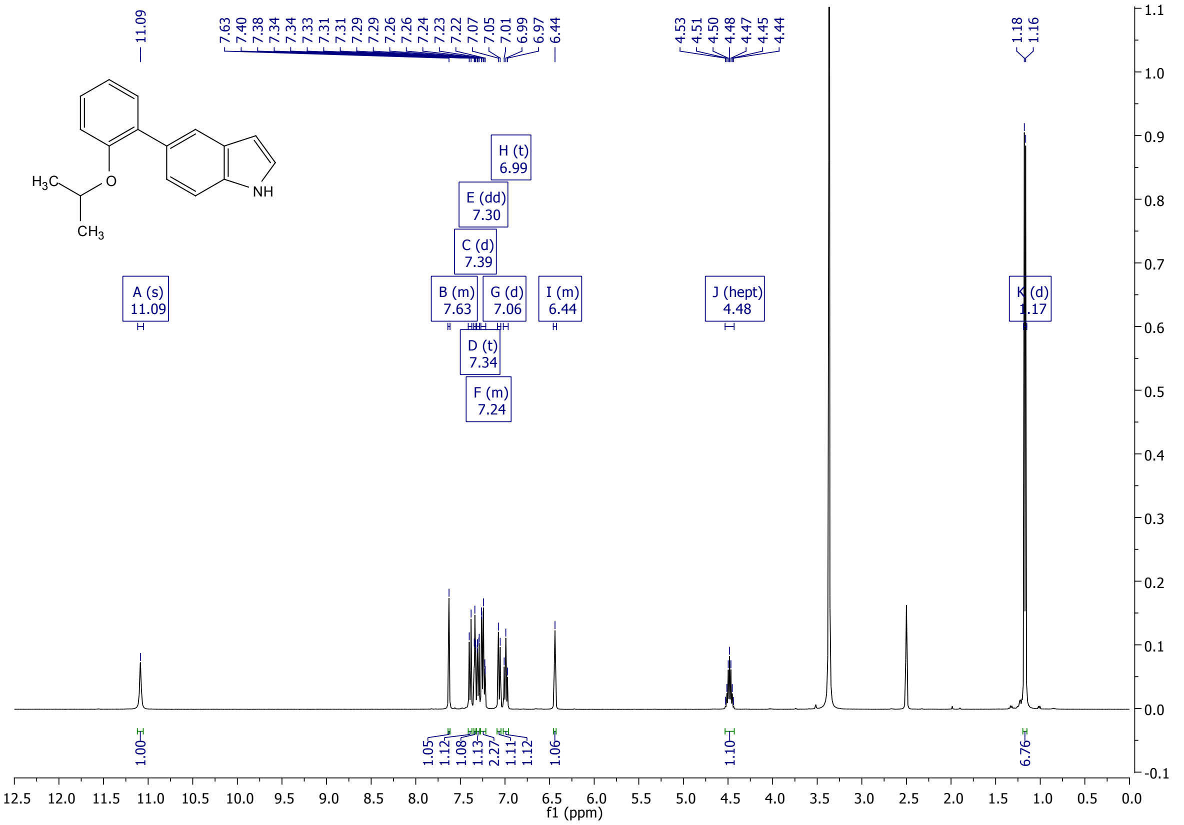

Figure S1.67 ^{1}H NMR spectrum of compound **i-14** in DMSO-d_6.

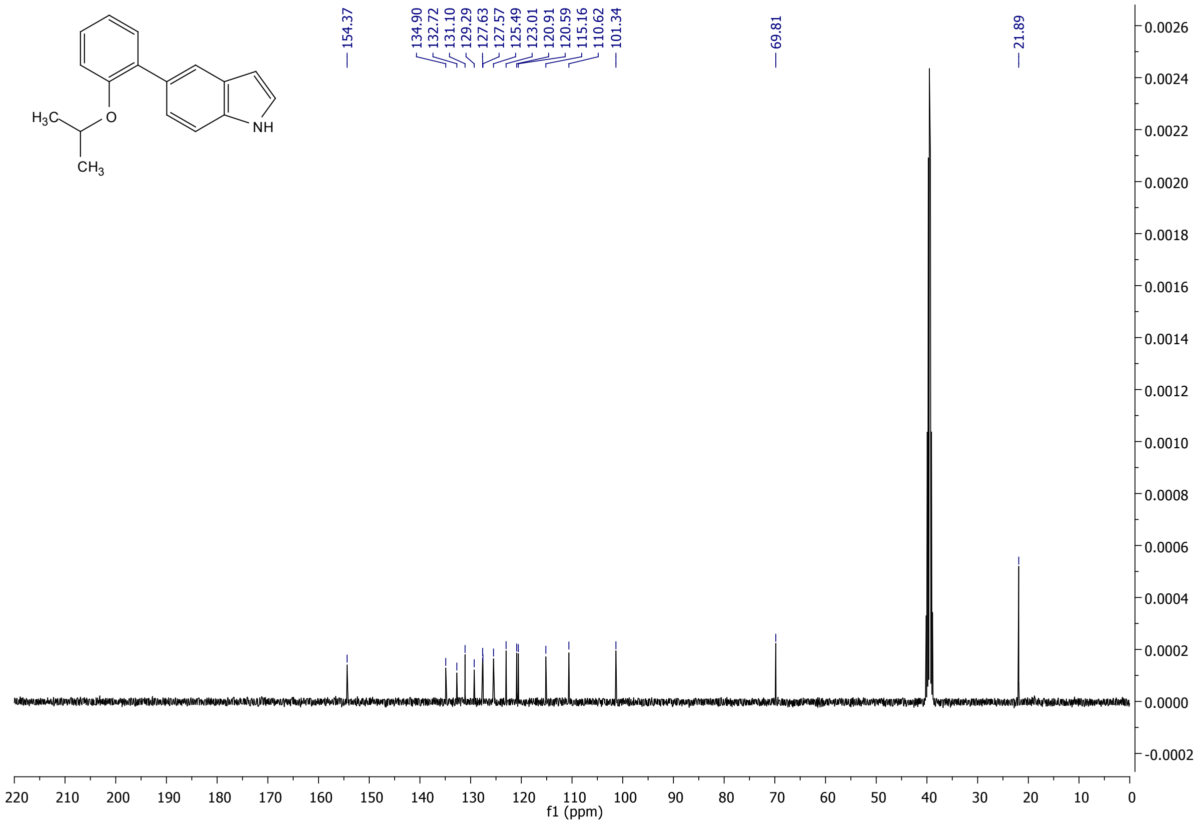

Figure S1.68 ^{13}C NMR spectrum of compound **i-14** in DMSO-d_6.

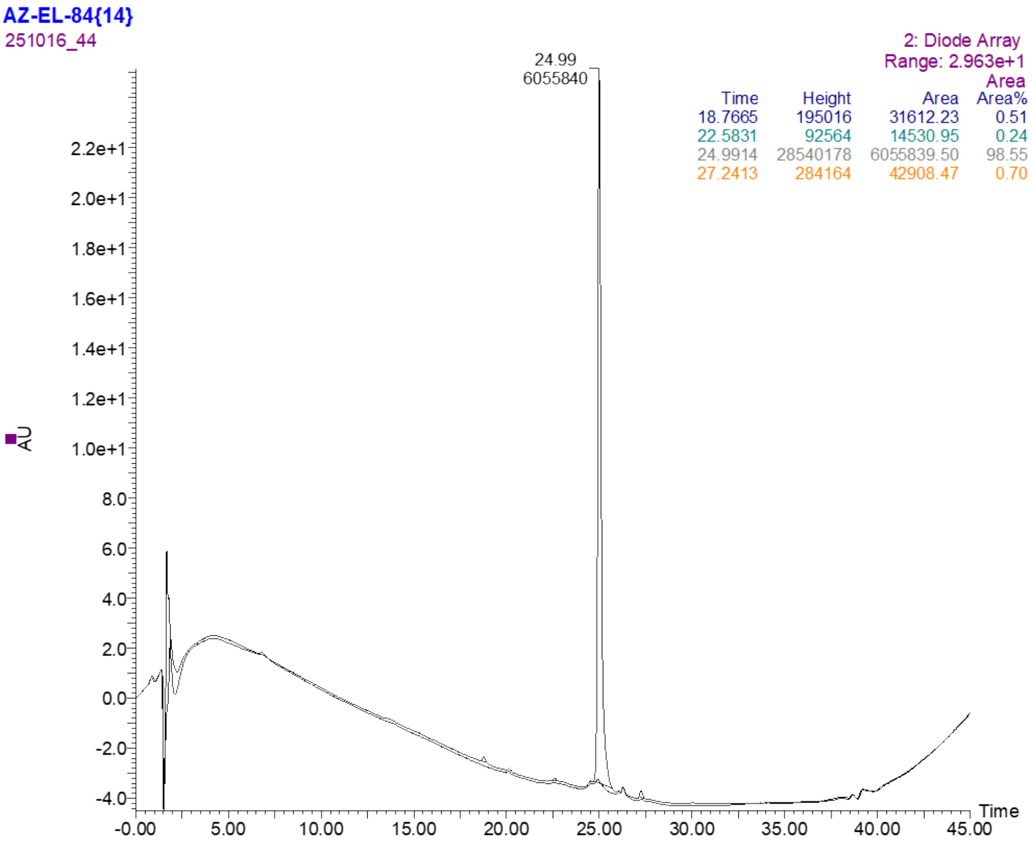

Figure S1.69 LC-MS chromatogram of compound **i-14**.

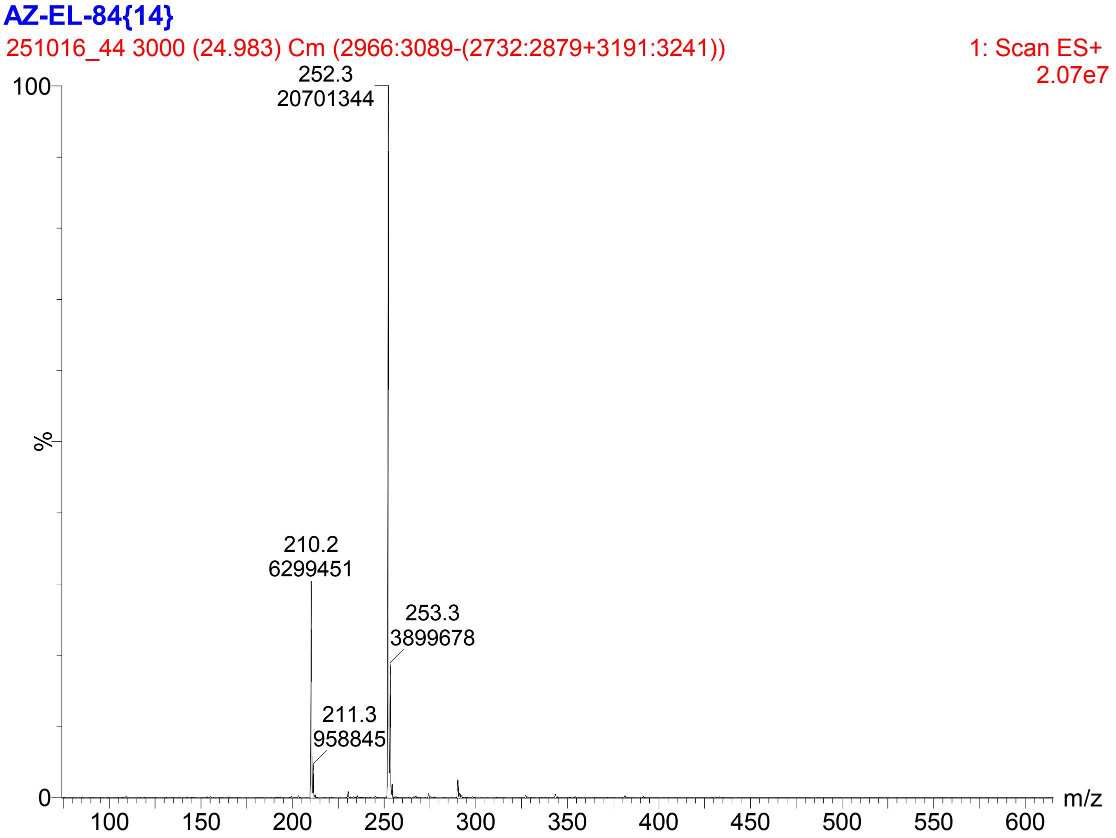

Figure S1.70 MS spectrum of compound **i-14**.

5-(3-Isopropoxyphenyl)-1*H*-indole **i-15**

Prepared in accordance with general procedure **II** from 3-(1*H*-indol-5-yl)phenol **i-2** (35 mg, 0.17 mmol) and 2-iodopropane (25 µL, 0.25 mmol). Yield 62%, brown viscous oil, R_f = 0.28 (*n*-hexane/ethyl acetate, 4/1). IR (neat) ν_{max}, cm^{-1}: 3412 (N-H), 2975, 1596, 1462, 1212, 1110, 966, 867, 768, 726. ^1H NMR (500 MHz, DMSO-d_6): δ 1.30 (6H, d, *J* = 6.0 Hz, 2×CH_3), 4.70 (1H, hept, *J* = 6.0 Hz, CH), 6.46-6.49 (1H, m, CH), 6.83 (1H, dd, *J* = 8.1, 2.4 Hz, CH), 7.13-7.14 (1H, m, CH), 7.17-7.20 (1H, m, CH), 7.31 (1H, t, *J* = 7.9 Hz, CH), 7.35-7.39 (2H, m, 2×CH), 7.45 (1H, d, *J* = 8.5 Hz, CH), 7.79-7.81 (1H, m, CH), 11.14 (1H, br s, NH). ^{13}C NMR (125 MHz, DMSO-d_6): δ 22.0 (2×CH_3), 69.0 (CH), 101.6 (CH), 111.7 (CH), 113.4 (CH), 113.9 (CH), 118.2 (CH), 118.9 (CH), 120.4 (CH), 126.1 (CH), 128.2 (C), 129.8 (CH), 131.3 (C), 135.6 (C), 143.5 (C), 157.9 (C). MS (pos. mode): m/z (%): 252.3 (M+H^+, 100%).

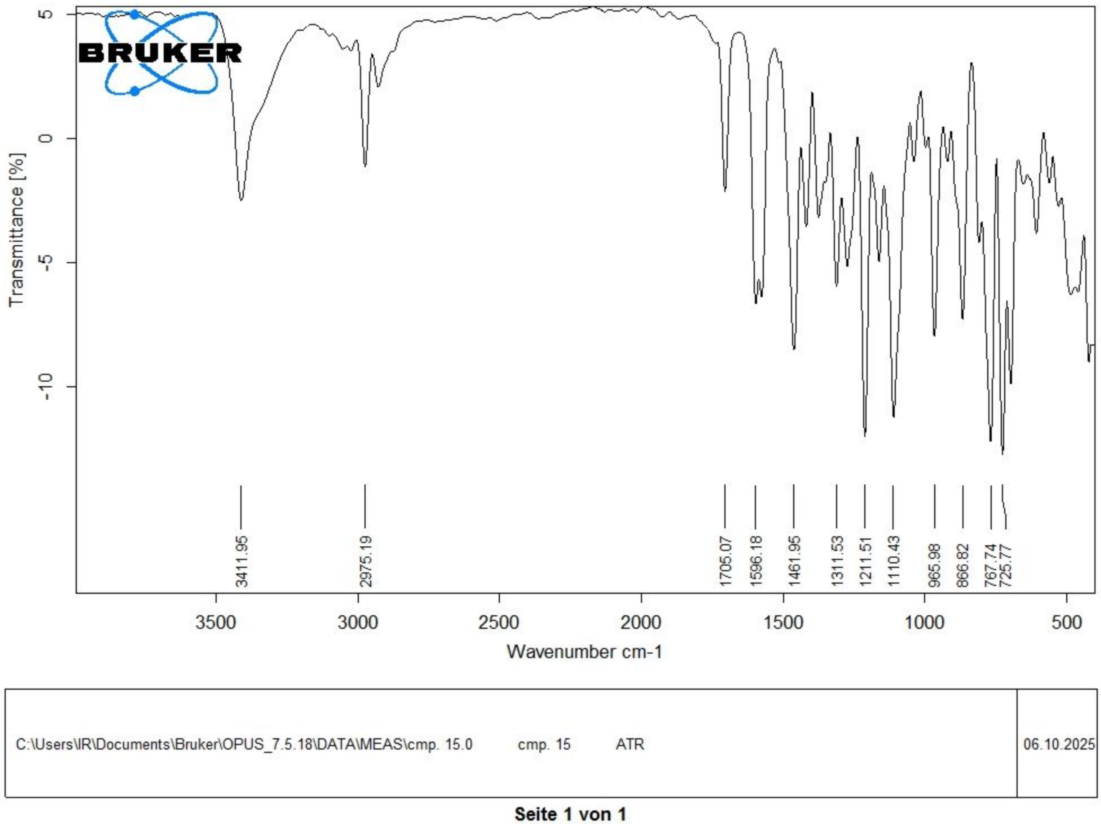

Figure S1.71 FTIR spectrum of compound **i-15**.

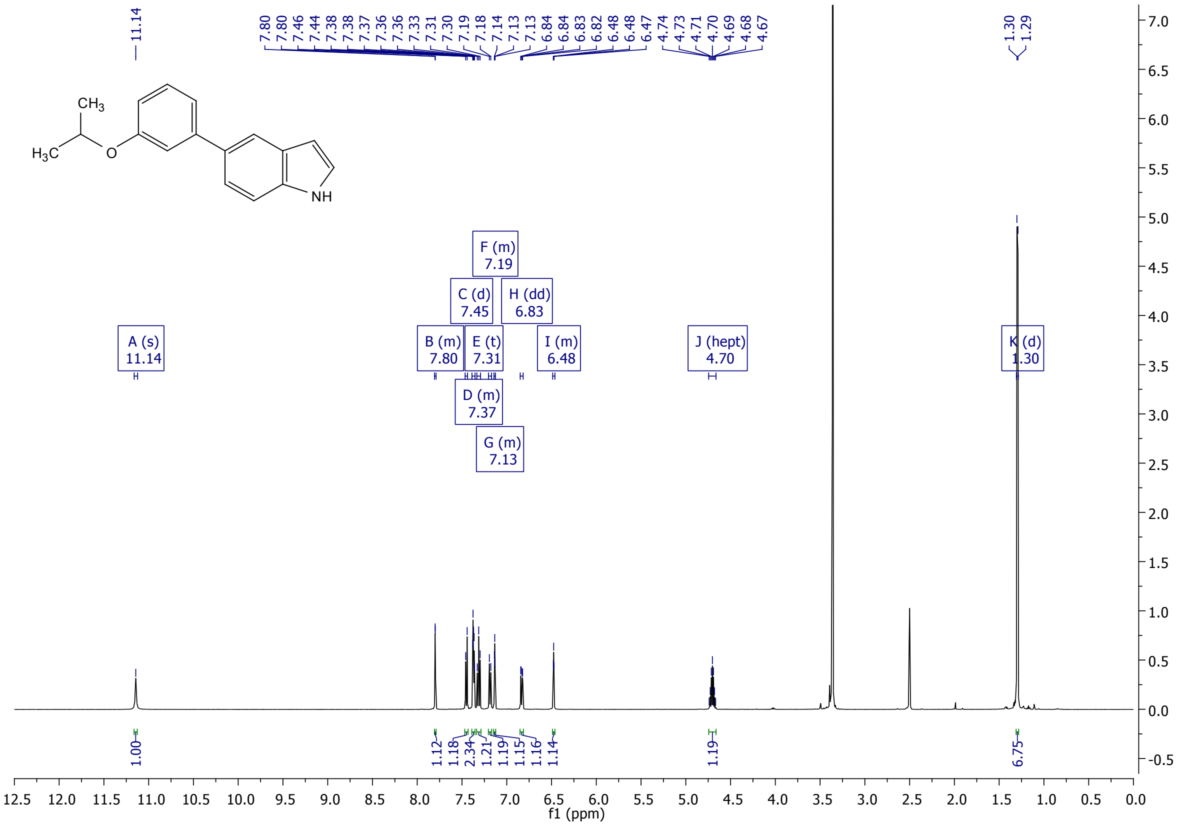

Figure S1.72 ^{1}H NMR spectrum of compound **i-15** in DMSO-d_6.

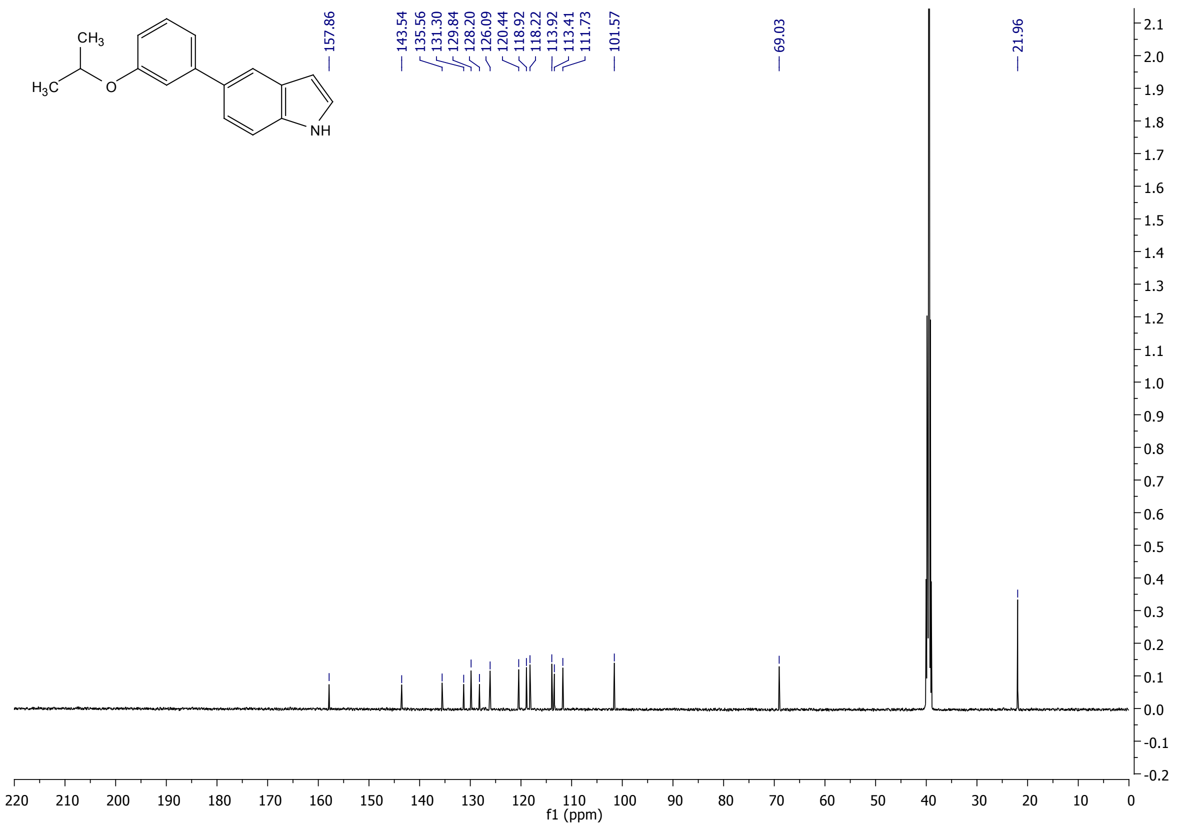

Figure S1.73 ^{13}C NMR spectrum of compound **i-15** in DMSO-d_6.

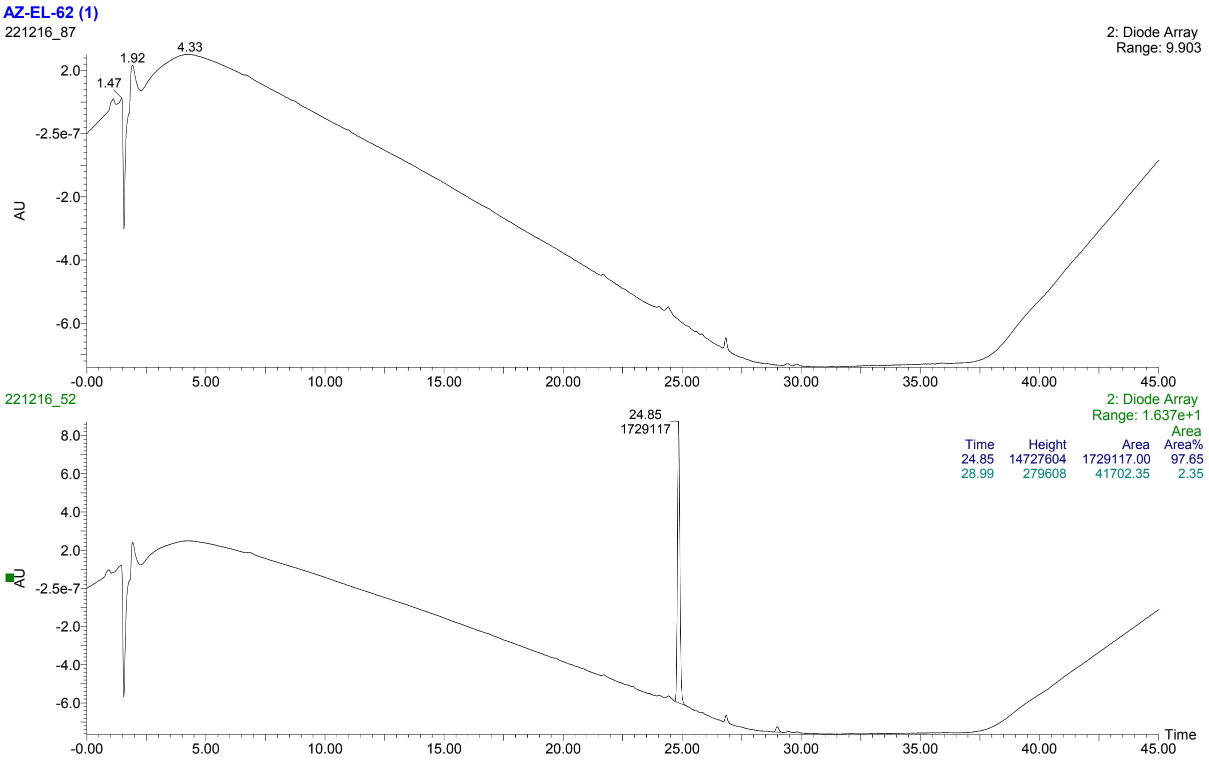

Figure S1.74 LC-MS chromatogram of compound **i-15**.

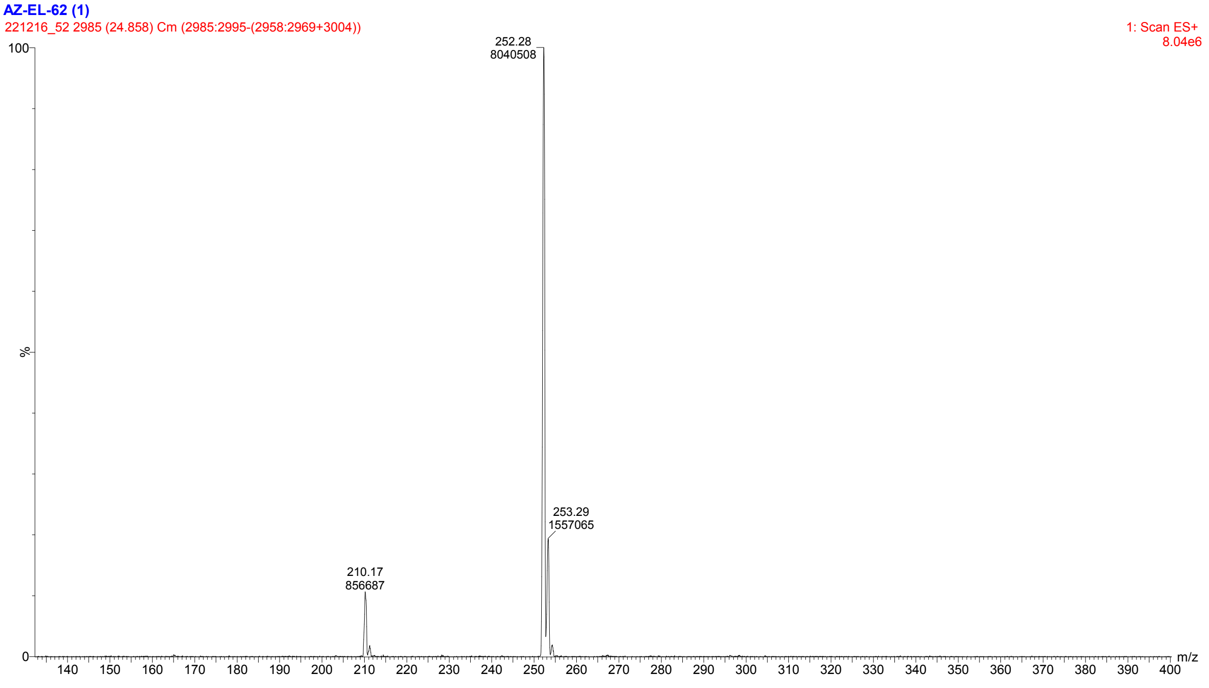

Figure S1.75 MS spectrum of compound **i-15**.

5-(4-Isopropoxyphenyl)-1*H*-indole **i-16**

Prepared in accordance with general procedure **I** from 5-bromo-1*H*-indole (137 mg, 0.70 mmol) and (4-isopropoxyphenyl)boronic acid (189 mg, 1.05 mmol). Yield 86%, pale yellow solid; mp = 72.8–74.7 °C, R_f = 0.26 (*n*-hexane/ethyl acetate, 4/1). IR (neat) ν_{max}, cm^{-1}: 3452 (N-H), 2978, 1509, 1464, 1241, 1098, 950, 803, 724, 467. ^1H NMR (500 MHz, DMSO-d_6): δ 1.27 (6H, d, J = 6.0 Hz, 2×CH_3), 4.55-4.64 (1H, m, CH), 6.44-6.48 (1H, m, CH), 6.95 (2H, d, J = 8.5 Hz, 2×CH), 7.30-7.38 (2H, m, 2×CH), 7.45 (1H, d, J = 8.4 Hz, CH), 7.54 (2H, d, J = 8.5 Hz, 2×CH), 7.71-7.76 (1H, m, CH), 11.11 (1H, br s, NH). ^{13}C NMR (125 MHz, DMSO-d_6): δ 21.9 (2×CH_3), 69.1 (CH), 101.4 (CH), 111.7 (CH), 115.9 (2×CH), 117.5 (CH), 120.1 (CH), 125.9 (CH), 127.7 (2×CH), 128.3 (C), 131.2 (C), 134.2 (C), 135.1 (C), 156.2 (C). MS (pos. mode): m/z (%): 252.2 ($M+H^+$, 100%).

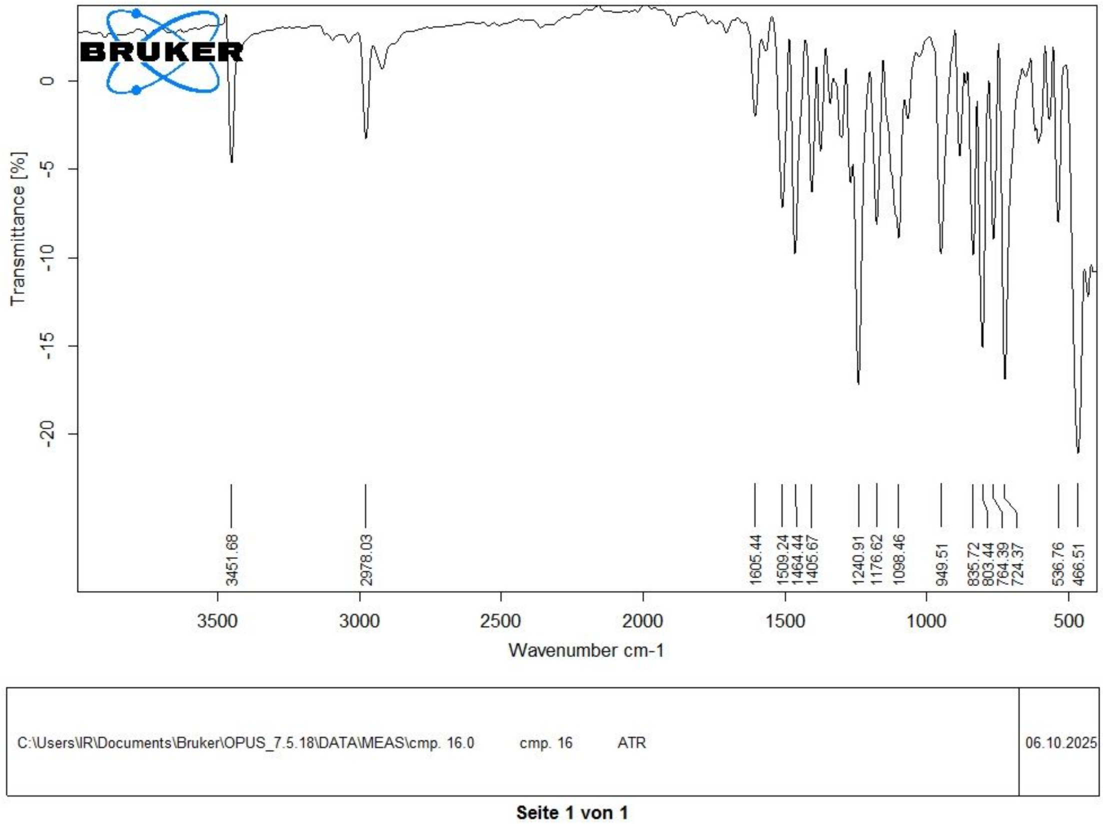

Figure S1.76 FTIR spectrum of compound **i-16**.

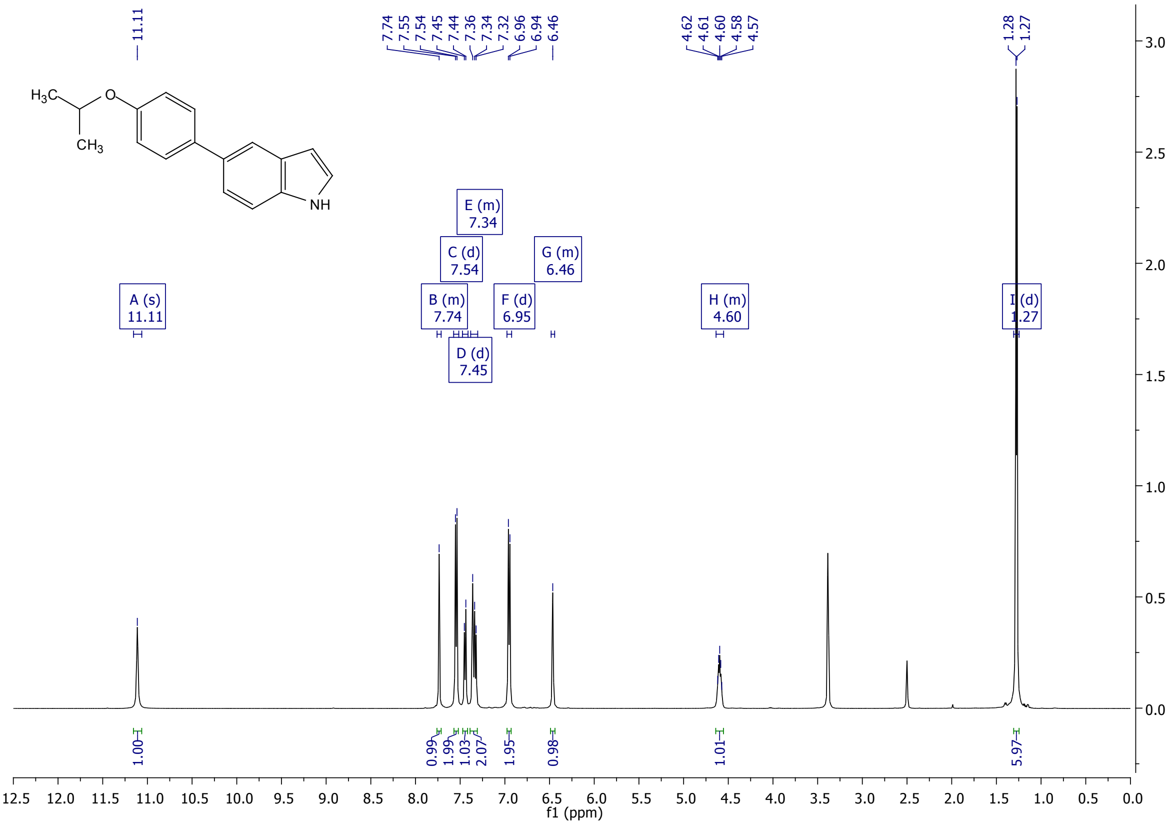

Figure S1.77 ^{1}H NMR spectrum of compound **i-16** in DMSO-d_6.

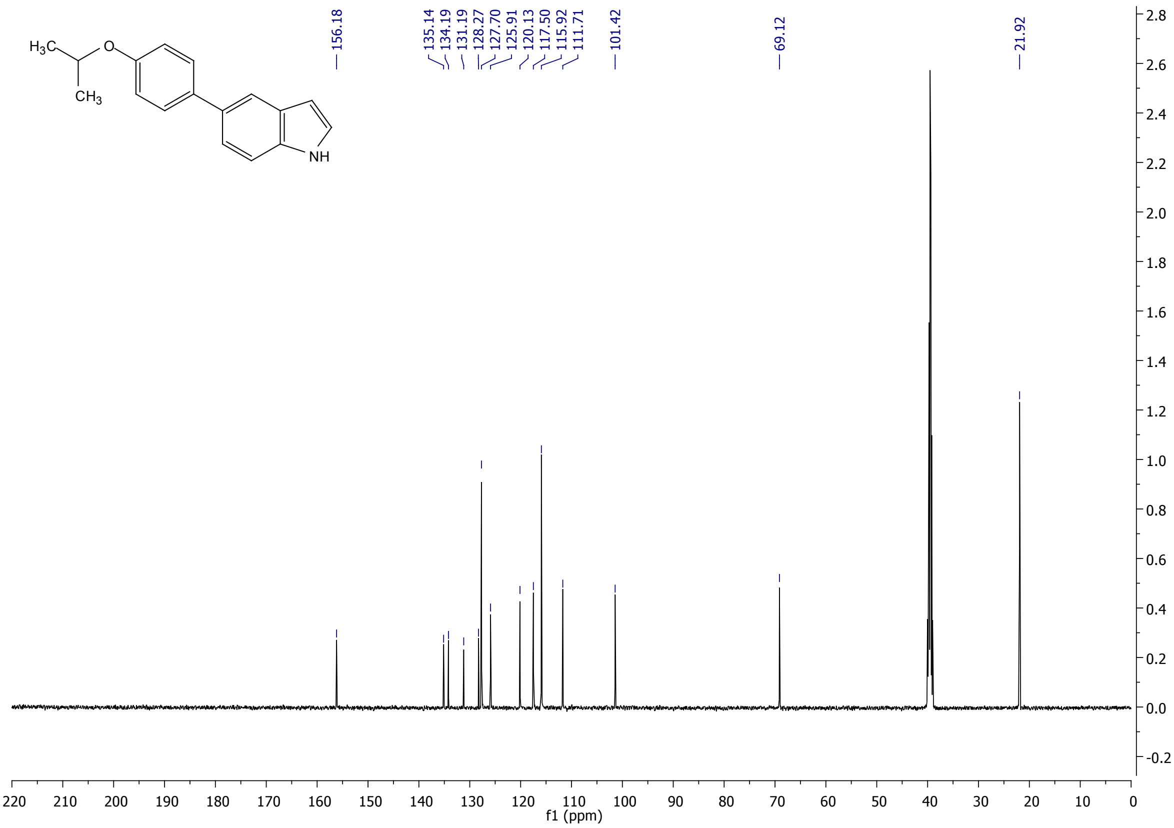

Figure S1.78 ^{13}C NMR spectrum of compound **i-16** in DMSO-d_6.

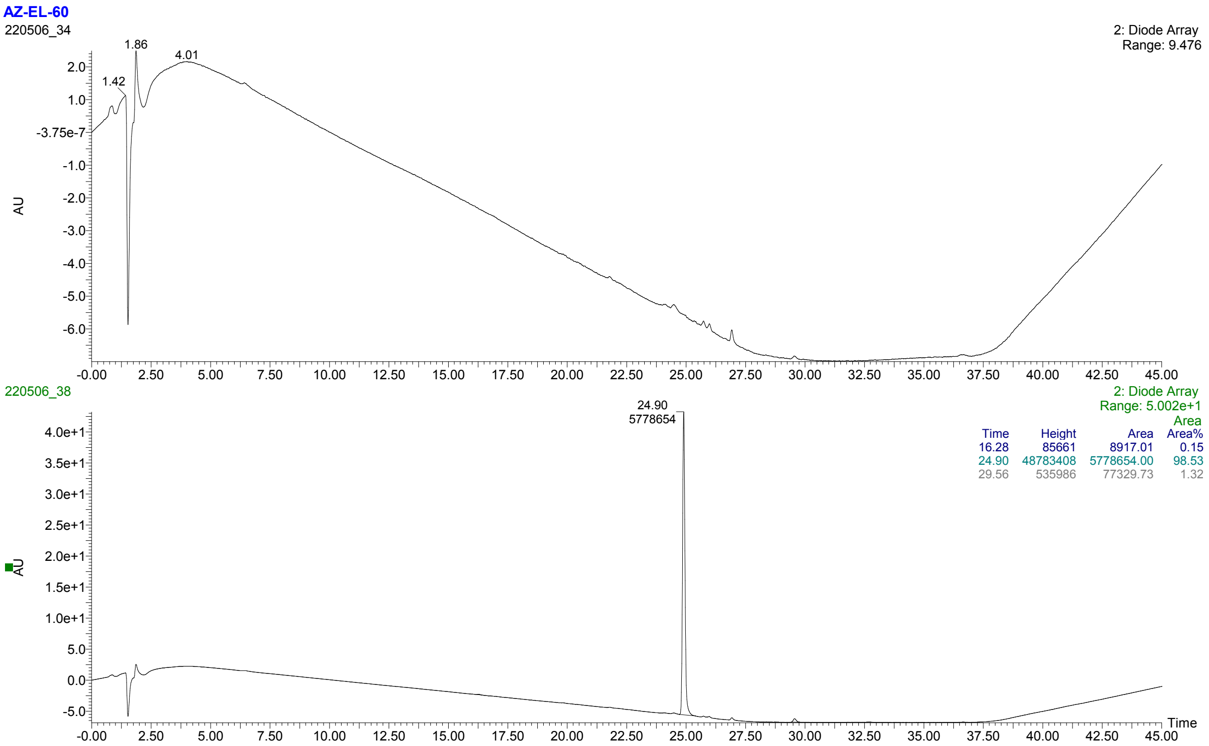

Figure S1.79 LC-MS chromatogram of compound **i-16**.

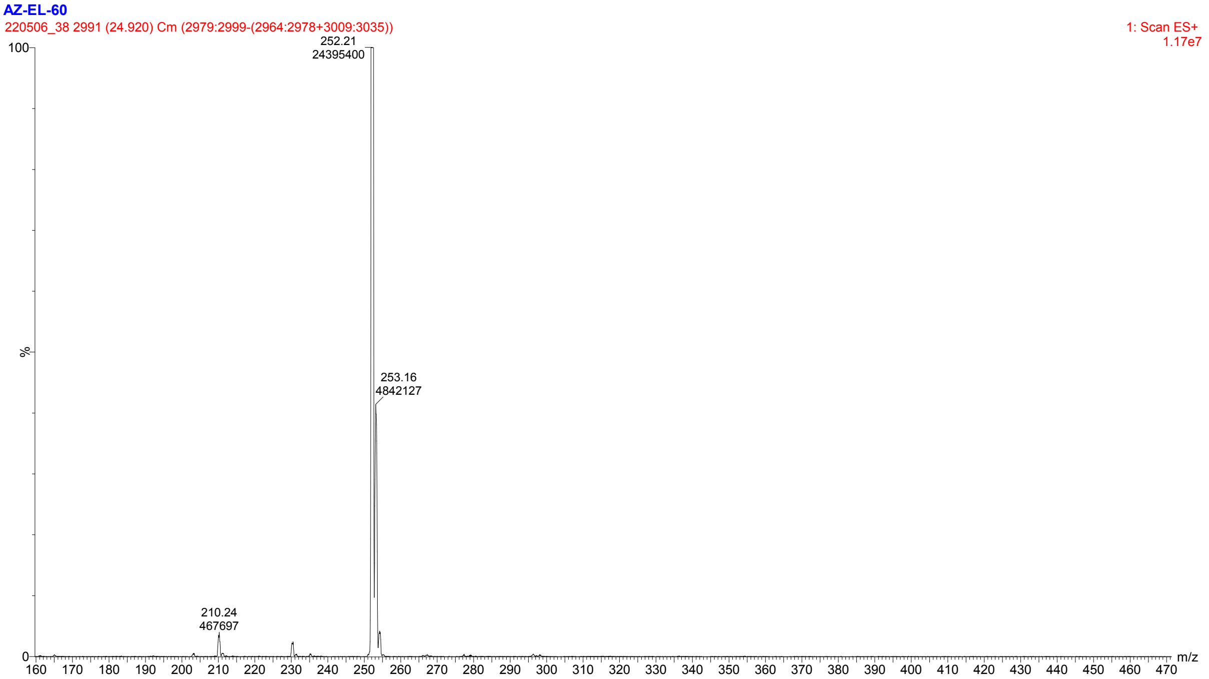

Figure S1.80 MS spectrum of compound **i-16**.

5-(4-Isopropylphenyl)-1*H*-indole **i-17**

Prepared in accordance with general procedure **I** from 5-bromo-1*H*-indole (102 mg, 0.52 mmol) and (4-isopropylphenyl)boronic acid (128 mg, 0.78 mmol). Yield 88%, white solid; mp = 109.2–110.6 °C, R_f = 0.32 (*n*-hexane/ethyl acetate, 4/1). IR (neat) ν_{max}, cm^{-1}: 3443 (N-H), 2956, 1464, 1407, 809, 723, 553, 483. ^{1}H NMR (500 MHz, DMSO-d_6): δ 1.24 (6H, d, J = 6.9 Hz, 2×CH_3), 2.91 (1H, hept, J = 6.8 Hz, CH), 6.45-6.49 (1H, m, CH), 7.29 (2H, d, J = 8.1 Hz, 2×CH), 7.34-7.39 (2H, m, 2×CH), 7.45 (1H, d, J = 8.4 Hz, CH), 7.57 (2H, d, J = 8.1 Hz, 2×CH), 7.77 (1H, s, CH), 11.13 (1H, br s, NH). ^{13}C NMR (125 MHz, DMSO-d_6): δ 24.0 (2×CH_3), 30.1 (CH), 101.5 (CH), 111.8 (CH), 117.9 (CH), 120.3 (CH), 126.0 (CH), 126.6 (2×CH), 126.7 (2×CH), 128.3 (C), 131.4 (C), 135.4 (C), 139.5 (C), 146.3 (C). MS (pos. mode): m/z (%): 236.4 (M+H$^+$, 100%).

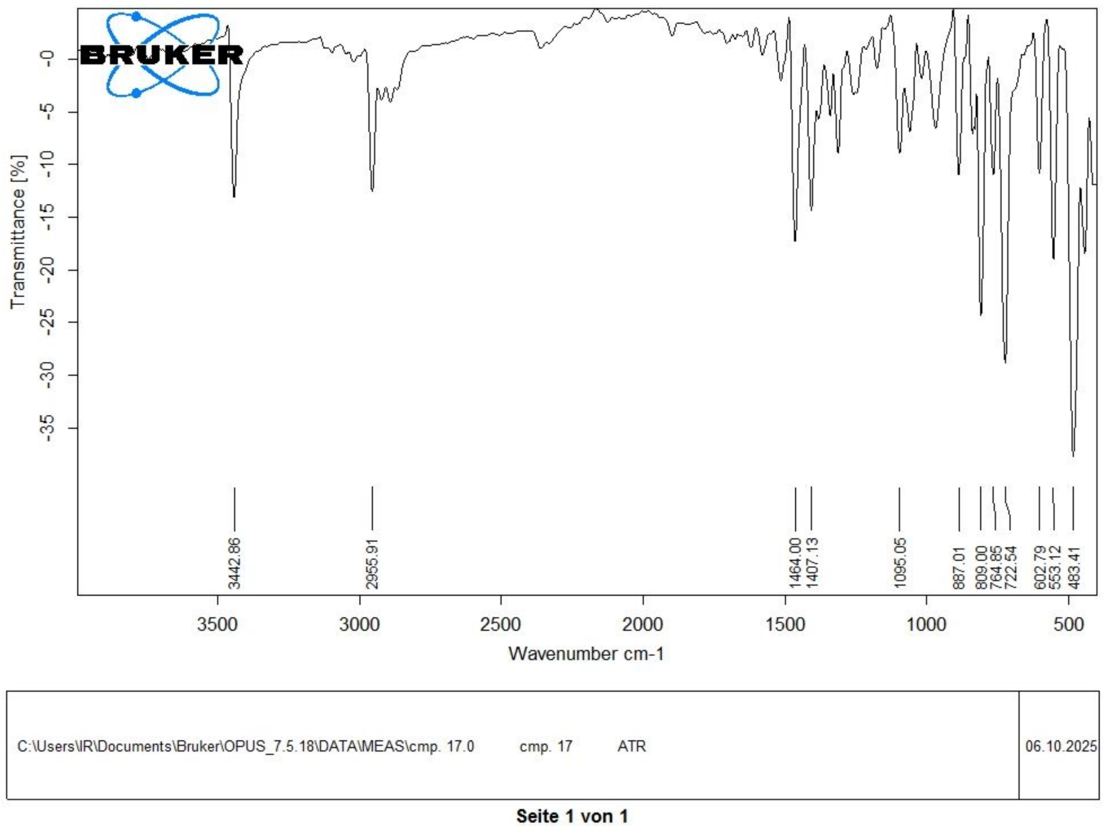

Figure S1.81 FTIR spectrum of compound **i-17**.

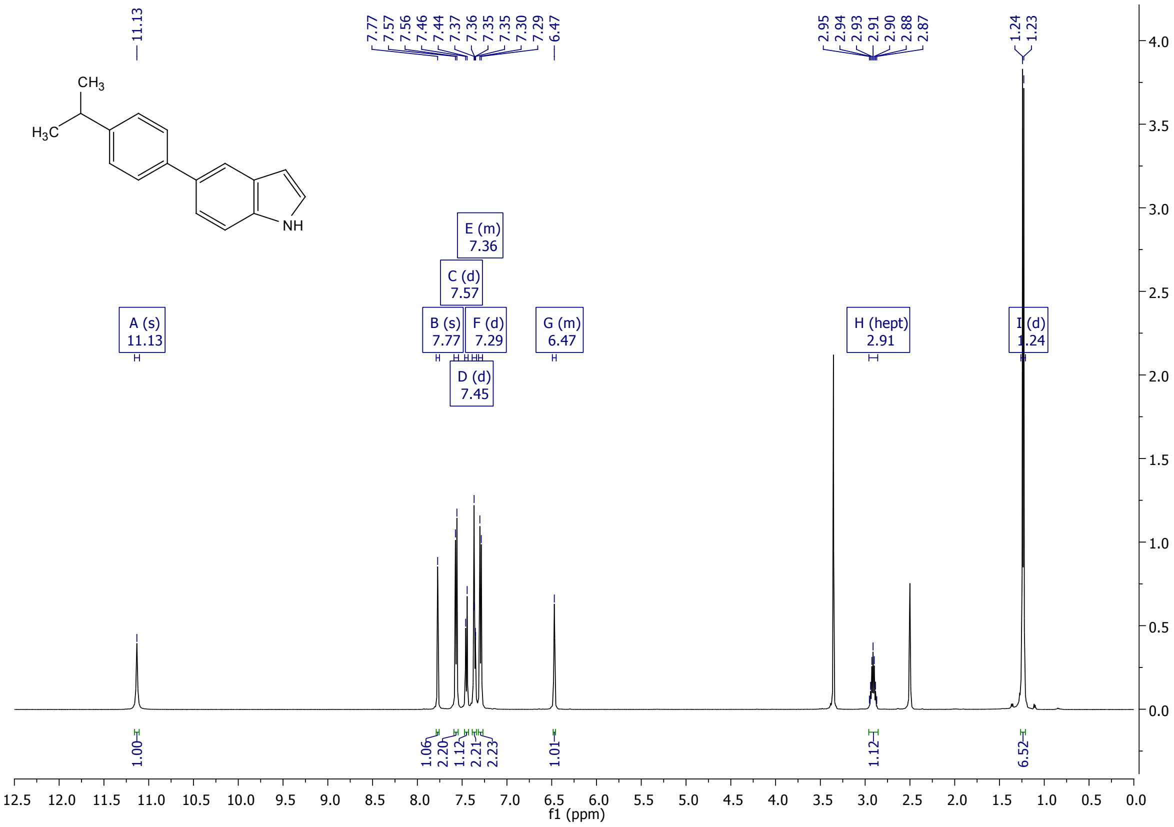

Figure S1.82 ^{1}H NMR spectrum of compound **i-17** in DMSO-d_6.

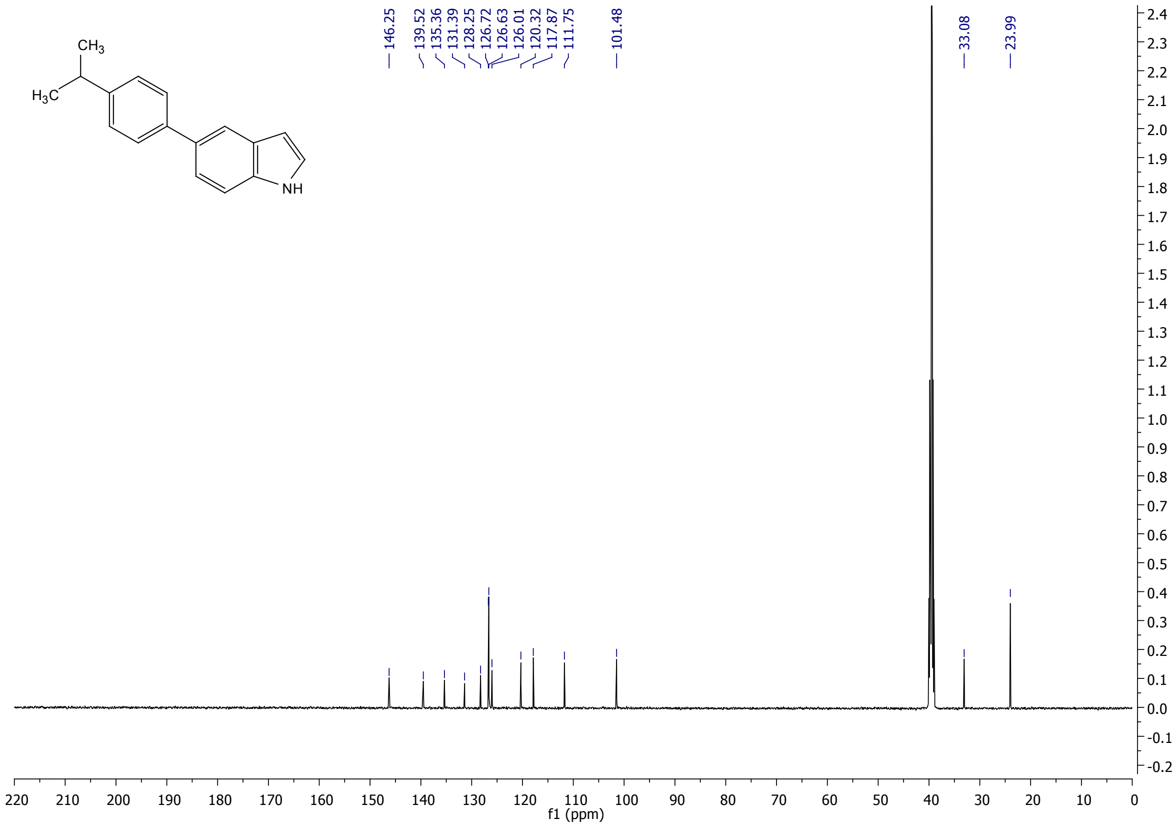

Figure S1.83 ^{13}C NMR spectrum of compound **i-17** in DMSO-d_6.

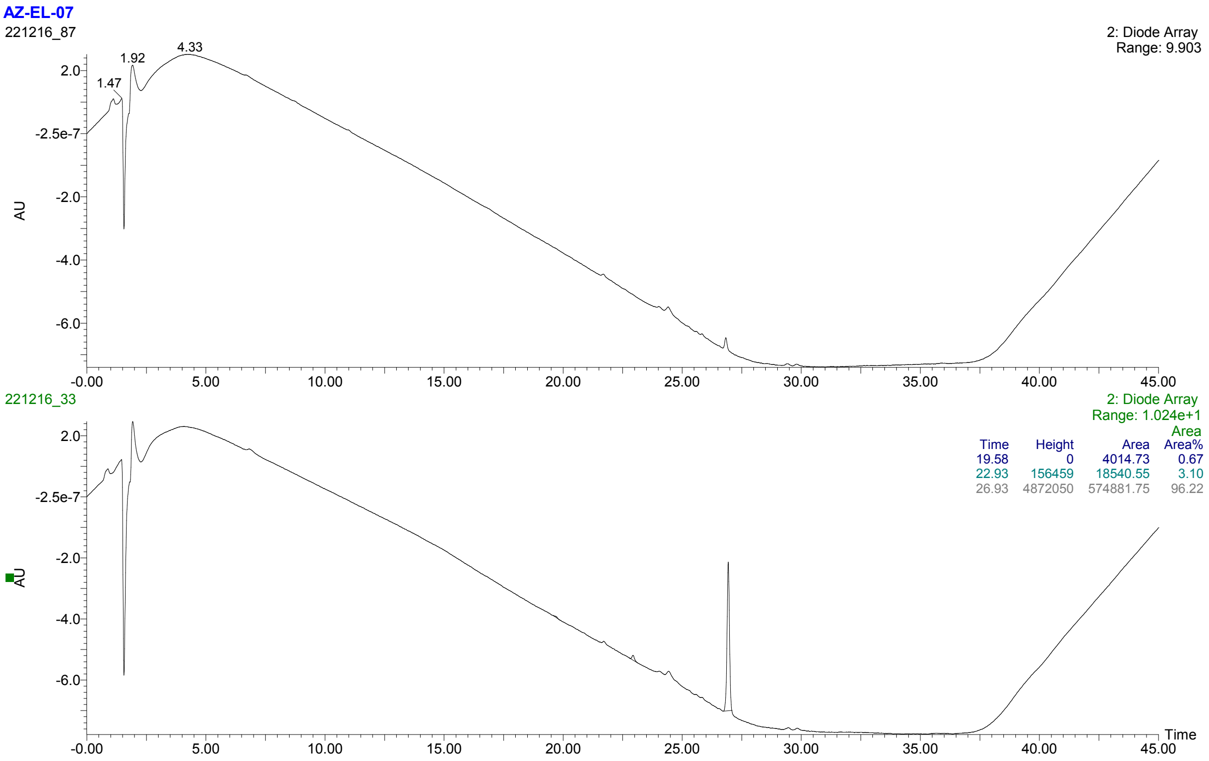

Figure S1.84 LC-MS chromatogram of compound **i-17**.

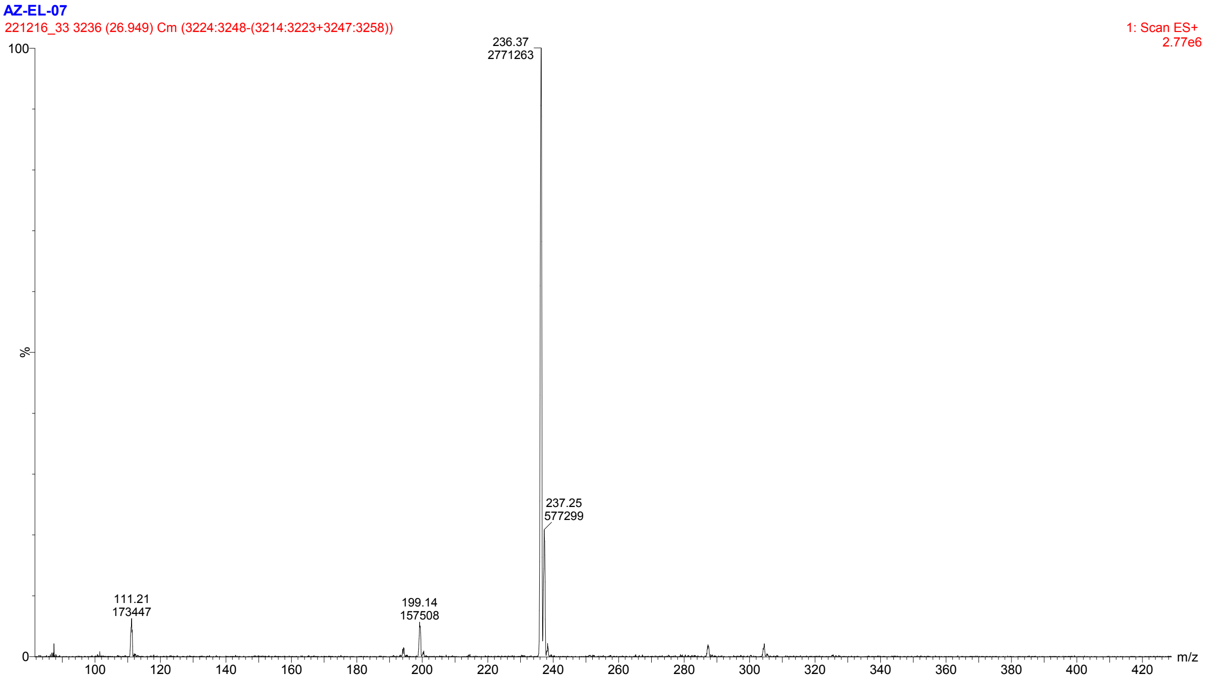

Figure S1.85 MS spectrum of compound **i-17**.

5-(3-Chlorophenyl)-1*H*-indole **i-18**

Previously reported in [5]. Prepared in accordance with general procedure **I** from 5-bromo-1*H*-indole (50 mg, 0.26 mmol) and (3-chlorophenyl)boronic acid (60 mg, 0.38 mmol). Yield 86%, white solid; mp = 62.6–62.8 °C, R_f = 0.26 (*n*-hexane/ethyl acetate, 4/1). IR (neat) ν_{max}, cm^{-1}: 3388 (N-H), 1455, 766, 728, 684, 490, 418. ^{1}H NMR (500 MHz, DMSO-d_6): δ 6.48-6.51 (1H, m, CH), 7.34 (1H, ddd, *J* = 7.9, 2.0, 0.9 Hz, CH), 7.39-7.42 (2H, m, 2×CH), 7.44-7.50 (2H, m, 2×CH), 7.62-7.65 (1H, m, CH), 7.68-7.70 (1H, m, CH), 7.85-7.87 (1H, m, CH), 11.20 (1H, br s, NH). ^{13}C NMR (125 MHz, DMSO-d_6): δ 101.7 (CH), 111.9 (CH), 118.5 (CH), 120.3 (CH), 125.3 (CH), 125.9 (CH), 126.28 (CH), 126.33 (CH), 128.2 (C), 129.7 (C), 130.6 (CH), 133.6 (C), 135.8 (C), 144.1 (C). MS (neg. mode): m/z (%): 226.2/228.2 (M-H$^-$, 100%).

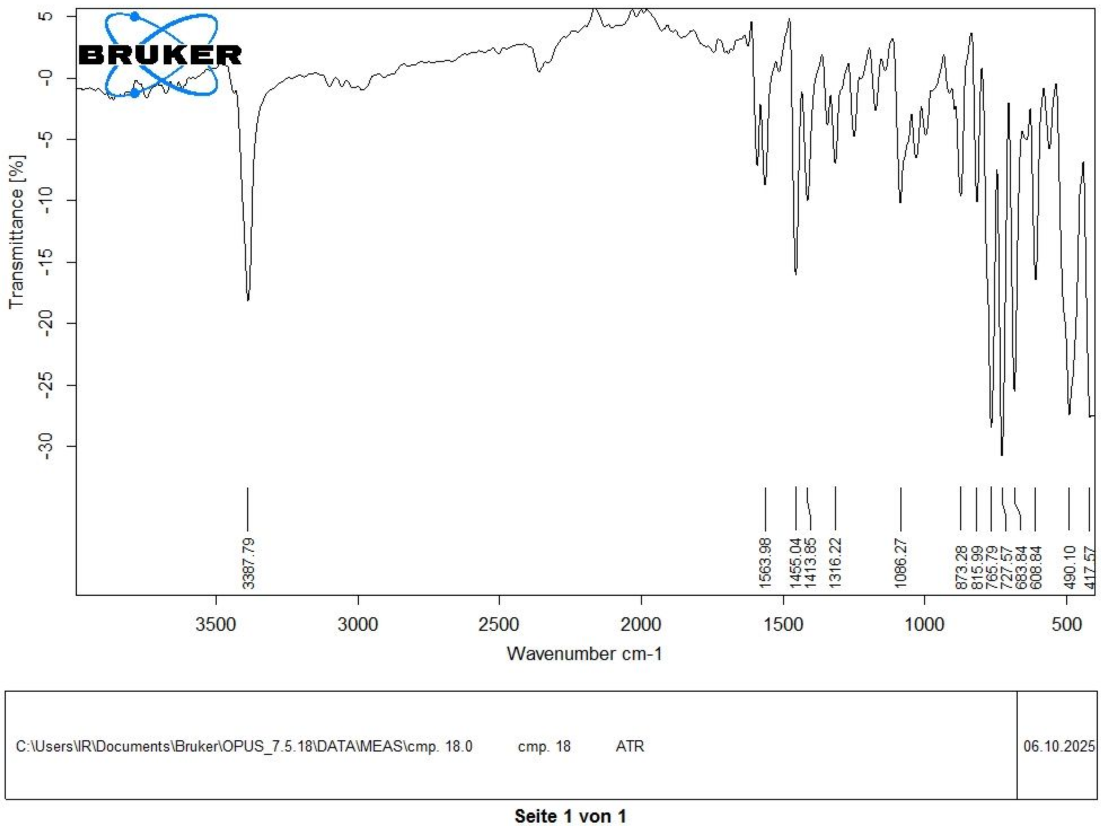

Figure S1.86 FTIR spectrum of compound **i-18**.

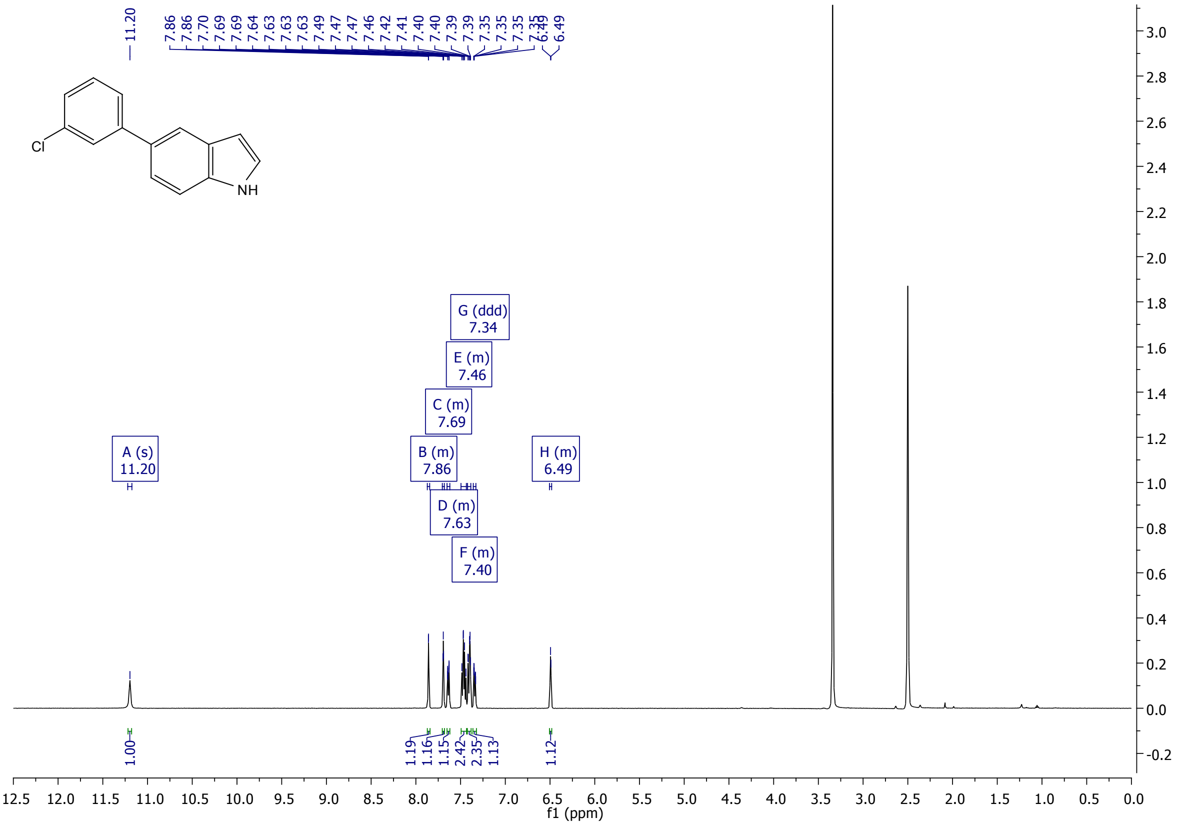

Figure S1.87 ^{1}H NMR spectrum of compound **i-18** in DMSO-d_6.

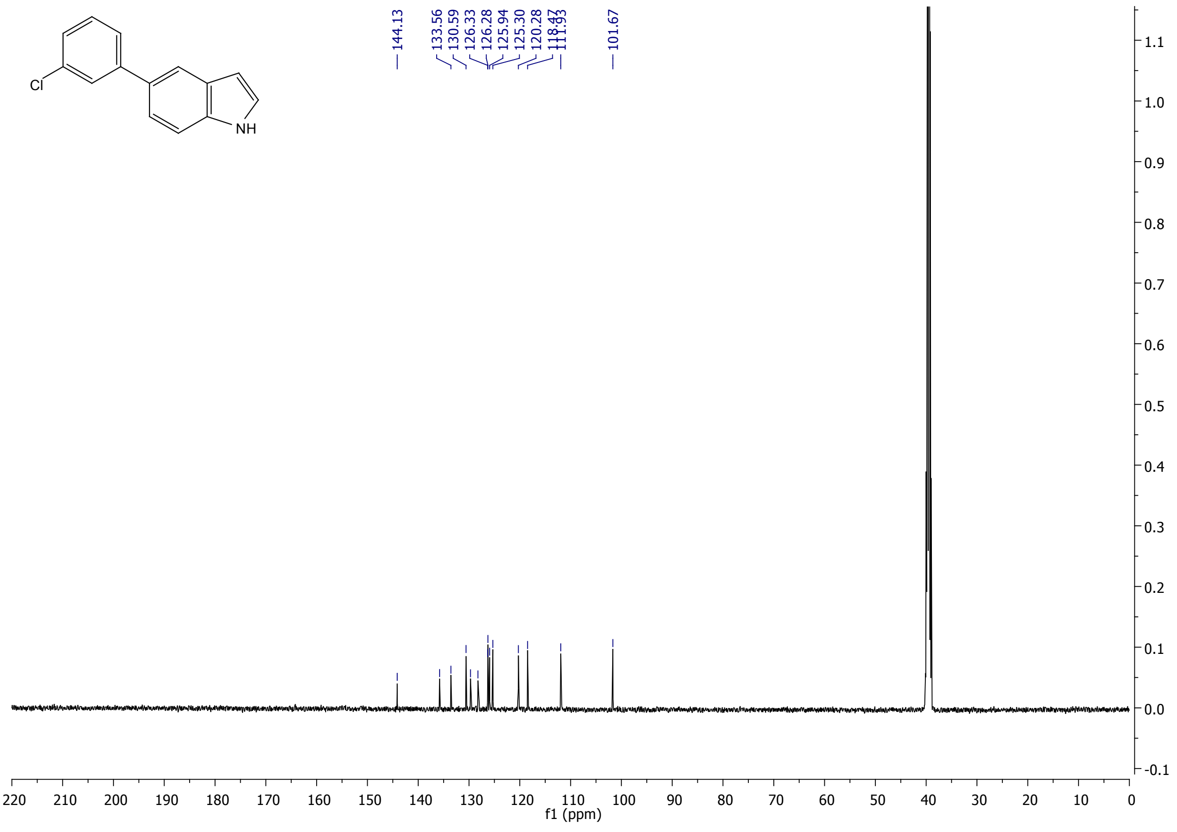

Figure S1.88 ^{13}C NMR spectrum of compound **i-18** in DMSO-d_6.

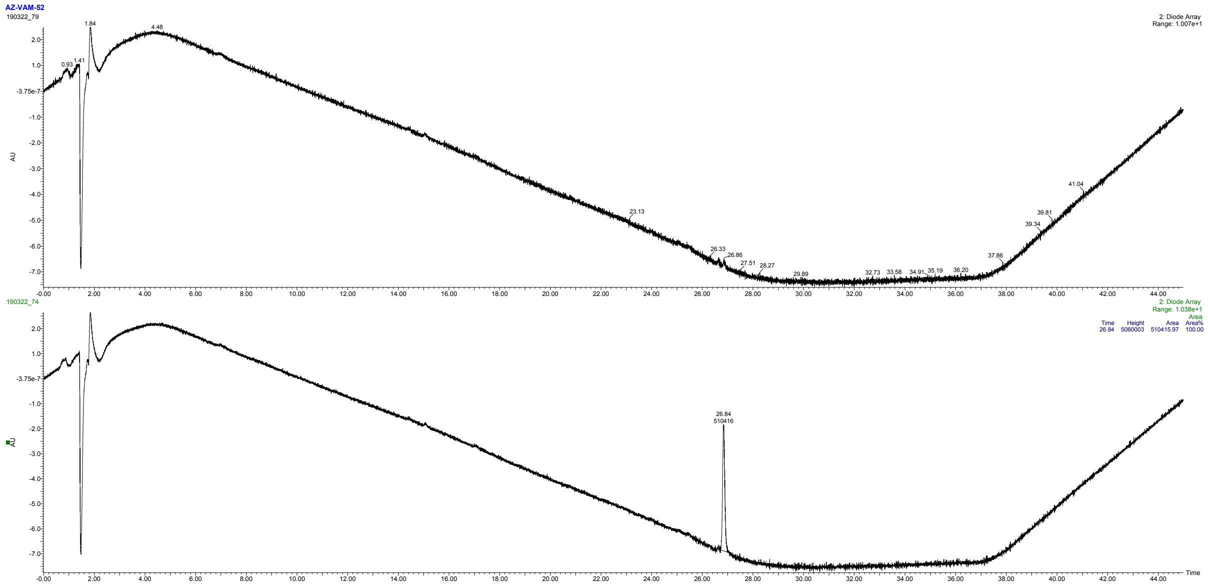

Figure S1.89 LC-MS chromatogram of compound **i-18**.

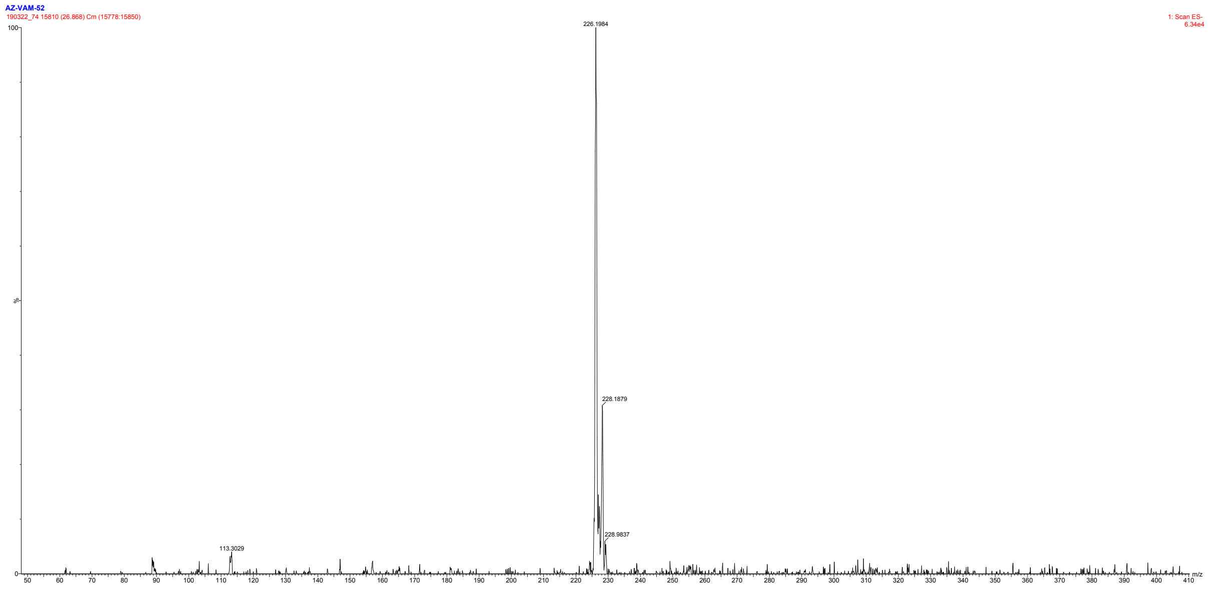

Figure S1.90 MS spectrum of compound **i-18**.

5-(4-Chlorophenyl)-1*H*-indole **i-19**

Previously reported in [5]. Prepared in accordance with general procedure **I** from 5-bromo-1*H*-indole (50 mg, 0.26 mmol) and (4-chlorophenyl)boronic acid (60 mg, 0.38 mmol). Yield 92%, white solid; mp = 91.7–92.0 °C, R_f = 0.25 (*n*-hexane/ethyl acetate, 4/1). IR (neat) ν_{max}, cm^{-1}: 3457 (N-H), 1457, 1086, 799, 732, 457, 421. ^{1}H NMR (500 MHz, DMSO-d_6): δ 6.47-6.50 (1H, m, CH), 7.36-7.41 (2H, m, 2×CH), 7.45-7.50 (3H, m, 3×CH), 7.68 (2H, d, *J* = 8.5 Hz, 2×CH), 7.82 (1H, s, CH), 11.18 (1H, br s, NH). ^{13}C NMR (125 MHz, DMSO-d_6): δ 101.6 (CH), 111.9 (CH), 118.2 (CH), 120.2 (CH), 126.3 (CH), 128.25 (CH), 128.34 (2×CH), 128.7 (2×CH), 130.0 (C), 131.0 (C), 135.6 (C), 140.8 (C). MS (pos. mode): m/z (%): 226.1/228.2 (M-H$^-$, 100%).

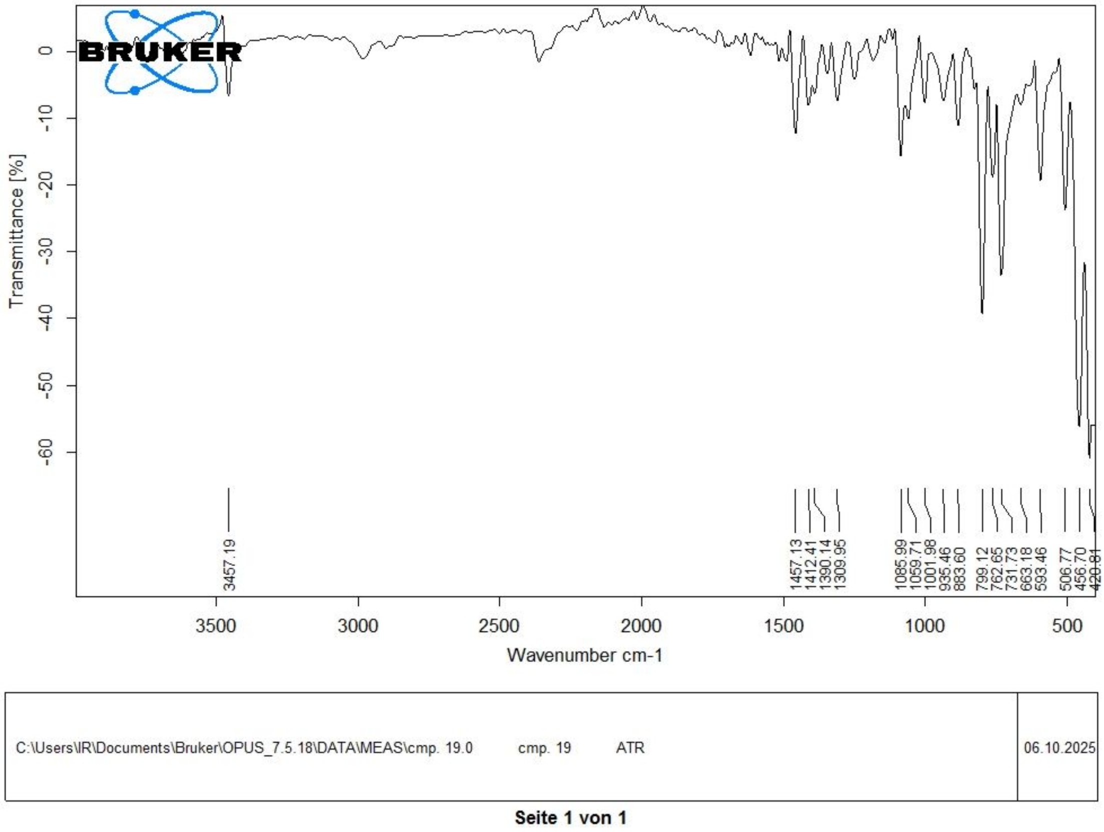

Figure S1.91 FTIR spectrum of compound **i-19**.

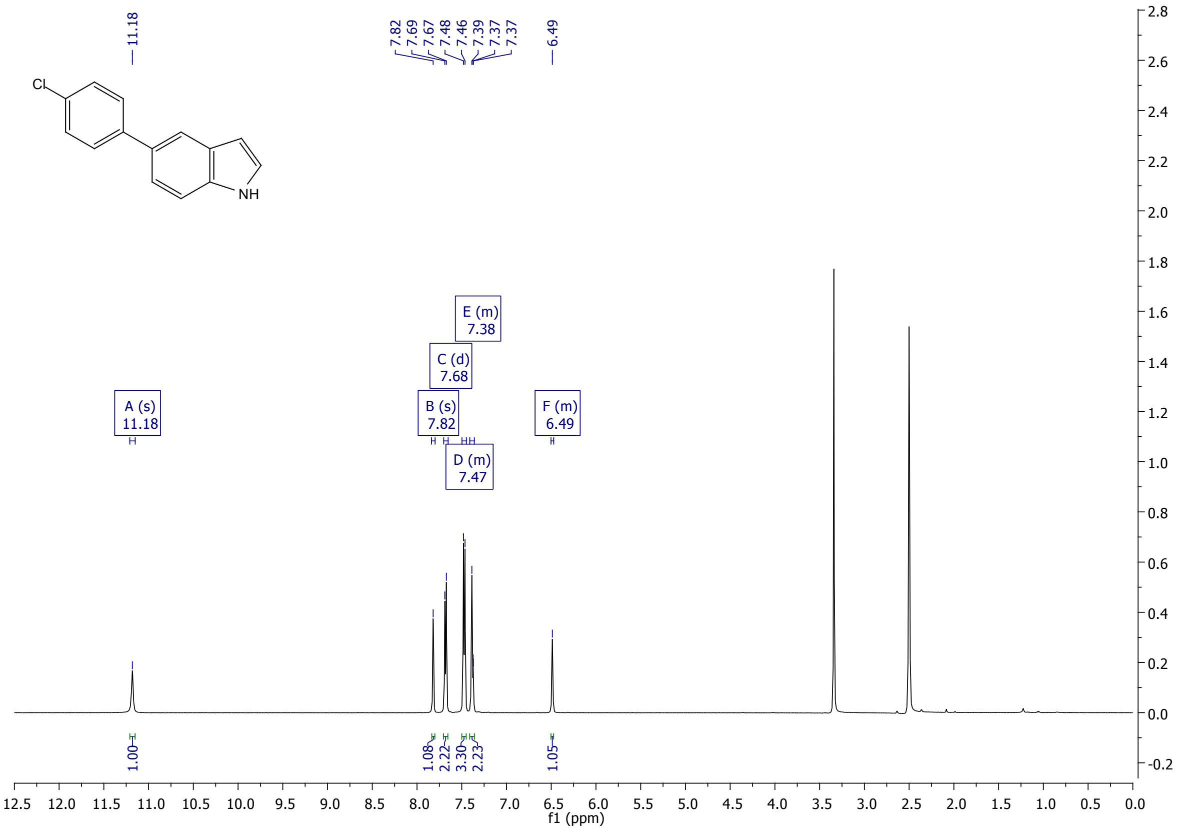

Figure S1.92 ^{1}H NMR spectrum of compound **i-19** in DMSO-d_6.

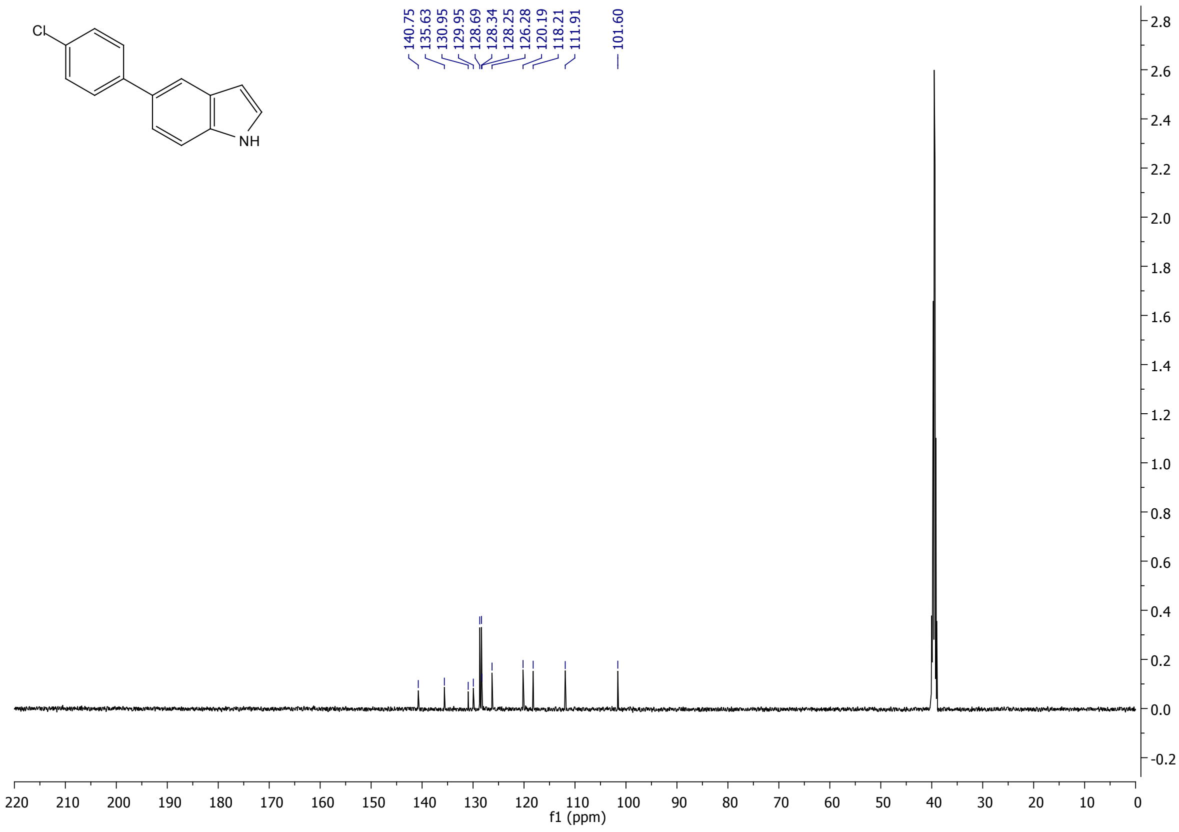

Figure S1.93 ^{13}C NMR spectrum of compound **i-19** in DMSO-d_6.

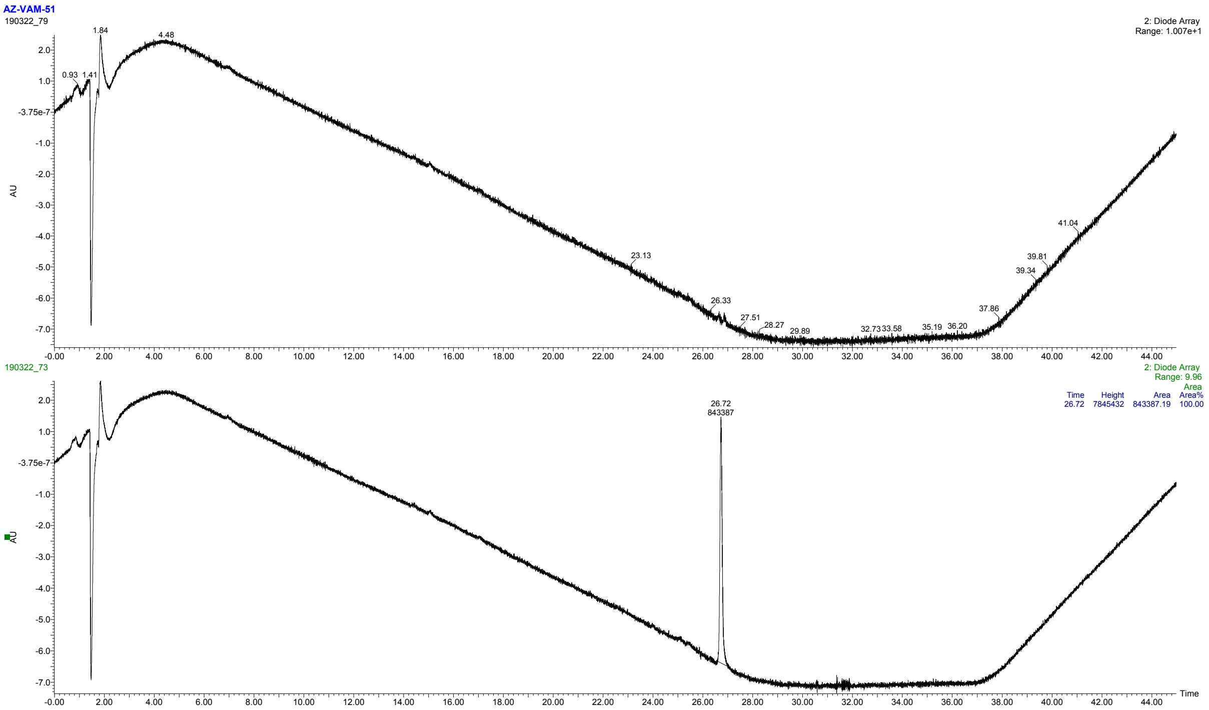

Figure S1.94 LC-MS chromatogram of compound **i-19**.

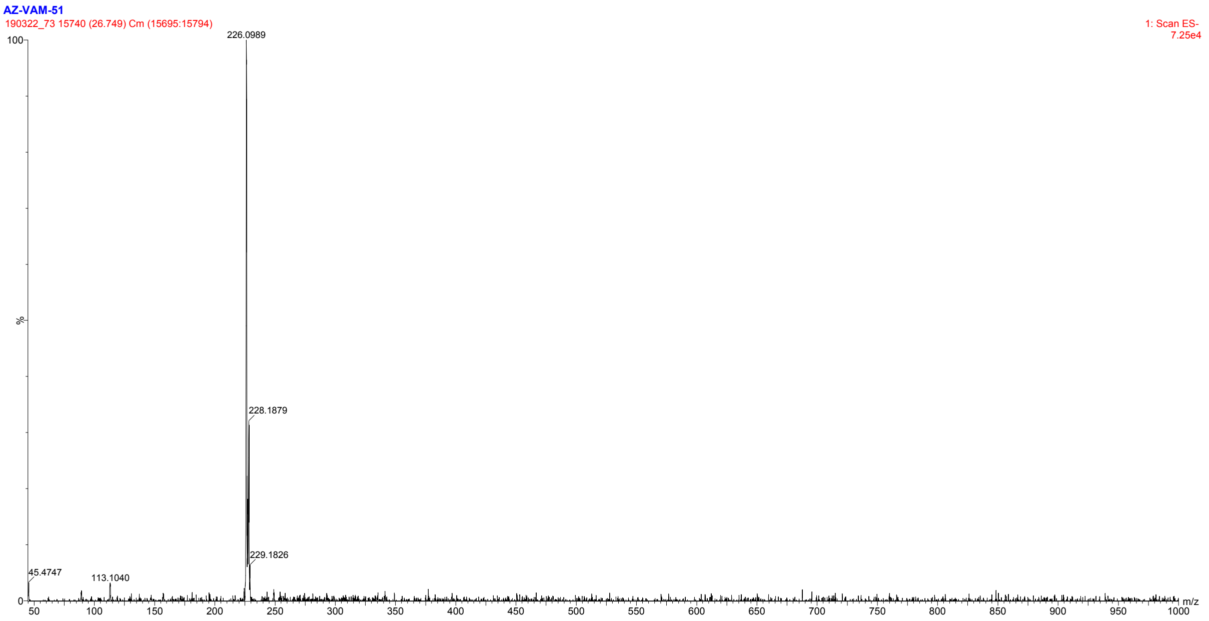

Figure S1.95 MS spectrum of compound **i-19**.

5-(4-Fluorophenyl)-1*H*-indole **i-20**

Previously reported in [5]. Prepared in accordance with general procedure **I** from 5-bromo-1*H*-indole (137 mg, 0.70 mmol) and (4-fluorophenyl)boronic acid (147 mg, 1.05 mmol). Yield 75%, pale yellow solid; mp = 87.4–88.3 °C, R_f = 0.26 (*n*-hexane/ethyl acetate, 4/1). IR (neat) ν_{max}, cm^{-1}: 3417 (N-H), 1507, 1465, 1212, 838, 807, 766, 738, 511. ^{1}H NMR (500 MHz, DMSO-d_6): δ 6.47-6.49 (1H, m, CH), 7.22-7.27 (2H, m, 2×CH), 7.34-7.40 (2H, m, 2×CH), 7.47 (1H, d, J = 8.4 Hz, CH), 7.65-7.70 (2H, m, 2×CH), 7.78 (1H, s, CH), 11.17 (1H, br s, NH). ^{13}C NMR (125 MHz, DMSO-d_6): δ 101.5 (CH), 111.8 (CH), 115.5 (d, $^2J_{C,F}$ = 21.3 Hz, 2×CH), 118.1 (CH), 120.3 (CH), 126.2 (CH), 128.3 (C), 128.5 (d, $^3J_{C,F}$ = 8.0 Hz, 2×CH), 130.4 (C), 135.4 (C), 138.4 (d, $^4J_{C,F}$ = 2.2 Hz, C), 161.2 (d, $^1J_{C,F}$ = 242.8 Hz, C). MS (pos. mode): m/z (%): 212.2 (M+H$^+$, 100%).

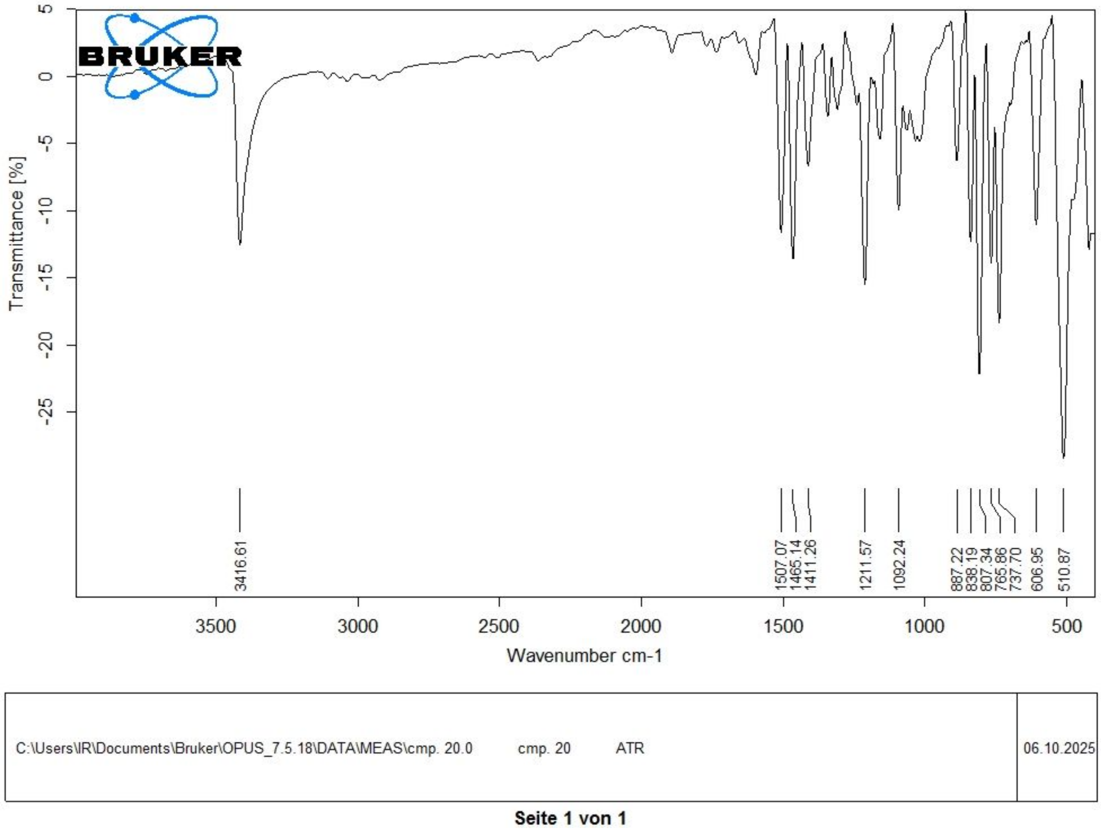

Figure S1.96 FTIR spectrum of compound **i-20**.

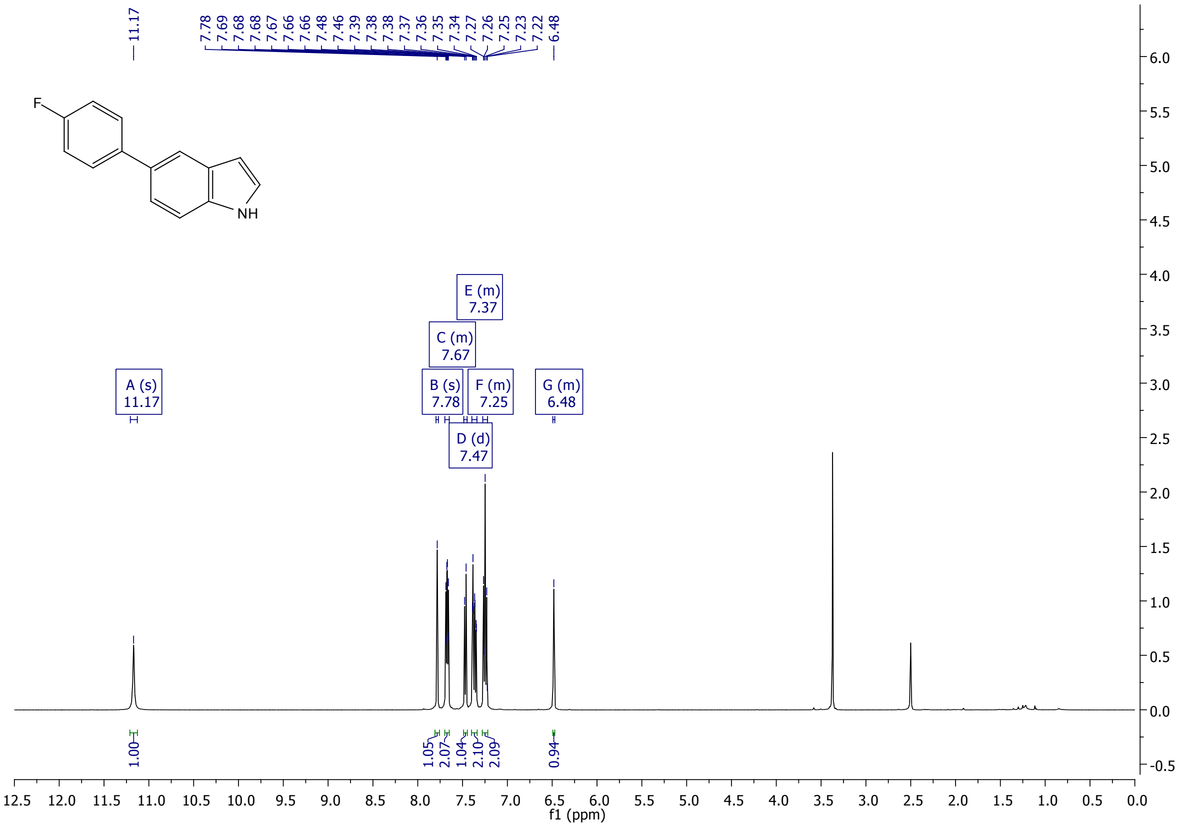

Figure S1.97 ^{1}H NMR spectrum of compound **i-20** in DMSO-d_6.

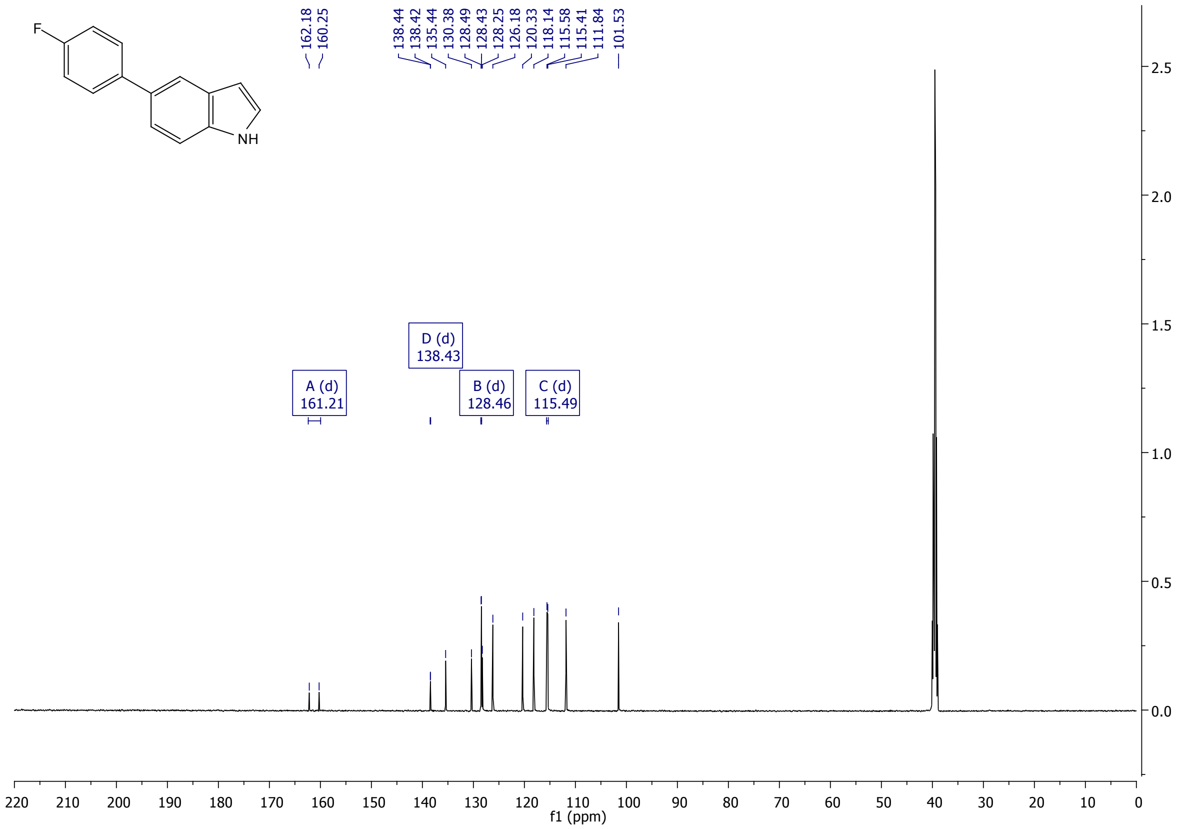

Figure S1.98 ^{13}C NMR spectrum of compound **i-20** in DMSO-d_6.

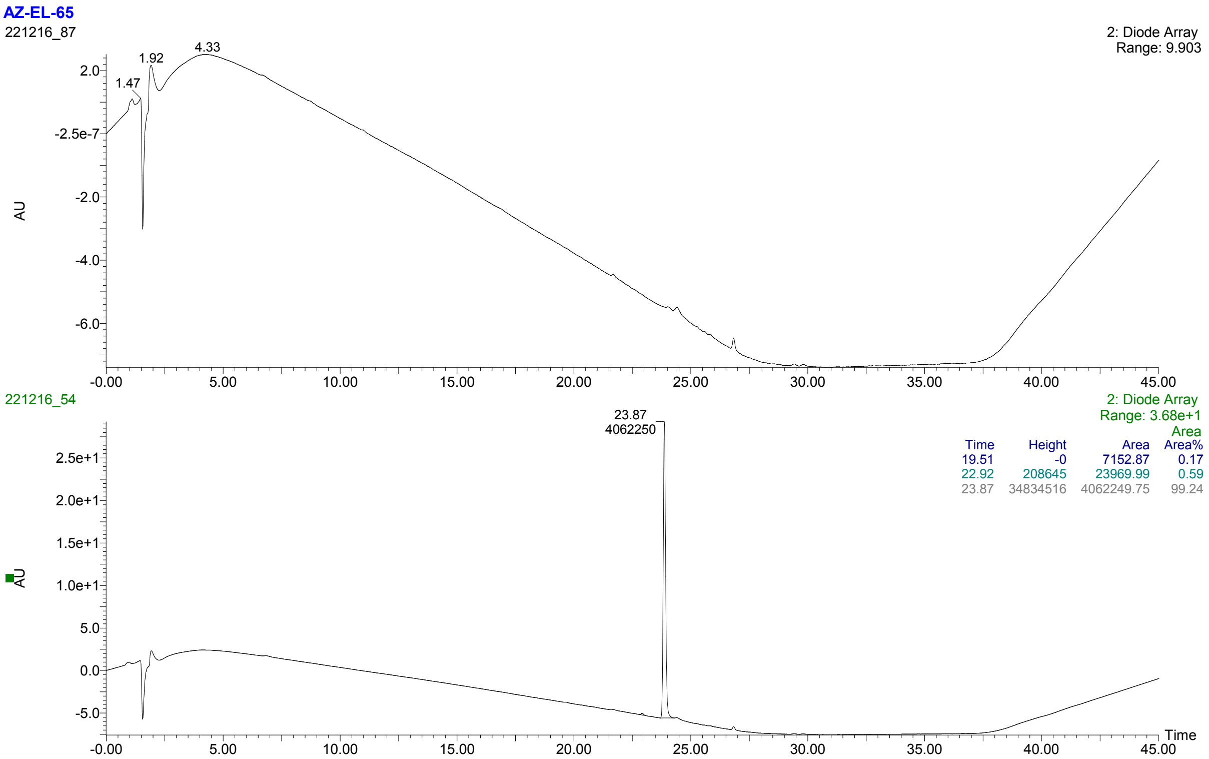

Figure S1.99 LC-MS chromatogram of compound **i-20**.

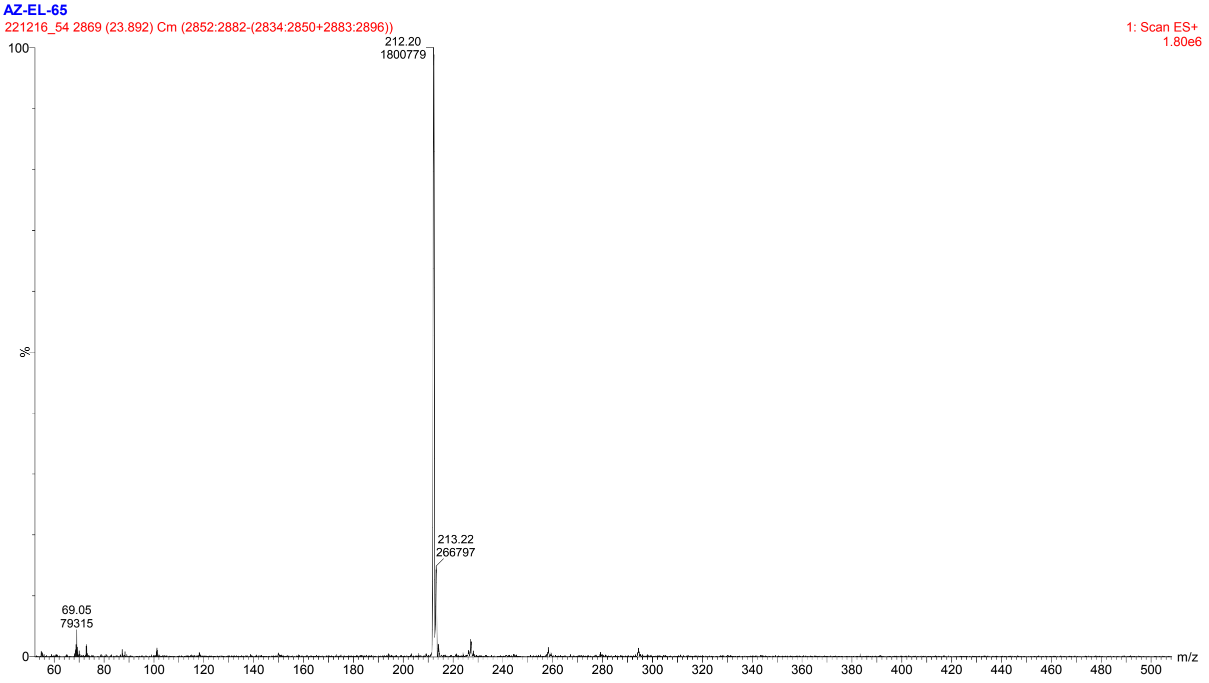

Figure S1.100 MS spectrum of compound **i-20**.

5-(4-(Trifluoromethoxy)phenyl)-1*H*-indole **i-21**

Previously reported in [4]. Prepared in accordance with general procedure **I** from 5-bromo-1*H*-indole (98 mg, 0.50 mmol) and (4-(trifluoromethoxy)phenyl)boronic acid (155 mg, 0.75 mmol). Yield 82%, pale yellow solid; mp = 75.2–76.1 °C, R_f = 0.26 (*n*-hexane/ethyl acetate, 4/1). IR (neat) ν_{max}, cm^{-1}: 3391 (N-H), 1213, 1146, 803, 732, 506. ^1H NMR (500 MHz, DMSO-d_6): δ 6.48-6.50 (1H, m, CH), 7.38-7.43 (4H, m, 4×CH), 7.48 (1H, d, J = 8.4 Hz, CH), 7.77 (2H, d, J = 8.7 Hz, 2×CH), 7.82-7.85 (1H, m, CH), 11.20 (1H, br s, NH). ^{13}C NMR (125 MHz, DMSO-d_6): δ 101.6 (CH), 112.0 (CH), 118.4 (CH), 120.2 (q, $^1J_{C,F}$ = 255.8 Hz, OCF_3), 120.4 (CH), 121.4 (2×CH), 126.3 (CH), 128.27 (C), 128.34 (2×CH), 129.9 (C), 135.7 (C), 141.3 (C), 147.0 (C). MS (pos. mode): m/z (%): 278.3 ($M+H^+$, 100%).

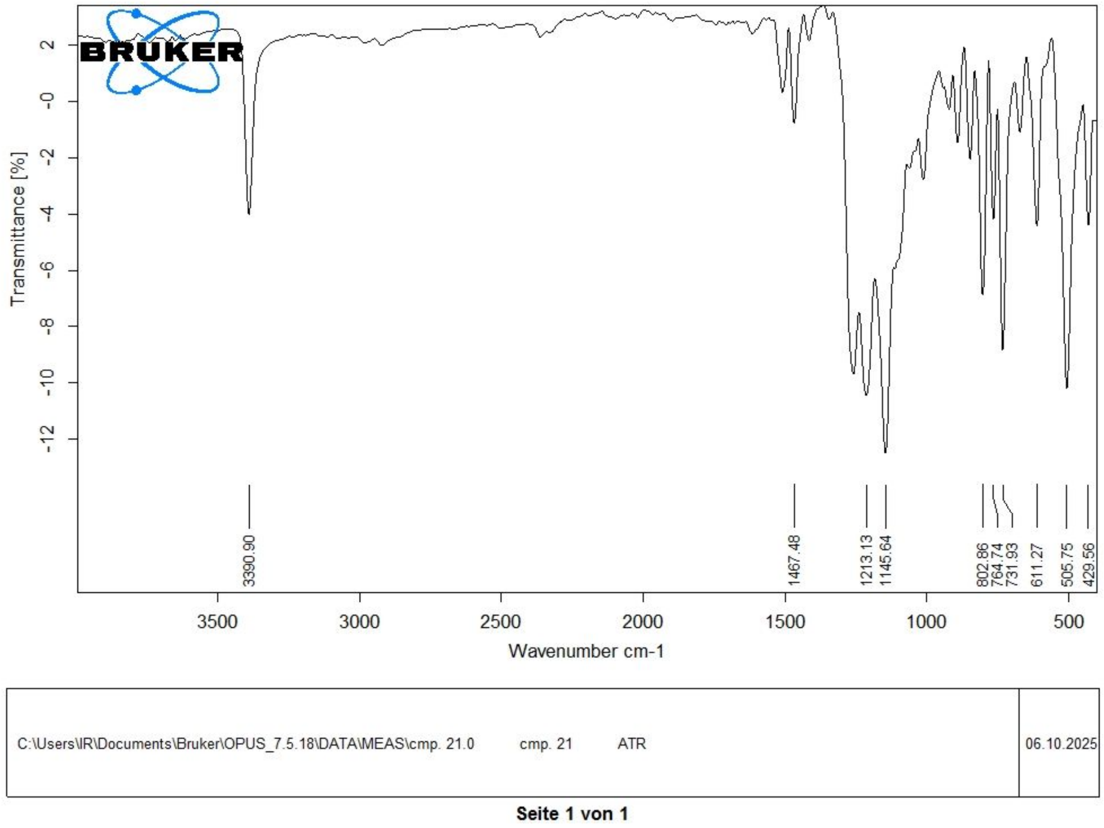

Figure S1.101 FTIR spectrum of compound **i-21**.

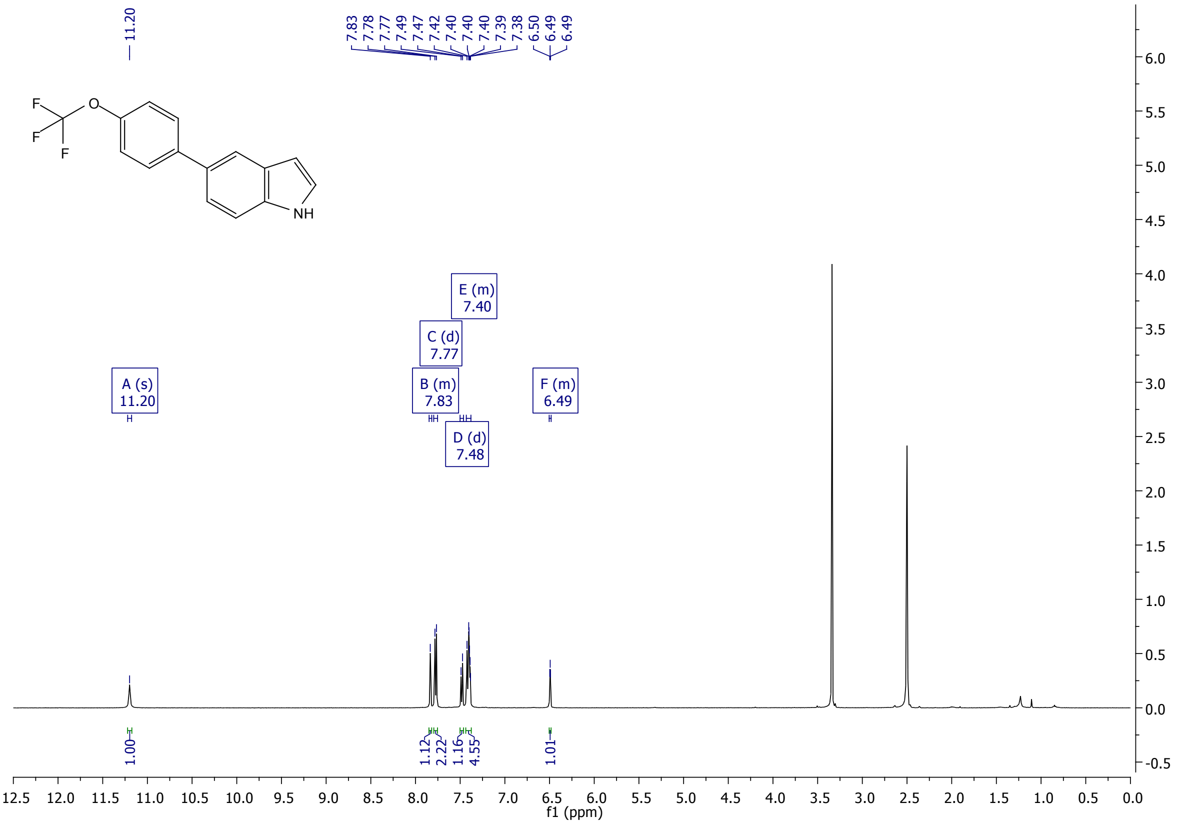

Figure S1.102 ^{1}H NMR spectrum of compound **i-21** in DMSO-d_6.

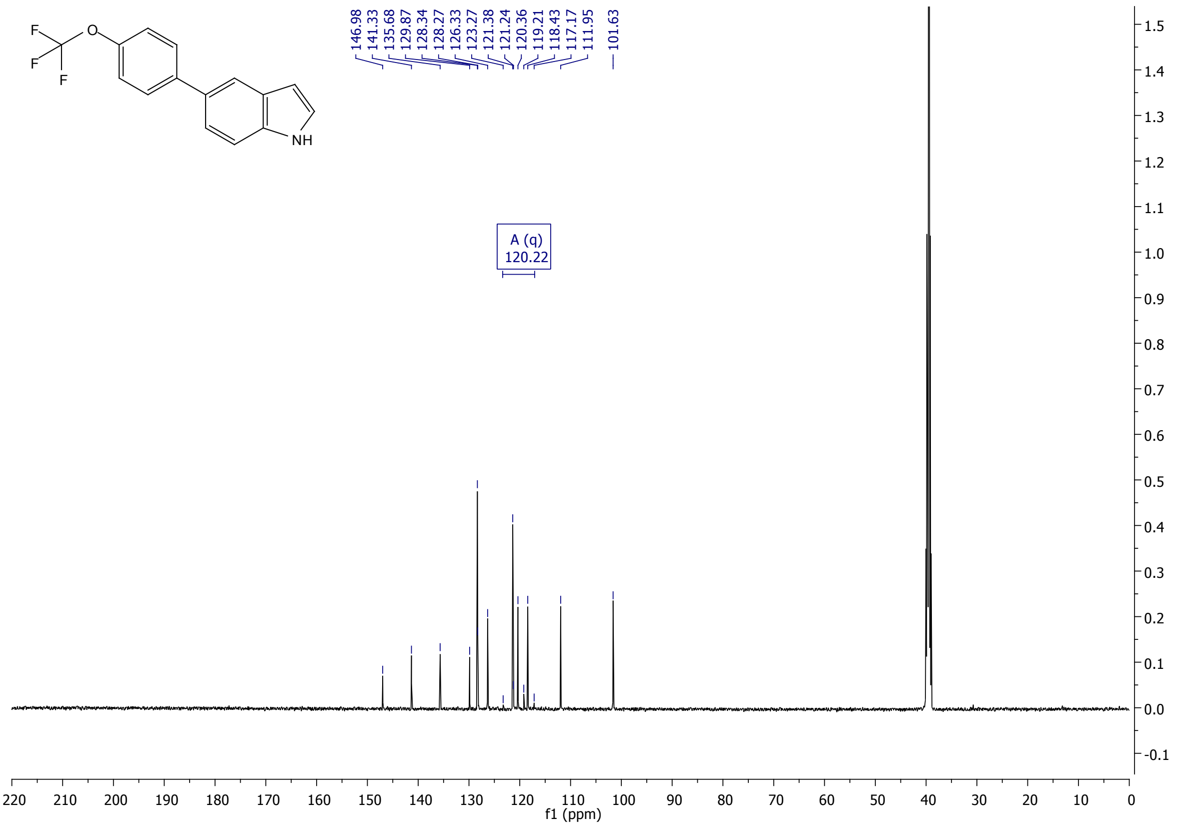

Figure S1.103 ^{13}C spectrum of compound **i-21** in DMSO-d_6.

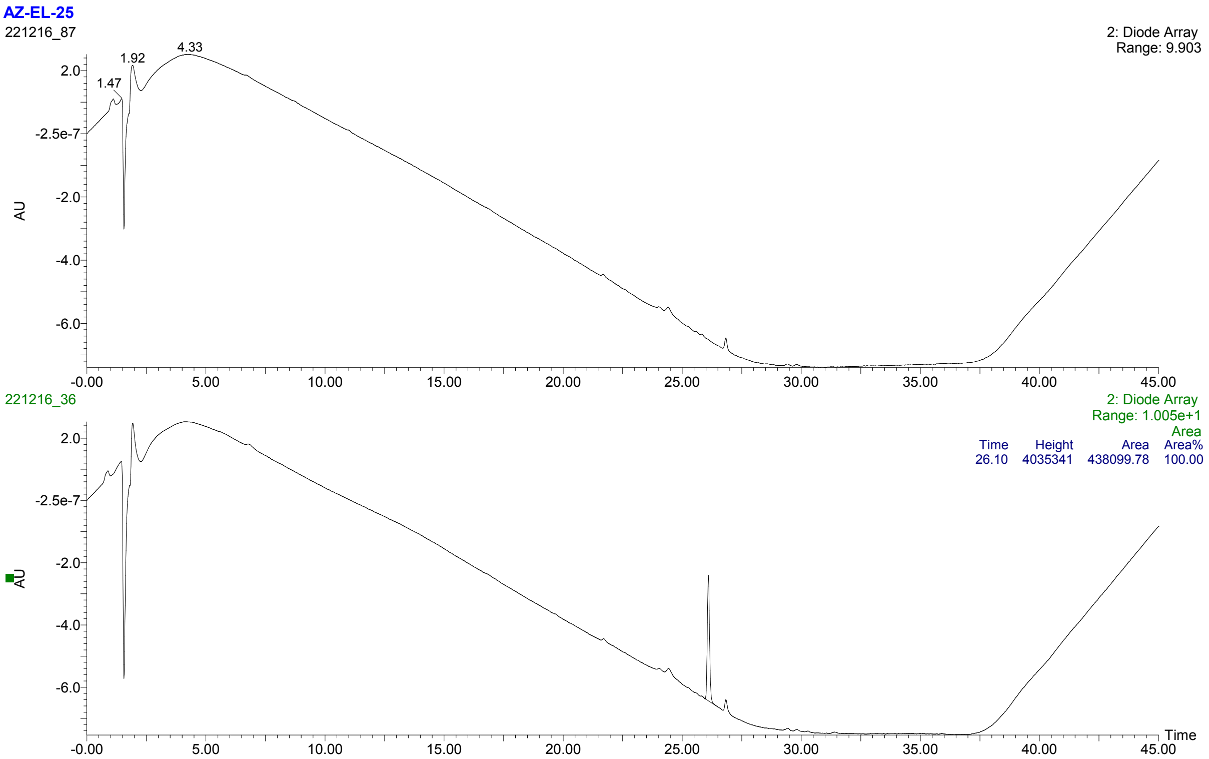

Figure S1.104 LC-MS chromatogram of compound **i-21**.

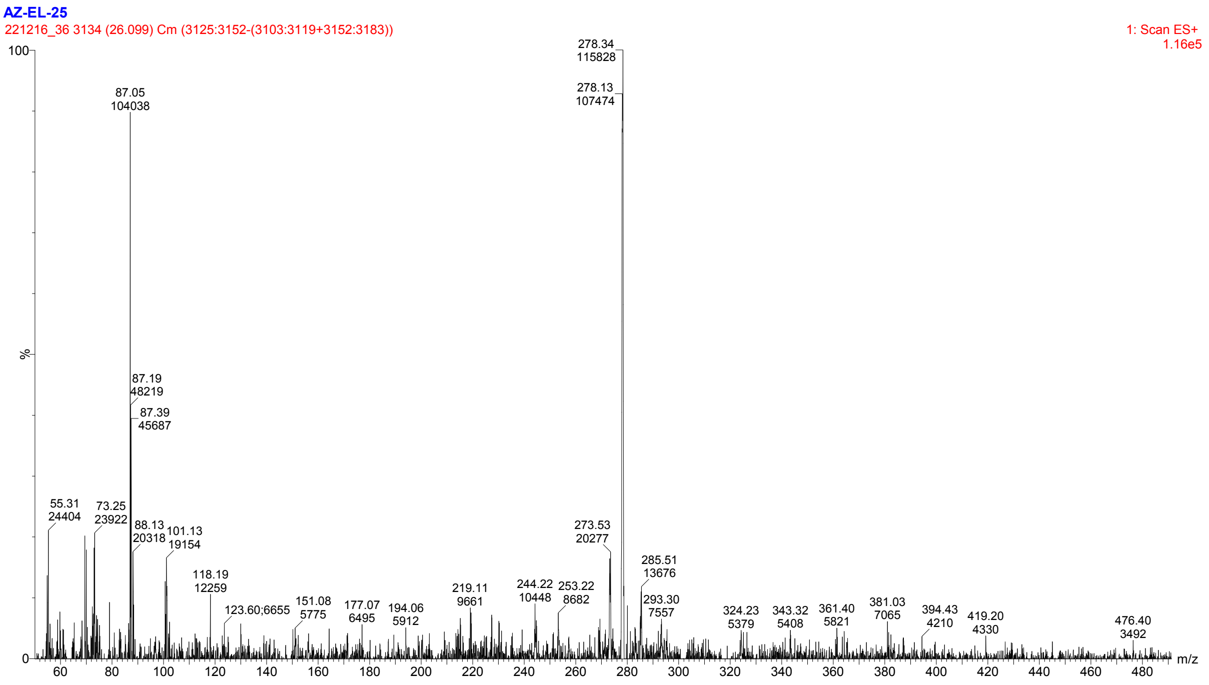

Figure S1.105 MS spectrum of compound **i-21**.

2-Chloro-4-(1*H*-indol-5-yl)phenol **i-22**

Prepared in accordance with general procedure **I** from 5-bromo-1*H*-indole (120 mg, 0.61 mmol) and (3-chloro-4-hydroxyphenyl)boronic acid (157 mg, 0.92 mmol). Yield 62%, beige solid; mp = 115.6–117.1 °C, R_f = 0.14 (*n*-hexane/ethyl acetate, 4/1). IR (neat) ν_{max}, cm^{-1}: 3418 (N-H), 3288 (O-H), 1462, 1222, 802, 765, 728, 471, 431. ^{1}H NMR (500 MHz, DMSO-d_6): δ 6.43-6.47 (1H, m, CH), 7.03 (1H, d, *J* = 8.4 Hz, CH), 7.31 (1H, dd, *J* = 8.5 Hz, 1.6 Hz, CH), 7.36 (1H, t, *J* = 2.7 Hz, CH), 7.40-7.46 (2H, m, 2×CH), 7.58 (1H, d, *J* = 2.2 Hz, CH), 7.72-7.75 (1H, m, CH), 10.14 (1H, br s, OH), 11.11 (1H, br s, NH). ^{13}C NMR (125 MHz, DMSO-d_6): δ 101.5 (CH), 111.8 (CH), 117.0 (CH), 117.6 (CH), 119.98 (CH), 120.02 (C), 126.1 (CH), 126.2 (CH), 127.7 (CH), 128.2 (C), 130.1 (C), 134.2 (C), 135.2 (C), 151.7 (C). MS (pos. mode): m/z (%): 244.2/246.2 (M+H$^+$, 100%).

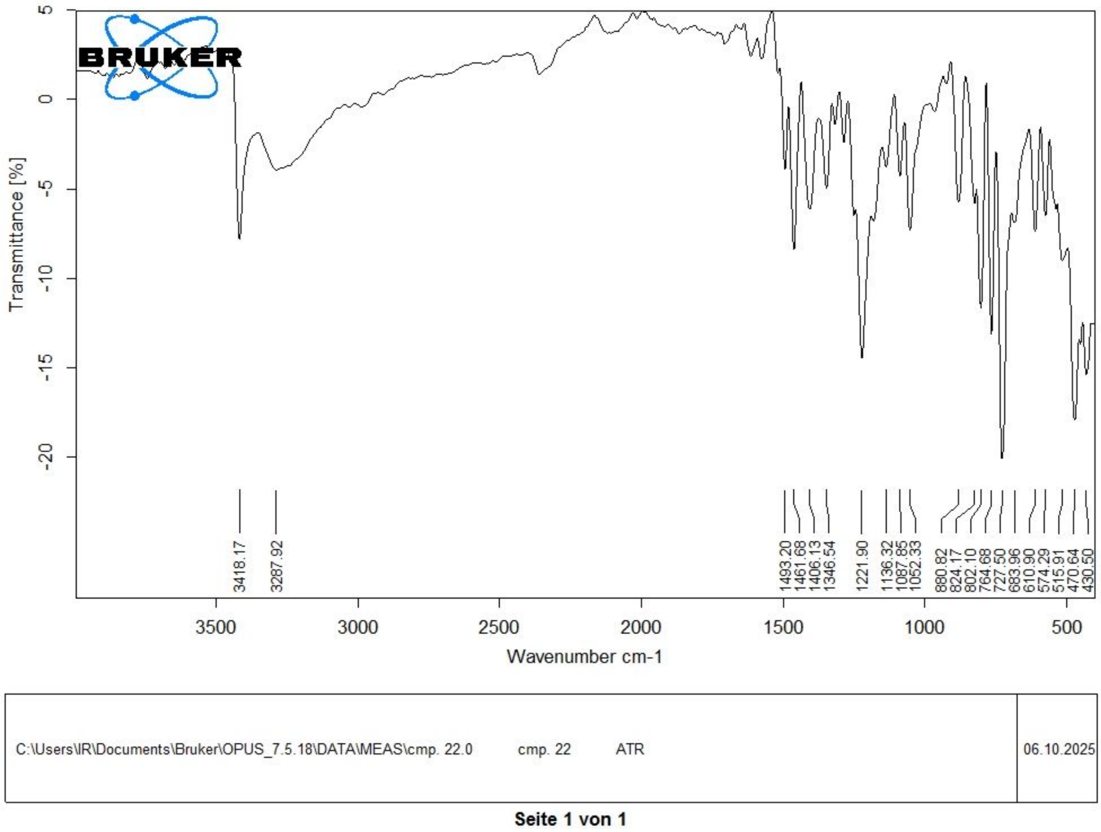

Figure S1.106 FTIR spectrum of compound **i-22**.

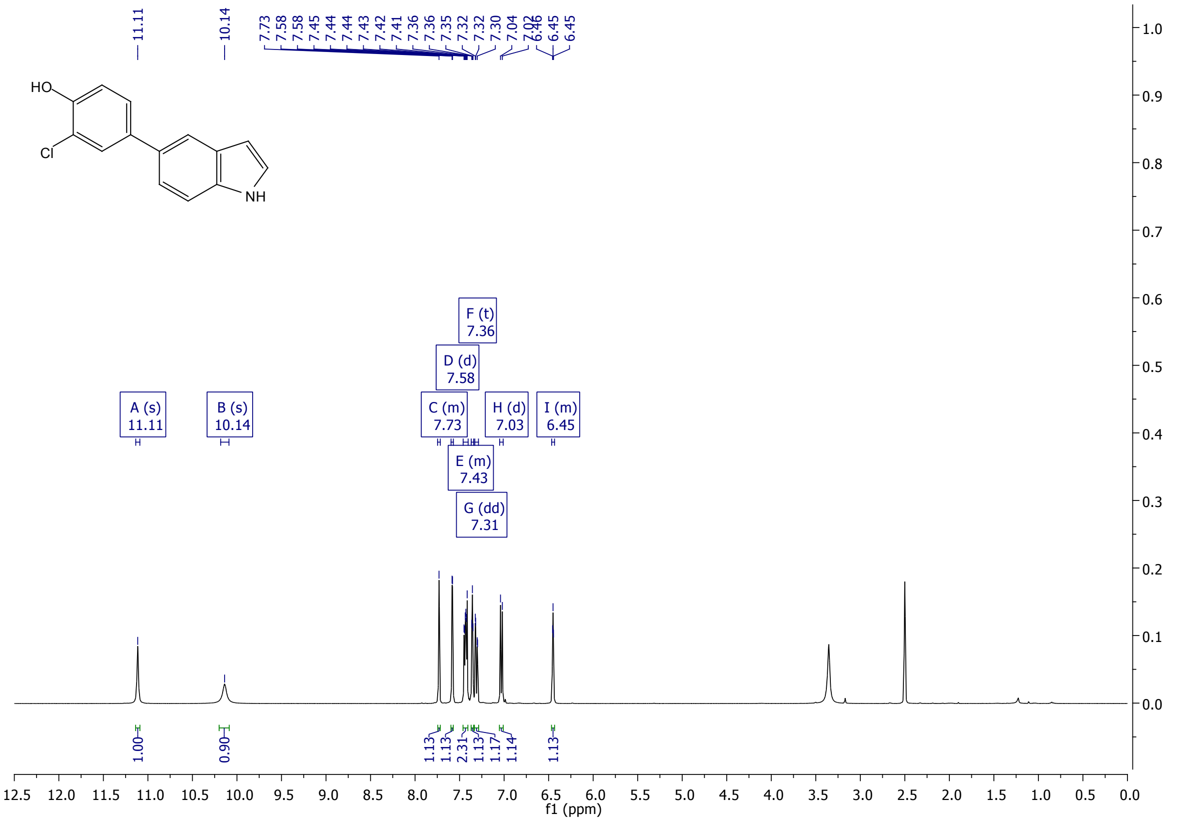

Figure S1.107 ^{1}H NMR spectrum of compound **i-22** in DMSO-d_6.

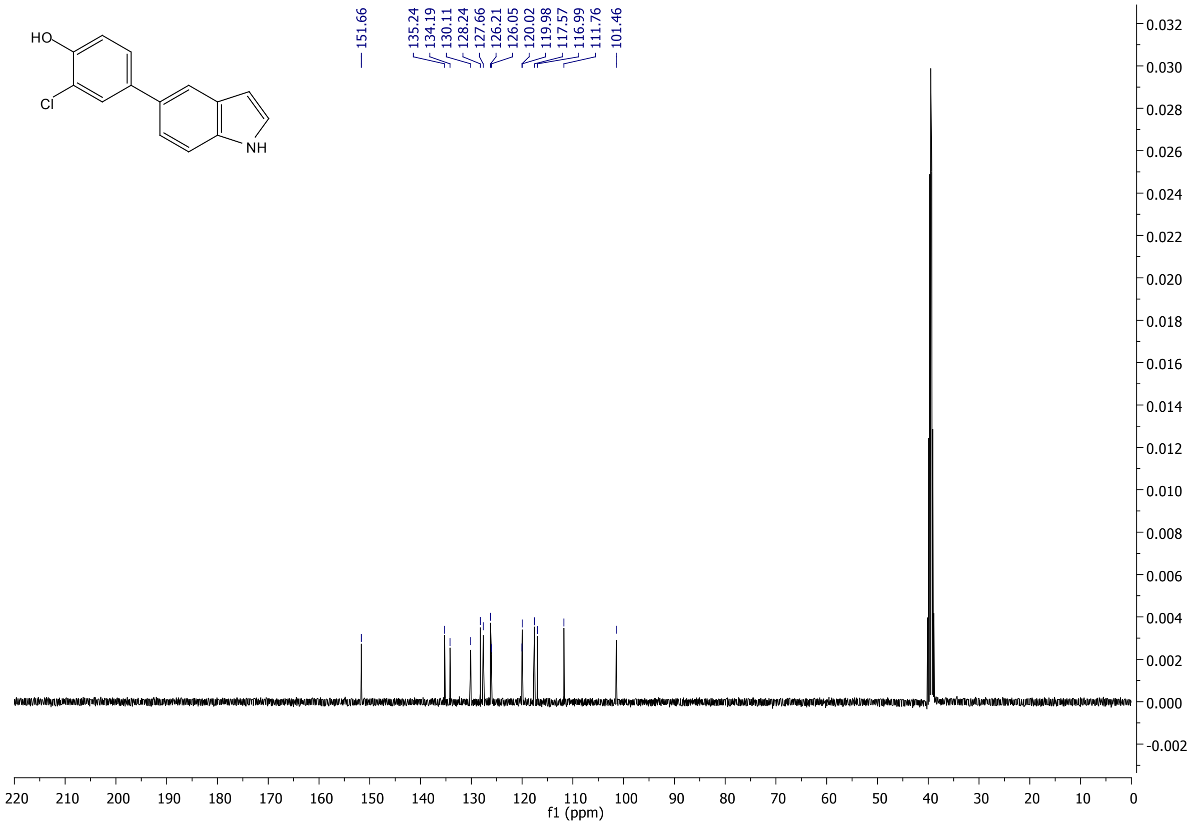

Figure S1.108 ^{13}C NMR spectrum of compound **i-22** in DMSO-d_6.

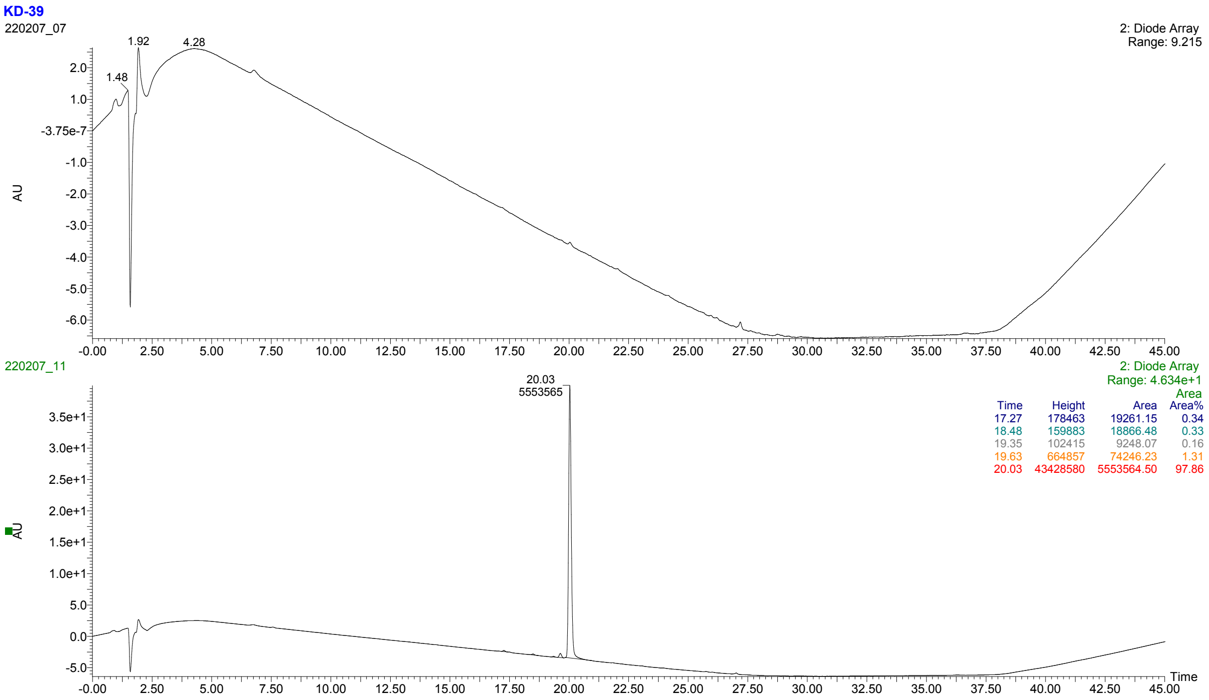

Figure S1.109 LC-MS chromatogram of compound **i-22**.

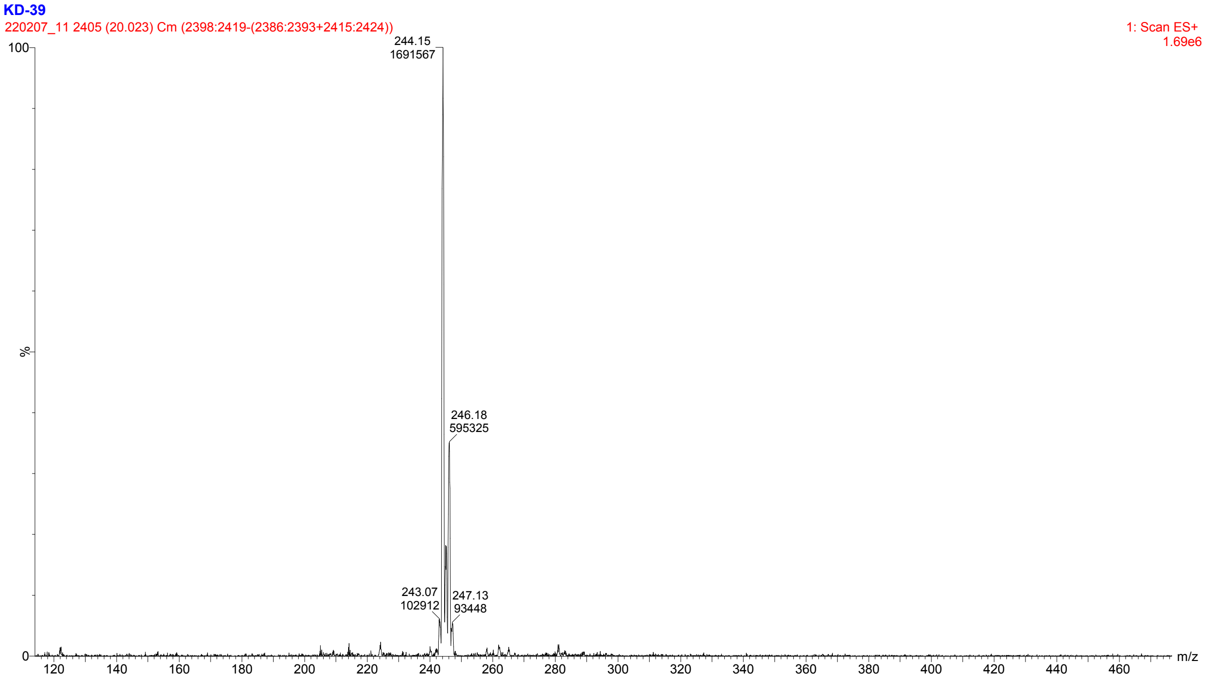

Figure S1.110 MS spectrum of compound **i-22**.

5-(3-Chloro-4-propoxyphenyl)-1*H*-indole **i-23**

Prepared in accordance with general procedure **II** from 2-chloro-4-(1*H*-indol-5-yl)phenol **i-22** (85 mg, 0.35 mmol) and 1-iodopropane (51 µl, 0.53 mmol). Yield 33%, beige solid; mp = 98.0–99.3 °C, R_f = 0.24 (*n*-hexane/ethyl acetate, 4/1). IR (neat) ν_{max}, cm^{-1}: 3390 (N-H), 1461, 1253, 1056, 1013, 798, 736, 516. ^1H NMR (500 MHz, DMSO-d_6): δ 1.02 (3H, t, J = 7.4 Hz, CH_3), 1.78 (2H, sext, J = 7.0 Hz, CH_2), 4.05 (2H, t, J = 6.4 Hz, CH_2), 6.45-6.47 (1H, m, CH), 7.19 (1H, d, J = 8.6 Hz, CH), 7.33-7.38 (1H, m, CH), 7.37 (1H, t, J = 2.6 Hz, CH), 7.44 (1H, d, J = 8.4 Hz, CH), 7.57 (1H, dd, J = 8.5 Hz, 2.2 Hz, CH), 7.68 (1H, d, J = 2.2 Hz, CH), 7.77-7.79 (1H, m, CH), 11.14 (1H, br s, NH). ^{13}C NMR (125 MHz, DMSO-d_6): δ 10.4 (CH_3), 22.1 (CH_2), 70.1 (CH_2), 101.5 (CH), 111.8 (CH), 114.2 (CH), 117.8 (CH), 120.0 (CH), 121.8 (C), 126.15 (CH), 126.23 (CH), 127.7 (CH), 128.3 (C), 129.7 (C), 135.38 (C), 135.40 (C), 152.5 (C). MS (pos. mode): m/z (%): 286.2/288.2 ($M+H^+$, 100%).

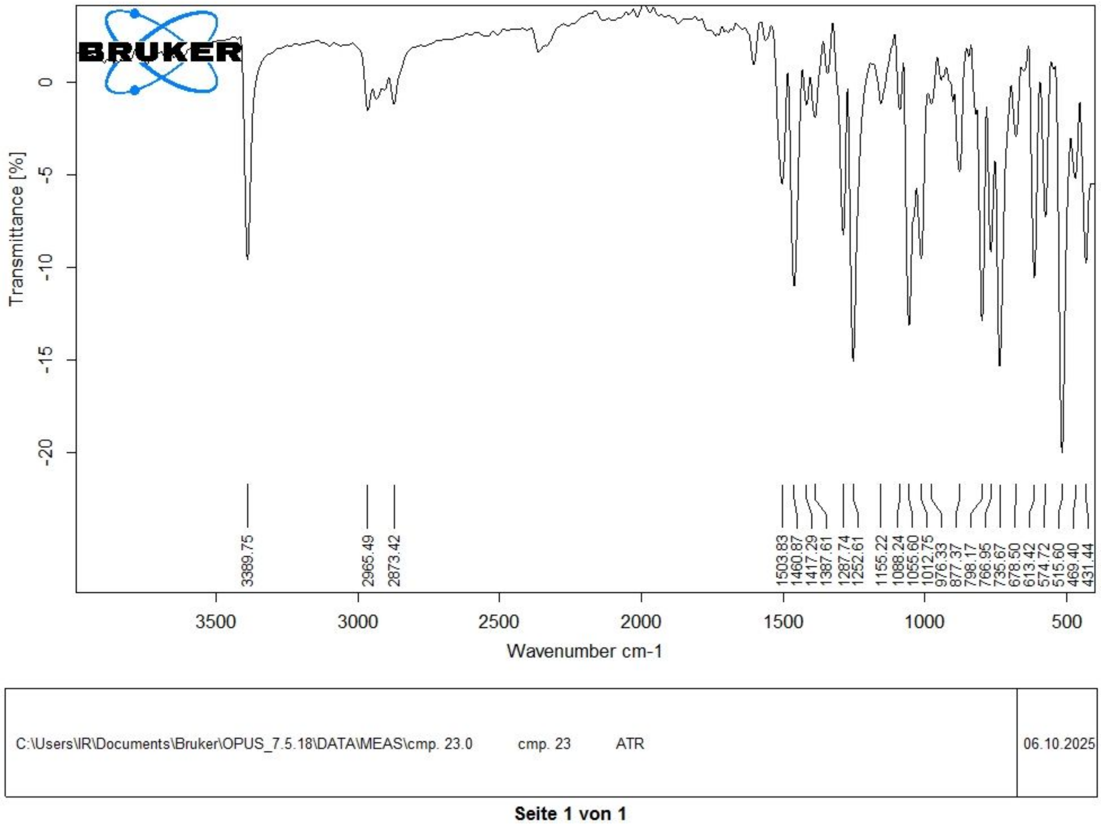

Figure S1.111 FTIR spectrum of compound **i-23**.

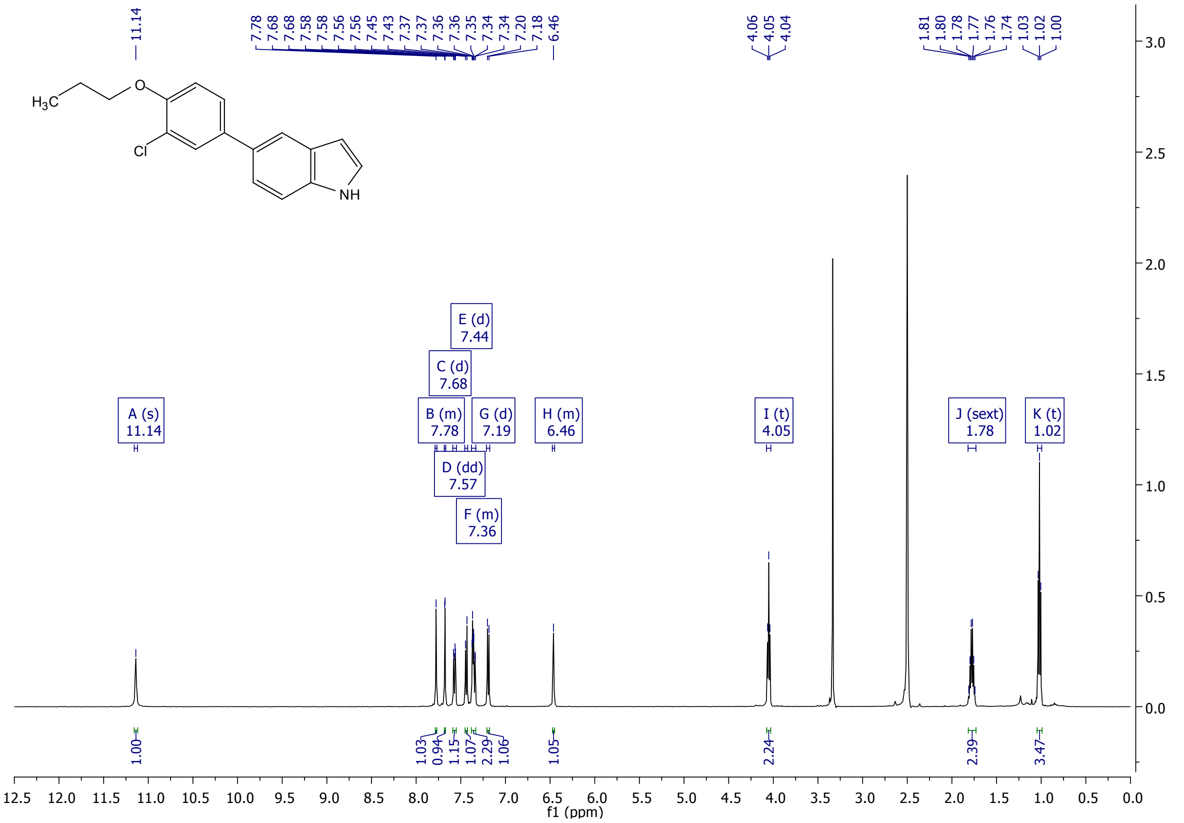

Figure S1.112 ^{1}H NMR spectrum of compound **i-23** in DMSO-d_6.

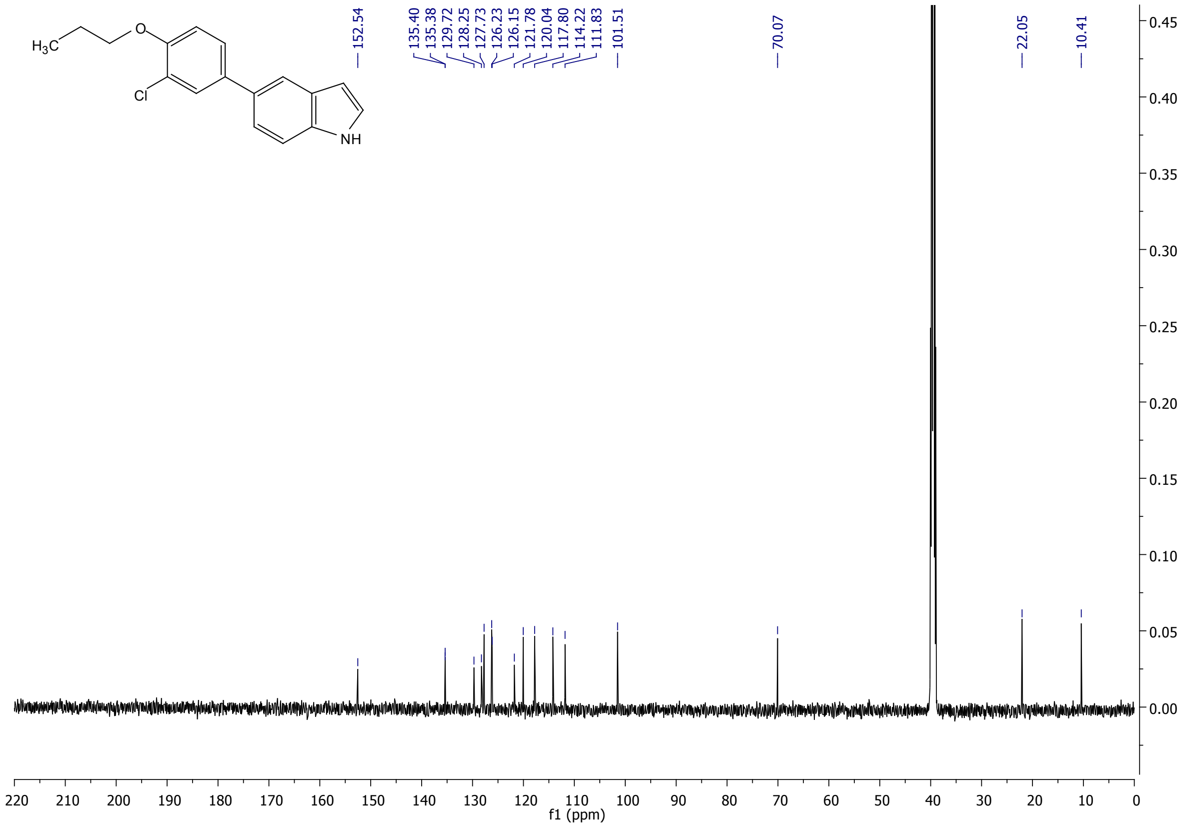

Figure S1.113 ^{13}C NMR spectrum of compound **i-23** in DMSO-d_6.

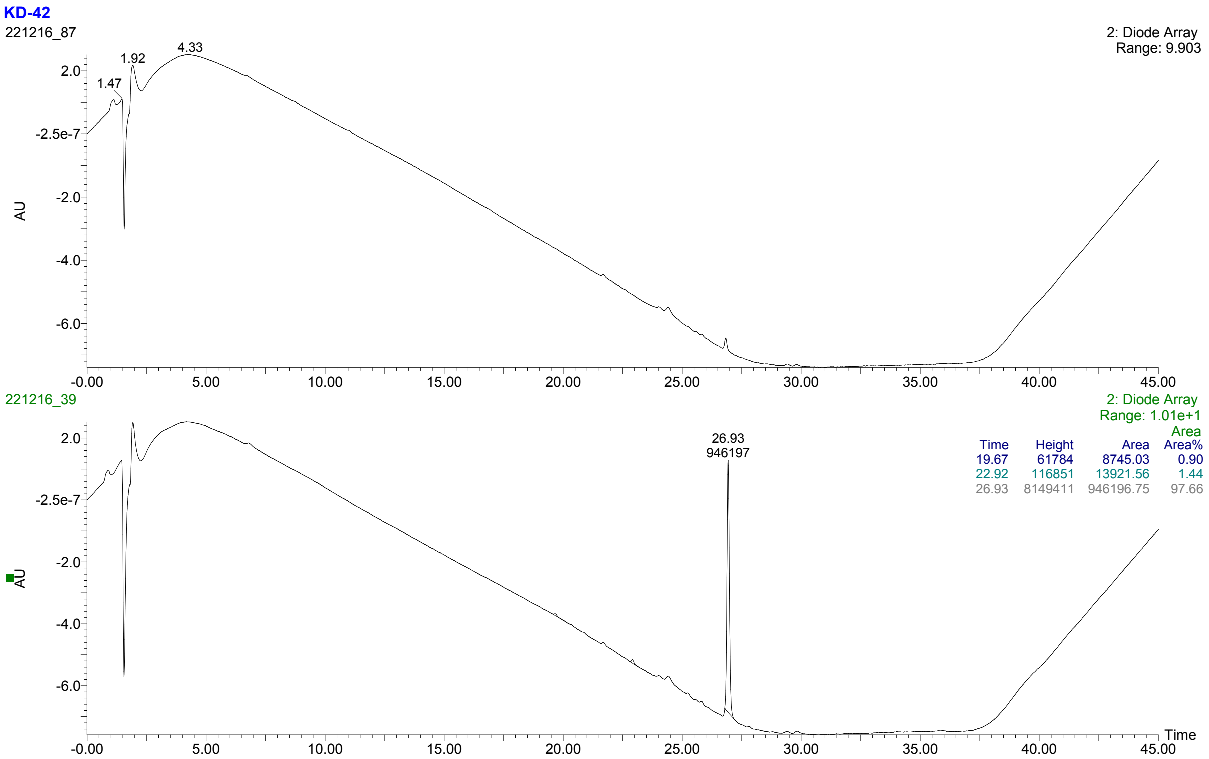

Figure S1.114 LC-MS chromatogram of compound **i-23**.

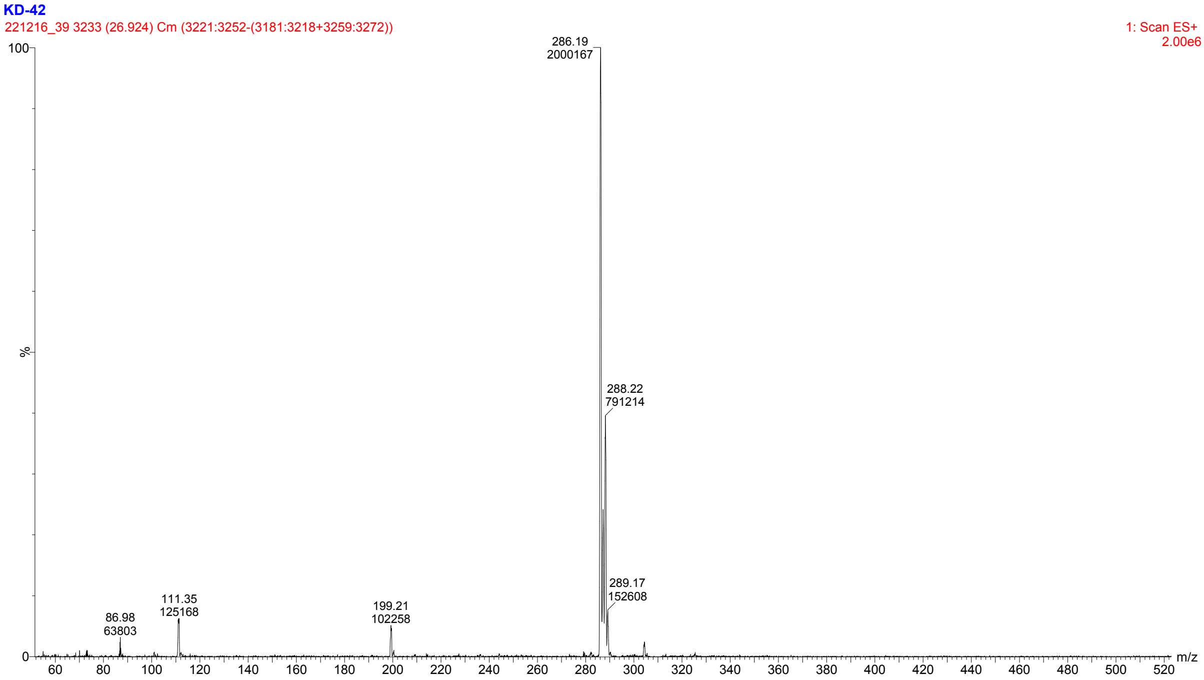

Figure S1.115 MS spectrum of compound **i-23**.

2-Chloro-5-(1*H*-indol-5-yl)phenol **i-24**

Prepared in accordance with general procedure **I** from 5-bromo-1*H*-indole (137 mg, 0.70 mmol) and (4-chloro-3-hydroxyphenyl)boronic acid (181 mg, 1.05 mmol). Yield 70%, beige solid; mp = 117.6–118.2 °C, R_f = 0.23 (*n*-hexane/ethyl acetate, 7/3). IR (neat) ν_{max}, cm^{-1}: 3487 (N-H), 3406 (O-H), 1314, 1175, 799, 764, 734, 501, 421. ^{1}H NMR (500 MHz, DMSO-d_6): δ 6.47-6.50 (1H, m, CH), 7.08 (1H, dd, *J* = 8.3 Hz, 2.1 Hz, CH), 7.22 (1H, d, *J* = 2.1 Hz, CH), 7.30 (1H, dd, *J* = 8.5 Hz, 1.7 Hz, CH), 7.35 (1H, d, *J* = 8.3 Hz, CH), 7.37-7.39 (1H, m, CH), 7.46 (1H, d, *J* = 8.4 Hz, CH), 7.72-7.73 (1H, m, CH), 10.20 (1H, br s, OH), 11.18 (1H, br s, NH). ^{13}C NMR (125 MHz, DMSO-d_6): δ 101.6 (CH), 111.9 (CH), 114.7 (CH), 117.7 (C), 118.0 (CH), 118.5 (CH), 120.1 (CH), 126.2 (CH), 128.2 (C), 130.0 (CH), 130.4 (C), 135.6 (C), 142.1 (C), 153.2 (C). MS (pos. mode): m/z (%): 244.0/246.1 (M+H^+, 100%).

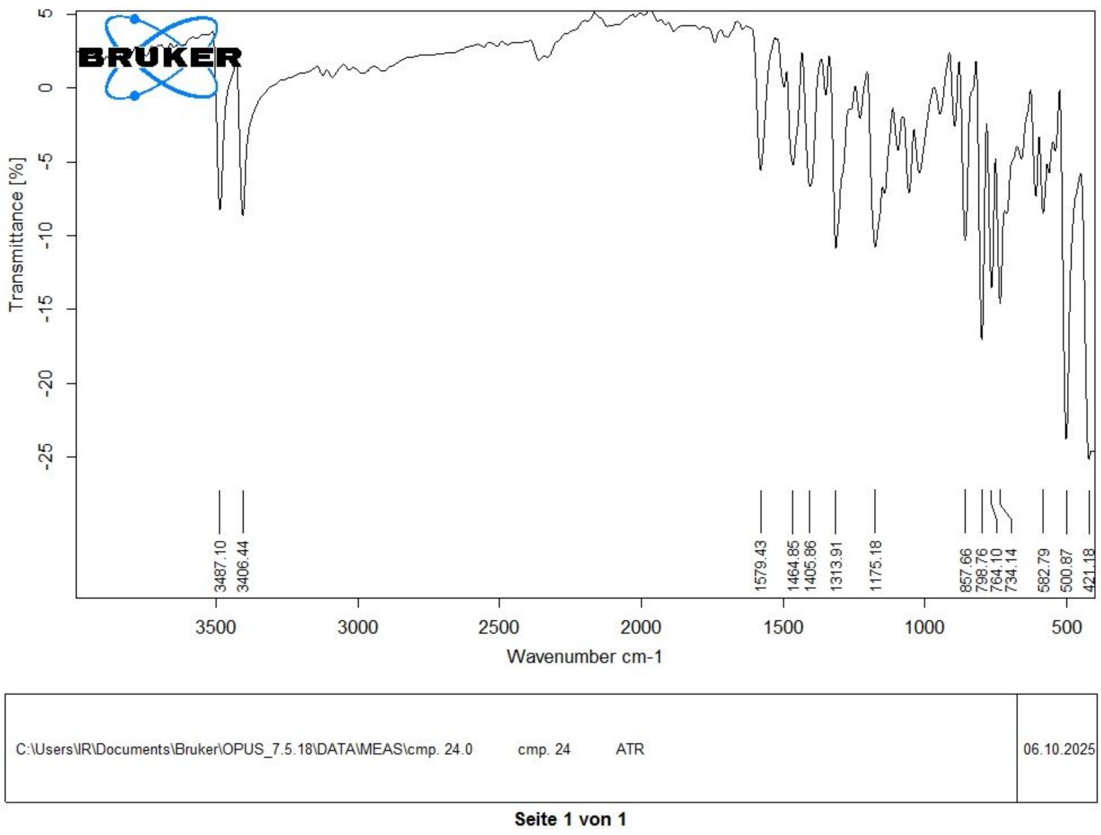

Figure S1.116 FTIR spectrum of compound **i-24**.

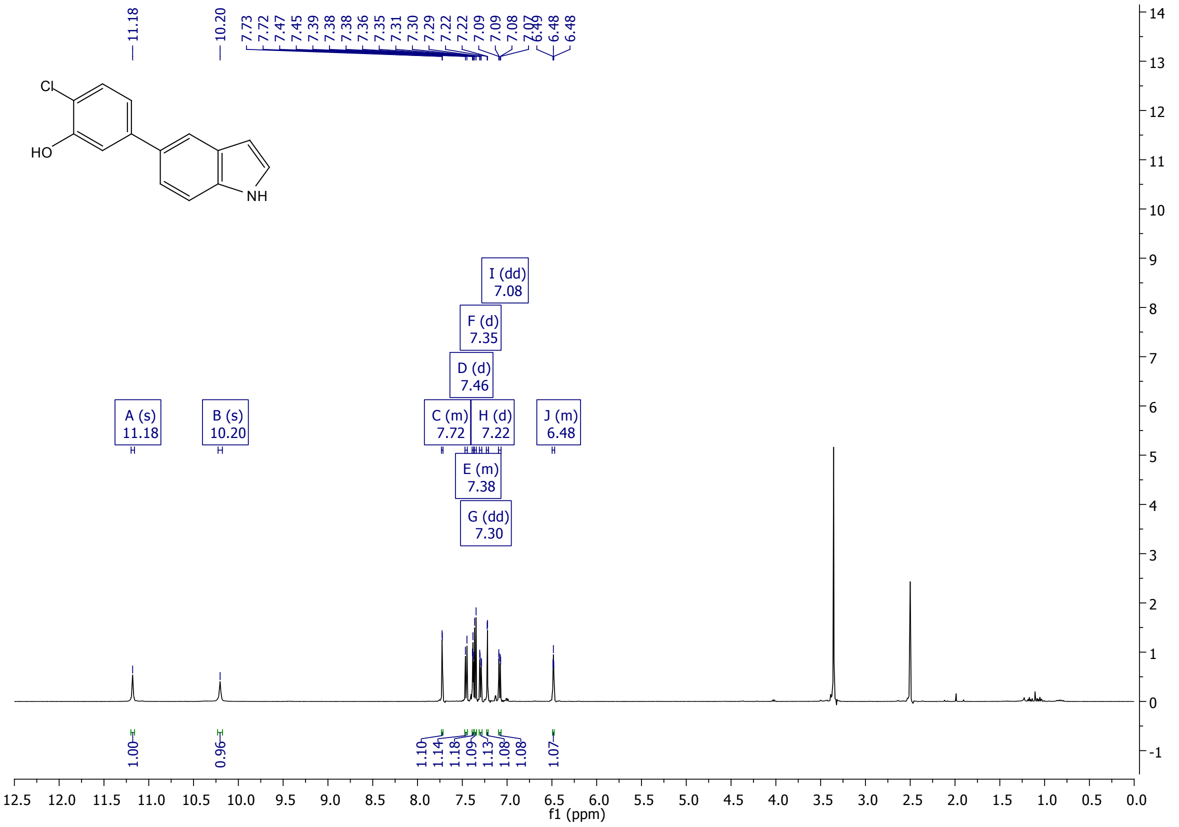

Figure S1.117 ^{1}H NMR spectrum of compound **i-24** in DMSO-d_6.

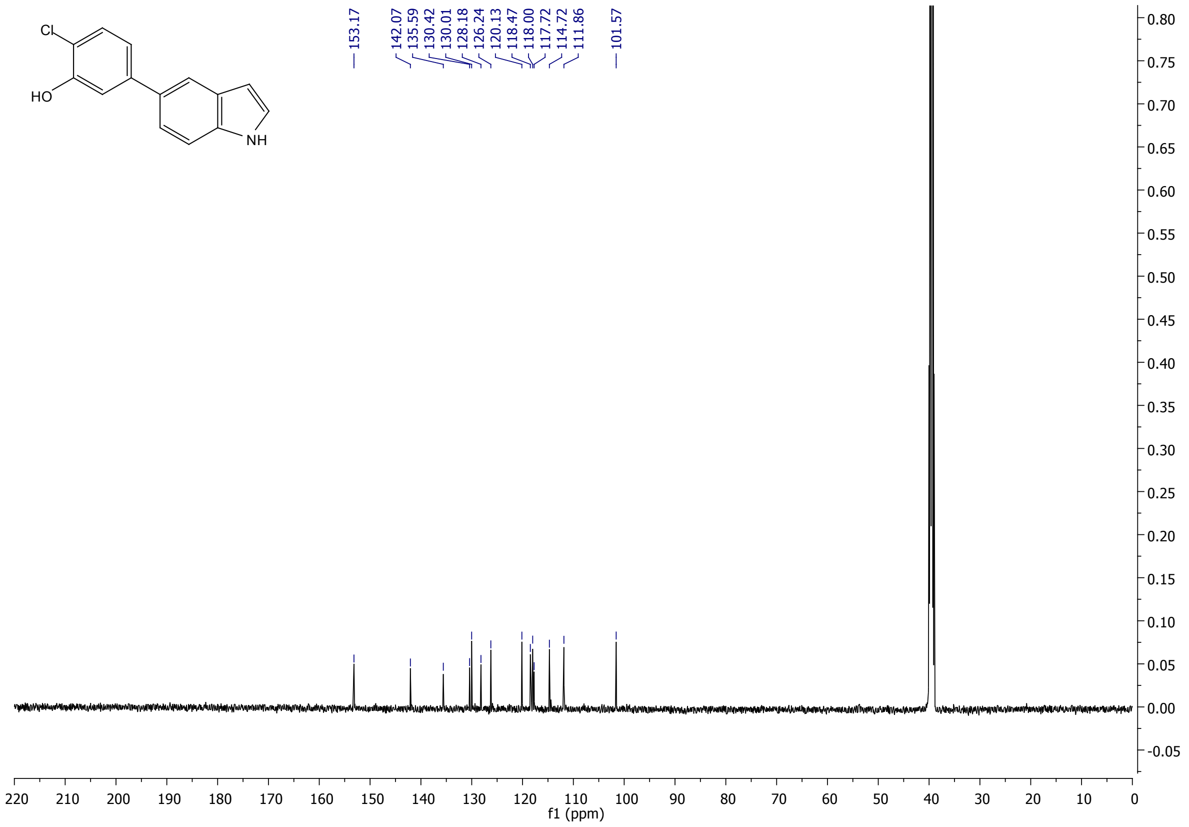

Figure S1.118 ^{13}C NMR spectrum of compound **i-24** in DMSO-d_6.

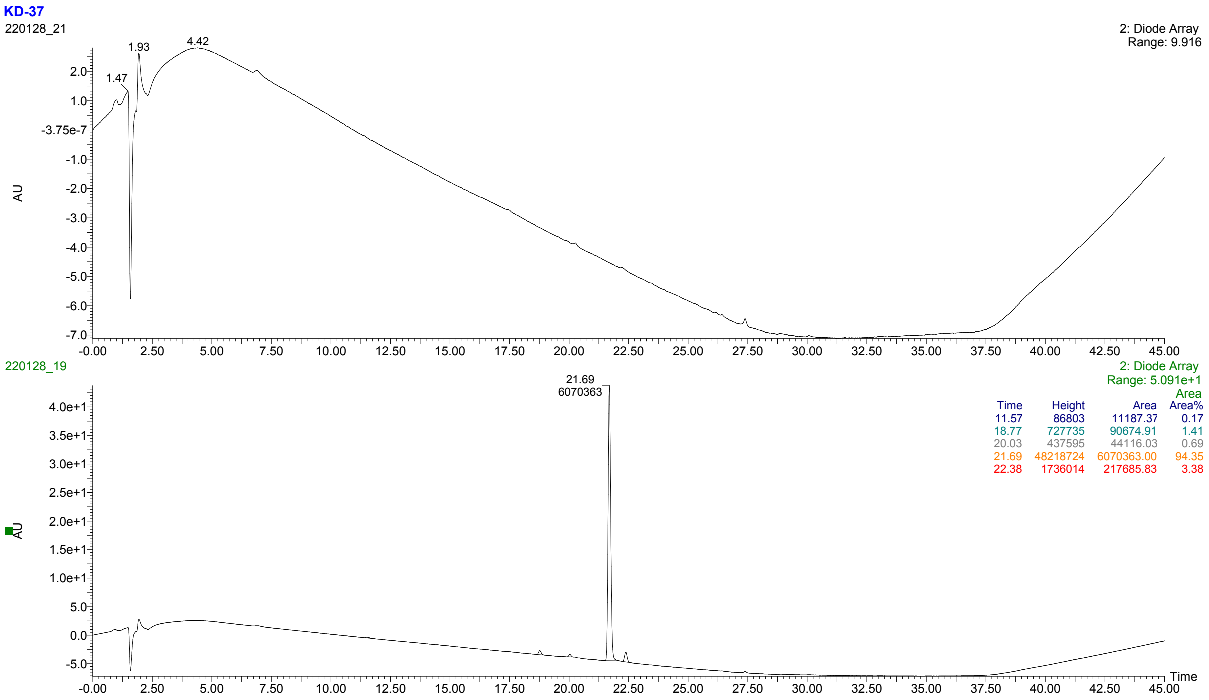

Figure S1.119 LC-MS chromatogram of compound **i-24**.

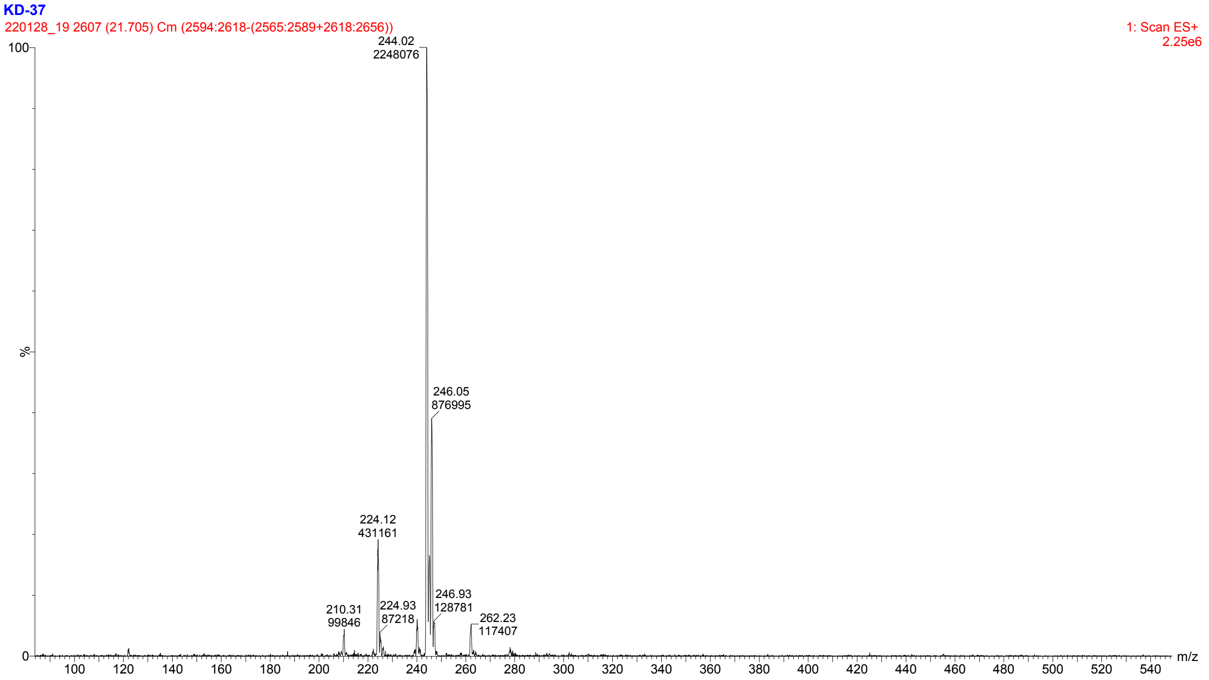

Figure S1.120 MS spectrum of compound **i-24**.

5-(4-Chloro-3-ethoxyphenyl)-1*H*-indole **i-25**

Prepared in accordance with general procedure **II** from 2-chloro-5-(1*H*-indol-5-yl)phenol **i-24** (137 mg, 0.56 mmol) and iodoethane (68 µL, 0.84 mmol). Yield 72%, beige solid; mp = 105.9–107.2 °C, R_f = 0.28 (*n*-hexane/ethyl acetate, 4/1). IR (neat) ν_{max}, cm^{-1}: 3433 (N-H), 1461, 1310, 1226, 1063, 1031, 806, 763, 726, 468, 426. ^{1}H NMR (500 MHz, DMSO-d_6): δ 1.39 (3H, t, J = 6.9 Hz, CH_3), 4.24 (2H, q, J = 6.9 Hz, CH_2), 6.47-6.50 (1H, m, CH), 7.22 (1H, dd, J = 8.2, 1.8 Hz, CH), 7.34-7.35 (1H, m, CH), 7.38-7.48 (4H, m, 4×CH), 7.85-7.87 (1H, m, CH), 11.18 (1H, br s, NH). ^{13}C NMR (125 MHz, DMSO-d_6): δ 14.7 (CH_3), 64.2 (CH_2), 101.6 (CH), 111.8 (CH), 112.0 (CH), 118.4 (CH), 119.3 (C), 119.5 (CH), 120.4 (CH), 126.3 (CH), 128.2 (C), 130.0 (CH), 130.4 (C), 135.7 (C), 142.4 (C), 154.0 (C). MS (pos. mode): m/z (%): 272.2/274.1 (M+H$^+$, 100%).

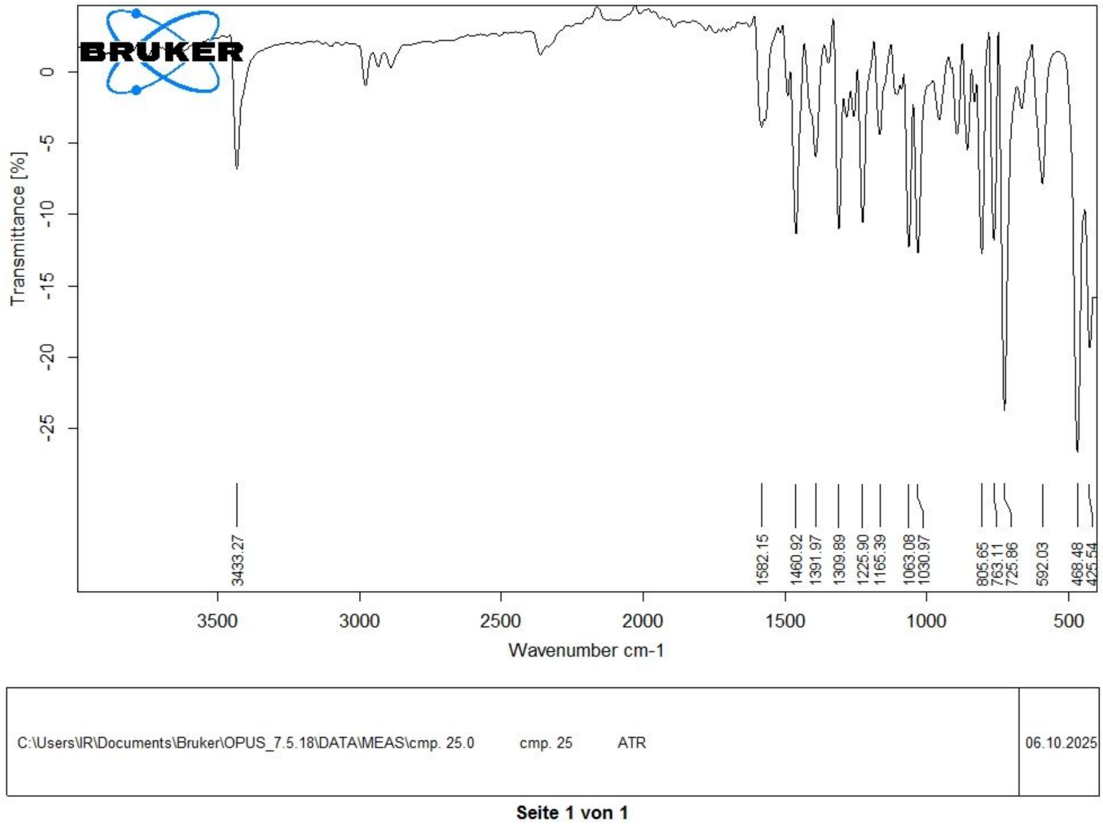

Figure S1.121 FTIR spectrum of compound **i-25**.

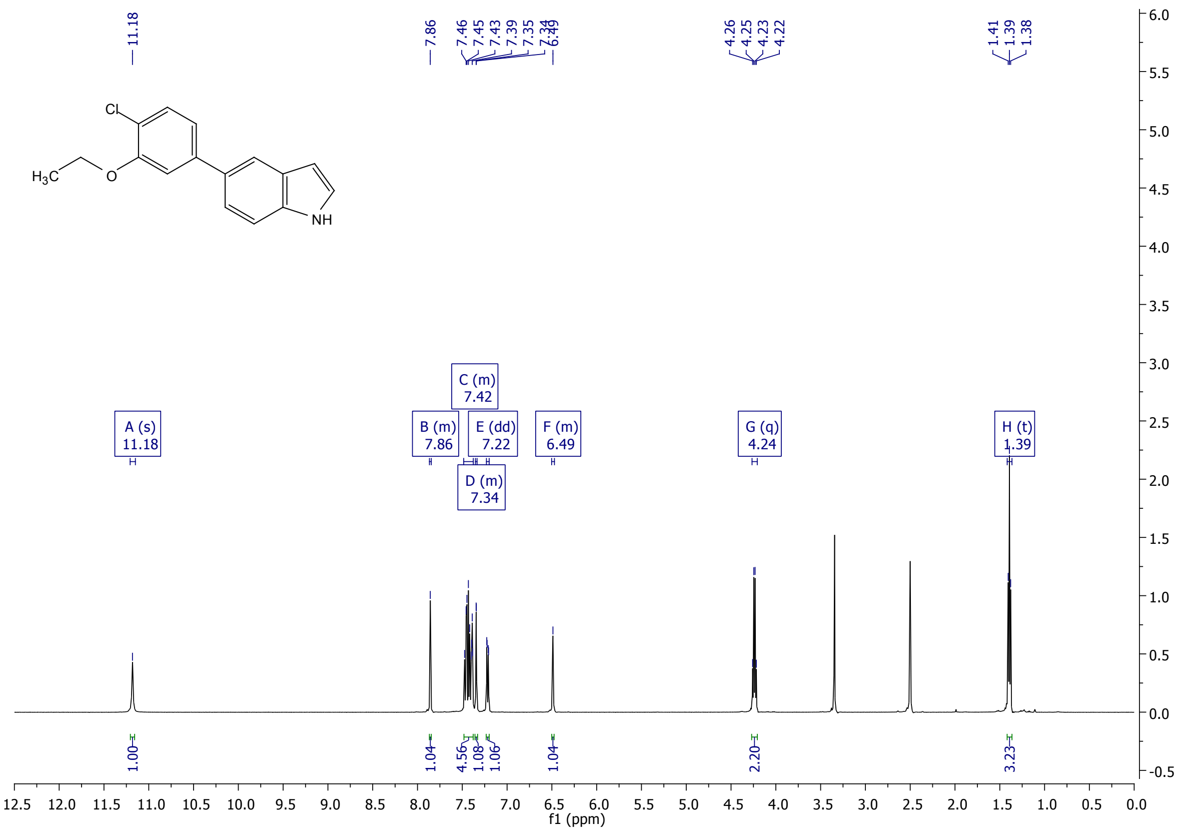

Figure S1.122 ^{1}H NMR spectrum of compound **i-25** in DMSO-d_6.

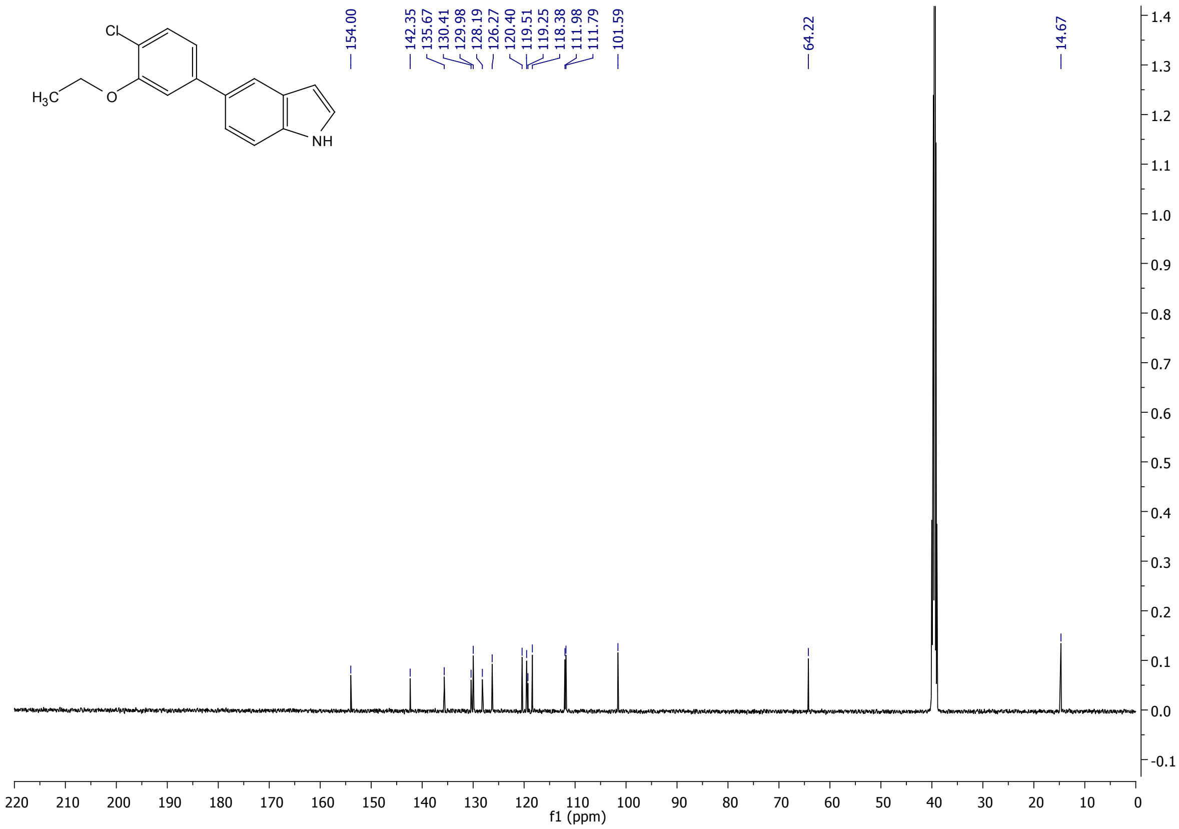

Figure S1.123 ^{13}C NMR spectrum of compound **i-25** in DMSO-d_6.

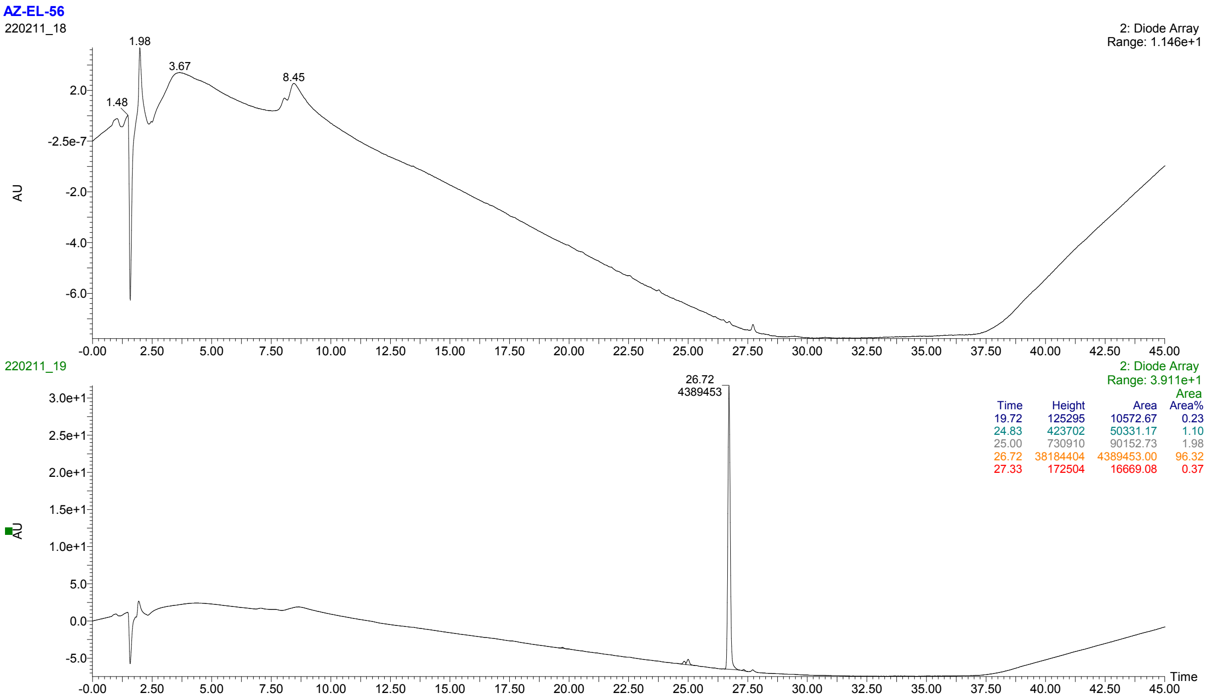

Figure S1.124 LC-MS chromatogram of compound **i-25**.

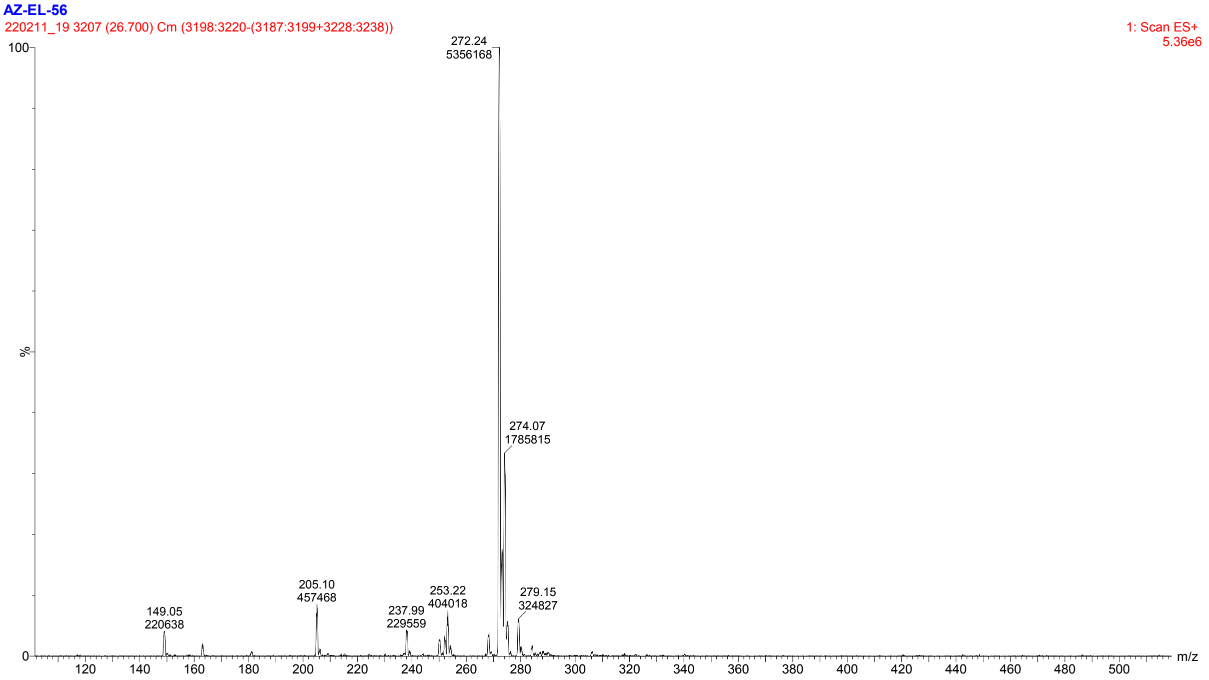

Figure S1.125 MS spectrum of compound **i-25**.

5-(4-Chloro-3-propoxyphenyl)-1*H*-indole **i-26**

Prepared in accordance with general procedure **II** from 2-chloro-5-(1*H*-indol-5-yl)phenol **i-24** (80 mg, 0.33 mmol) and 1-iodopropane (48 μL, 0.49 mmol). Yield 68%, dark brown viscous oil, R_f = 0.31 (*n*-hexane/ethyl acetate, 4/1). IR (neat) ν_{max}, cm^{-1}: 3414 (N-H), 1460, 1314, 1222, 1063, 980, 799, 766, 723, 460, 421. ^1H NMR (500 MHz, DMSO-d_6): δ 1.03 (3H, t, J = 7.4 Hz, CH_3), 1.79 (2H, sext, J = 7.0 Hz, CH_2), 4.14 (2H, t, J = 6.4 Hz, CH_2), 6.47-6.51 (1H, m, CH), 7.21 (1H, dd, J = 8.2 Hz, 1.6 Hz, CH), 7.34-7.49 (5H, m 5×CH), 7.84-7.88 (1H, m, CH), 11.19 (1H, br s, NH). ^{13}C NMR (125 MHz, DMSO-d_6): δ 10.5 (CH_3), 22.1 (CH_2), 69.9 (CH_2), 101.6 (CH), 111.8 (CH), 112.0 (CH), 118.4 (CH), 119.4 (C), 119.5 (CH), 120.4 (CH), 126.3 (CH), 128.2 (C), 129.9 (C), 130.4 (C), 135.7 (C), 142.4 (C), 154.1 (C). MS (pos. mode): m/z (%): 286.1/288.2 ($M+H^+$, 100%).

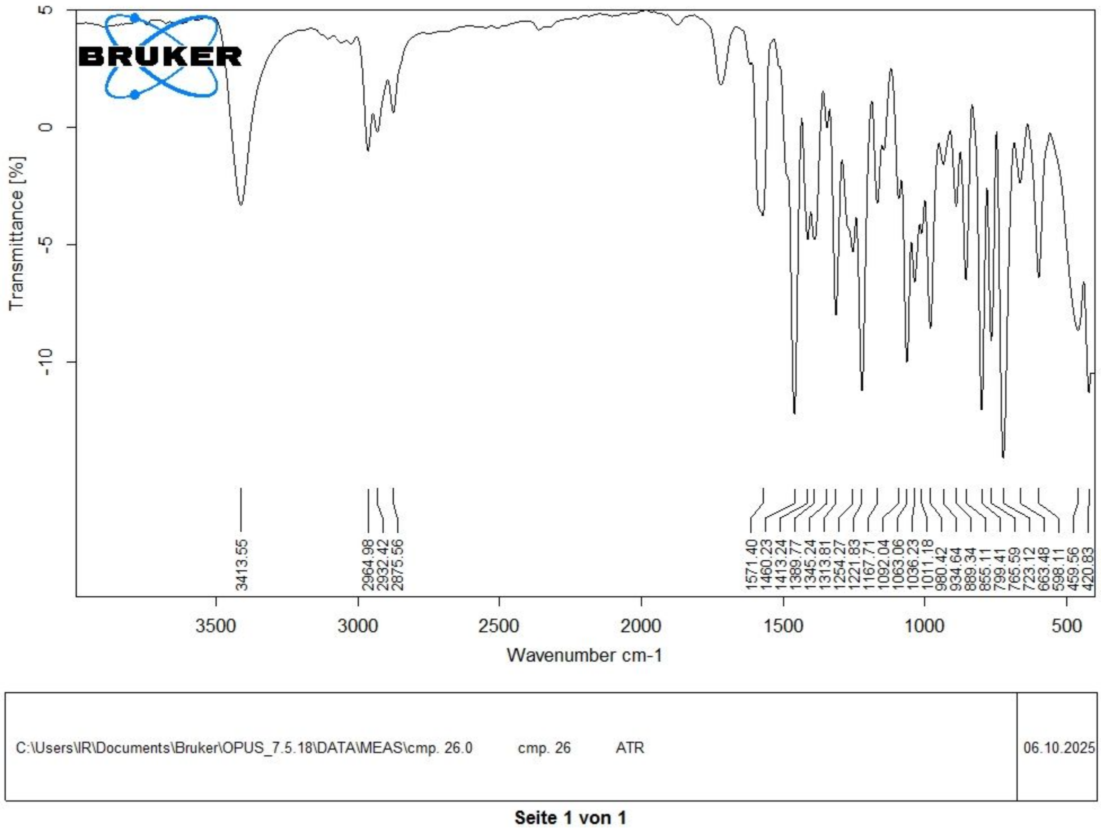

Figure S1.126 FTIR spectrum of compound **i-26**.

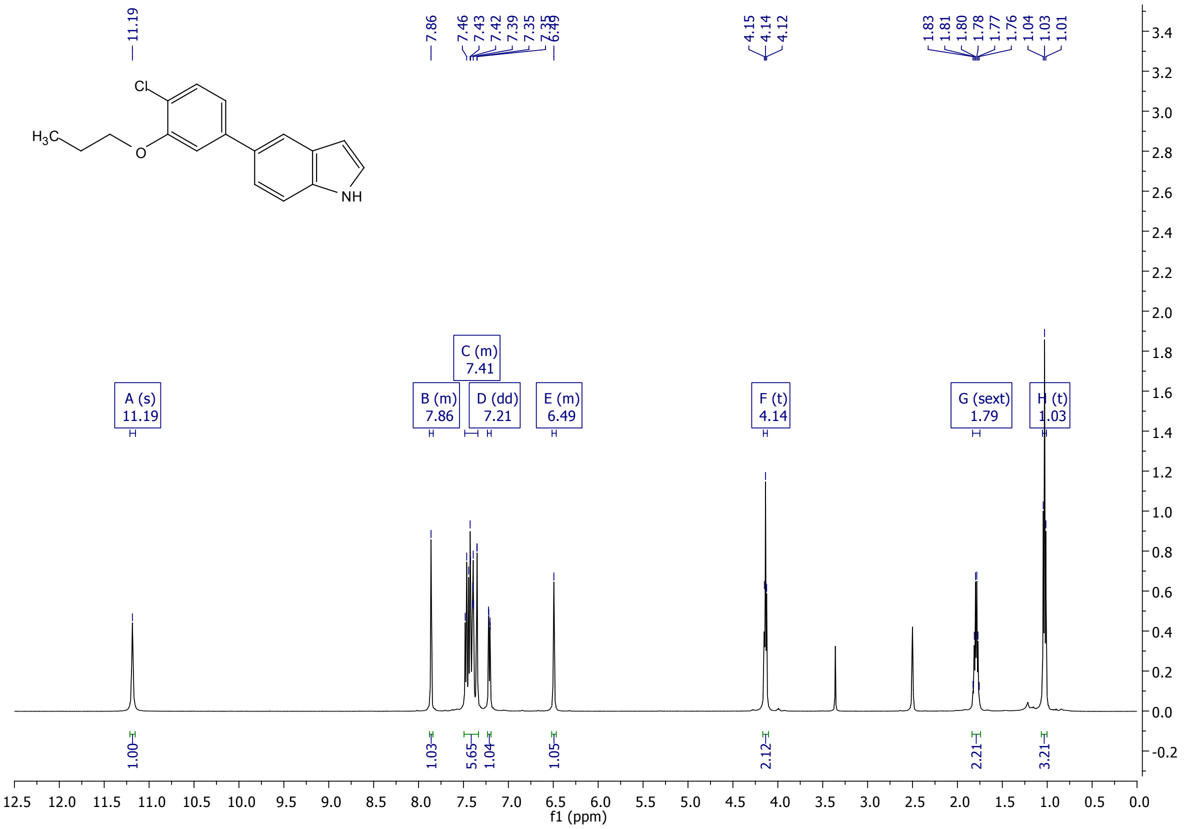

Figure S1.127 ^{1}H NMR spectrum of compound **i-26** in DMSO-d_6.

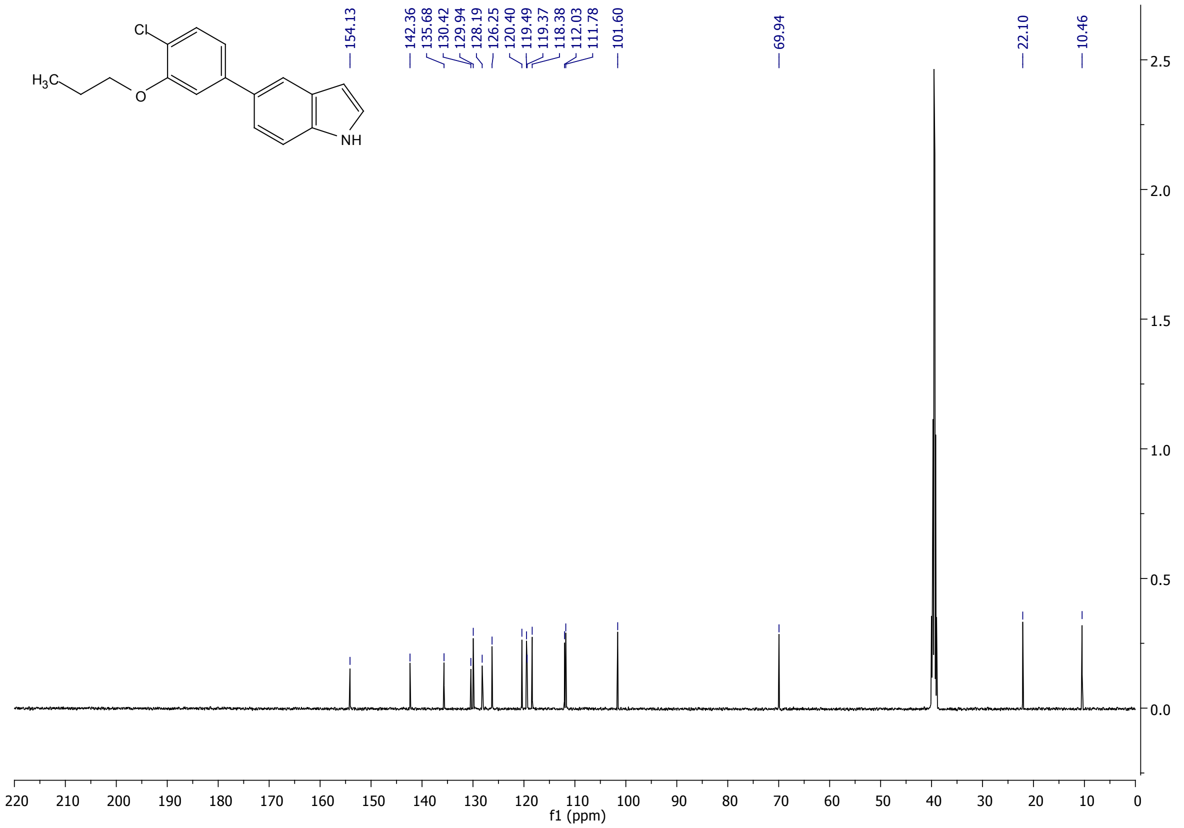

Figure S1.128 ^{13}C NMR spectrum of compound **i-26** in DMSO-d_6.

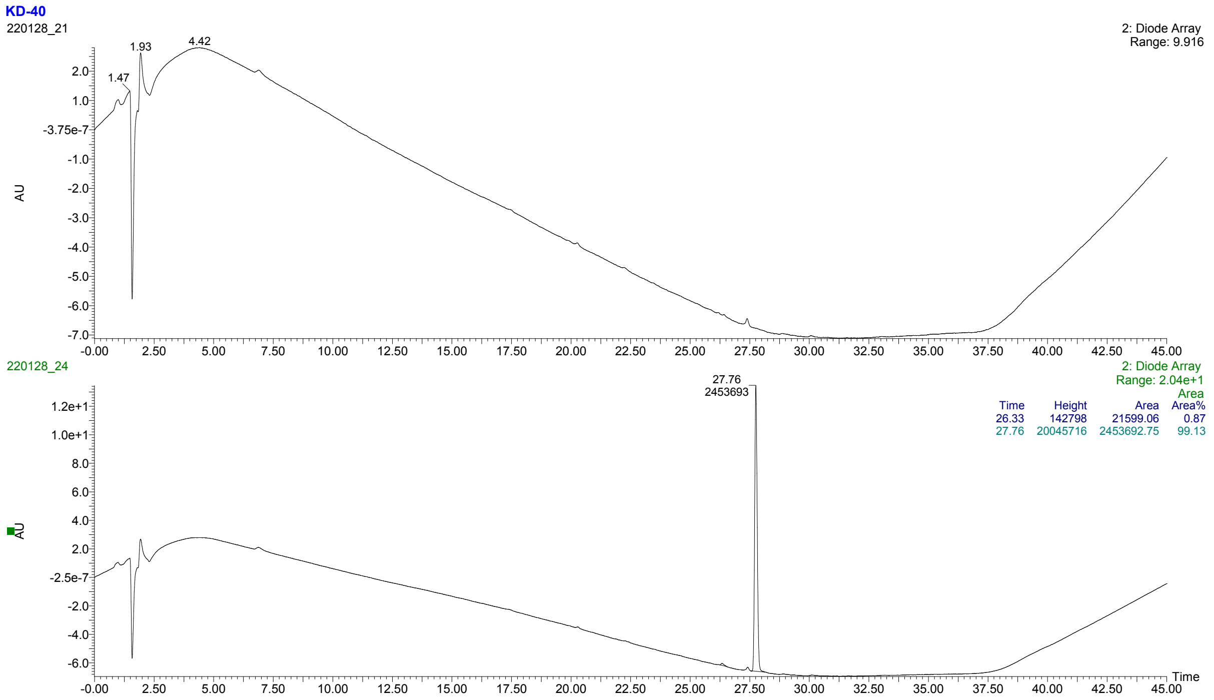

Figure S1.129 LC-MS chromatogram of compound **i-26**.

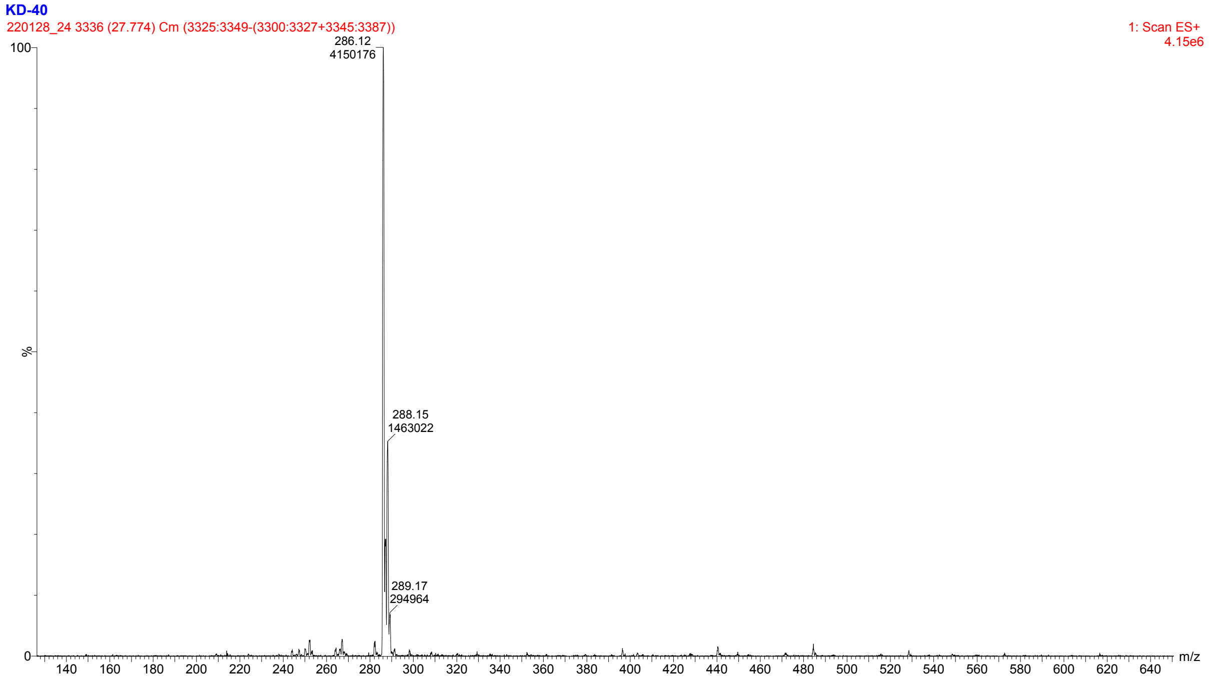

Figure S1.130 MS spectrum of compound **i-26**.

5-(4-Chloro-3-isopropoxyphenyl)-1*H*-indole **i-27**

Prepared in accordance with general procedure **II** from 2-chloro-5-(1*H*-indol-5-yl)phenol **i-24** (47 mg, 0.19 mmol) and 2-iodopropane (29 µL, 0.29 mmol). Yield 65%, pale yellow viscous oil, R_f = 0.33 (*n*-hexane/ethyl acetate, 4/1). IR (neat) ν_{max}, cm^{-1}: 3414 (N-H), 2977, 1462, 1313, 1221, 1103, 1061, 967, 800, 766, 726. ^{1}H NMR (500 MHz, DMSO-d_6): δ 1.33 (6H, d, *J* = 6.0 Hz, 2×CH_3), 4.84 (1H, hept, *J* = 6.0 Hz, CH), 6.47-6.50 (1H, m, CH), 7.21 (1H, dd, *J* = 8.3, 2.0 Hz, CH), 7.36-7.41 (3H, m, 3×CH), 7.42-7.48 (2H, m, 2×CH), 7.83-7.85 (1H, m, CH), 11.18 (1H, br s, NH). ^{13}C NMR (125 MHz, DMSO-d_6): δ 21.9 (2×CH_3), 71.1 (CH), 101.6 (CH), 111.8 (CH), 114.1 (CH), 118.4 (CH), 119.8 (CH), 120.4 (CH), 120.6 (C), 126.3 (CH), 128.2 (C), 130.2 (CH), 130.4 (C), 135.7 (C), 142.3 (C), 153.1 (C). MS (pos. mode): m/z (%): 286.1/288.2 (M+H^+, 100%).

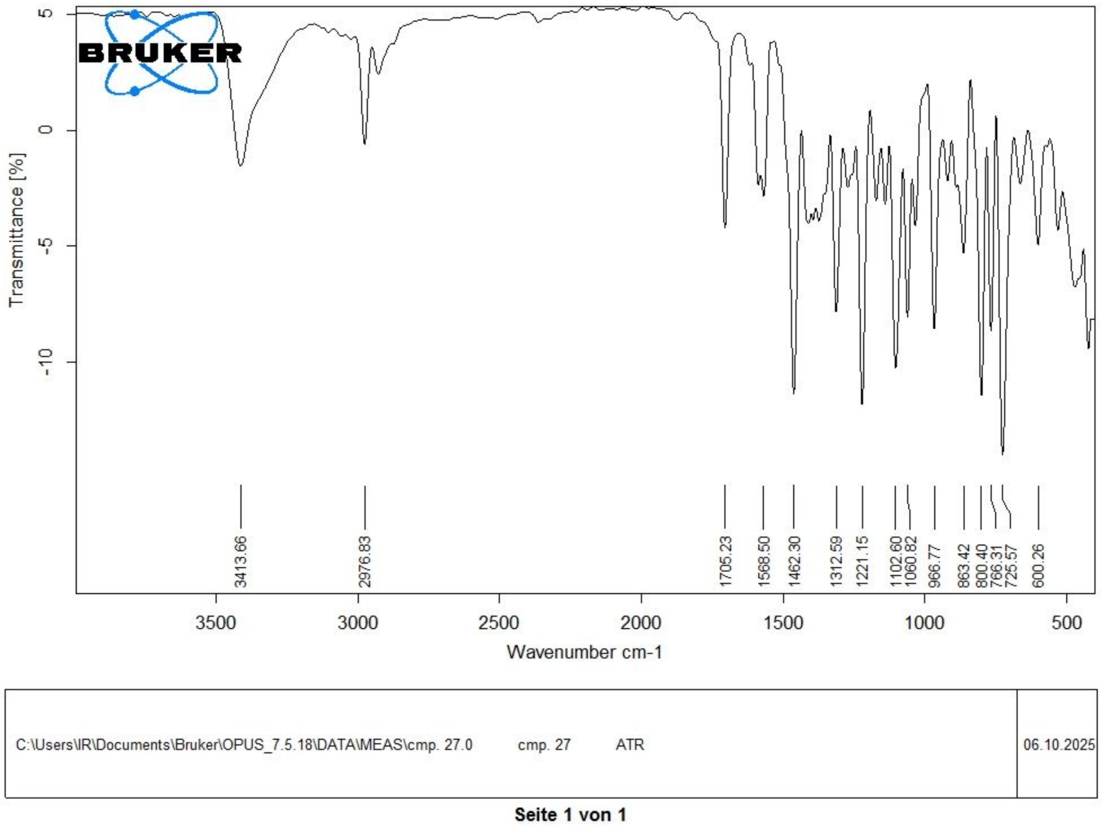

Figure S1.131 FTIR spectrum of compound **i-27**.

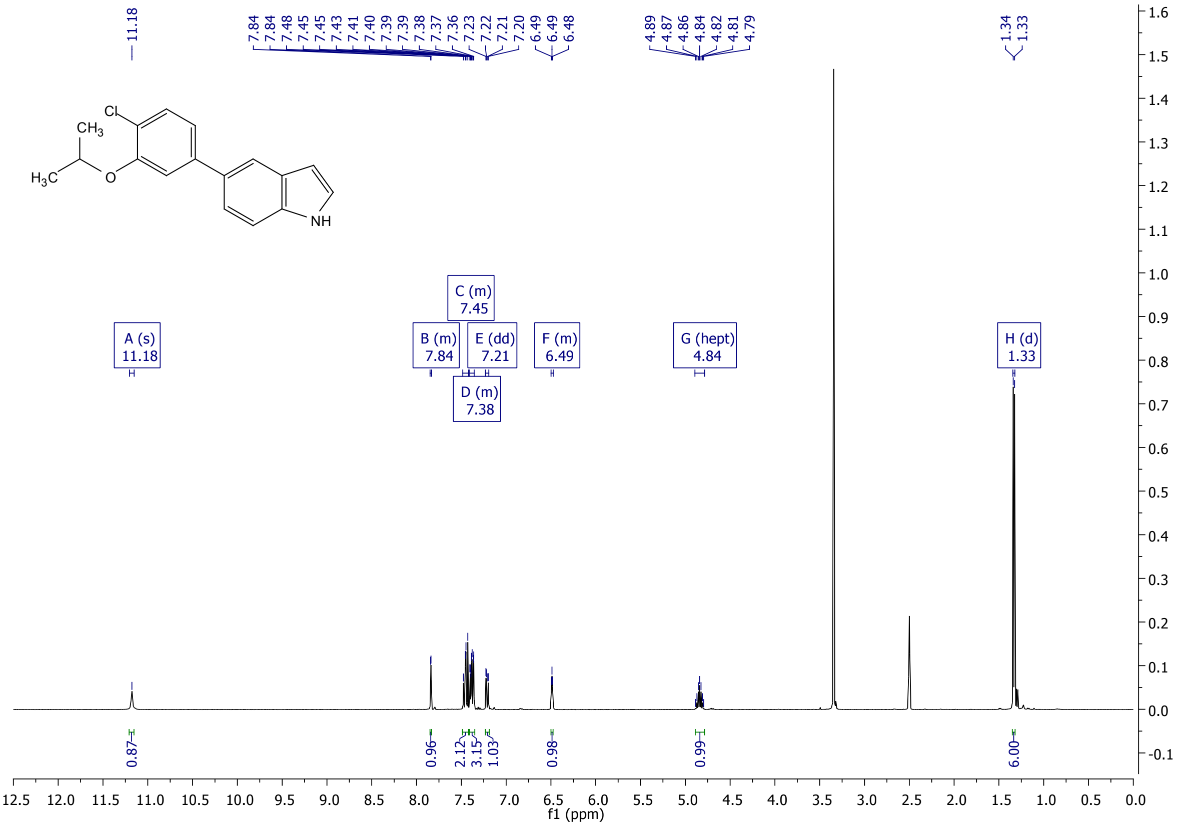

Figure S1.132 ^{1}H NMR spectrum of compound **i-27** in DMSO-d_6.

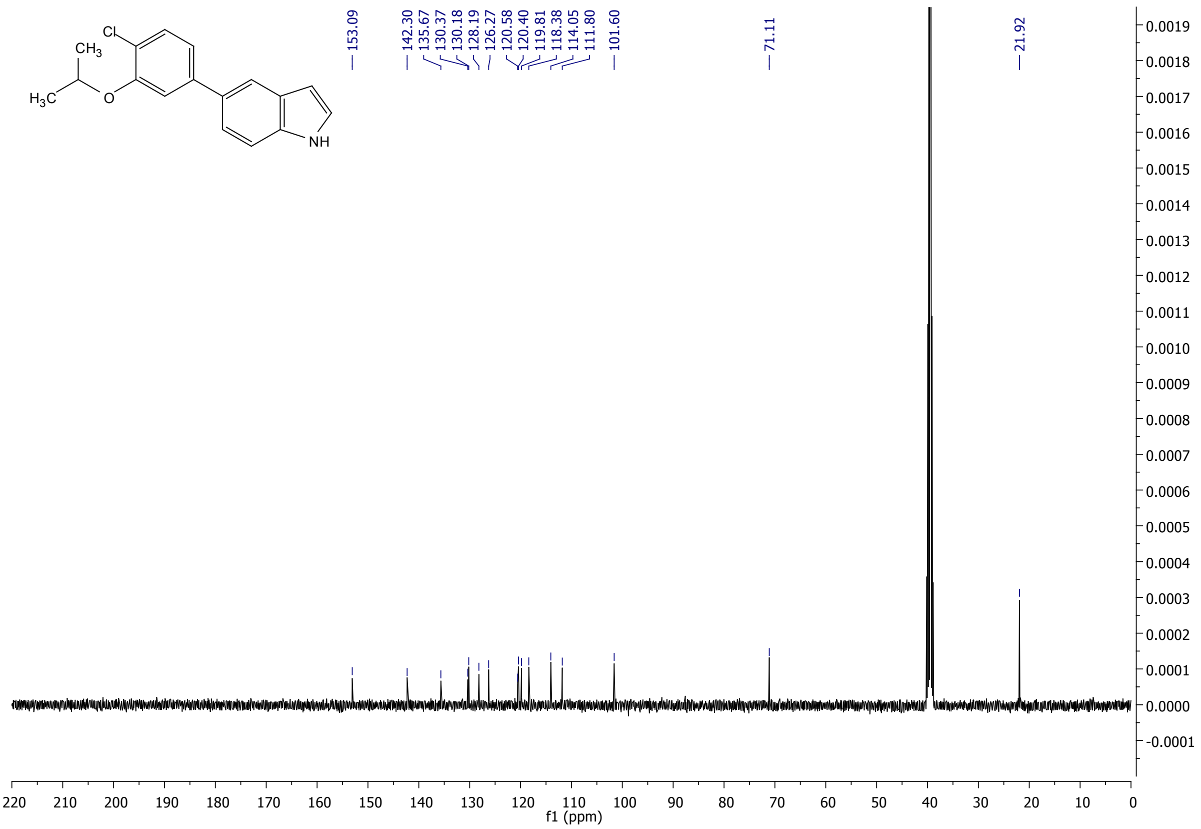

Figure S1.133 ^{13}C NMR spectrum of compound **i-27** in DMSO-d_6.

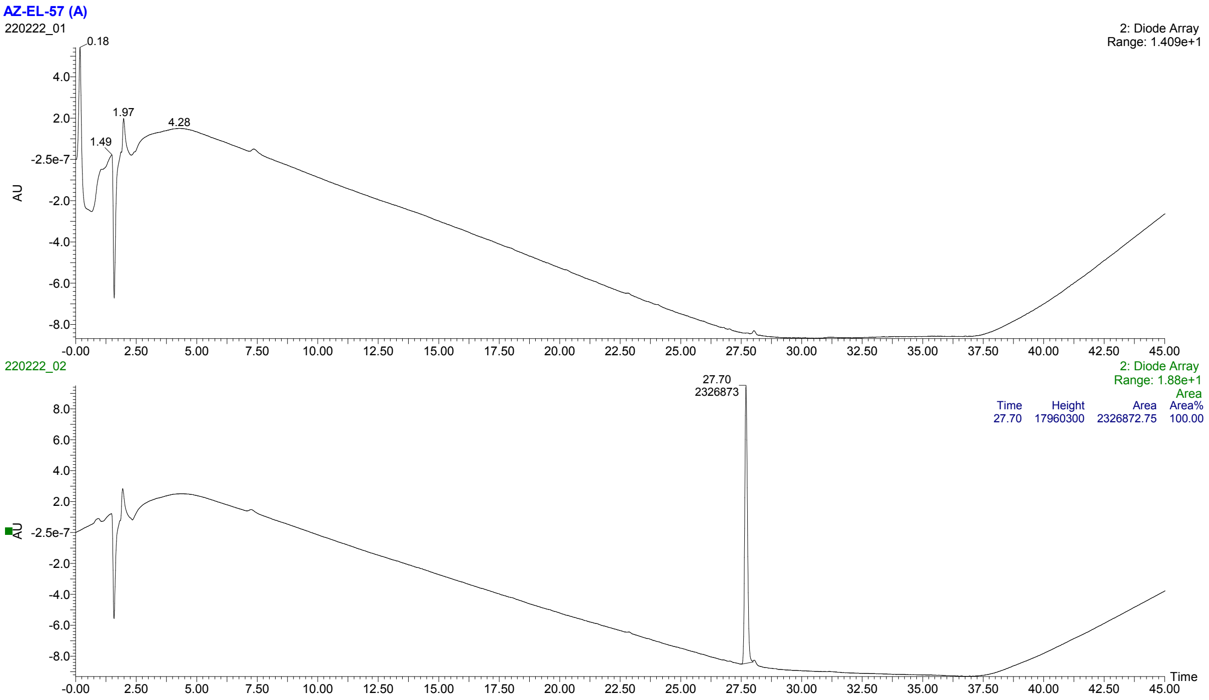

Figure S1.134 LC-MS chromatogram of compound **i-27**.

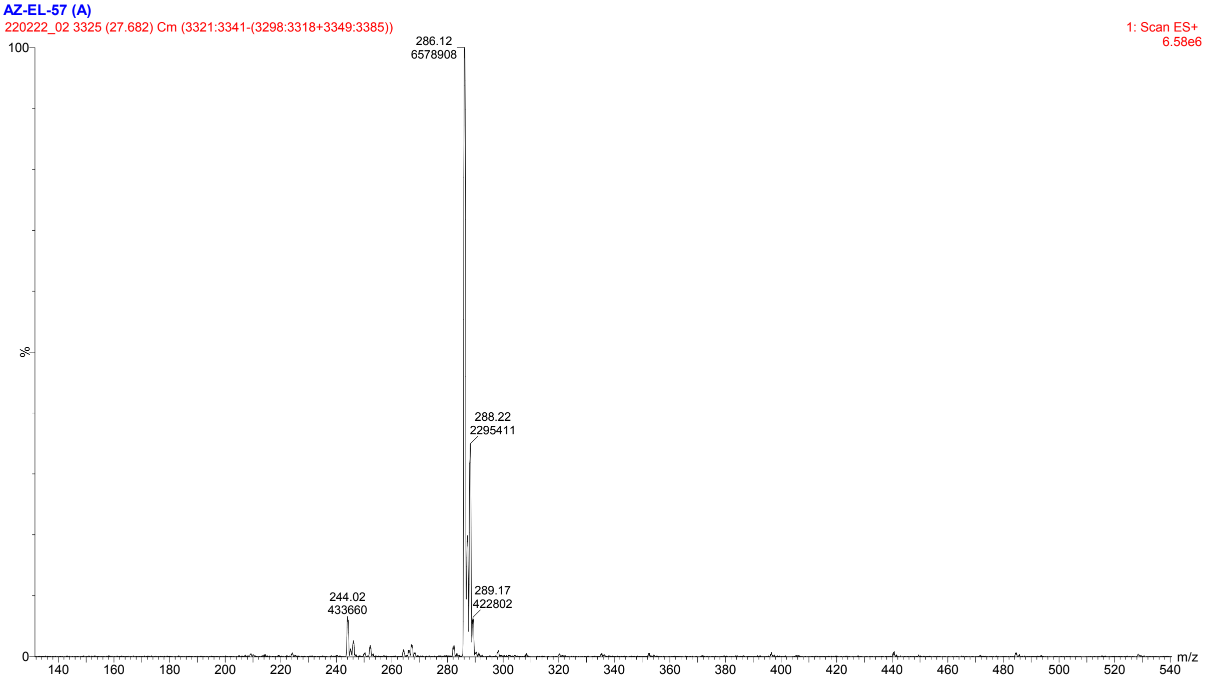

Figure S1.135 MS spectrum of compound **i-27**.

Section S2 – Biology supporting information

Table S2.1 The effect of selected compounds on the motility of age unsynchronized *Ditylenchus destructor*. Numbers indicate average activity counts measured across wells in 3 independent biological experiments (12 wells total), standard error of the mean and P values (repeated measures two-way ANOVA, Dunnett's post-hoc test).

compound	c (µM)	initial			48 h			72 h		
		AVG	SEM	P value	AVG	SEM	P value	AVG	SEM	P value
**i-9**	100	228	12	0.9993	171	19	0.2976	181	14	0.1127
	50	200	19	0.6904	176	18	0.3941	212	7	0.7150
	10	199	21	0.7407	183	16	0.4814	205	7	0.3731
**i-12**	100	235	9	0.9999	207	13	0.9950	202	16	0.7802
	50	201	19	0.7285	186	15	0.5273	202	12	0.5432
	10	220	19	0.9959	197	15	0.9012	218	7	0.9721
**i-13**	100	239	8	0.9997	211	10	0.9989	228	8	0.9997
	50	221	14	0.9897	211	8	0.9960	219	7	0.9863
	10	204	20	0.8376	200	17	0.9724	207	15	0.8875
**i-15**	100	217	12	0.9416	199	13	0.8841	206	15	0.8627
	50	226	13	0.9991	198	17	0.9576	218	15	0.9990
	10	224	14	0.9990	201	16	0.9774	224	7	0.9992
**i-17**	100	245	11	0.9993	206	13	0.9904	212	9	0.7974
	50	228	12	0.9993	223	9	>0.9999	224	8	0.9993
	10	228	15	0.9994	211	9	0.9989	212	11	0.9195
**i-19**	100	233	16	0.9998	113	24	0.0150	88	17	<0.0001
	50	239	12	0.9998	85	22	0.0012	67	23	0.0003
	10	226	16	0.9993	227	13	0.9996	232	9	0.9999
**i-20**	100	226	10	0.9699	2	1	<0.0001	2	1	<0.0001
	50	223	16	0.9897	84	25	0.0016	64	20	0.0009
	10	215	20	0.9480	200	16	0.5447	187	19	0.9993
**i-21**	100	221	15	0.9628	158	16	0.0096	94	13	0.0012
	50	223	6	0.6670	172	17	0.0608	139	20	0.2884
	10	227	9	0.9738	220	13	0.9802	183	17	0.9990
**i-25**	100	230	11	0.9989	210	10	0.4519	203	9	0.9998
	50	246	7	0.9992	209	11	0.5427	197	11	0.9998
	10	234	11	0.9994	221	5	0.7114	194	12	0.9996
**i-27**	100	213	12	0.5732	207	8	0.2022	202	11	0.9999
	50	248	7	0.9958	227	6	0.9950	223	9	0.9505
	10	242	4	0.9997	235	8	0.9999	211	11	0.9993
**controls**	NaN_3 10 mM	223	11	0.8602	7	4	<0.0001	2	1	<0.0001
	water	238	6	0.9597	228	6	0.9990	216	10	>0.9999
	DMSO 0.2%	230	8		231	6		215	6	

Table S2.2 The effect of selected compounds on the motility of *Heterodera schachtii* second-stage juveniles (J2). Numbers indicate average activity counts measured across wells in 3 independent biological experiments (12 wells total), standard error of the mean and P values (repeated measures two-way ANOVA, Dunnett's post-hoc test).

**compound**	**c (µM)**	**initial**			**48 h**			**72 h**		
		AVG	SEM	P value	AVG	SEM	P value	AVG	SEM	P value
**i-9**	100	193	13	0.9996	202	10	0.8881	171	11	0.1658
	50	201	18	0.9991	207	13	0.8051	188	12	0.3472
	10	167	17	0.9815	161	10	0.8912	159	7	0.9912
**i-12**	100	195	12	0.9993	166	21	0.9991	177	9	0.9899
	50	176	17	0.9992	195	7	0.9887	136	16	0.9997
	10	192	15	0.9997	200	9	0.9267	158	13	0.9990
**i-13**	100	192	11	0.9996	144	13	0.3501	150	14	0.9954
	50	192	14	0.9997	183	13	>0.9999	163	14	0.9989
	10	166	15	0.9514	159	10	0.8041	141	14	0.9994
**i-15**	100	195	10	0.9993	212	9	0.4573	175	11	0.4797
	50	188	13	>0.9999	193	7	0.9912	144	10	0.9991
	10	169	16	0.9876	191	8	0.9991	164	14	0.9991
**i-17**	100	197	11	0.9991	158	9	0.7236	122	15	0.9198
	50	181	11	0.9994	158	15	0.9195	158	10	0.9991
	10	178	12	0.9991	160	13	0.9380	139	12	0.9999
**i-19**	100	195	8	0.9993	121	8	0.0058	30	13	<0.0001
	50	183	11	0.9996	90	15	0.0022	18	8	<0.0001
	10	190	10	0.9999	179	13	0.9999	192	6	0.9993
**i-20**	100	180	8	0.4751	4	1	<0.0001	5	3	0.0002
	50	174	10	0.3638	81	17	0.0827	27	8	0.0005
	10	165	10	0.1025	169	10	0.9638	176	15	0.9950
**i-21**	100	187	10	0.9697	150	15	>0.9999	104	17	0.5081
	50	177	9	0.4402	142	12	0.9996	102	19	0.5283
	10	175	12	0.5588	170	12	0.9681	146	14	0.9996
**i-25**	100	172	11	0.3584	195	9	0.1702	190	11	0.7414
	50	178	10	0.5354	184	8	0.4529	170	9	0.9990
	10	174	9	0.2862	160	15	0.9993	171	12	0.9990
**i-27**	100	169	13	0.3897	161	19	0.9993	111	18	0.7097
	50	189	7	0.9579	124	26	0.9952	113	25	0.9053
	10	169	12	0.3345	161	16	0.9993	167	12	0.9993
**controls**	NaN_3 10 mM	182	10	0.9393	2	1	<0.0001	2	0	<0.0001
	water	179	9	0.7678	150	13	0.9414	145	13	0.9955
	DMSO 0.2%	194	6		165	10		160	11	

Table S2.3 The effect of selected derivatives on seed germination (measured at 5 DPS) of *Arabidopsis thaliana* Col-0. Numbers indicate average fraction of germinated seeds from 3 independent biological experiments (at least 30 plants total), standard error of the mean and P values (one-way ANOVA, Dunnett's post-hoc test).

compound	c (µM)	AVG	SEM	P value
**i-9**	5	0.96	0.02	0.9946
	10	0.95	0.02	0.9993
	50	0.96	0.01	0.9937
**i-12**	5	0.96	0.02	0.9943
	10	0.93	0.04	0.9999
	50	0.93	0.01	0.9997
**i-13**	5	0.98	0.02	0.9135
	10	0.93	0.04	>0.9999
	50	0.91	0.03	0.9996
**i-15**	5	0.98	0.02	0.9135
	10	0.93	0.02	0.9998
	50	0.95	0.02	0.9992
**i-17**	5	0.95	0.02	0.9992
	10	0.98	0.01	0.8793
	50	0.89	0.03	0.9940
**i-19**	5	0.93	0.04	0.9998
	10	0.96	0.03	0.9943
	50	0.97	0.01	0.9935
	100	0.93	0.04	0.9998
**i-20**	5	0.96	0.02	0.9943
	10	0.91	0.03	0.9996
	50	0.96	0.03	0.9943
	100	0.95	0.00	0.9991
**i-21**	5	0.98	0.02	0.9135
	10	1.00	0.00	0.5923
	50	1.00	0.00	0.5923
	100	0.96	0.01	0.9940
**i-25**	5	0.98	0.02	0.9135
	10	0.95	0.03	0.9993
	50	0.96	0.03	0.9943
**i-27**	5	0.94	0.03	0.9993
	10	0.98	0.01	0.9017
	50	0.95	0.02	0.9991
**control**	DMSO 0.3%	0.92	0.01	

Table S2.4 The effect of selected derivatives on main root length (measured at 10 DPS) of *Arabidopsis thaliana* Col-0. Numbers indicate average value in mm from 3 independent biological experiments (at least 30 plants total), standard error of the mean and P values (one-way ANOVA, Dunnett's post-hoc test).

compound	c (µM)	AVG	SEM	P value
**i-9**	5	40.21	0.92	>0.9999
	10	41.46	0.80	>0.9999
	50	35.05	0.72	0.6201
**i-12**	5	37.25	0.89	>0.9999
	10	38.66	1.17	>0.9999
	50	35.82	0.80	>0.9999
**i-13**	5	40.24	1.17	>0.9999
	10	39.42	1.06	>0.9999
	50	36.26	1.04	>0.9999
**i-15**	5	36.67	0.69	>0.9999
	10	36.13	1.04	>0.9999
	50	35.01	1.00	0.6446
**i-17**	5	38.83	0.94	>0.9999
	10	37.53	1.01	>0.9999
	50	31.19	1.01	0.0002
**i-19**	5	38.83	0.91	>0.9999
	10	37.74	0.98	>0.9999
	50	33.27	0.71	0.0089
	100	19.96	0.59	<0.0001
**i-20**	5	39.53	0.95	>0.9999
	10	34.00	0.92	0.1180
	50	31.49	0.76	0.0001
	100	25.82	0.79	<0.0001
**i-21**	5	41.56	0.94	>0.9999
	10	42.21	1.03	>0.9999
	50	35.82	0.77	>0.9999
	100	30.90	0.74	<0.0001
**i-25**	5	38.29	1.18	>0.9999
	10	38.15	0.89	>0.9999
	50	34.25	0.80	0.1443
**i-27**	5	39.90	1.07	>0.9999
	10	40.35	0.92	>0.9999
	50	37.27	0.88	>0.9999
**control**	DMSO 0.3%	38.27	1.04	

Table S2.5 The effect of selected compounds on the plant weight (measured at 10 DPS) of *Arabidopsis thaliana* Col-0. Numbers indicate average value in mg from 3 independent biological experiments (at least 30 plants total), standard error of the mean and P values (one-way ANOVA, Dunnett's post-hoc test).

compound	c (µM)	AVG	SEM	P value
**i-9**	5	33.97	2.60	0.9995
	10	35.69	1.89	>0.9999
	50	31.4	4.65	0.9947
**i-12**	5	32.51	3.47	0.9990
	10	34.91	3.82	0.9997
	50	27.55	2.07	0.7448
**i-13**	5	37.8	4.82	0.9995
	10	36.95	4.92	0.9997
	50	26.29	2.23	0.5483
**i-15**	5	35.21	2.85	0.9998
	10	31.55	3.91	0.9951
	50	29.94	2.05	0.9817
**i-17**	5	35.01	4.45	0.9997
	10	28.28	3.47	0.8473
	50	26.18	5.45	0.5315
**i-19**	5	32.28	3.74	0.9989
	10	30.72	3.97	0.9932
	50	24.85	3.32	0.3479
	100	15.22	0.91	0.0022
**i-20**	5	30.62	1.92	0.9930
	10	27.14	1.91	0.6809
	50	24.67	3.76	0.3262
	100	16.94	2.39	0.0068
**i-21**	5	34.37	1.59	0.9996
	10	36.56	5.16	0.9999
	50	24.65	0.87	0.3243
	100	21.97	1.52	0.1066
**i-25**	5	30.25	3.96	0.9854
	10	31.97	4.38	0.9988
	50	27.79	1.97	0.7796
**i-27**	5	33.97	4.56	0.9995
	10	35.66	5.18	>0.9999
	50	30.34	2.18	0.9860
**control**	DMSO 0.3%	35.95	4.64	

Table S2.6 Cytotoxicity of selected compounds in cell lines derived from human skin.

compound	c (µM)	Average viability (signal intensity relative to control) from at least 3 independent experiments (9 wells total) ± SEM	
		**BJ**	**HaCaT**
**i-3**	10	1 ± 0.01	1.02 ± 0.06
	20	0.97 ± 0	1 ± 0.06
	40	0.89 ± 0	0.93 ± 0.06
**i-9**	10	0.92 ± 0.03	0.97 ± 0.04
	20	0.97 ± 0.04	1.01 ± 0.05
	40	0.94 ± 0.03	0.99 ± 0.02
**i-12**	10	0.96 ± 0.01	1.06 ± 0.14
	20	1.04 ± 0.05	1.09 ± 0.13
	40	0.96 ± 0.03	1.06 ± 0.09
**i-13**	10	0.97 ± 0.01	0.97 ± 0.01
	20	1 ± 0.05	0.98 ± 0.02
	40	0.98 ± 0.03	0.91 ± 0.04
**i-15**	10	1 ± 0.03	1.01 ± 0.04
	20	1.03 ± 0.04	1.04 ± 0.05
	40	0.98 ± 0.05	1.01 ± 0.05
**i-17**	10	0.9 ± 0.01	0.98 ± 0.01
	20	0.92 ± 0.05	0.99 ± 0.01
	40	0.82 ± 0.07	0.87 ± 0.04
**i-19**	10	0.98 ± 0.02	1.01 ± 0.02
	20	1.02 ± 0.06	1.02 ± 0.05
	40	1 ± 0.03	0.86 ± 0.1
**i-20**	10	0.96 ± 0.01	0.97 ± 0.01
	20	0.98 ± 0.02	0.99 ± 0.02
	40	0.96 ± 0.03	0.94 ± 0.02
**i-21**	10	0.94 ± 0.02	0.96 ± 0.02
	20	0.96 ± 0.04	0.96 ± 0.04
	40	0.87 ± 0.03	0.74 ± 0.11
**i-25**	10	0.92 ± 0.01	0.93 ± 0.02
	20	0.9 ± 0.05	0.88 ± 0.06
	40	0.74 ± 0.06	0.53 ± 0.1
**i-27**	10	0.92 ± 0.02	0.78 ± 0.12
	20	0.92 ± 0.03	0.6 ± 0.19
	40	0.89 ± 0.03	0.48 ± 0.21

Table S2.7 Binding energies of the best hits based on the docking studies to the quinone binding sites of two crystal structures of *Ascaris suum* SDH and complementary *Caenorhabditis elegans* models.

**compound**	**Binding energy in quinone binding site of the SDH protein (kcal/mol)**			
	***A. suum* 4YSX**	***A. suum* 3VRB**	***C. elegans* 4YSX model**	***C. elegans* 3VRB model**
fluopyram	-8.3	-9.9	-7.7	-9.8
flutolanil	-7.7	-9.8	-7.2	-9.9
**i-1**	-7.0	-9.2	-7.4	-8.7
**i-2**	-7.4	-9.0	-6.9	-8.5
**i-3**	-7.2	-9.3	-7.0	-8.7
**i-4**	-6.9	-8.8	-7.3	-8.5
**i-5**	-7.5	-9.0	-7.2	-8.5
**i-6**	-7.3	-9.2	-7.9	-8.6
**i-7**	-7.0	-8.4	-7.9	-8.6
**i-8**	-7.5	-8.2	-7.2	-8.0
**i-9**	-7.7	-9.1	-7.9	-8.7
**i-10**	-6.9	-8.6	-6.4	-8.7
**i-11**	-7.9	-8.6	-6.6	-8.0
**i-12**	-7.5	-9.0	-6.8	-8.9
**i-13**	-6.9	-8.7	-6.4	-8.9
**i-14**	-7.3	-8.9	-7.3	-8.2
**i-15**	-7.1	-8.7	-7.1	-8.6
**i-16**	-7.0	-9.0	-6.8	-9.1
**i-17**	-7.5	-9.0	-7.1	-9.5
**i-18**	-7.4	-9.3	-7.3	-8.8
**i-19**	-7.2	-9.1	-7.6	-9.0
**i-20**	-7.2	-8.9	-7.5	-8.9
**i-21**	-7.2	-9.3	-7.1	-9.3
**i-22**	-7.3	-9.1	-7.3	-8.9
**i-23**	-7.2	-8.5	-6.5	-9.3
**i-24**	-7.5	-8.9	-6.7	-9.1
**i-25**	-7.0	-8.6	-6.6	-8.7
**i-26**	-7.2	-8.6	-6.6	-9.0
**i-27**	-7.1	-8.4	-6.9	-8.5

Table S2.8 The effect of **i-19** and fluopyram on developmental rates of *Caenorhabditis elegans* wild-type and complex II mutants. Numbers indicate mean score across wells in 3 independent biological experiments (9 wells total), standard deviations of the mean and adjusted P values (two-way ANOVA, Dunnett's post-hoc test).

**compound**	**c (µM) / strain**	**N2**			**RP2699**				**RP2700**				**RP2702**			
		**mean**	**SD**	**N**	**mean**	**SD**	**N**	**Adjusted P Value**	**mean**	**SD**	**N**	**Adjusted P Value**	**mean**	**SD**	**N**	**Adjusted P Value**
fluopyram	100	1	0	9	1	0	9	>0.9999	1	0	9	>0.9999	1	0	9	>0.9999
	50	1	0	9	1.222	0.629	9	0.6411	1	0	9	>0.9999	1	0	9	>0.9999
	25	1	0	9	1	0	9	>0.9999	1.333	0.471	9	0.325	1	0	9	>0.9999
	12.5	1	0	9	1	0	9	>0.9999	1	0	9	>0.9999	1	0	9	>0.9999
	6.25	1	0	9	1.222	0.416	9	0.6411	1.222	0.416	9	0.6411	2.333	0.667	9	>0.9999
	3.125	1	0	9	3.333	0.471	9	<0.0001	2.444	0.832	9	<0.0001	3.111	0.737	9	<0.0001
	1.563	1	0	9	3.111	0.875	9	<0.0001	3.667	0.471	9	<0.0001	3.556	0.497	9	<0.0001
	0.781	1	0	9	3.889	0.314	9	<0.0001	3.556	0.497	9	<0.0001	4.111	0.314	9	<0.0001
	0.391	2	0.817	9	4.444	0.497	9	<0.0001	4.444	0.685	9	<0.0001	4.667	0.471	9	<0.0001
	0.195	2.778	1.133	9	4.556	0.497	9	<0.0001	4.778	0.416	9	<0.0001	4.889	0.314	9	<0.0001
	0.098	3.667	0.943	9	4.667	0.471	9	<0.0001	4.889	0.314	9	<0.0001	5	0	9	<0.0001
	0.049	3.667	0.943	9	4	0.817	9	0.325	5	0	9	<0.0001	5	0	9	<0.0001
	0.024	4.333	0.817	9	4.444	0.685	9	0.9296	5	0	9	0.0098	5	0	9	0.0098
	0.012	4.667	0.471	9	4.667	0.471	9	>0.9999	5	0	9	0.325	5	0	9	0.325
**i-19**	100	1	0	9	1	0	9	>0.9999	1	0	9	>0.9999	1	0	9	>0.9999
	75	1	0	9	1	0	9	>0.9999	1	0	9	>0.9999	1	0	9	>0.9999
	56.25	1	0	9	1	0	9	>0.9999	1	0	9	>0.9999	1	0	9	>0.9999
	42.188	1	0	9	1	0	9	>0.9999	1	0	9	>0.9999	1	0	9	>0.9999
	31.641	1	0	9	2.111	0.994	9	<0.0001	1.111	0.314	9	0.9296	1.111	0.314	9	0.9296
	23.731	1.333	0.471	9	2.778	1.133	9	<0.0001	3.444	1.165	9	<0.0001	3.889	0.737	9	<0.0001
	17.798	2.556	0.956	9	3.111	0.875	9	0.0397	3.667	0.667	9	<0.0001	4.667	0.471	9	<0.0001
	13.348	4	0.817	9	4.111	0.875	9	0.9296	4.333	0.471	9	0.325	4.889	0.314	9	0.0003
	10.011	4.444	0.685	9	4.333	0.667	9	0.9296	4.667	0.667	9	0.6411	4.444	0.832	9	>0.9999
	7.509	4.667	0.667	9	4.444	0.497	9	0.6411	4.667	0.471	9	>0.9999	5	0	9	0.325

	5.631	4.444	0.497	9	4.333	0.667	9	0.9296	4.778	0.416	9	0.325	4.889	0.314	9	0.1279
	4.224	4.667	0.471	9	4.556	0.497	9	0.9296	4.778	0.416	9	0.9296	4.889	0.314	9	0.6411
	3.168	4.889	0.314	9	4.444	0.685	9	0.1279	4.889	0.314	9	>0.9999	5	0	9	0.9296
	2.376	4.889	0.314	9	4.556	0.497	9	0.325	4.667	0.471	9	0.6411	5	0	9	0.9296
	1.782	4.222	0.916	9	4.667	0.471	9	0.1279	4.778	0.416	9	0.0397	5	0	9	0.0019
	1.336	5	0	9	4.667	0.471	9	0.325	4.778	0.416	9	0.6411	5	0	9	>0.9999
controls	ivermectin 1 µM	1	0	9	1.333	0.943	9	0.325	1	0	9	>0.9999	1	0	9	>0.9999
	DMSO 0.2%	5	0	9	4.444	0.497	9	0.0397	4.778	0.416	9	0.6411	5	0	9	>0.9999

Table S2.9 The key ADME parameters computed for standards flutolanil, fluopyram and **i-19-21**.

compound	MW	logP (XLOGP3)	TPSA (Å²)	HBD	HBA	Solubility (mg/ml, ESOL)	ESOL class	GI absorption	BBB permeant	P-gp substrate	CYP1A2i	CYP2C19i	CYP2C9i	CYP2D6i	CYP3A4i	Lipinski viol.	Bioavail. score	Syn. access.
flutolanil	323.31	3.70	38.33	1	5	0.022	Moderately soluble	High	Yes	No	Yes	Yes	Yes	Yes	No	0	0.55	2.15
fluopyram	396.71	4.52	41.99	1	8	0.004	Moderately soluble	Low	No	No	Yes	Yes	Yes	Yes	Yes	0	0.55	2.44
**i-19**	228.70	4.19	12.03	1	0	0.009	Moderately soluble	High	Yes	Yes	Yes	No	No	Yes	Yes	0	0.55	1.71
**i-20**	212.24	3.67	12.03	1	1	0.023	Soluble	High	Yes	Yes	Yes	No	No	Yes	No	0	0.55	1.75
**i-21**	278.25	4.75	21.26	1	4	0.004	Moderately soluble	High	Yes	Yes	Yes	No	No	Yes	Yes	0	0.55	1.91

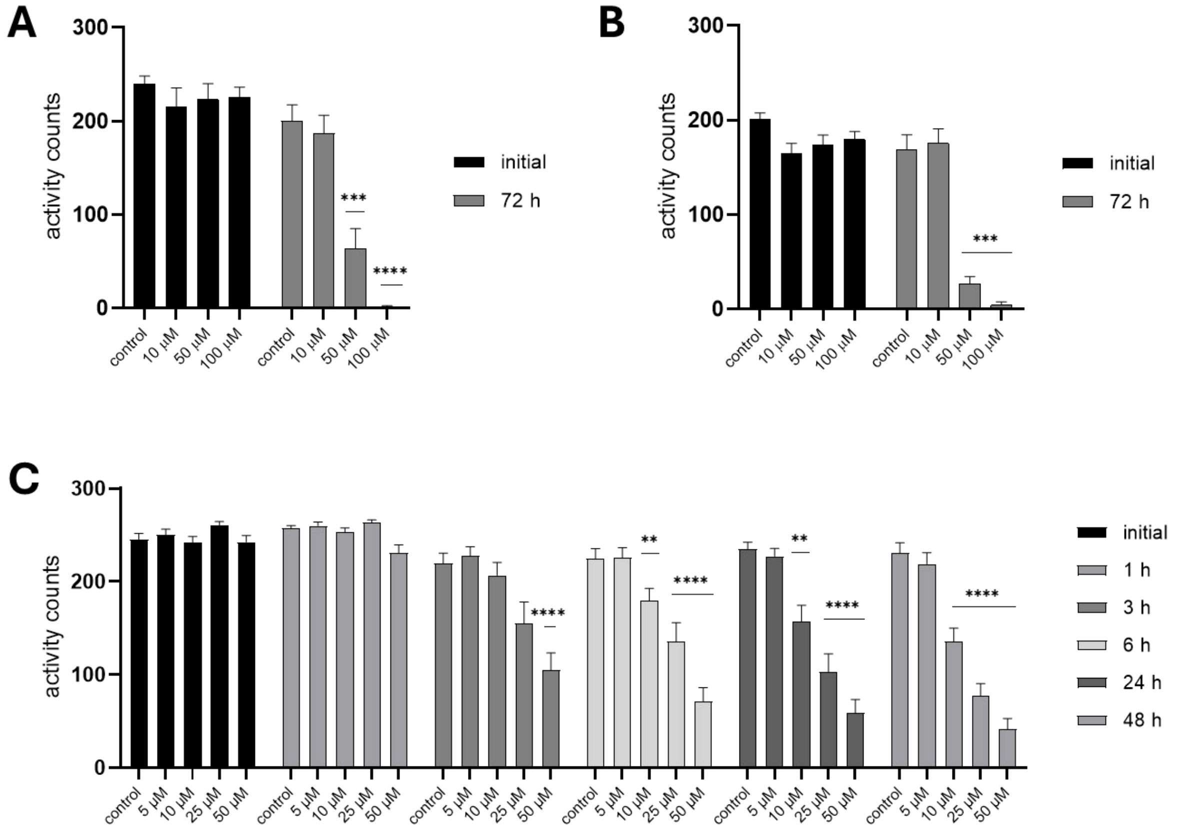

Figure S2.1 The effect of compound **i-20** on the motility of mixed populations of *Ditylenchus destructor* (A), *Heterodera schachtii* J2 (B), and xL3 *Haemonchus contortus* (C) measured by WMicrotracker platform. The graphs show average activity counts +SEM from 3 biological replicates (12 independent wells total). Time point "initial" represents the motility of the population prior to the treatment. Asterisks indicate statistical significance in comparison to vehicle-treated control populations (repeated measures two-way ANOVA with Dunnett's multiple comparison test, * $p < 0.05$, ** $p < 0.01$, *** $p < 0.001$, **** $p < 0.0001$).

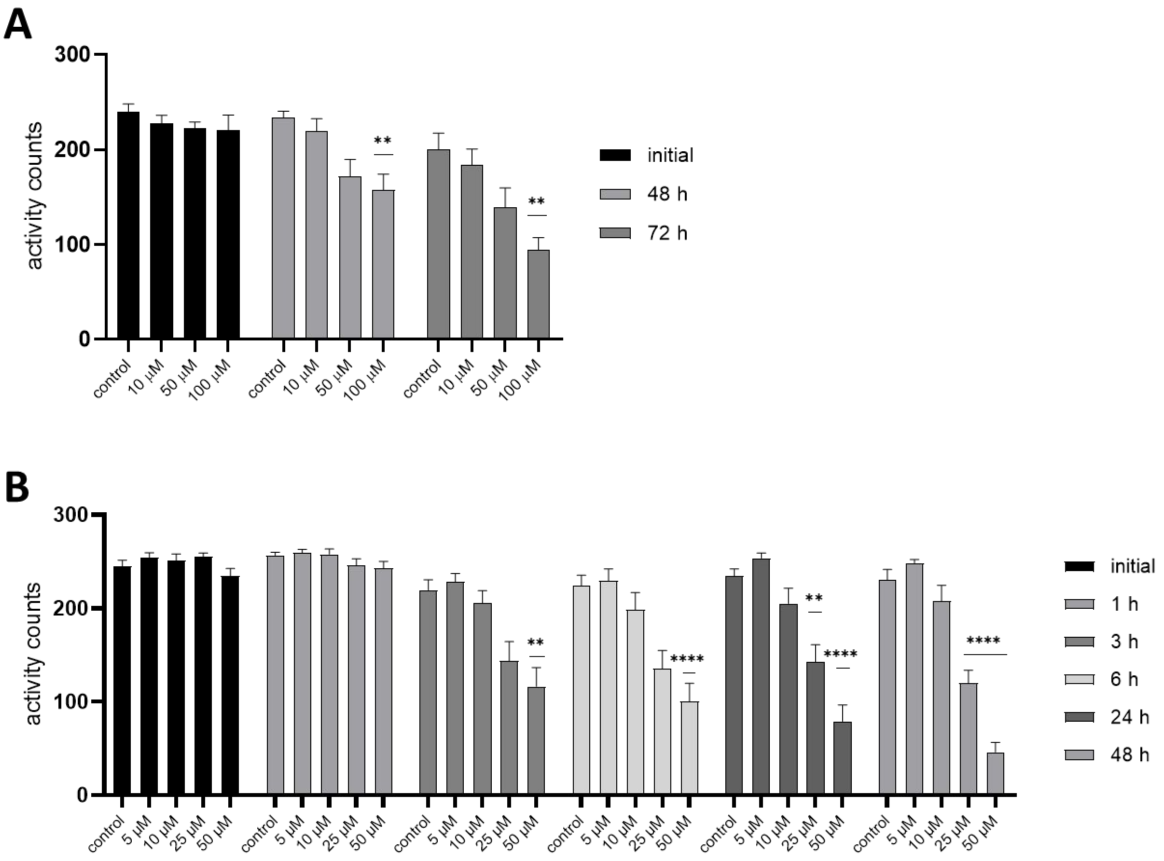

Figure S2.2 The effect **i-21** on the motility of mixed populations of *Ditylenchus destructor* (A), and xL3 *Haemonchus contortus* (B) measured by WMicrotracker platform. The graphs show average activity counts +SEM from 3 biological replicates (12 independent wells total). Time point "initial" represents the motility of the population prior to the treatment. Asterisks indicate statistical significance in comparison to vehicle-treated control populations (repeated measures two-way ANOVA with Dunnett's multiple comparison test, * $p < 0.05$, ** $p < 0.01$, *** $p < 0.001$, **** $p < 0.0001$).

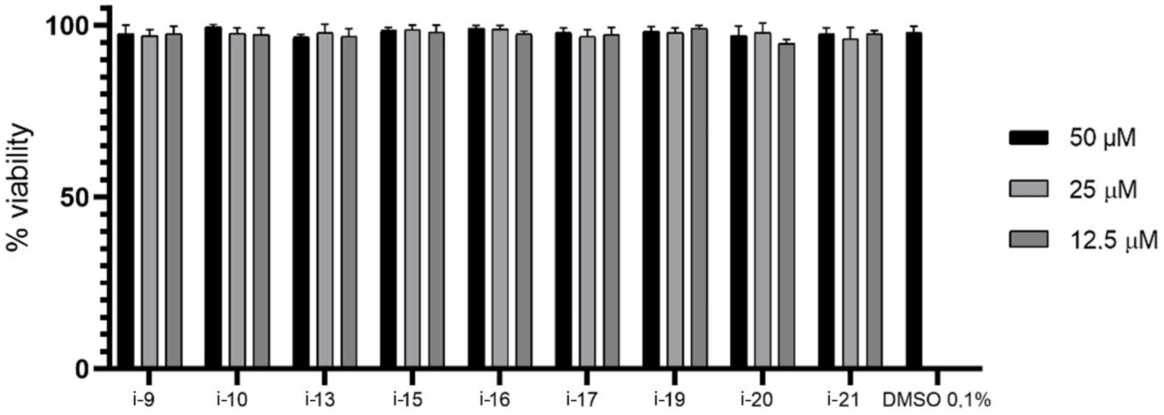

Figure S2.3 The effect of selected derivatives on the viability of *Heterodera schachtii* infective juveniles (J2) evaluated by counting non-curled nematodes under a microscope. The graph shows the average J2 viability from 1 experiment and error bars indicate SEM among the technical replicates (4 wells total).

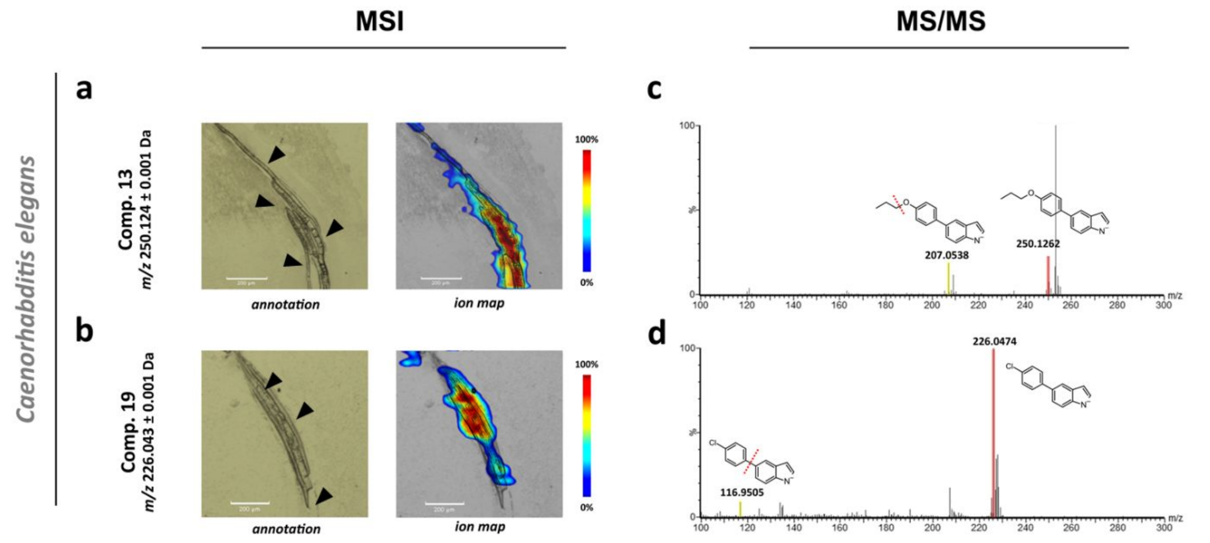

Figure S2.4 The spatial distribution of **i-13** and **i-19** ions observed in C*aenorhabditis elegans* young adults *via* DESI-IMS. Left panel: Ion intensity maps and their annotation images of **i-13** (a) and **i-19** (b). As some nematodes clumped up during the drying process, black arrows on the annotation pictures indicate individual nematodes or fragments of these that broke apart during the spraying process. Right panel: The *in situ* MS/MS spectra of **i-13** (c) and **i-19** (d) with assigned chemical structures.

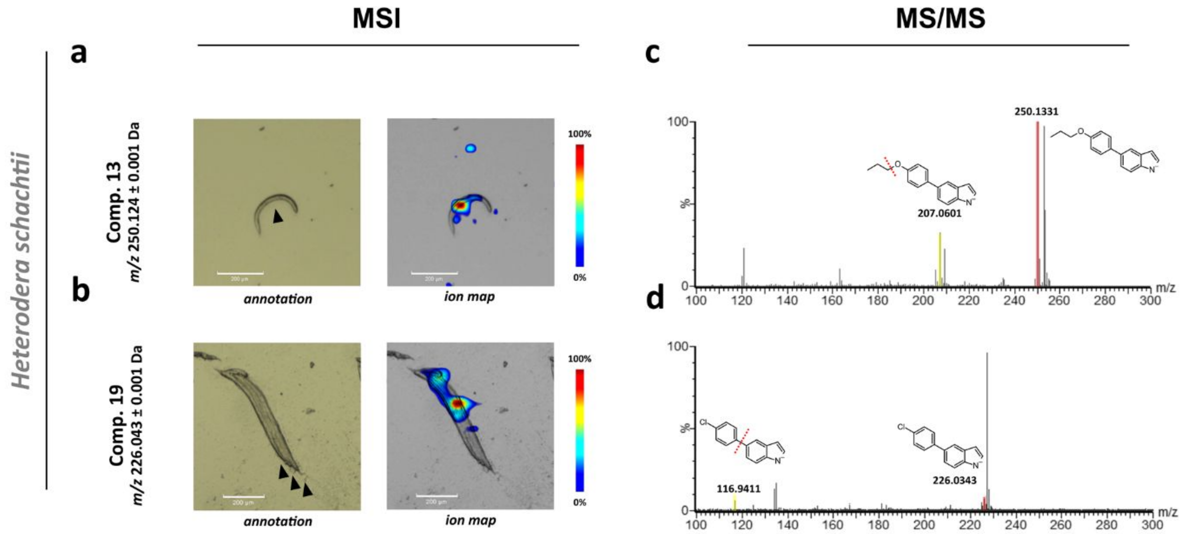

Figure S2.5 The spatial distribution of the selected compound ions observed in *Heterodera schachtii* infective-stage juveniles (J2) *via* DESI-IMS. Left panel: Ion intensity maps and their annotation images of **i-13** (a) and **i-19** (b). As some J2 clumped up during the drying process, black arrows on the annotation pictures indicate individual J2 or fragments of these that broke apart during the spraying process. Right panel: The *in situ* MS/MS spectra of **i-13** (c) and **i-19** (d) with assigned chemical structures.

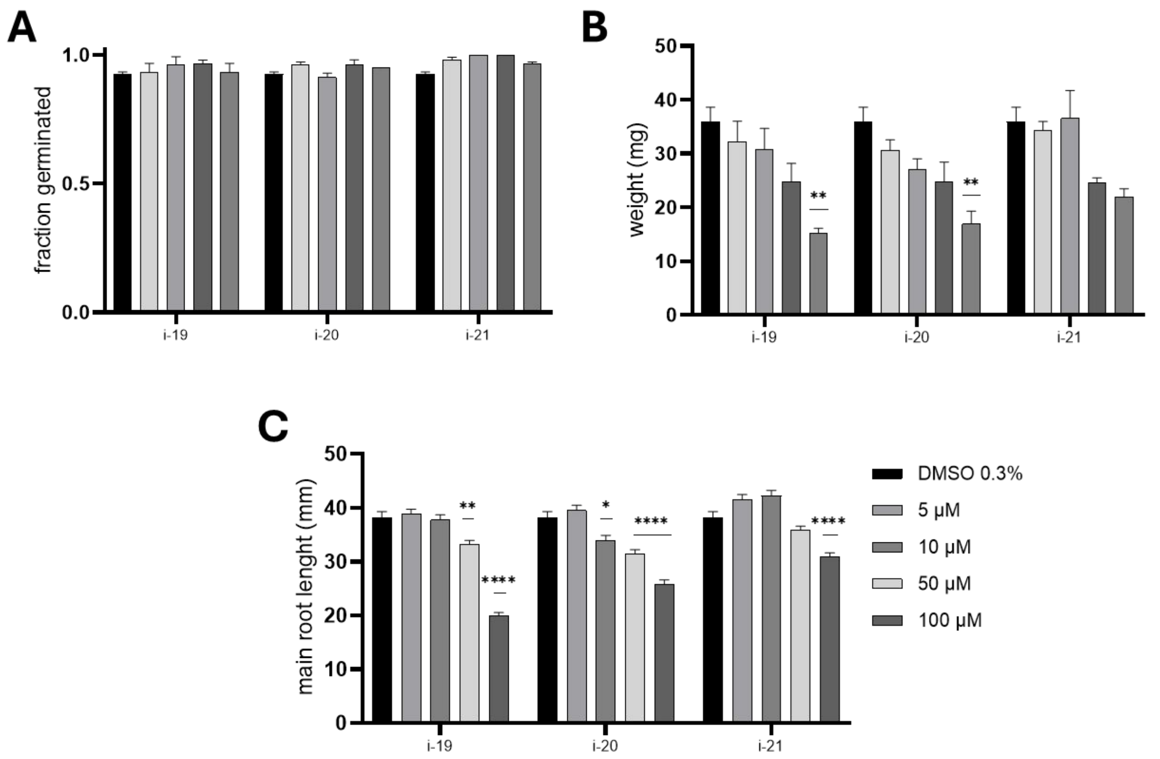

Figure S2.6 The effect of **i-19**, **i-20** and **i-21** on (A) seed germination (evaluated at 5 DPS), (B) plant weight (measured at 10 DPS) and (C) primary root length (measured at 10 DPS) of *Arabidopsis thaliana* Col-0. The graphs show average values from 3 independent experiments (i.e., at least 30 individual plants) and error bars indicate SEM. Asterisks indicate statistical significance in comparison to vehicle-treated control plants (one-way ANOVA with Dunnett's multiple comparison test, * $p < 0.05$, ** $p < 0.01$, *** $p < 0.001$, **** $p < 0.0001$).

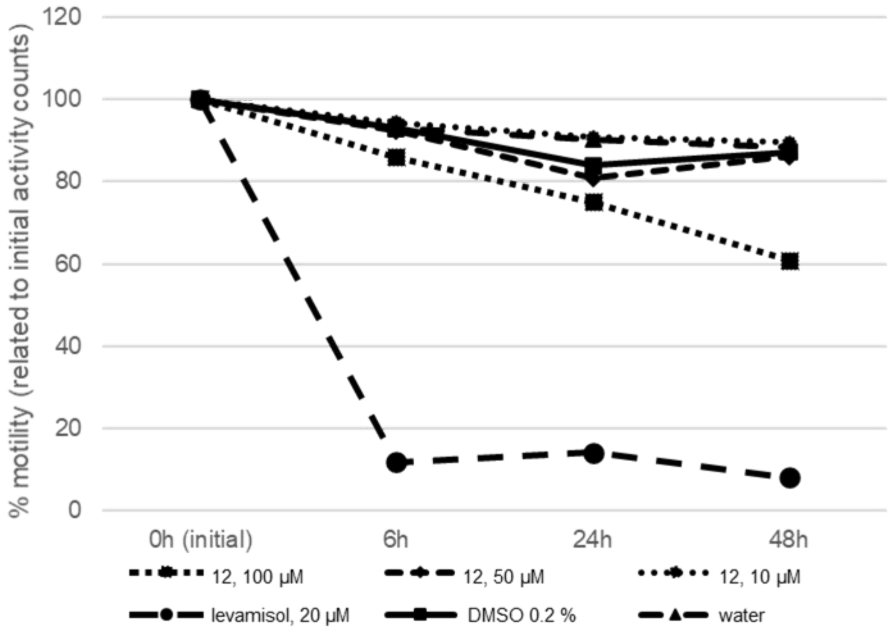

Figure S2.7 The effect of **i-12** on the motility of *Haemonchus contortus* xL3 measured by WMicrotracker. The graph shows results from 1 independent experiment (i.e., values obtained from 4 wells total).

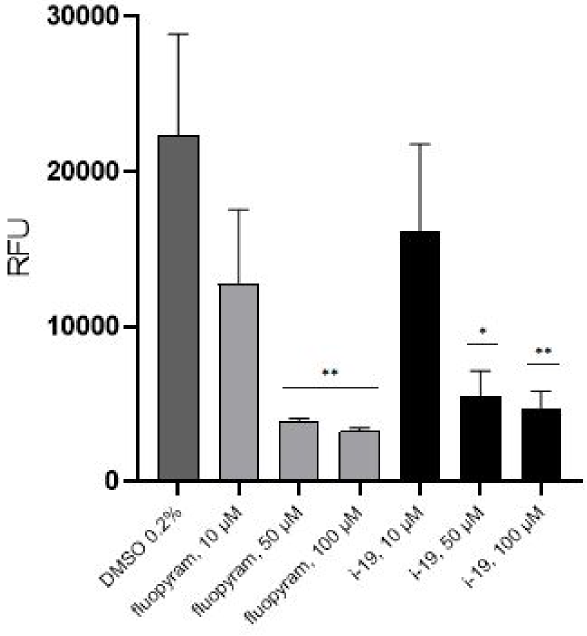

Figure S2.8 The effect of **i-19** and fluopyram on the mitochondrial membrane potential of *Caenorhabditis elegans*. The graphs show average RFU (relative fluorescence unit) of the tracker dye from 3 independent experiments and error bars indicate SEM. Asterisks indicate statistical significance in comparison to negative control (one-way ANOVA with Dunnett's multiple comparison test, * $p < 0.05$, ** $p < 0.01$).

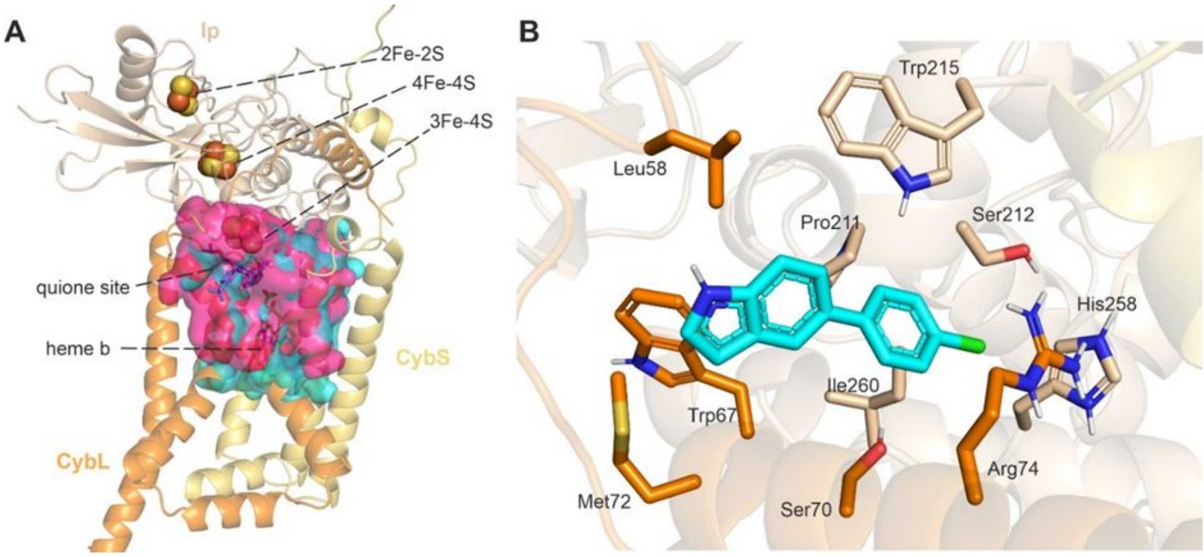

Figure S2.9 (A) *Ascaris suum* crystal structure shown in cartoon (PDB: 3VRB) with binding cavity and flutolanil (magenta) aligned with *A. suum* binding cavity with NN23 (blue) from crystal structure (PDB: 4YSX). The cavities are shown in surface, Ip (chain B), CybL (chain C) and CybS (chain D) subunits are coloured in beige, orange and yellow, respectively. Fe(orange)-S(yellow) clusters are shown as spheres, with S yellow and Fe orange. (B) *Caenorhabditis elegans* homologous model based on 4YSX with **i-19** modelled in the quinone binding with interacting residues shown in sticks.

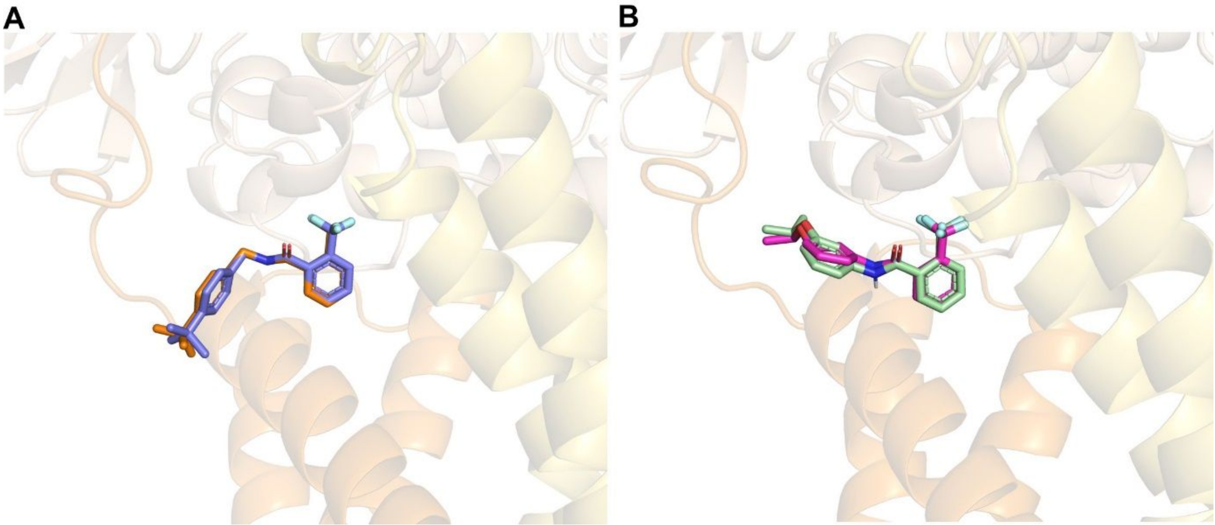

Figure S2.10 (A) Docking validation of the original position of ligand NN23 (violet) in PDB: 4YSX with re-docked NN23 best pose (orange). The RMSD of the ligand positions was equal to 0.546 Å. (B) Docking validation of the original position of ligand flutolanil (magenta) in PDB: 3VRB with re-docked flutolanil best pose (pale green). The RMSD of the ligand positions was equal to 0.425 Å.

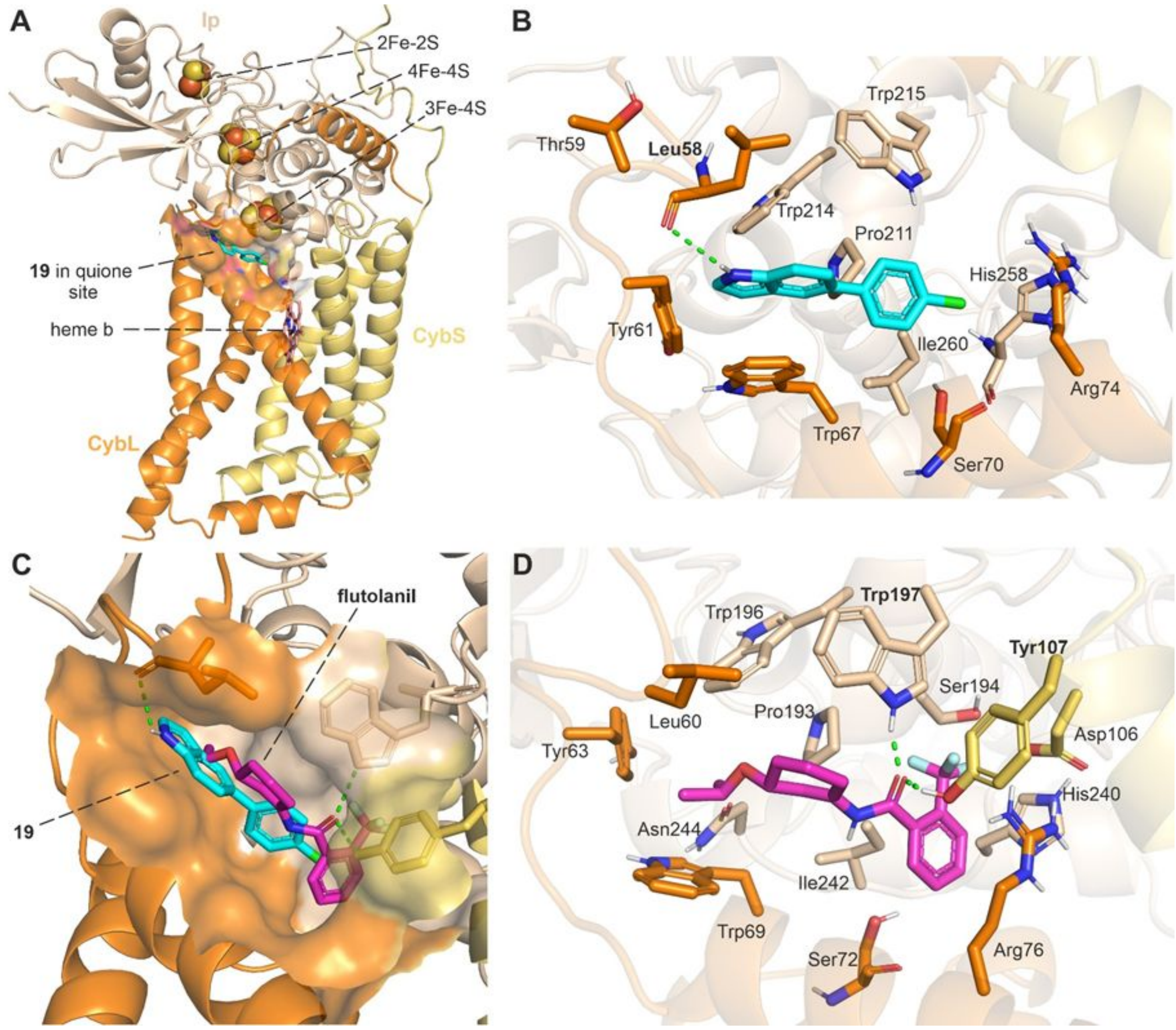

Figure 2.11 (A) Structure of a *Caenorhabditis elegans* homologous model of MCII based on *Ascaris suum* crystal structure (PDB: 3VRB) with modelled **i-19** in the quinone site. Fp (chain A) is omitted and Ip (chain B), CybL (chain C) and CybS (chain D) subunits are coloured in beige, orange and yellow, respectively. Fe(orange)-S(yellow) clusters are shown as spheres. (B) Best pose of **i-19** in the quinone site of the *C. elegans* MCII model (homologous with (PDB: 3VRB)) with interacting residues shown in sticks. (C) Structural alignment of the best pose of **i-19** in the quinone site of the *C. elegans* model (homologous with (PDB: 3VRB)) and original structure of flutolanil in the *A. suum* MCII crystal structure (PDB: 3VRB). (D) Flutolanil in the quinone site with the interacting residues shown in licorice. Hydrogen bonds are shown as green dashed lines and heteroatoms are coloured as follows: nitrogen in blue, oxygen in red, sulphur in yellow and fluorine in pale cyan.

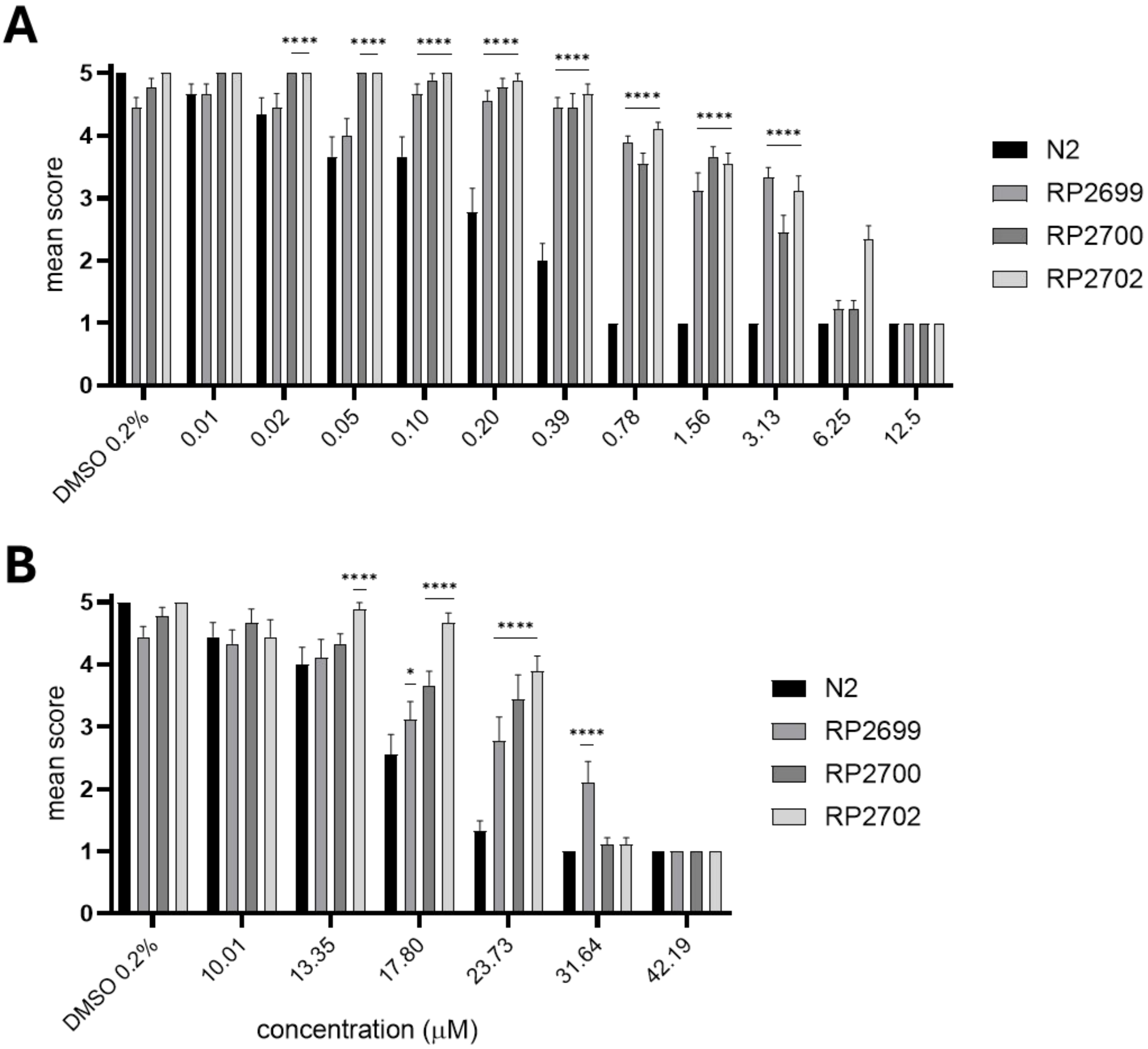

Figure S2.12 *Caenorhabditis elegans* SDH mutants are growing and developing faster than the wild-type after a 5 d exposure to various concentrations of fluopyram (A) and **i-19** (B). The graph shows mean score from 3 independent experiments (9 wells total) and error bars indicate SEM. Asterisks indicate statistical significance in comparison to *C. elegans* wild-type subjected to the same treatment (two-way ANOVA with Dunnett's multiple comparison test, **** $p < 0.0001$).

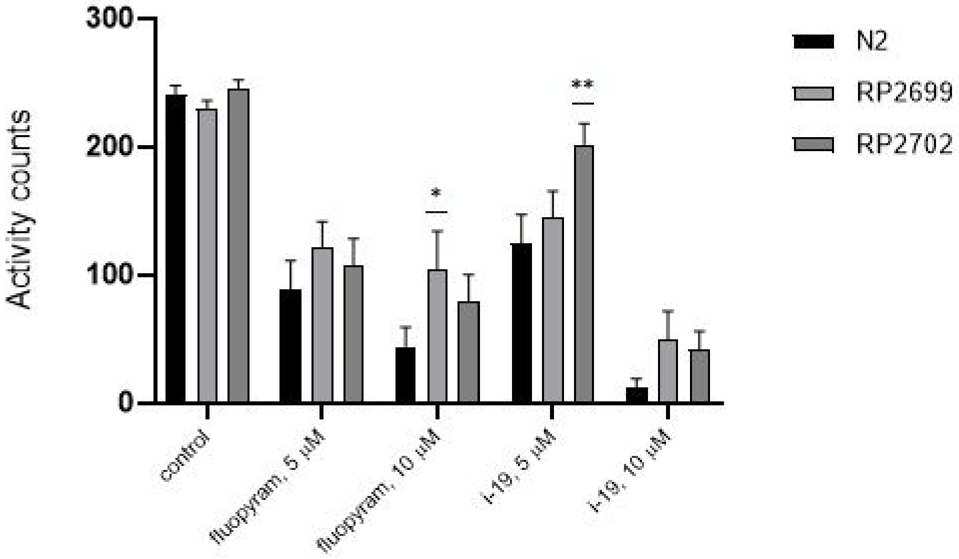

Figure S2.13 Motility of *Caenorhabditis elegans* L1 wild-type and complex II mutants, pre-treated with **i-19** and fluopyram for 72 h, 24 h after the compounds were removed. The graphs show average activity counts from 4 independent experiments (at least 15 wells total) and error bars indicate SEM. Asterisks indicate statistical significance in comparison to negative control (repeated measures two-way ANOVA with Dunnett's multiple comparison test, * $p < 0.05$, ** $p < 0.01$).

REFERENCES

[1] Miao, G., Ye, P., Yu, L., Baldino, C. M. Microwave-promoted Suzuki reactions of aryl chlorides in aqueous media. *J Org Chem* **70**, 2332–2334 (2005).

[2] Bavo, F., Pallavicini, M., Pucci, S., Appiani, R., Giraudo, A., Oh, H., Kneisley, D. L., Eaton, B., Lucero, L., Gotti, C., Clementi, F., Whiteaker, P., Bolchi, C. Sub-nanomolar affinity and selective antagonism at α7 nicotinic receptor by combined modifications of 2-triethylammonium ethyl ether of 4-stilbenol (MG624). *J Med Chem* **66**, 1, 306–332 (2023).

[3] Prieto, M., Zurita, E., Rosa, E., Muñoz, L., Lloyd-Williams, P., Giralt, E. Arylboronic acids and arylpinacolboronate esters in Suzuki coupling reactions involving indoles. Partner role swapping and heterocycle protection. *J Org Chem* **69**, 6812–6820 (2004).

[4] Song, Z., Huang, X., Jiang, S., He, C., Tang, L., Ni, Q., Ma, M., Chen, B., Ma, Y. C(sp^2)−C(sp^2) reductive cross-coupling of triarylphosphines with aryl halides by palladium/nickel co-catalysis. *Org Lett* **24**, 5573–5578 (2022).

[5] Bose, A., Mal, P. Using weak interactions to control C–H mono-nitration of indolines. *Chem Commun* **53**, 11368–11371 (2017).
